# Post-polyploid chromosomal diploidization in plants is affected by clade divergence and constrained by shared genomic features

**DOI:** 10.1038/s41467-026-73793-8

**Published:** 2026-06-06

**Authors:** Yile Huang, Manuel Poretti, Terezie Mandáková, Milan Pouch, Xinyi Guo, Hussein Anani, Estela Perez-Roman, Manuel B. Crespo, Stefan Grob, Alexandros Bousios, Christian Parisod, Martin A. Lysak

**Affiliations:** 1 Central European Institute of Technology (CEITEC), Masaryk University, Brno, Czechia; 2 National Centre for Biomolecular Research (NCBR), Faculty of Science, Masaryk University, Brno, Czechia; 3 Department of Biology, University of Fribourg, Fribourg, Switzerland; 4 School of BioSciences, University of Melbourne, Melbourne, Australia; 5 Department of Experimental Biology, Faculty of Science, Masaryk University, Brno, Czechia; 6 School of Life Sciences, University of Sussex, Brighton, UK; 7 Department of Environmental Sciences and Natural Resources (dCARN), University of Alicante, Alicante, Spain; 8 IBMP, University of Strasbourg, Strasbourg, France

**Keywords:** Plant evolution, Speciation, Evolutionary genetics

## Abstract

Genomic redundancy following whole-genome duplication creates opportunities for double-strand misrepair that can lead to chromosomal rearrangements and reductions in chromosome number, known as descending dysploidy. Although flowering plants often undergo post-polyploid rediploidization, the pathways and consequences of descending dysploidy remain poorly understood. Here, we sequence and assemble the genomes of eight *Biscutella* species ranging from 0.6 to 1.1 Gb and exhibiting chromosome numbers of *n* = 6, 8 and 9. Our analysis reveals ~11 million years of diploidization from an allotetraploid ancestor with *n* = 14, characterized by independent descending dysploidy. We identify early-diverging (*n* = 8/6) and late-diverging (*n* = 9) genomes that share convergent and divergent features. While both clades show similar levels of gene fractionation and preferential retention of polyploidy-derived genes, the early-diverging genomes exhibit a higher solo-to-intact ratio of Ty3/Gypsy long terminal repeat retrotransposons and fewer conserved topologically associated domains (TADs). We also identify 14 chromosome breakpoints frequently located at TAD boundaries and often associated with transitions between A and B compartments. These results suggest that, although post-polyploid descending dysploidy appears to be independent and superficially stochastic, shared genomic features may predispose recurrent chromosome breakpoints across species.

## Abstract (web summary)

Flowering plants can undergo whole-genome duplications, but how they return to diploid-like states is poorly understood. Here authors show diploidization in the *Biscutella* genus is shaped by clade divergence and constrained by shared genomic features.

## Introduction

Land plants are known for their remarkable karyological variation, with chromosome numbers ranging from two pairs (*n* = 2; e.g., *Colpodium versicolor* in Poaceae) to several hundred pairs (e.g., *Ophioglossum* species in Ophioglossaceae)^1^. This extraordinary variation is primarily shaped by two antagonistic evolutionary processes: polyploidy (whole-genome duplication, WGD) and diploidization^2–8^. Polyploidy contributes to an increase in the number of chromosomes, whereas diploidization is typically associated with the restoration of bivalent pairing and a decrease in the number of chromosomes, a process known as descending dysploidy. The cyclic nature of the independent emergence of genomic redundancy followed by its gradual reduction is widespread in most clades of land plants^9,10^. Although significant progress has been made in understanding WGDs, our knowledge of the triggers, mechanisms, and implications of post-polyploid diploidization remains relatively sparse^11^.

In plants, a common way of descending dysploidy is non-allelic homologous recombination (NAHR) between two or more different chromosomes, reducing the number of chromosomes typically by one^2,12,13^ (Supplementary Fig. 1). Descending dysploidy is particularly common after WGD events, as polyploidization multiplies homologous sequences as substrates for illegitimate recombination, leading first to simple fusion chromosomes and later, as diploidization progresses, to complex fusion chromosomes (CFCs), in which segments of multiple non-homologous chromosomes are combined. CFCs frequently occur in diploidized tetraploid or hexaploid plant genomes, such as cotton (*Gossypium*)^14^, soybean^15^, *Brassica*^16^ and camelina (*Camelina*)^17^. The frequency and stability of CFCs depends, among other factors, on the degree of DNA homology and repeat content, the frequency of double-strand breaks (DSBs) and NAHR as well as structural (e.g., the size of CFCs) and functional (e.g., altered gene expression) constraints.

WGD events often occur before the diversification of a clade^18,19^, leading to the assumption that all clade members are descended from a single polyploid genome or metapopulation^20^. The polyploid clade diversifies as a result of adaptive radiation and is typically associated with some level of genetic, epigenetic, or cytological diploidization. Assuming that the polyploid populations are genetically identical or nearly identical, the results of independent diploidization may be convergent, or alternatively, variable abiotic and biotic conditions may determine or modulate the outcomes of the diploidization process. Nevertheless, the question of the likelihood of convergent vs. divergent post-polyploid diploidization is still largely unresolved. For example, if the genetic makeup of spatially separated polyploid populations or descendant species is the same, are there chromosomal regions or sequences that are more prone to DSBs and chromosomal rearrangements (CRs)? Furthermore, what is the likelihood that DSB misrepair at recombination hotspots in different diploidizing populations or species will lead to the same CRs and eventually to the same or very similar fusion chromosomes?

The cruciferous genus *Biscutella* comprises about 60 species distributed throughout the Mediterranean basin, and as far as Iran and the Arabian Peninsula^21^. *Biscutella* is a polybasic genus with three different base chromosome numbers: *x* = 6 (*B. lyrata* L.), 8 (a dozen) and 9 (most species)^22^. Intrageneric karyological variation attracted scientific interest and was first recognized as an interesting evolutionary problem by Manton^23^, who then introduced the *B. laevigata* L. species complex as one of the first models for studying the role of autopolyploidy in the evolution of plant genomes^24^. The identification of *x* = 9 in most *Biscutella* species led to the conclusion that nine chromosomes represent the ancestral condition, with lower chromosome numbers (*n* = 8 and 6) interpreted as resulting from chromosome loss (aneuploidy)^22^. Much later, however, transcriptomic and cytogenomic studies of the diploid species *B. laevigata* subsp. *varia* (Dumort.) Rouy and Foucaud (2*n* = 18)^25^, *B. baetica* Boiss and Reuter (2*n* = 16)^26,27^ and *B. lyrata* (2*n* = 12)^26,27^ indicated that these species underwent a common WGD event. Consequently, the current chromosome numbers are thought to have evolved from a mesotetraploid progenitor genome through a process of descending dysploidy that accompanied post-polyploid diploidization^25–28^.

To investigate the pathways and constraints of cytological diploidization, we generate chromosome-level genome assemblies for eight *Biscutella* species from morphologically and phylogenetically distinct infrageneric clades (Supplementary Fig. 2)^29,30^, with three different base numbers (*n* = 6, 8 and 9). We show (1) post-polyploid cytological diploidization mediated by independent descending dysploidy in early- and late-diverging species, (2) convergence in the extent of subgenome fractionation and retention of polyploidy-derived genes, (3) divergence in the activities of LTR retrotransposons (LTR-RTs) and the distinct conservation patterns of the active A and repressive B compartments, along with topologically associated domains (TADs) between early- and late-diverging species, and (4) the role of 3D genome architecture in establishing chromosome breakpoints. The current study offers a thorough genomic analysis of post-polyploid diploidization within a single genus of the Brassicaceae family.

## Results

### Chromosome-scale genome assembly and annotation of eight *Biscutella* species with varying chromosome numbers

**Fig. 1:**
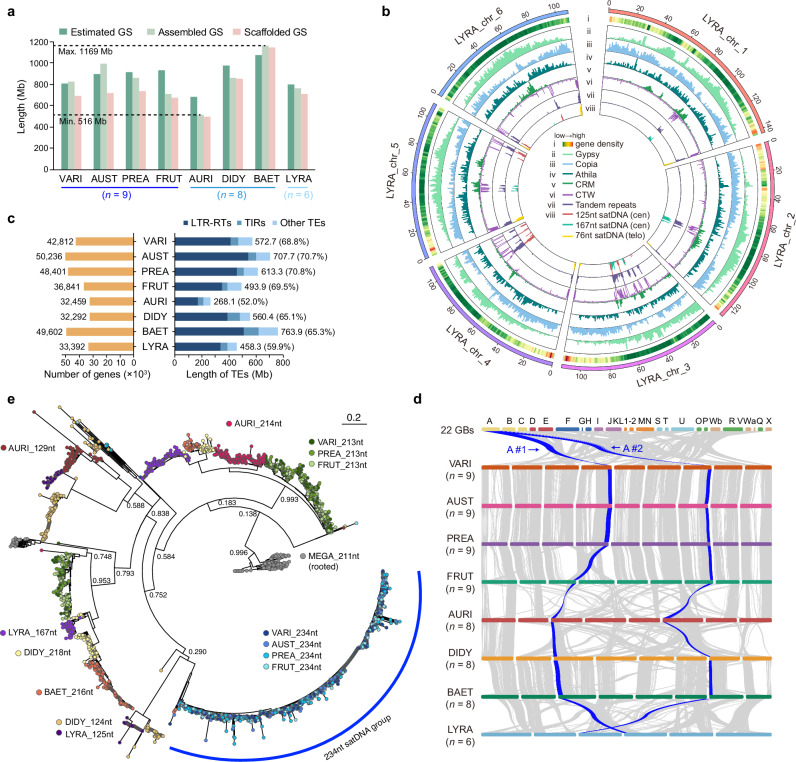
Genome size variation, repeatome, and inter-species chromosome collinearity of eight *Biscutella* species. **a** Genome size (GS) variation. The estimated (flow cytometry-based; green), assembled (light green), and scaffolded (pink) genome size is shown for the eight *Biscutella* species. The assembled genome size ranged from c. 516 to 1169 Mb. **b** Chromosomal organization in *B. lyrata*. The six chromosomes of *B. lyrata* are depicted as circular tracks (from outer to inner) representing: (i) gene density, (ii) Gypsy density, (iii) Copia density, (iv) Athila density, (v) CRM density, (vi) DNA compression (CTW), (vii) tandem repeat density, and (viii) densities of 125-bp (pink) and 167-bp (green) centromeric (cen) and 76-bp (yellow) subtelomeric (telo) satellite DNAs. Densities were calculated using the sliding-window method, counting each element within a non-overlapping 1-Mb window across the genome. Chromosomal organizations for the other seven *Biscutella* species are shown in Supplementary Figs. 5, 6. **c** Comparison of gene number and TE (transposable element) length. The number of annotated protein-coding genes (left), total TE length (right, Mb), and proportion of TEs relative to the entire genome (%) are shown. **d** Syntenic relationships between the eight *Biscutella* genomes. A riparian plot illustrates macrosynteny across 22 ancestral genomic blocks (GBs A – X) and the eight assemblies, with the dark blue lines highlighting the two genomic copies of block A. Species acronyms are as follows: *B. laevigata* subsp*. varia* (VARI), *B. laevigata* subsp*. austriaca* (AUST)*, B. prealpina* (PREA), *B. frutescens* (FRUT), *B. auriculata* (AURI), *B. didyma* (DIDY), *B. baetica* (BAET), and *B. lyrata* (LYRA). **e** Phylogenetic analysis of centromeric satellite DNAs. A maximum-likelihood tree was constructed using 2358 centromeric satDNA monomers from eight *Biscutella* species and 168 monomers of 211-bp satDNA from *M. pygmaea* (MEGA) using FastTree^127^. Local support values are labeled on nodes of the main branches. Each dot represents an individual monomer. Different satDNA types are color-coded at the tips of the tree to indicate their classification.

We generated chromosome-scale genome assemblies using Illumina short-read (c. 83× genome coverage), Oxford Nanopore long-read (ONT; c. 62×), and high-throughput chromosome conformation capture (Hi-C) sequencing for eight *Biscutella* species, including *B. laevigata* subsp*. varia* (*n* = 9; haploid nuclear genome size [1 C] = 814 Mb; referred to as *B. varia*)*, B. laevigata* subsp*. austriaca* (*n* = 9; 904 Mb; referred to as *B. austriaca*)*, B. prealpina* (*n* = 9; 916 Mb), *B. frutescens* (*n* = 9; 936 Mb), *B. auriculata* (*n* = 8; 686 Mb), *B. didyma* (*n* = 8; 980 Mb), *B. baetica* (*n* = 8; 1.1 Gb), and *B. lyrata* (*n* = 6; 806 Mb). The PacBio high-fidelity (HiFi; c. 28×) sequencing was additionally performed for *B. lyrata* (Supplementary Data 1). Eight genome assemblies with total lengths of c. 516 to 1169 Mb were obtained following either ONT- or PacBio-based assembly strategy (Fig. 1a; Methods). Compared to *Biscutella* genomes assembled via the ONT-based strategy, which yielded an average of 1258 contigs with an average contig N50 length of ~2.9 Mb, the *B. lyrata* genome, assembled using the PacBio-based strategy, consists of 119 contigs and a contig N50 length of 22.89 Mb (Supplementary Data 2), representing the most continuous *Biscutella* genome assembly (Fig. 1b). The assembled contigs were subsequently organized into 6, 8, or 9 pseudochromosomes ranging in size from 56 to 159 Mb based on Hi-C contact maps (Supplementary Fig. 3). Benchmarking Universal Single-Copy Ortholog (BUSCO) analysis of the assembled sequences showed high completeness of all eight assemblies (92.0 – 99.1%; Supplementary Data 3).

Using a combination of ab initio, homology-based and evidence-based approaches (Methods), we annotated 32,292 to 50,236 high-confidence protein-coding genes (Fig. 1c), with comparable average gene lengths (~2160 bp) and exon numbers (average ~5 per gene; Supplementary Data 4). Synteny analysis further identified 22,492 to 31,397 gene pairs across the eight *Biscutella* genomes, corresponding to 22 ancestral genomic blocks (GBs A—X; defined as conserved chromosomal segments in the Brassicaceae genomes)^31,32^. Syntenic genes are predominantly distributed along chromosome arms, whereas gene-poor regions are typically associated with (peri)centromeres. The position of syntenic genes delimiting the GBs flanking the gene-poor regions was used to define the boundaries of (peri)centromeric regions (Supplementary Data 5). Utilizing these syntenic gene pairs as pillars, we identified two genomic copies for nearly all 22 GBs (Supplementary Fig. 4). An exception was block G in *B. prealpina* and *B. varia*, where only one copy was detected, likely due to extensive gene loss or misassembly in these regions. The overall 2:1 syntenic relationship between the *Biscutella* genomes and the 22 GBs confirms a mesotetraploid WGD predating diversification of the genus^25,27,28^ and further supports the reliability of the gene annotations. However, only the closely-related *B. austriaca*, *B. varia* and *B. prealpina*^33^ retained relatively stable syntenic relationships, while the remaining genomes contain extensive CRs (Fig. 1d).

A total of 268 to 764 Mb of transposable elements (TEs) were identified, constituting 52 to 71% of the assembled genomes (Fig. 1c). The most abundant TEs were long terminal repeat retrotransposons (LTR-RTs; 34.3–54.3%), followed by helitrons (8.8–14.1%) and terminal inverted repeats (TIRs; 5.9–10.4%; Supplementary Data 6). Ty3/Gypsy was the predominant superfamily of LTR-RTs (53.3–62.2% of total LTR-RTs), followed by unclassified LTR-RTs (19.2–27.6%) and the Ty1/Copia superfamily (13.1–25.7%; Supplementary Data 6). Tandem repeat sequences ranged from 19.5 to 88.7 Mb (Supplementary Data 7), with centromeric satellite DNA (satDNA) identified in all eight species and (sub)telomeric satDNA detected in four species (Methods). In *n* = 9 species, two types of centromeric satDNAs were identified (monomer length of 213 bp and 234 bp), while in the *n* = 8/6 species, the monomer lengths of centromeric satDNAs ranged from 124 to 218 bp, comprising seven types (Supplementary Figs. 5–7 and Supplementary Data 8). Phylogenetic analysis and pairwise comparisons further revealed that the 234-bp satDNAs form a distinct conserved clade with high sequence similarities (Fig. 1e and Supplementary Fig. 8), suggesting a conserved centromere structure in recently diverging *n* = 9 species. In contrast, other centromeric monomers displayed lower sequence similarity, reflecting their higher variability.

### Subgenome divergence revealed the allotetraploid origin of *Biscutella*

**Fig. 2:**
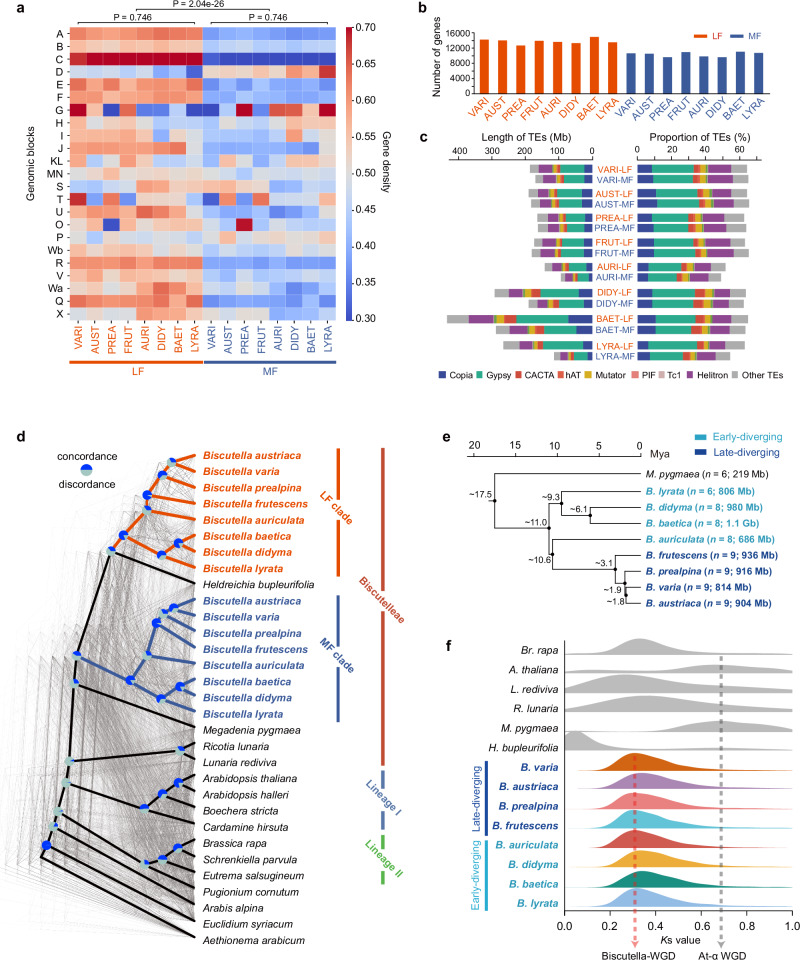
Structural and phylogenomic characteristics of the two *Biscutella* subgenomes. **a** Syntenic gene density in the LF and MF subgenomes across eight *Biscutella* species compared to the 22 ancestral genomic blocks. Colors represent the average percentage of retained homeologous genes in the *Biscutella* subgenomes surrounding each ancestral gene. Here, 300 genes flanking each side of a given gene locus were analyzed, giving a total window size of 601 genes. A significant difference in gene density was found between the LF and MF subgenomes (two-sided Wilcoxon rank-sum test), whereas interspecific differences were not significant (two-sided Kruskal-Wallis test). See Source Data for detailed statistical results. **b** Total number of genes assigned to the LF and MF subgenomes. **c** TE content in two subgenomes, showing the length (left) and proportion (right) of eight major TE types. The proportion of TEs (%) was calculated as the ratio of the total TE length in a subgenome to its size. **d** Subgenome-aware phylogenetic analysis. A cloud tree shows concordance and discordance among 1234 nuclear gene trees. The coalescent-based tree including the two *Biscutella* subgenomes and 15 other crucifer genomes representing the major Brassicaceae lineages is shown with thick black lines. Pie charts indicate the proportions of gene trees concordant or discordant with the species tree topology. *Aethionema arabicum* was used as an outgroup. **e** Simplified time-calibrated phylogeny with inferred divergence times. The *Biscutella* species are grouped into late-diverging species (*B. varia*, *B. austriaca*, *B. prealpina*, and *B. frutescens*) and early-diverging species (*B. auriculata*, *B. didyma*, *B. baetica*, and *B. lyrata*). Four raw time-calibrated trees that include more species are shown in Supplementary Fig. 12. **f** *K*s-value distribution for homeologous gene pairs in eight *Biscutella* genomes and six other species, including *Br. rapa*, *A. thaliana*, *L. rediviva*, *R. lunaria*, *M. pygmaea*, and *H. bupleurifolia*. The *K*s-value peak for *Biscutella* is ~0.33 (red dashed line), corresponding to a WGD event ~11 Mya. Source data are provided as a Source Data file.

Subgenomes of *Biscutella* were identified based on unequal gene density of two genomic copies and verified by phylogeny and *K*-mer sequence similarity. Biased gene fractionation (gene loss following WGD) was observed between duplicated GBs, with one copy generally exhibiting higher gene density than the other (Fig. 2a), as confirmed by comparative chromosome painting experiments (Supplementary Fig. 9). This discrepancy in gene density permitted the classification of the genomic regions into two distinct groups corresponding to the two subgenomes: the less fractionated subgenome (LF) and the more fractionated subgenome (MF). The LF subgenome harbored more protein-coding genes and generally a larger total length of TEs (12,700–14,899 genes; 142.5–433.2 Mb TEs) compared to the MF subgenome (9656–11,025 genes; 95.0–288.2 Mb TEs; Fig. 2b,c), while the proportion of different TE categories was comparable between subgenomes in all species (Fig. 2c), suggesting similarity of the two progenitor genomes in TE content prior to the hybridization.

Subgenome-aware maximum likelihood (ML) and coalescent analyses were performed on orthologous gene groups from eight *Biscutella* species and 15 other Brassicaceae genomes representing major crucifer lineages to infer the phylogenetic placement of the putative progenitor species. Considerable topological discordance was observed among 1234 syntenic gene trees and the coalescent-based tree (Fig. 2d). The internodes with conflicting topologies were particularly evident in the placements of *B. auriculata*, the neotetraploid *Heldreichia bupleurifolia* (Biscutelleae), and the basal node of the Biscutelleae clade (the diploid *Megadenia pygmaea*, and the mesotetraploid *Lunaria* and *Ricotia* species), suggesting a complex, non-bifurcating phylogeny. The ten distinct topologies observed among the phylogenetic trees constructed separately for the 22 GBs (Supplementary Fig. 10) consistently identified two coherent clades: one comprising all *n* = 9 species (*B. varia*, *B. austriaca*, *B. prealpina*, and *B. frutescens*) and another comprising *B. baetica*, *B. didyma* and *B. lyrata*. Despite different placement of *B. auriculata* in the nuclear gene dataset, the coalescent-based tree and the chloroplast tree (Supplementary Fig. 2) positioned *B. auriculata* as a sister to the *n* = 9 clade, despite its chromosome number being the same as in *B. baetica* and *B. didyma* (*n* = 8). The LF subgenome formed a sister clade with the neotetraploid *H. bupleurifolia*, whereas the MF subgenome formed a monophyletic clade outside this sister group, confirming the hierarchical clustering of *K*-mer abundance and principal component analysis (PCA; Supplementary Fig. 11).

The ancestral *Biscutella* genome originated from hybridization between two progenitor species that had diverged around 9–13 million years ago (Mya), based on the time-calibrated trees (Fig. 2e and Supplementary Fig. 12) and the distribution of synonymous substitution rate for homeologue pairs (*K*s-value peak = ~0.33; Fig. 2f). Time-calibrated phylogenies revealed that the divergence of *B. lyrata*, *B. didyma*, and *B. baetica* (~9.3 Mya; hereafter referred to as early-diverging species) significantly predates that of the *n* = 9 species (~3.1 Mya; hereafter referred to as late-diverging species; Fig. 2e). Although the coalescent-based tree retrieved *B. auriculata* as sister to the *n* = 9 clade (Fig. 2d), it diverged from the *n* = 9 species as early as ~10.6 Mya (and is therefore also categorized as an early-diverging species).

### Reconstruction of the ancestral mesotetraploid genome of *Biscutella*

**Fig. 3:**
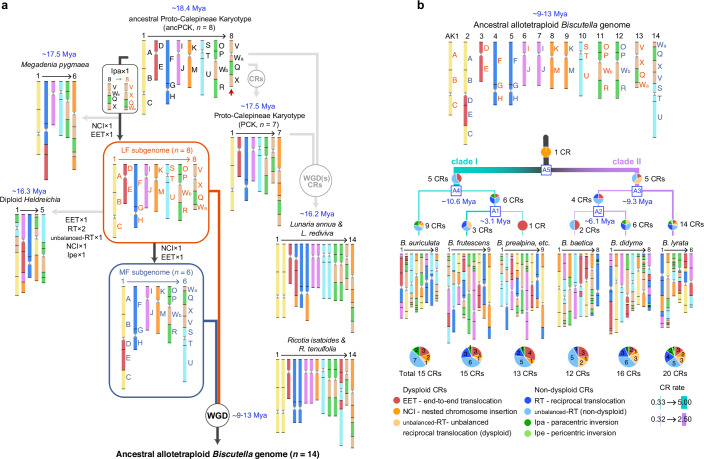
Karyotype evolution of *Bicutella* genomes. **a** Reconstructed genome evolution in the tribe Biscutelleae. The earliest ancestor of Biscutelleae structurally resembled the ancPCK-like genome (*n* = 8), which diversified into an 8-chromosome genome with a paracentric inversion on chromosome AK8 and into a PCK genome by descending dysploidy (*n* = 8 → *n* = 7). Hybridization(s) between ancPCK-like (*n* = 8) and PCK-like (*n* = 7) genomes gave rise to the ancestral tetraploid genome(s) of *Lunaria* and *Ricotia*, subsequently diploidized to *n* = 14^27^. The Ipa-bearing ancPCK-like genome, structurally resembling the LF subgenome of *Biscutella* (*n* = 8), underwent three independent descending dysploidies forming (i) the *M. pygmaea* genome (*n* = 8 → *n* = 6), (ii) the *H. bupleurifolia* genome (*n* = 8 → *n* = 5), and (iii) the MF subgenome of the tetraploid *Biscutella* ancestor (*n* = 8 → *n* = 6). KL and MN blocks are abbreviated to K and M. EET end-to-end translocation, RT reciprocal translocation, unbalanced-RT unbalanced reciprocal translocation, NCI nested chromosome insertion, Ipa paracentric inversion, Ipe pericentric inversion, CR chromosomal rearrangement. **b** Post-polyploid genome evolution in *Biscutella*. The ancestral allotetraploid genome (*n* = 14) underwent a shared NCI event and diverged into two clades: clade I (cyan lines) includes *B. auriculata* (*n* = 8) and the *n* = 9 species, clade II (purple lines) contains *B. baetica*, *B. didyma* (both *n* = 8) and *B. lyrata* (*n* = 6). The conserved *n* = 9 genomes are represented by *B. prealpina*. The ancestral karyotype at each evolutionary node (A1 to A5) was inferred using WGDI based on a bottom-up strategy (the detailed evolutionary pathway is shown in Supplementary Figs. 15–17). The different types of CRs (see Supplementary Fig. 1 for simplified CR models) are shown as pie charts, with the total number of CRs per species indicated below. CR rate was calculated as the total number of CRs divided by the elapsed time and indicated by line thickness (thin: low rate, thick: high rate). The detailed CR rates are listed in Supplementary Data 9.

The ancestral karyotype of *Biscutella* genomes was reconstructed using both top-down and bottom-up strategies. The top-down approach included comparing frequent GB associations in *Biscutella* species with those in the known ancestral karyotypes of Brassicaceae, including Ancestral Crucifer Karyotype (ACK)^31,32^, ancestral Proto-Calepineae Karyotype (ancPCK)^25^ and Proto-Calepineae Karyotype (PCK)^34^. The ancPCK karyotype (*n* = 8) best explained the observed GB associations in *Biscutella*, with 73.3% and 66.7% of GB associations in ancPCK also found in the LF and MF subgenomes, respectively (Supplementary Fig. 13). A shared paracentric inversion (Ipa) predating the speciation of *Biscutella*, *M. pygmaea* and *H. bupleurifolia* restructured the ancestral chromosome AK8 by rearranging the V + Wa + Q + X association into V + X + Q + Wa. This event is supported by the presence of the V + X + Q + Wa association in the MF subgenome of all *Biscutella* species and in the LF subgenome of three species (*B. auriculata*, *B. baetica*, *B. didyma*). The four *n* = 9 species and *B. lyrata* retained a truncated X + Q + Wa association in the LF subgenome, resulting from subsequent rearrangements that disrupted the V + X association (Fig. 3a and Supplementary Fig. 13). Furthermore, additional conserved GB associations identified in the MF subgenome, e.g., D + E + C and S + V + X + Q + Wa, but absent in the LF subgenome (Supplementary Fig. 13), indicate that the two diploid *Biscutella* ancestors had distinct karyotypes. Based on these observations, we infer that the LF subgenome originated from an ancPCK-like genome (*n* = 8) with the Ipa on chromosome AK8, and that the MF subgenome underwent additional nested chromosome insertion (NCI) and end-to-end translocation (EET) events, which reduced its chromosome number to six (*n* = 6), while preserving more ancestral GB associations during subsequent speciation events (Supplementary Fig. 13). To further validate the accuracy of the top-down inference, we used a bottom-up strategy with the WGDI tool^35^ to hierarchically reconstruct ancestral karyotypes at each phylogenetic node, from youngest to oldest (Supplementary Fig. 14). The most recent common allotetraploid ancestral karyotype of *Biscutella* was eventually reconstructed and matched the results of the top-down strategy (Fig. 3a,b). Our integrative analysis revealed that the ancestral mesotetraploid *Biscutella* genome comprised 14 chromosome pairs (*n*  =  6  +  8; 2*n*  =  4*x*  =  28) formed through hybridization between two distinct parental genomes: LF (*n* = 8) and MF (*n* = 6) (Fig. 3a).

### Independent descending dysploidy

The post-polyploid evolution of the allotetraploid ancestor (*n* = 14) to the modern species (*n* = 9, 8 and 6) was reconstructed by integrating multiple CR events, including EET, reciprocal translocation (RT), unbalanced reciprocal translocation (unbalanced-RT), NCI, Ipa and pericentric inversion (Ipe) (Fig. 3b, see Supplementary Figs. 15–17 for detailed evolutionary pathways). Only one NCI event is shared by all eight species (Fig. 3b), whereas the remaining CRs are clade- or species-specific. The early-diverging species experienced 2–14 independent, private CR events, while the late-diverging species had only one to three. Unbalanced-RTs were the predominant CR type across all *Biscutella* genomes (5–9 events per species) and, when accompanied by stable centromere inactivation in dicentric chromosomes (Supplementary Fig. 1), contributed to the reduction of the ancestral chromosome number (*n* = 14) to present-day chromosome numbers (dysploid unbalanced-RTs). Despite the identical chromosome number (*n* = 8), *B. auriculata* and the *B. baetica*–*B. didyma* clade followed distinct evolutionary trajectories. The chromosome restructuring in *B. auriculata* primarily involved 15 CRs, with descending dysploidy being predominantly driven by EETs (3) and NCIs (2). However, *B. baetica* and *B. didyma* underwent 12 and 16 CRs, respectively, with dysploid CRs mainly mediated by EETs (3 and 2) and unbalanced-RTs (2 and 3) (Fig. 3b). A comparable number of CRs, 15 and 13, was observed in the *n* = 9 genomes, also dominated by unbalanced-RTs, EETs, and RTs. The highest number of CRs (20) was identified in the highly diploidized *B. lyrata* (*n* = 6). Furthermore, the CR rate varied along the phylogeny, peaking during early divergence (between A5 and A4/A3) at 2.5–5.0 chromosomal rearrangements per million years (CRs/My) and decreasing to 0.3–1.5 CRs/My towards the tips (between A4/A3 and extant species; Fig. 3b and Supplementary Data 9).

### Ty3/Gypsy retrotransposons show a higher solo-to-intact LTR ratio in early-diverging *Biscutella* species

**Fig. 4:**
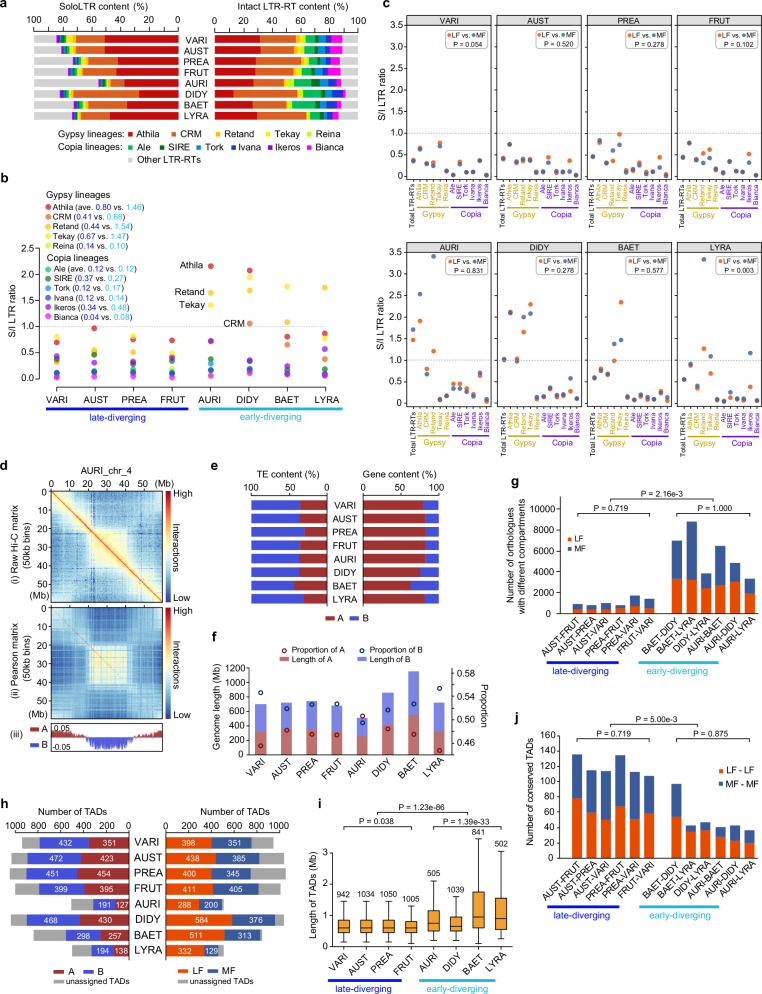
The evolution of LTR retrotransposons and 3D chromatin organization. **a** Content of soloLTRs and intact LTR-RTs, showing the relative proportions of soloLTRs (left) and intact copies (right) in five Gypsy and six Copia lineages. **b** Solo:intact (S/I) LTR ratios across 11 Gypsy or Copia lineages in eight *Biscutella* species. Average S/I LTR ratios for late-diverging (dark blue) and early-diverging (light blue) species are labeled. **c** S/I LTR ratios in subgenomes. Ratios for total LTR-RTs and individual lineages are shown for the LF and MF subgenomes. No significant differences were observed between the two subgenomes of the eight species (two-sided Wilcoxon signed-rank test). See Supplementary Data 10 for detailed statistical results. **d** Hi-C map of chromosome 4 in *B. auriculata*. The tracks from top to bottom represent: (i) the raw Hi-C interaction matrix at 50-kb resolution, (ii) the corresponding Pearson matrix, and (iii) the first principal component (PC1) derived from the PCA analysis of this matrix, which was used to define the A (red) and B (blue) compartments. The Hi-C maps of other chromosomes of the analyzed *Biscutella* species are shown in Supplementary Fig. 19. **e** TE and gene content in A/B compartments, shown as proportions of total TEs (left) and genes (right). **f** Length and proportion of A/B compartments. The total length (bars; Y-axis left) and proportion (dots; Y-axis right) of A/B compartments were calculated for each genome. **g** Orthologous gene pairs assigned to different compartments between species, with statistical comparisons among species and between late- and early-diverging groups (two-sided Wilcoxon rank-sum test). See Source Data for detailed statistical results. **h** Total number of TADs in A/B compartments (left) and LF/MF subgenomes (right). Unassigned TADs include those spanning mixed compartments or subgenome regions. **i** Distribution of TAD lengths in the eight *Biscutella* genomes. Box plot shows the median (center black line), interquartile range (box limits), and whiskers extending to 1.5× the interquartile range. The sample size (n) is indicated above each box. Statistical tests were performed as in (g). See Source Data for detailed statistical results. **j** Conserved TADs between species (two-sided Wilcoxon rank-sum test), defined by proximity of orthologous genes to TAD boundaries. See Source Data for detailed statistical results. Source data are provided as a Source Data file.

Although LTR-RT sequences represent the predominant component of repeats in the *Biscutella* genomes, intact LTR-RT elements accounted for only about 18.7% of the total LTR-RTs (Supplementary Data 6). Most of these intact LTR-RTs are relatively young, with the average insertion times of the two major superfamilies—Ty3/Gypsy and Ty1/Copia—both within 1 million years (Supplementary Fig. 18). Alongside their recent amplification, most LTR-RTs are also subject to rapid deletion, as we identified a substantial number (4869–8401; Supplementary Data 10) of recombination remnants defined as soloLTRs, that is, LTR-RT sequences where the internal domain and one of the two LTRs have been deleted (Methods). Analysis of different LTR-RT lineages revealed that the Athila and CRM dominated the landscape and collectively accounted for 65% of intact LTR-RTs and 55.9% of soloLTRs (Fig. 4a). CRM elements were primarily concentrated around (peri)centromeric regions, while Athila elements were more randomly distributed throughout the chromosomes (Fig. 1b and Supplementary Figs. 5, 6). Comparison of solo-to-intact LTR ratios (S/I LTR ratios) revealed significantly higher values for several Ty3/Gypsy lineages, including Athila, CRM, Retand, and Tekay, in early-diverging species compared to late-diverging species (average S/I LTR ratio of ~1.290 vs. ~0.577; Wilcoxon rank-sum *P*-value = 4.25 × 10^−4^; Fig. 4b). Nevertheless, no significant differences in the S/I LTR ratios were observed for the Ty1/Copia lineages (Fig. 4b) and between the subgenomes (Fig. 4c).

### Two major *Biscutella* species clades show contrasting chromatin organization

Hi-C data provided a higher-order perspective of chromatin organization in the *Biscutella* genomes. The Hi-C maps revealed that contact densities were predominantly concentrated along the main diagonals, with weak *trans*-interactions (Fig. 4d and Supplementary Fig. 19). PCA-based analysis of Hi-C contact data at 50-kb resolution partitioned the *Biscutella* chromosomes into distinct A and B compartments (Fig. 4d and Supplementary Fig. 19). Consistent with observations in other plant genomes (e.g., pepper and soybean)^36,37^, the A compartments were located near the telomeres whereas B compartments occupied the central, repeat-rich regions of the chromosomes, consistent with the global distribution of gene and TE sequences (Fig. 4e and Supplementary Fig. 19). With the exception of the smallest *B. auriculata* genome, which contains more A compartments (50.6% of the genome), the remaining genomes had a slightly higher proportion of B compartments (~53%; Fig. 4f). The examination of orthologous gene pair composition between any two species demonstrated the variability in the A/B compartment assignments. The proportion of orthologous gene pairs assigned to different chromatin compartments was significantly higher in the early-diverging species (14.63–30.42%) than in the late-diverging species (3.51–7.26%; Fig. 4g). Furthermore, the organization of topologically associated domains (TADs) also underwent significant restructuring during post-polyploid diploidization. The late-diverging species had more and relatively shorter TADs (942–1050 TADs, 0.65–0.72 Mb), whereas early-diverging species showed considerable variability in TAD number and length (502–1039 TADs; 0.81–1.35 Mb), with the fewest but longest TADs in *B. lyrata* (502 TADs; 1.35 Mb average length; Fig. 4h,i). TAD conservation, assessed by comparing orthologous gene pairs at TAD boundaries (Methods), indicated that only ~9.49% of TADs were conserved between any two species, with a higher proportion of conserved TADs retained between late-diverging species (11.09–13.44%) than between early-diverging species (5.57–10.43%, Fig. 4j).

### Chromosome breakpoints are associated with changes in 3D genome organization

**Fig. 5:**
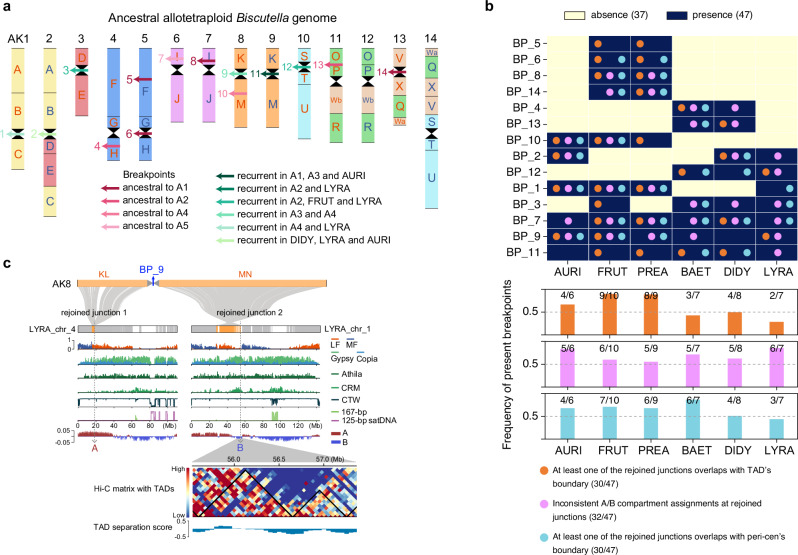
Positions and characteristics of shared chromosome breakpoints. **a** Fourteen chromosome breakpoints (BP_1 to BP_14) were identified in the ancestral allotetraploid *Biscutella* genome. Eight breakpoints are inherited from ancestors at different phylogenetic nodes (gradient pink arrows) and six that arose recurrently across clades (gradient green arrows). Based on cytogenomically determined centromere positions in the closely related diploid *M. pygmaea* and autopolyploid *H. bupleurifolia*^27,38^, we inferred the most likely centromere positions in the ancestral allotetraploid *Biscutella* genome and defined them as paleocentromeres (opposite triangles). **b** Presence and absence of the fourteen breakpoints in *Biscutella* genomes. The conserved *n* = 9 genomes are represented by *B. prealpina*. Three breakpoints are shared by all species, and the remaining are shared by at least two species. The absence (37) and presence (47) of breaks are indicated by light yellow and dark blue squares. Of the 47 present rejoined junctions, 30 overlapped with TAD boundaries (orange dots), and 32 were associated with shifts in A/B compartment assignments (magenta dots), and 30 overlapped with (peri)centromeric regions boundaries (blue dots). **c** Origin and evolution of BP_9. BP_9, a breakpoint near the paleocentromere of ancestral chromosome AK8, underwent CRs that relocated blocks KL and MN to chromosomes 1 and 4, respectively, in *B. lyrata*. The tracks from top to bottom show: the ancestral chromosome AK8; syntenic relationship between AK8 and chromosomes 1 and 4 in *B. lyrata*; distributions of genes on chromosomes 1 and 4; gene density in LF and MF subgenomes; density of Ty3/Gypsy (light green) and Ty1/Copia (light blue) retrotransposons; density of Athila retroelements; density of CRM retroelements; CTW values; density of 125-bp (pink) and 167-bp (green) centromeric tandem repeats; A/B compartment assignment; a Hi-C matrix for the 55.0–57.5 Mb region, along with TADs and corresponding TAD separation scores.

Synteny comparisons of eight *Biscutella* genomes with their ancestral (sub)genomes revealed 14 breakpoints shared by multiple species, including eight inherited from ancestors at different phylogenetic nodes and six that arose recurrently across clades (Fig. 5a and Supplementary Fig. 20; Methods). In the ancestral *Biscutella* genome, nine breakpoints belonged to the LF subgenome (*n* = 8) and five to the MF subgenome (*n* = 6) (Fig. 5a). Based on the inferred ancestral centromere positions, which we define as paleocentromeres^27,38^, eight breakpoints were associated with breaks at/near these regions, while six were located within ancestral chromosome arms (Fig. 5a). In the extant *Biscutella* genomes, more than half of the rejoined junctions aligned with (peri)centromeric regions (Fig. 5b), a proportion higher than expected from 200 simulations using randomly sampled genomic regions (Supplementary Fig. 21).

To assess the relationship between 3D genome organization and chromosome breakage, we examined A/B compartment assignments and TAD distribution within the breakpoint junction regions (Methods). Among the 47 shared breakpoints present in the *Biscutella* genomes, 32 showed inconsistent A/B compartment assignments and 30 matched TAD boundaries (Fig. 5b). Although statistical support within individual species is limited, both proportions are consistently higher than expected from 200 random simulations (Supplementary Figs. 22, 23), suggesting a potential association between breakpoints and 3D genome organization. For example, a breakpoint at/near the paleocentromere of ancestral chromosome AK8 (BP_9 in Fig. 5c), resulted in the rearrangement of blocks MN and KL on arms of chromosome 1 and 4 in *B. lyrata*. The distal end of the MN block likely contains paleocentromeric remnants, characterized by gene depletion and CRM accumulation (Fig. 5c). Despite being located within a chromosome arm, the rejoined junction at the MN block remains in the B compartment, whereas the KL junction occupies the A compartment (Fig. 5c), indicating an A/B compartment shift. More interestingly, the rejoined junction at the MN block colocalized with TAD boundaries (Fig. 5c), suggesting a relationship between chromosome break and TAD architecture. This overlap implies that breakpoints of fixed CRs preferentially occurred at conserved TAD boundaries^39,40^, highlighting the presumed role of TADs as functional entities during chromosome evolution.

### Gene duplication and deletion, and gene sub/neofunctionalization contributed to the emergence of unique traits in *Biscutella*

**Fig. 6:**
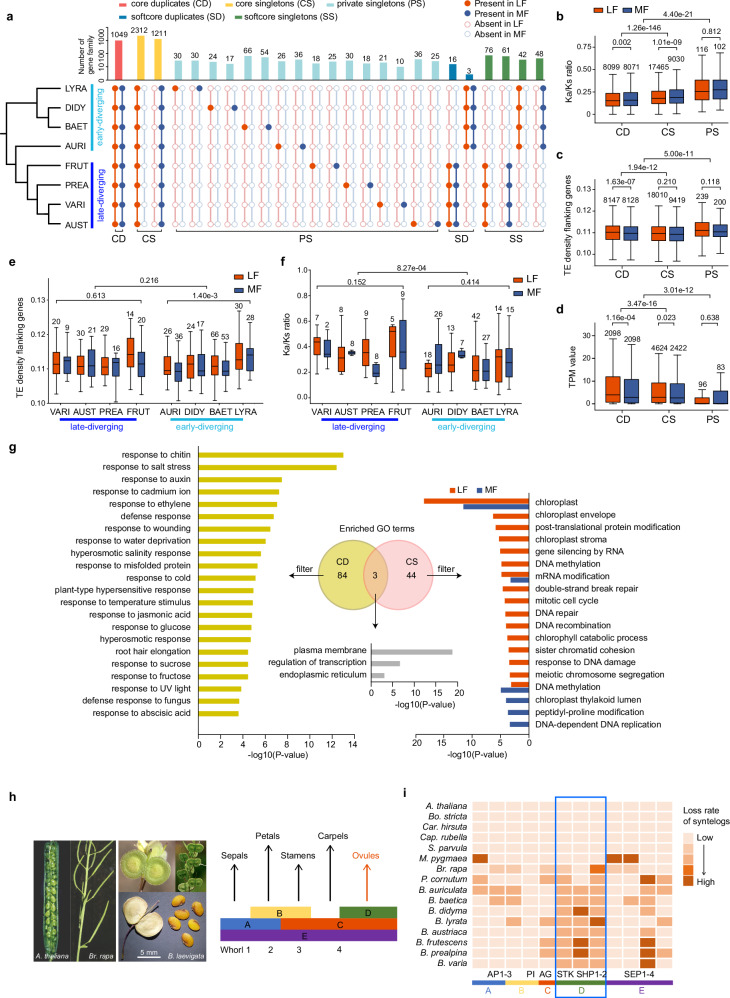
Analysis of WGD-derived gene families in *Biscutella* species. **a** Presence (filled circles) and absence (open circles) of gene families in eight *Biscutella* genomes. The species tree is shown at left, and the upper panel shows the number of gene families in five categories: core duplicates (CD) and singletons (CS) shared by all eight species, softcore duplicates (SD) and singletons (SS) shared by late- or early-diverging species, and private singletons (PS) specific to a particular species. **b** *K*a/*K*s ratios of genes in CD, CS and PS categories, using syntelogs in *M. pygmaea* as reference (two-sided Wilcoxon rank-sum test). Box plot shows the median (center black line), interquartile range (box limits), and whiskers extending to 1.5× the interquartile range. The sample size (*n*) is indicated above each box. Figures (**b**–**f**) follow the same layout; Figures (**b**–**d**) use the same statistical test method. See Source Data for detailed statistical results. **c** TE density at the gene bodies and their 5-kb flanking regions in the CD, CS and PS categories. See Source Data for detailed statistical results. **d** Gene expression levels (TPM values) in the CD, CS and PS categories for *B. baetica* and *B. lyrata*. See Source Data for detailed statistical results. **e** TE density at the gene bodies and their 5-kb flanking regions in the PS category. No significant differences in TE density were found between late- and early-diverging species (two-sided Wilcoxon rank-sum test), while significant differences were found among the four early-diverging species (two-sided Kruskal-Wallis test). Figures (**e**, **f**) use the same statistical test method. See Source Data for detailed statistical results. **f** *K*a/*K*s ratio of genes in the PS category, using syntelogs in *M. pygmaea* as reference. See Source Data for detailed statistical results. **g** GO functional enrichments for CD (left) and CS (right) family categories. A Venn diagram shows the shared (gray bars) and specific GO terms enriched for both CD (yellow bars) and CS (orange and blue bars) categories. **h** Fruit and seed morphology of *A. thaliana*, *Br. rapa*, and *Biscutella laevigata* (left) and the refined ABCDE model^128^ (right). **i** Loss rates of syntelogs for 12 gene families of the ABCDE model (*AP1 – 3*, *PI*, *AG*, *STK*, *SHP1* – *2*, *SEP1* – *4*) are shown for eight *Biscutella* species and seven other Brassicaceae species. Source data are provided as a Source Data file.

To explore the preferential retention of WGD-derived genes following infrageneric cladogenesis and speciation, we identified 4572 syntenic core gene families shared by all eight *Biscutella* genomes. Of these, 1049 families (22.94%) retained duplicates (core duplicates, CD, i.e., paralogues retained in both subgenomes) and 3523 families (77.06%) retained singletons (core singletons, CS, i.e., genes specific to one subgenome, Fig. 6a). In addition, we identified 246 syntenic softcore gene families exclusive to either late- or early-diverging species (softcore duplicates and singletons, SD and SS) and 465 private singletons (PS) specific to individual species (Fig. 6a). No species-specific duplicates were detected. Core gene families, especially CDs, displayed lower *K*a/*K*s ratio (Fig. 6b), were flanked by fewer TEs (Fig. 6c), and showed significantly higher expression levels (Fig. 6d), suggesting they have undergone more stringent selective pressures. Moreover, TE accumulation flanking retained genes was comparable between late- and early-diverging species (Fig. 6e), yet the late-diverging species had significantly higher *K*a/*K*s ratios (Fig. 6f), indicating more relaxed purifying selection.

Functional differences between the retained duplicates and the singletons were assessed through Gene Ontology (GO) functional enrichment. Only three GO terms—“plasma membrane”, “regulation of transcription” and “endoplasmic reticulum”—were shared between the CD and CS gene families (Fig. 6g), while 84 and 44 unique GO terms were identified for each gene family (Supplementary Data 11, 12), revealing functional biases for gene retention. A substantial number of GO terms involved in environmental adaptation were specific to the CD family, particularly those related to ionic and salinity stress, e.g., “response to cadmium ion”, “response to salt stress”, and “hyperosmotic salinity response” (Fig. 6g). The over-representation of ion/salt response-related functions align with the unique ecological niche of *Biscutella*, i.e., more than 50 species are native to rocky, dry and warm habitats throughout the Mediterranean^41^. Notably, domain examination between paralogues revealed 45.28% of CD duplicates (475/1049 pairs) exhibited divergent protein domain compositions, with 220 pairs showing species-specific alterations (Supplementary Fig. 24). GO terms related to chloroplast function and DNA damage repair were exclusively enriched in the CS family, whereas they were underrepresented in the CD family (Fig. 6g). This is consistent with results in 20 flowering plants^42^, as these functions likely involve dosage-sensitive gene networks that constrain gene loss^43,44^. Although duplicates and singletons showed significant functional divergence, no GO terms were enriched among genes specifically retained in early- and late-diverging species.

In contrast to over-retention of genes related to environmental adaptation, the development of lens-like silicles in *Biscutella* might be associated with excessive gene deletions and relaxed selection. Compared to the slender, cylindrical siliques of *Arabidopsis* or *Brassica*, *Biscutella* species produce characteristic didymous flattened silicles with a prominent and accumbent radicle^45,46^ (Fig. 6h). We examined copy number variation in homeologues involved in the ABCDE flowering model^47,48^ (Fig. 6h) and found that, unlike A-, B-, C-, or E-class genes, which regulate sepal, petal, stamen, and carpel development, respectively, all D-class genes in *Biscutella*, including *SEEDSTICK* (*STK*), *SHATTERPROOF1* (*SHP1*), and *SHP2*, specifically regulate ovule development, transitioned from duplicates to singletons (Fig. 6i). In addition, D-class genes in *Biscutella* species experienced relaxed selection, as indicated by their generally higher *K*a/*K*s ratios compared to other seven Brassicaceae species (Supplementary Fig. 25). Specifically, *SHP1*, a MADS-box gene member specifying ovule integument identity in *Arabidopsis thaliana*^49^, remains intact in the mesohexaploid *Brassica rapa* (three copies) and the mesotetraploid *Pugionium cornutum* (two copies), but has reverted to a singleton or even lack syntenic *SHP1* copies in *Biscutella* species (Fig. 6i and Supplementary Data 13). The retained *SHP1* singletons exhibit lower motif conservation in the NCBI-CDD database compared to other Brassicaceae species (Supplementary Fig. 26). Given the key roles of D-class genes in ovule primordial development, we propose that their fractionation and relaxed selection contributed to the evolution of lens-like silicles in *Biscutella* (Supplementary Fig. 27). This finding aligns with observations in Arabidopsis, where loss-of-function mutants of D-class genes produce shorter siliques with rounder, smaller seeds^50,51^.

## Discussion

An inherent feature of whole-genome duplications is the resulting genome redundancy, which provides raw material for random genomic changes under natural selection. One of the characteristic features of genome rediploidization is gene fractionation and the occurrence of chromosomal rearrangements, including those that reduce the increased chromosome numbers towards a lower, often diploid-like number (descending dysploidy). While the trend of post-polyploid diploidization through descending dysploidy is well documented^2,4,8^, the triggers, mechanisms, and evolutionary consequences of this complex process remain poorly understood. Here, we explored the rediploidization patterns of the ancestral mesoallotetraploid *Biscutella* genome by analyzing genomes of eight species with varying genome sizes (0.6–1.1 Gb), chromosome numbers (*n* = 6, 8, and 9), and divergence times (spanning ~11 to 2 Mya).

The *Biscutella* genus likely originated through hybridization between two parental genomes with comparable size, each exhibiting distinct gene fractionation patterns. While both parental genomes descended from a common eight-chromosome ancestor (*n* = 8, ancPCK), the more fractionated parental genome (MF) has undergone descending dysploidy, which reduced its chromosome number from *n* = 8 to *n* = 6. This genome then hybridized with a more ancestral and presumably less fractionated dominant genome (LF, *n* = 8) (Fig. 3a). Although the LF subgenome contains a higher number of protein-coding genes and a greater total length of TEs compared to the MF subgenome (Fig. 2b,c), both subgenomes show similar proportions of major TE types (Fig. 2c) and comparable ratios of soloLTRs to intact LTR-RTs (Fig. 4c). This suggests that the parental (sub)genomes likely had roughly equivalent TE contents prior to the hybridization, with differential fractionation occurring afterward. The differences in S/I LTR ratios between early- and late-diverging genomes for specific Ty3/Gypsy lineages likely reflect lineage-dependent turnover dynamics. These dynamics may result from variation in recombination-mediated removal and/or recent transposition activities^52,53^. Specifically, differences in recombination frequencies among lineages and across chromosomal regions may lead to varying probabilities of soloLTR formation. In addition, lineage-specific transposition bursts can temporarily increase the number of intact LTR-RTs, thereby reducing S/I LTR ratios independently of removal processes.

In crucifers, multiple base numbers need not be directly associated with mesopolyploidy and the diploidization process, but more commonly post-polyploid diploidization is associated with independent reductions in chromosome number, rendering genera and clades polybasic (i.e., having more than one base number)^26,54^. Modern *Biscutella* species are descendants of an allotetraploid ancestral genome that originated ~11 Mya and whose chromosome number (*n* = 14) was reduced 1.6 to 2.3-fold to *n* = 9, 8, and 6^25,27,28^. We show that although modern *Biscutella* species differ in chromosome number, degree of chromosomal diploidization and divergence times, the independent post-polyploid descending dysploidy in *Biscutella* species with *n* = 8 and 9 was mediated by 12 to 16 CRs, and 20 CRs in *B. lyrata* (*n* = 6), all genomes experienced a comparable number of EETs, NCIs and unbalanced-RTs. This is consistent with inferred CR types mediating descending dysploidy in other eudicots^14,55^ and presumably consistent with genome instability after WGD^56,57^. The early stages of cytological diploidization in *Biscutella* were indeed accompanied by high TE dynamics^28^ and higher CR rates, resulting in extensive chromosomal restructuring, compared to the later stages characterized by lower CR rates. Thus, initial inter-subgenome homogenization through CRs and descending dysploidy associated with gene fractionation may gradually slow down the process of cytological diploidization.

We identified 14 chromosome breakpoints shared by several *Biscutella* species. These breakpoints were frequently found non-randomly distributed in relation to higher-order chromatin (A/B) compartments and local TADs. CR breakpoints in *Biscutella* genomes often colocalize with TAD boundaries rather than within the domains themselves (Supplementary Fig. 23), consistent with patterns observed in *Drosophila* and cotton genomes^40,58,59^. Rearrangements at TAD boundaries are more likely to be tolerated because they are less likely to disrupt the internal regulatory structure of a domain. However, in contrast to the highly conserved TADs found in the diploid subgenomes of more recent polyploids such as cotton (origin ~1–2 Mya)^60^ and horseradish (~5 Mya)^61^, *Biscutella* species retained only a limited number of conserved A/B compartments and TADs between orthologs. This highlights the erosion of chromatin topologies over deep evolutionary time scales. Particularly, the early-diverging genomes showed fewer and more variable sizes of conserved TADs compared to those in the late-diverging genomes (Fig. 4i, j). While the fixation of rearrangements compatible with preserved TAD boundaries is plausible, the polyploidy-driven genomic redundancy may enable some TADs being restructured, disrupted, or newly formed. As a result, WGD and post-polyploid rearrangements may lead to changes in chromatin architecture and the reorganization of TAD structures, which can ultimately alter gene expression^62^.

The Biscutelleae exhibit remarkable variation in fruit morphology. The *Biscutella*, *Heldreichia*, and *Megadenia* species are characterized by angustiseptate siliquae (i.e., flattened perpendicular to the septum; Fig. 6h) with mericarps as diaspores (winged in *Biscutella*, winged or weakly winged in *Heldreichia*, and wingless in *Megadenia*)^63,64^, whereas both *Lunaria* and *Ricotia* produce latiseptate siliquae (i.e., flattened parallel to the septum)^63^. Such morphological diversity in Biscutelleae is likely the consequence of independent hybridization-driven evolutionary trajectories^27^. Consistent with this view, we detected signatures of relaxed selection acting on D-class MADS-box genes in *Biscutella* and *Megadenia*, including extensive D-class gene loss in *Biscutella* (Fig. 6i), elevated *K*a/*K*s ratios of D-class genes in *Biscutella* and *Megadenia* compared with non-Biscutelleae species (Supplementary Fig. 25), and the lower expression of D-class genes in floral tissues of *B. austriaca* (Supplementary Fig. 27). Although further functional validation is needed, the evidence suggests that diploidization–by reshaping regulatory networks and relaxing selective constraints on key developmental genes–may contribute to the emergence or stabilization of fruit morphological diversity within the Biscutelleae.

## Methods

### Plant material, library preparation, and sequencing

#### Plant material

The following *Biscutella* accessions were used: *B. laevigata* subsp*. varia* (V12-4; Germany, Beuron), *B. laevigata* subsp. *austriaca* (Jord.) Mach.-Laur. (A2Schnee 3B; Austria, Schneealpe Altenberg), *B. prealpina* Raffaelli & Baldoin (RCBO_NC17; Italy, Recoaro Terme), *B. frutescens* Coss. (PI 650129, USDA collection; Spain), *B. auriculata* L. (PI 650127, USDA collection; Spain, Ames), *B. didyma* L. (LBN-00579, LBN seed bank; Lebanon), *B. baetica* Boiss. & Reut. (Gaucín; Spain, Gaucín), and *B. lyrata* L. (Cádiz; Spain, Cádiz). *B. auriculata* is the type species of section *Iondraba* (Medik.) DC., whereas the remaining 7 species belong to section *Biscutella* that includes two different lineages: series *Biscutellae* with *B. baetica*, *B. didyma* and *B. lyrata*, and series *Laevigatae* Malin. with *B. frutescens*, *B. laevigata* subsp*. austriaca*, *B. laevigata* subsp*. varia*, and *B. prealpina*^30,65^. The plants were grown from seed and cultivated under standard conditions (21/18 °C, 16/8 h of light/dark cycle) in growth chambers.

#### Illumina

Genomic DNA was isolated from ~50 mg of young, green, and undamaged leaves at the pre-reproductive stage using the NucleoSpin Plant II kit (Macherey-Nagel). Illumina sequencing libraries were prepared using the TruSeq Nano DNA HT Sample preparation kit following the manufacturer’s recommendations. Genome sequencing was performed using the Illumina HiSeq Xten platform (Illumina; San Diego, CA, USA).

#### Nanopore and PacBio HiFi

High-molecular-weight (HMW) DNA for long-read sequencing was extracted from approximately 1 g of young leaves (following a 2–3 day dark treatment) using the “Arabidopsis (*Arabidopsis thaliana* LEr) leaf DNA” protocol (https://nanoporetech.com/document/extraction-method/arabidopsis-leaf-dna) with minor modifications. To remove DNA fragments shorter than 10 kb, the Short Read Eliminator (SRE) XS kit (PacBio) was used. The quality and quantity of the extracted genomic DNA were assessed using NanoDrop 2000c Spectrophotometer (Thermo Scientific), Qubit 4 Fluorometer (Thermo Scientific), and Genomic DNA ScreenTape assay (Agilent).

Long-read sequencing processes followed standard protocols of Oxford Nanopore Technologies (ONT; Oxford, UK) and PacBio HiFi technology (PacBio; San Diego, CA, USA). DNA libraries of *Biscutella* species with an average fragment length of >20 kb were constructed for ONT sequencing and sequenced on the PromethION platform. The PacBio circular consensus sequencing (CCS) library for *B. lyrata* was additionally produced and sequenced on one SMRT cell of the PacBio Sequel II system.

#### Hi-C and Omni-C

Hi-C libraries were prepared from ~1 g of leaf samples using the Proximo Hi-C Kit (Phase Genomics, Seattle, USA) with Dpn II as the restriction enzyme, following the manufacturer’s protocol. The Hi-C libraries were sequenced on Illumina HiSeq X-Ten instruments to generate 150-base paired-end reads. The Hi-C library for *B. lyrata* was generated using the Omni-C Proximity Ligation Assay (Dovetail Genomics, Scotts Valley, CA) following the manufacturer’s “Non-Mammalian Samples Protocol version 1.2B”. The library was then sequenced on the Illumina HiSeq X-Ten platform.

#### IsoSeq and RNAseq

Total RNA was extracted from ~50 mg of leaf, flower, and ~100 mg of root tissues using the Quick-RNA Miniprep Kit (Zymo Research). The quality and quantity of the extracted RNA were assessed using NanoDrop 2000c Spectrophotometer (Thermo Scientific), Qubit 4 Fluorometer (Thermo Scientific), and Fragment Analyzer (Agilent). Total RNAs from the tissues of leaves and flowers were mixed equally, along with individual RNA from root tissues, for long-read (PacBio Iso-Seq) sequencing technology to generate transcriptome data. The Iso-Seq cDNA libraries were constructed according to the PacBio standard protocol and sequenced on the PacBio sequel II platform. The short-reads RNA-seq data for *B. baetica* and *B. lyrata* were downloaded from ref. ^27^. The outputs of all sequencing reads are summarized in Supplementary Data 1.

### Genome size measurement by flow cytometry and *K*-mer frequency

Holoploid genome size was estimated by flow cytometry. The samples (young intact leaves; ~1 cm in length) were stained using a solution containing propidium iodide + RNAase IIA, both at final concentrations of 50 μg/ml, for 5 min at room temperature and analyzed using a CyFlow cytometer Partec equipped with a 532 nm diodepumped solid-state laser Cobolt Samba^66^. A fluorescence intensity of 5000 particles was recorded. *Pisum sativum* ‘Ctirad’ (1C = 4.38 pg)^67^ served as the primary reference standard and *Solanum pseudocapsicum* as the secondary standard (1C = 1.29 pg recalculated against the primary reference). Three different samples for each species measured on three consecutive days was used for genome size estimation. In addition, using ~74.2 Gb of Illumina short-read data, genome sizes of *Biscutella* species were further confirmed by *K*-mer frequency analysis (*K* = 21) with the findGSE (v1.94)^68^. The 21-mers were counted with Jellyfish (v2.2.10)^69^. The estimated genome sizes and heterozygosity are summarized in Supplementary Data 2.

### Genome assembly, scaffolding, and quality assessment

We employed two strategies for de novo genome assembly: an ONT-based strategy for *B. auriculata*, *B. baetica*, *B. didyma*, and all *n* = 9 species^70^, and a PacBio-based strategy for *B. lyrata*. Initial assemblies were generated from Nanopore long reads using NextDenovo (v2.2; https://github.com/Nextomics/NextDenovo) with default parameters. The resulting contigs were further polished in two iterative rounds using both Nanopore long reads and Illumina short reads with NextPolish (v1.1.0)^71^. For highly heterozygous genomes (estimated heterozygosity higher than 1%), the program Khaper^72^ with the parameters “--kmer 17 Compress 0.3” was used to select primary contigs and filter redundant sequences from the initial assemblies. For *B. lyrata*, haplotype-resolved assemblies were generated using hifiasm (v0.19.5)^73^ in hybrid mode by integrating PacBio HiFi reads, ONT reads (>= 25 kb), and Hi-C reads. The assembly graph of primary contigs (p_ctg) was used for scaffolding and downstream analyses. The sizes, contig numbers, and contig N50 values of the draft genome assemblies are summarized in Supplementary Data 2. Completeness of genome assemblies was evaluated using BUSCO (v3.0.1)^74^ with embryophyta_odb10 database (1,614 total BUSCOs). For assemblies generated using the ONT-based strategy, Hi-C reads for each genome were aligned to the corresponding contigs using Juicer (v1.5.7)^75^. The 3D-DNA pipeline (v180922)^76^ was used to correct potential mistakes and to order, orient and scaffold the sequences. For *B. lyrata*, the Omni-C data were used to scaffold the genome assembly by YaHS (v1.1)^77^ with default parameters. The linkage results were manually curated to correct misjoins and misassemblies based on visualization using JuiceBox (v1.1.08)^78^. The chloroplast genome was assembled based on Illumina short reads using the GetOrganelle toolkit (v1.7.7)^79^.

### Gene prediction and functional annotation

Protein-coding genes were predicted for *n* = 8/6 species using an evidence-based annotation workflow that integrated multiple sources of evidence. The gene annotations for *n* = 9 species were sourced from ref. ^70^. For transcriptome-based predictions, RNAseq data from *B. baetica* and *B. lyrata* were mapped using Hisat2 (v2.1.0)^80^ and subsequently assembled into transcripts by StringTie (v2.1.4)^81^. IsoSeq datasets for each species were aligned to the genome assemblies using GMAP (v2018-07-04)^82^. All transcripts from RNAseq and IsoSeq were merged with StringTie into a pool of candidate transcripts. TransDecoder (v5.5.0; http://transdecoder.github.io) identified potential coding regions in the resulting transcripts. Additionally, two rounds of PASA (v.2.3.3)^83^ were conducted to refine gene models by identifying untranslated regions and isoforms, using transcripts generated by genome-guided Trinity (v2.11.0)^84^ assemblies. De novo gene predictions were generated using AUGUSTUS (v3.4.0)^85^, with *Biscutella*-specific AUGUSTUS gene models trained using GeneMark-ET (v4.0)^86^. This model leveraged RNA-seq and IsoSeq evidence in two iterative rounds of predictions to refine parameters. For annotation of homologs, protein sequences from *A. thaliana*, *Cardamine hirsuta*, *Eutrema salsugineum*, *Thlaspi arvense*, and *Br. rapa* were aligned to each *Biscutella* genome to identify the homologous genes using GenomeThreader (v1.7.1)^87^. Finally, all gene predictions were integrated into a final gene model set using EVidenceModeler (v1.1.1)^88^ after removing pseudogenes and non-coding genes. FeatureCounts (v2.0.6)^89^ was used to extract the mapped reads for each gene in *B. baetica* and *B. lyrata*, allowing for the calculation of transcripts per million (TPM) values as the expression level of the genes.

Functional assignments for the predicted protein-coding genes were performed with BLAST (v2.12.0+)^90^ to align coding sequences against public protein databases, including NCBI non-redundant (NR) protein^91^, SwissProt^92^, and InterProScan^93^. Gene Ontology (GO) terms for each gene were provided by InterProScan.

### Identification of syntenic genes and fragments

Syntenic gene pairs between the *Biscutella* genomes and the 22 conserved GBs^31,32^ were identified using SynOrths (v1.5)^94^, which integrates sequence similarity and the syntenic context of flanking genes. Homologous gene pairs were first detected by BLASTP embedded in SynOrths, with only best hits or gene pairs showing an E-value < 1 × 10^−20^ retained. A gene pair was considered as syntenic if at least 20% of the 20 flanking genes on either side of the query gene were homologous to those within a 100-gene window surrounding the subject gene. Syntenic gene pairs that were continuously distributed along the *Biscutella* genomes and 22 GBs were considered as ancestral fragments inherited from the progenitors. Due to local structural variations and potential genome assembly errors, local syntenic gene pairs may not be distributed immediately adjacent to other syntenic genes. Thus, adjacent syntenic gene pairs were merged into a single syntenic fragment if they were separated by fewer than 50 intervening genes or a physical distance of <300 kb. A total of 50–55 syntenic fragments were identified in eight genomes, including 11 GB breaks (Supplementary Data 5). Macrosynteny relationships across multiple species were visualized as a riparian plot (Fig. 1d) using NGenomeSyn (v1.41)^95^.

### Repetitive element annotation

The Extensive de novo TE Annotator (EDTA v1.8.3)^96^ was utilized to annotate TEs in each *Biscutella* species with the following parameters: “--species others --step all --anno 1”. Within EDTA, LTRharvest (v1.6.1)^97^, LTR_FINDER_parallel (v1.2)^98^ and LTR_retriever (v2.9.6)^99^ were used for LTR-RTs identification. Intact LTR-RTs, along with their coordinates and structural annotations, were identified using LTR_retriever, which defines intact elements as those containing: (a) a pair of LTRs flanked by conserved dinucleotide palindromic motifs, (b) a 5-bp target site duplication (TSD) at both ends; and (c) an internal region encoding protein domains such as *gag*, *pol*, and *env*^99^. Tandem repeats were identified using TRASH (v2)^100^. The ribosomal DNA (rDNA) sequences were predicted with Barrnap (v0.9; https://github.com/tseemann/barrnap) using the Eukaryota database. For precise classification of LTR-RTs at the lineage level, TEsorter (v1.4.6)^101^ was employed. SoloLTRs were identified using the new soloLTRseeker pipeline (https://github.com/estpr/soloLTRseeker)^102^. In brief, soloLTRseeker first generates a high-quality non-redundant LTR library using LTRs of intact elements classified in advance to LTR lineages by TEsorter. It then runs BLASTn on the genome with 99% and 80% thresholds for query coverage and sequence identity, respectively. 200-bp sequences flanking each putative soloLTR locus are compared with sequences of the same length from both termini of the internal domain of intact elements of the same lineage to filter out cases of partially deleted TEs (that are not soloLTRs). Finally, for each candidate, upstream and downstream sequences of 6-bp length are locally aligned to annotate target-site duplications.

The coordinates of syntenic genes at the ends of GBs flanking gene-poor regions were used to delineate the boundaries of (peri)centromeric regions (Supplementary Data 5). DNA compression was generated using the context-tree weighting (CTW) function of the BCT package in R to further refine centromere localization. Tandem repeats that were significantly enriched in (peri)centromeric regions and overlapped with the troughs of the CTW values were identified as centromeric satDNA candidates, and those located at the ends of chromosomes were identified as (sub)telomeric satDNA candidates. The screened candidates were further validated through FISH experiments.

Insertion ages of LTR-RTs were estimated using the method proposed by SanMiguel et al.^103^, which compared the 5’- and 3’-LTRs of each full-length element. Nucleotide substitutions per site (*K*) between LTR pairs were calculated using Kimura’s two-parameter model^104^. For truncated LTR-RTs (where no paired LTRs are available), helitrons and TIRs, the calculation of insertion time follows the method proposed by Ou and Jiang^99^, i.e., dating the divergence of each TE copy to its consensus sequence. Following Koch et al. assumptions^105^, we employed the mutation rate (*r*) of 1.5 × 10^−8^ substitutions per year per synonymous site and calculated the insertion times (*T*) of LTR-RTs with the formula *T* = *K*/2*r*.

### FISH experiment and comparative chromosome painting

The mitotic and meiotic (pachytene) chromosome spreads were prepared from young anthers. Oligoprobes (60-bp in length) were used to visualize the identified tandem repeats on chromosomes (Supplementary Data 8). The most conserved regions within the consensus sequences of monomers were selected, with a preference for regions with low GC content (30–50%) and minimal self-annealing. For chromosomal localization of conserved genomic blocks^32^, *A. thaliana* BAC clones were assembled to represent the 22 GBs of the ancestral crucifer karyotype. DNA probes were labeled with biotin-dUTP, digoxigenin-dUTP, or Cy3-dUTP by nick translation^106^. Labeled BAC DNAs were pooled, precipitated, and resuspended in 20 μl of hybridization mixture (50% formamide and 10% dextran sulfate in 2×SSC) per slide. Labeled probes and chromosomes were denatured together on a hot plate at 80 °C for 2 min and incubated in a moist chamber at 37 °C for 16–72 h. Post-hybridization washing was performed in 20% formamide in 2 × SSC at 42 °C. Fluorescence signals were analyzed with an Axioimager Z2 epifluorescence microscope (Zeiss) and CoolCube CCD camera (MetaSystems).

### Phylogenetic analysis

To infer the ancestral origin of the identified syntenic fragments, gene trees were inferred using maximum likelihood (ML) and coalescent-based analyses for each fragment. The protein-coding genes of eight *Biscutella* species and 15 other Brassicaceae species (*Aethionema arabicum*, *Euclidium syriacum*, *Arabis alpina*, *P. cornutum*, *E. salsugineum*, *Schrenkiella parvula*, *Br. rapa*, *Car. hirsuta*, *Boechera stricta*, *Arabidopsis halleri*, *A. thaliana*, *Lunaria rediviva*, *Ricotia lunaria*, *M. pygmaea*, and *H. bupleurifolia*) were used to generate syntenic gene groups. All genomes were compared against 22 conserved GBs using SynOrths (v1.5)^94^. For each GBs, the *Biscutella* generally retained two syntenic copies, both of which were preserved to construct subgenome-aware phylogenetic trees. For other species, we selected the best syntenic match for orthogroup construction. Finally, we obtained 1234 syntenic gene families present across all analyzed genomes. For each gene group, coding sequences were aligned using MAFFT (v7.427)^107^ and trimmed using TrimAL (v1.4)^108^. ML analyses were performed using IQ-TREE (v1.6.11)^109^ with default parameters. Coalescent-based inference of the species tree was performed using ASTRAL-Pro (v1.1.3)^110^, which allows species-tree inference in the presence of paralogy. To further visualize single-gene tree conflicts, the Python package Toytree (v.3.0.5)^111^ was utilized in the construction of cloud tree plots.

### Subgenome phasing and validation

Using the 22 GBs as the reference, two genomic copies of each diploid chromosome in the tetraploid ancestor of *Biscutella* were identified. To reconstruct the two subgenomes, three key rules were applied: (i) no overlapping or redundant regions were allowed within each reconstructed chromosome of the subgenomes; (ii) each ancestral chromosome adhered to the gene density distribution pattern (LF  >  MF) between the subgenomes; and (iii) the phylogenetic relationship of the syntenic gene fragments in the 22 GBs was consistent with the two subgenomic branches. Based on these rules, syntenic fragments were categorized into two groups: the less fractionated (LF) and more fractionated (MF) subgenomes, following the nomenclature for *Brassica* subgenomes^94^. To validate the subgenome phasing, hierarchical clustering of subgenome-specific repetitive DNA sequences and principal component analysis were performed using SubPhaser (v1.2)^112^.

### Calibration of the WGD timing

We performed self-to-self BLASTP alignments for *Br. rapa*, *A. thaliana*, *L. rediviva*, *R. lunaria*, *M. pygmaea*, and *H. bupleurifolia*, respectively, and selected the best hits among the homologous gene pairs with identity >=90%. Paralogues from the eight *Biscutella* species and homologs from other species were used for the non-synonymous (*K*a) and synonymous (*K*s) rate calculations. Each pair of protein sequences was aligned by MAFFT and pairwise nucleotide sequence alignments were generated by transforming protein alignments into codon alignments with ParaAT (v2.0)^113^. The *K*s values and *K*a/*K*s ratios were calculated based on the Nei–Gojobori method implemented in KaKs_Calculator (v2.0)^114^. Timing of subgenome divergence was calculated according to the formula *T* = *K*/2*r*, where *r* is the mutation rate of 1.5 × 10^−8^ substitutions per year per synonymous site^104^.

To further validate divergence times for *Biscutella*, four time-calibrated trees were constructed using r8s (v1.81)^115^, PATHd8 (v1.0)^116^, RelTime method in MEGA X^117^, and MCMCtree (v4.9)^118^, respectively. A total of 94 single-copy ortholog groups, identified using OrthoFinder (v2.5.4)^119^, were used to construct an ML phylogenetic tree with IQ-TREE (v1.6.11)^109^. The divergence at 20.6 Mya between crucifer Lineage I and Lineage II (http://www.timetree.org/) was used as the calibration node.

### Reconstruction of the ancestral karyotype

We utilized both top-down and bottom-up strategies to reconstruct the ancestral *Biscutella* karyotype. The top-down strategy involved comparing GB associations in *Biscutella* species with established ancestral karyotypes of the Brassicaceae (ACK, ancPCK, and PCK)^25,31,32,34^. Conserved GB associations shared among multiple species were considered to be inherited from their ancestral genome. For validation, we utilized the WGDI tool (v0.6.5)^35^ following a bottom-up strategy, which is applicable to species with unknown ancestral karyotypes, as applied in Lamiales^120^ and Buxales^121^. Synteny maps generated by WGDI among *Biscutella* species enabled the inference of ancestral karyotypes at various evolutionary nodes based on phylogeny (Fig. 3b). Intact chromosomes with continuous synteny were initially identified as ancestral chromosomes. Chromosomal breaks or fusions shared by multiple species, as well as those unique to particular species, were systematically characterized. We traced the origins of these breaks or fusions for each species hierarchically and considered six typical CR types, i.e., EET, RT, unbalanced-RT, NCI, Ipa, and Ipe, which allowed us to reconstruct the karyotype for each node sequentially, progressing from the youngest to the oldest (nodes A1 to A5; Supplementary Figs. 14–17). The CR rate was calculated as the total number of CRs divided by the elapsed time between any two nodes, The elapsed time is rounded to get integers, and those less than 1 Mya are calculated as 1 Mya (Supplementary Data 9).

### Identification of chromosome breakpoints

Chromosomal rearrangements may result in breakage of ancestral chromosomes, generating synteny breakpoints that manifest as rejoined junctions between syntenic fragments in extant *Biscutella* genomes. Each breakage and subsequent rejoining of the ancestral chromosomal fragments theoretically produces two rejoined junctions (except for terminal fragments), corresponding to the both ends of ancestral chromosome fragments. To trace these events, WGDI^35^ was utilized to generate syntenic gene dot plots between the eight species and the reconstructed ancestral *Biscutella* genome (using the gene order of *B. baetica* for karyotype projection). Synteny analyses between five putative ancestral genomes at five phylogenetic nodes (A1 to A5) and the reconstructed ancestral *Biscutella* genome were also performed. For each candidate rejoined junctions, we retrieved 20 genes upstream and 20 genes downstream (40 in total). If more than 10 genes on either side showed conserved synteny between any two species, the breakage was considered likely to have occurred at the same ancestral position and is incorporated into downstream analyses. Based on the presence and absence of breakpoints across the *Biscutella* genomes and five phylogenetic nodes, the identified 14 breakpoints were classified into two categories: (i) ancestral breakpoints, present exclusively in a single phylogenetic clade, and (ii) recurrent breakpoints, shared by species from at least two independent phylogenetic clades. To mitigate the influence of local gene rearrangements or potential assembly errors, we analyzed the genomic regions spanning the adjacent border genes of two rejoined blocks, along with 100-kb of flanking sequences on each side (total length = 200-kb + the inter-boundary interval). When one end of a synteny block was located in a (peri)centromeric region, we extracted the boundary gene of the rejoined block plus a 100-kb downstream (or upstream; depending on the orientation of the blocks) sequence, along with 100-kb of flanking sequences on each side (total length = 300-kb). Using these criteria, breakpoint regions were defined for each genome and subsequently analyzed for their overlap with (peri)centromeres and TAD boundaries, as well as their distribution across A/B chromatin compartments. To generate a null expectation, we randomly sampled 200 genomic regions per genome, matching the length of breakpoint regions. This random sampling procedure was repeated three times for each genome, resulting in three replicate datasets. For each genome and each replicate, we constructed a 2 × 2 contingency table based on the number of regions that overlapped or did not overlap the genomic features of interest (i.e., (peri)centromeres, TAD boundaries, or A/B compartments) in breakpoint and random sets. Statistical significance was assessed using two-sided Fisher’s exact tests. To evaluate the overall trend across all genomes, the overlap frequencies of breakpoint regions and their corresponding random controls were treated as paired observations (*n* = 18, 6 genomes × 3 replicates). These paired values were compared using a two-sided Wilcoxon signed-rank test to assess whether breakpoint regions consistently showed higher overlap frequencies than expected by chance.

### Gene family classification and functional enrichment

We classified orthologous gene families identified by SynOrths across eight *Biscutella* species into core, softcore, and private categories. Genes with two copies in both subgenomes of *Biscutella* species were considered as duplicates, and the remaining genes were classified as singletons. Thus, five types of gene sets, i.e., core duplicates shared in all eight *Biscutella* species (CD), core singletons shared in all eight *Biscutella* species (CS), softcore duplicates and singletons shared in late-diverging clade comprised by all *n* = 9 species or in early-diverging species comprised by *B. auriculata*, *B. didyma*, *B. baetica*, and *B. lyrata* (SD and SS), and private singletons specific to a certain species (PS), were further subjected to GO functional enrichment analysis. The GO terms and pathways of gene enrichment were identified by the clusterProfiler package (v3.14.3)^122^. GO enrichments were estimated using one-sided Fisher’s exact tests, and an adjusted *P*-value < 0.05 was set as the cutoff criterion for the significance of the gene enrichment.

The Pfam protein domains identified by InterproScan^93^ were used to examine the conserved domains of 1049 CD gene families. We assumed that gene duplicates could either split functions (subfunctionalization) or generate a new function (neofunctionalization)^123^. For each CD gene family, if both copies retained identical protein domain(s), we considered that the domain(s) inherited from the ancestral gene, and if one copy lost the conserved domain or diverged into a new domain distinct from the other, we considered that the gene undergone sub/neofunctionalization. Based on these criteria, we identified 220 species-specific (protein domains in a certain species differ from others) gene families that underwent potential sub/neofunctionalization.

### Calculation of the loss rate for syntelogs

We investigated 12 syntenic gene families, including class A genes *APETALA1* and *2* (*AP1* and *AP2*), class B genes *APETALA3* (*AP3*) and *PISTILLATA* (*PI*), class C gene *AGAMOUS* (*AG*), class D genes *SEEDSTICK* (*STK*), *SHATTERPROOF1* and *2* (*SHP1* and *SHP2*), and class E genes *SEPALLATA1*, *2*, *3*, and *4* (*SEP1 – 4*), belonging to the ABCDE model^47,48^ using the *A. thaliana* genome as a reference, and the diploid genomes *Bo. stricta*, *Capsella rubella*, *Car. hirsuta*, *M. pygmaea*, and *S. parvula*, the mesotetraploid genomes *P. cornutum*, *Biscutella*, and the mesohexaploid genome *Br. rapa* as queries. The expected number of copies retained per gene family was predetermined based on the ploidy level of each species: one copy in diploid species, two copies in mesotetraploid species, and three copies in mesohexaploid species. The loss rate of syntelogs (*r*) was then calculated using the formula: *r* = 1 – (Actual copy number/Expected copy number).

### TE distribution in neighboring regions of genes

The TE density around genes was calculated using the following method^124^: the 5-kb upstream and downstream regions of each gene were defined as flanking sequences (totaling 10-kb). Flanking sequences overlapping with the coding regions of adjacent genes were hard-masked as Ns. A 50-bp sliding window with a 10-bp step size was used to scan these flanking regions and gene bodies. The TE density for each gene was then determined as the proportion of TE-derived bases across all sliding windows within gene bodies and its flanking sequences.

### 3D genome analysis

Hi-C sequencing reads were mapped to their corresponding reference genomes using BWA-MEM (v0.7.17)^125^ with the parameters “-A1 -B4 -E50 -L0”. Hi-C contact matrices were generated at multiple resolutions (10, 25, 50, and 100 kb) using the hicBuildMatrix option in HiCExplorer (v3.7.2)^126^ with the parameters “--restrictionSequence GATC --danglingSequence GATC --binSize 10000 20000 50000 100000”. Matrix correction was performed using the hicCorrectMatrix option, with filtering thresholds (“--filterThreshold −1.5 5”). The contact matrix plots for each chromosome were generated using hicPlotMatrix, and the 50-kb resolution matrices were selected for visualization and downstream analyses (Fig. 4d and Supplementary Fig. 19). A/B compartments were identified using the hicPCA option in HiCExplorer, which computes Pearson correlation matrices from the raw contact matrices (50-kb resolution), followed by calculation of covariance matrices. The eigenvectors of the covariance matrices were used to assign genomic regions to compartments. Manual examination of compartment assignments for each chromosome was performed to determine the final eigenvector orientation, where negative values corresponded to B compartments (gene-poor regions) and positive values corresponded to A compartments (gene-rich regions). Based on genome-wide compartment assignments, we examined whether orthologous gene pairs between different species and paralogous gene pairs within species shared identical or distinct compartment assignments. TAD-like structures were identified from Hi-C data using a separation score–based approach implemented in the hicFindTADs option of HiCExplorer, with the parameters “--thresholdComparisons 0.01 --delta 0.01 --correctForMultipleTesting fdr”. The overlap between TADs, A/B compartments, and subgenomes was calculated, and TADs were able to be categorized as entirely within the A compartment (or LF subgenome), entirely within the B compartment (or MF subgenome), or spanning both compartments (or subgenomes). Conserved TADs between species were identified following the method of Shen et al.^61^, in which two TADs from different species were considered conserved if syntenic gene pairs were found within 100 kb on both sides of their boundaries.

### Reporting summary

Further information on research design is available in the Nature Portfolio Reporting Summary linked to this article.

## Supplementary information

Supplementary Information

Descriptions of Additional Supplementary Files

Supplementary Data 1-14

Reporting Summary

Transparent Peer Review file

## Source data

Source Data

## Data Availability

The genome assemblies and annotations generated in this study have been deposited in the National Genomics Data Center under BioProject number PRJCA060288 [https://ngdc.cncb.ac.cn/gwh/search/advanced/result?search_category=&search_term=&source=0&query_box=PRJCA060288] and Figshare (10.6084/m9.figshare.28451828). The raw DNA and RNA sequencing data generated in this study have been deposited in the NCBI Sequence Read Archive under BioProject numbers PRJNA1439612 [https://www.ncbi.nlm.nih.gov/bioproject/PRJNA1439612/] and PRJNA1124645 [https://www.ncbi.nlm.nih.gov/bioproject/PRJNA1124645/]. Additionally, we incorporated previously published sequencing data: DNA and RNA sequencing data for *B. austriaca* from the European Nucleotide Archive under BioProject number PRJNA646339 [https://www.ncbi.nlm.nih.gov/bioproject/PRJNA646339/], and RNA sequencing data for *B. baetica* and *B. lyrata* from NCBI SRA under BioProject number PRJNA634714 [https://www.ncbi.nlm.nih.gov/bioproject/?term=PRJNA634714]. All accession numbers are provided in Supplementary Data 14. All other data needed to evaluate the conclusions in this study are provided in the Supplementary Information/Source Data file. Source data are provided with this paper.

## Code availability

The code used to perform soloLTR analysis has been deposited in GitHub at https://github.com/estpr/soloLTRseeker. All other software used, along with specific versions and references, are provided in the Supplementary Information file.
