# Supplementary material for: Post-polyploid chromosomal diploidization in plants is affected by clade divergence and constrained by shared genomic features
Source: Nat Commun. 2026 Jun 6;17:8244. doi: 10.1038/s41467-026-73793-8 (PMC13462695; doi:10.1038/s41467-026-73793-8)
Supplement: Supplementary file 1 [file 41467_2026_73793_MOESM1_ESM.pdf]

Supplementary Information for

Post-polyploid chromosomal diploidization in plants is affected by clade divergence and constrained by shared genomic features

Yile Huang, Manuel Poretti, Terezie Mandáková, Milan Pouch, Xinyi Guo, Hussein Anani, Estela Perez-Roman, Manuel B. Crespo, Stefan Grob, Alexandros Bousios, Christian Parisod, Martin A. Lysak

Supplementary Figures 1 – 27

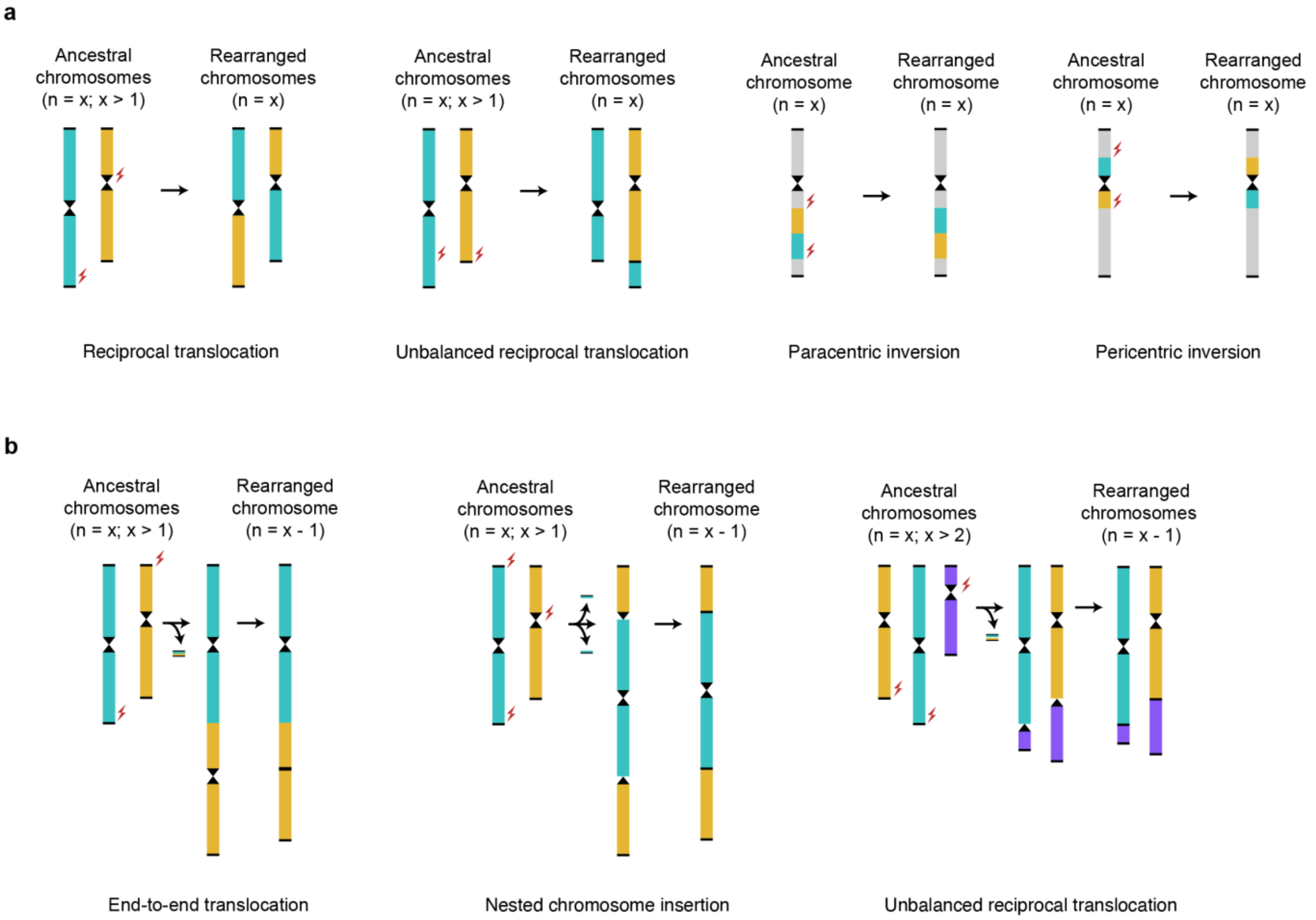

Supplementary Fig. 1 | Proposed non-dysploid (**a**) and dysploid (**b**) chromosomal rearrangement (CR) types in angiosperms (adopted from Mandáková & Lysak[1]). (**a**) Non-dysploid CRs comprise at least four types, including reciprocal translocation (RT), single unbalanced reciprocal translocation (unbalanced-RT), paracentric and pericentric inversions (Ipa and Ipe). RT involves the breakage of two non-homologous chromosomes. The double-strand breaks (DSBs) are labelled by red arrows. The broken segments are exchanged, forming two translocation chromosomes. In contrast to RTs, unbalanced-RTs involve the unidirectional transfer of one or more segments from a donor chromosome to the recipient(s) (note that unidirectionality can result from the insufficient resolution of analytical methods to detect very short DNA sequence exchanged between the two chromosomes). While single unbalanced-RT do not alter chromosome number (non-dysploid unbalanced-RT), two or more unbalanced-RTs can redistribute segments from a single donor to multiple recipient chromosomes, ultimately resulting in complete relocation of segments of the donor chromosome (dysploid unbalanced-RTs; see (**b**)). Ipa occurs within a chromosome arm, without involving the centromere, while Ipe involves the centromere. (**b**) End-to-end translocations (EETs) represent the fusion of two non-homologous chromosomes via DSBs at chromosome ends, resulting in the formation of a dicentric fusion chromosome. To ensure chromosome stability, one of the two centromeres must be inactivated and/or eliminated. Nested chromosome insertions (NCIs) are translocation events in which an "insertion" chromosome becomes integrated into the (peri)centromeric region of a "recipient" chromosome. In descending dysploidy mediated by unbalanced-RTs, repeated unidirectional recombination events (at least twice) occur between a donor chromosome and multiple recipient chromosomes.

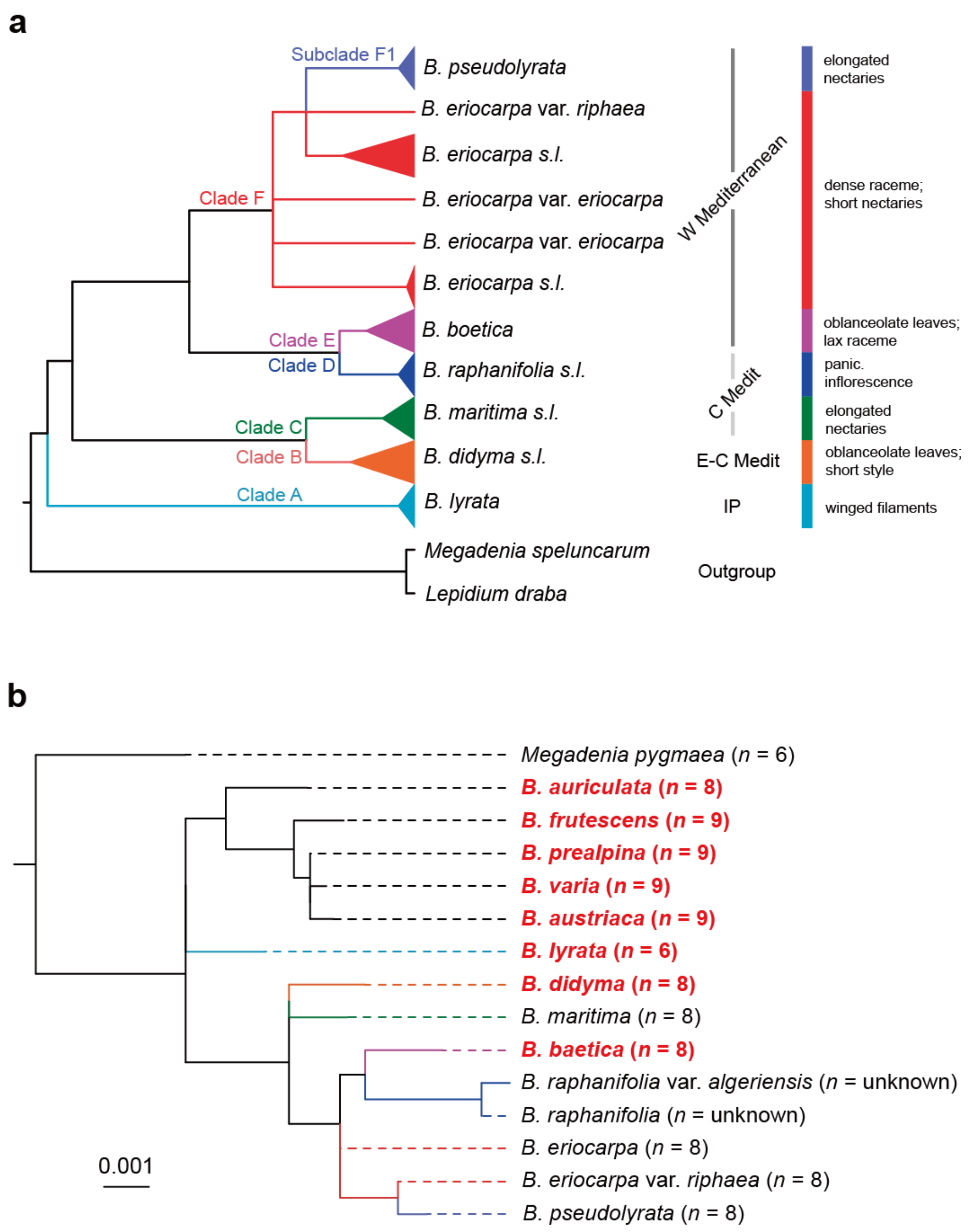

Supplementary Fig. 2 | Phylogenetic relationships between *Biscutella*s species ser. *Biscutella*. (**a**) Condensed maximum likelihood (ML) phylogenetic tree from Alonso et al.[2], inferred from concatenated cpDNA (rpl32-trnL and trnV) and nrDNA (ITS) sequences (final dataset of 47 nucleotide sequences, with a total of 2,254 positions). W Mediterranean: Western Mediterranean; C Medit: Central Mediterranean; E-C Medit: Eastern-Central Mediterranean; IP: Ibérian Peninsula. (**b**) A chloroplast gene (rpl32-trnV) tree constructed based on 1,430 nucleotide sites, using the same clade color scheme as in (**a**). The sequences of eight species in this study (red bold font) were obtained from chloroplast genome assemblies, and the sequences of the remaining species were obtained from Vicente et al.[3]

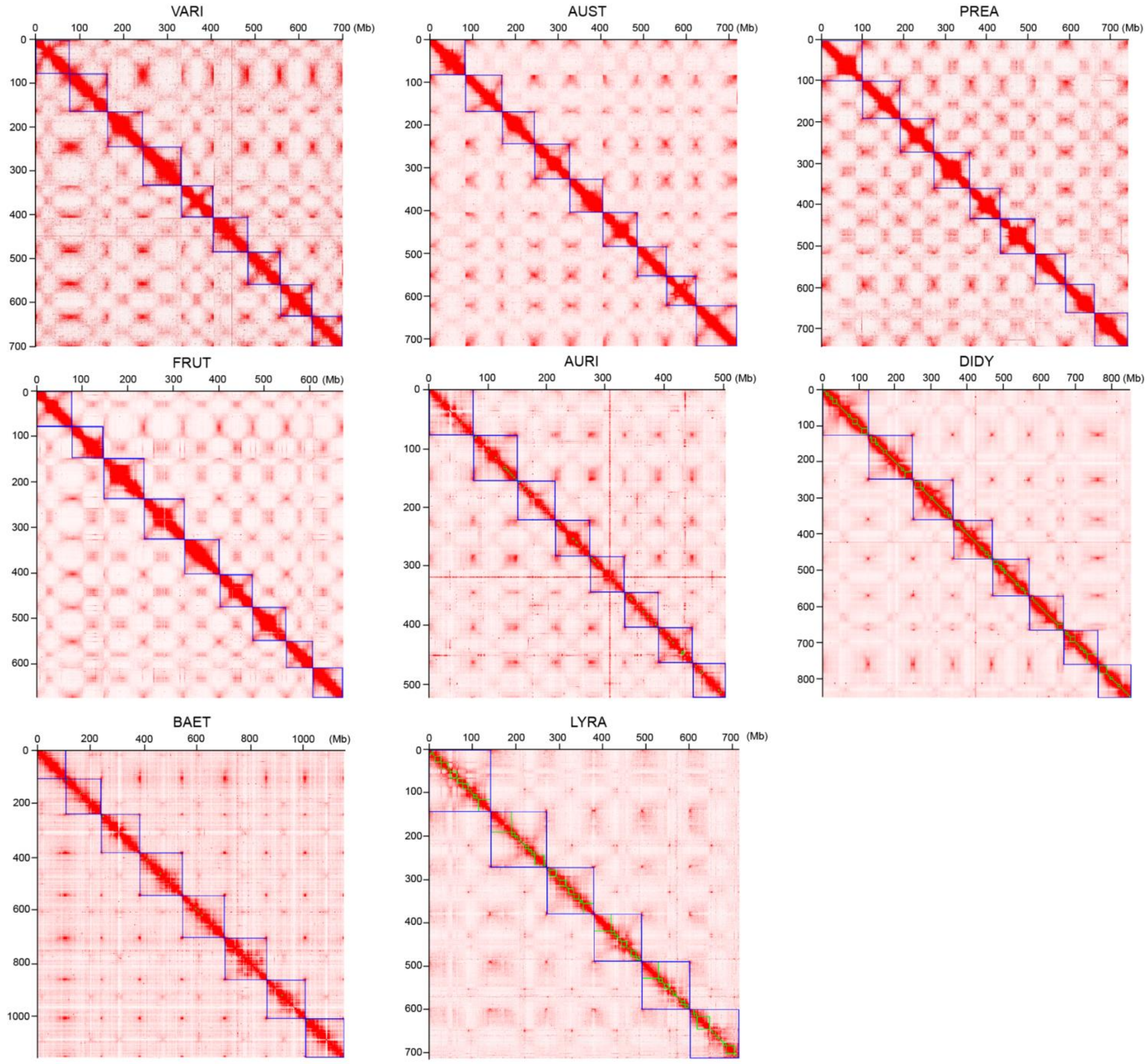

Supplementary Fig. 3 | Genome-wide all-by-all Hi-C interactions between 6 to 9 pseudochromosomes in *Biscutella*. The intensity of the red color is proportional to the interaction frequency. The blue boxes represent either 6, 8, or 9 pseudochromosomes.

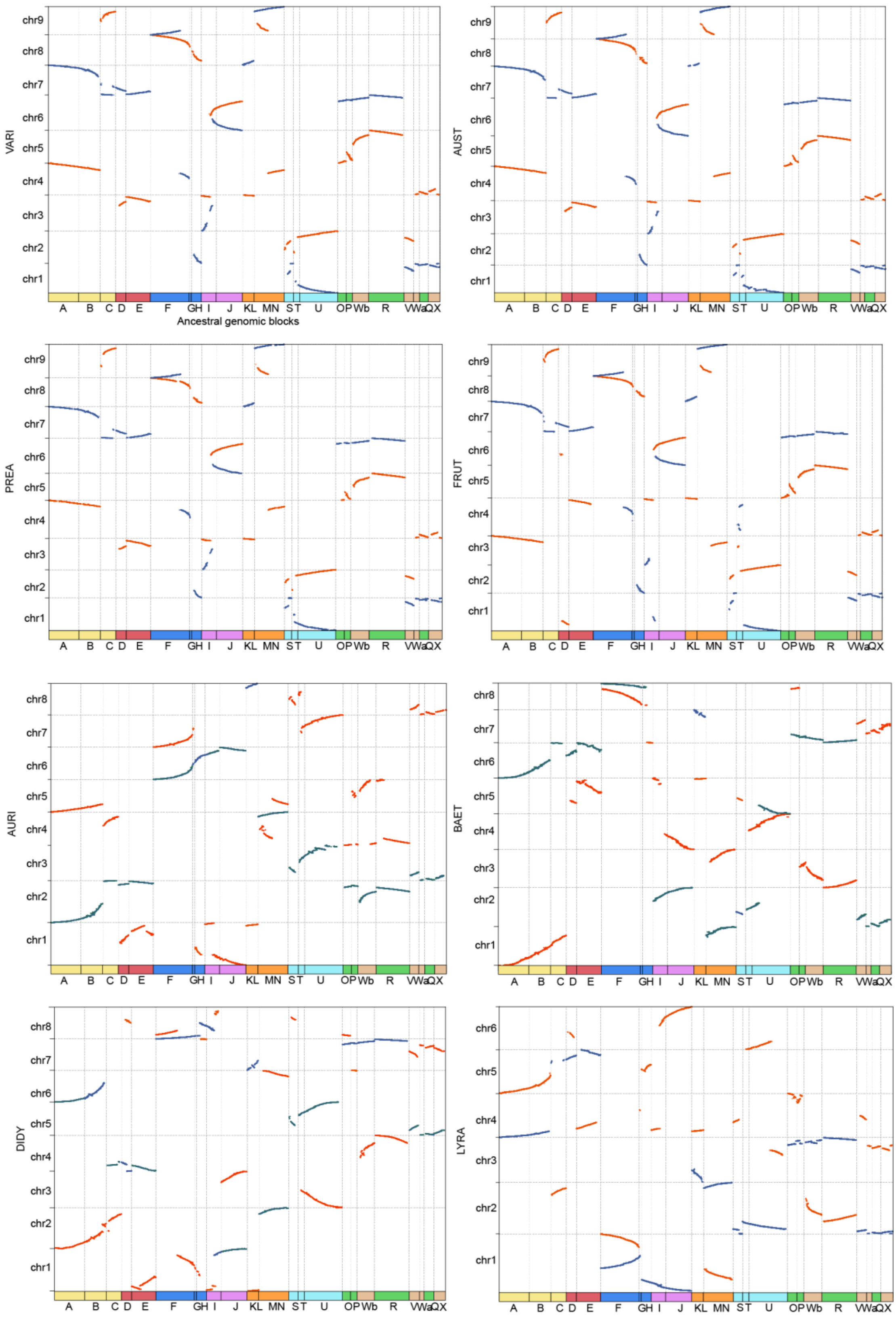

VARI
chr9
chr8
chr7
chr6
chr5
chr4
chr3
chr2
chr1
A B C D E F GH I J KL MN ST U OP Wb R VWaQX
Ancestral genomic blocks
AUST
chr9
chr8
chr7
chr6
chr5
chr4
chr3
chr2
chr1
A B C D E F GH I J KL MN ST U OP Wb R VWaQX
PREA
chr9
chr8
chr7
chr6
chr5
chr4
chr3
chr2
chr1
A B C D E F GH I J KL MN ST U OP Wb R VWaQX
FRUT
chr9
chr8
chr7
chr6
chr5
chr4
chr3
chr2
chr1
A B C D E F GH I J KL MN ST U OP Wb R VWaQX
AURI
chr8
chr7
chr6
chr5
chr4
chr3
chr2
chr1
A B C D E F GH I J KL MN ST U OP Wb R VWaQX
BAET
chr8
chr7
chr6
chr5
chr4
chr3
chr2
chr1
A B C D E F GH I J KL MN ST U OP Wb R VWaQX
DIDY
chr8
chr7
chr6
chr5
chr4
chr3
chr2
chr1
A B C D E F GH I J KL MN ST U OP Wb R VWaQX
LYRA
chr6
chr5
chr4
chr3
chr2
chr1
A B C D E F GH I J KL MN ST U OP Wb R VWaQX

Supplementary Fig. 4 | Syntenic relationships between 6, 8 or 9 chromosomes of *Biscutella* genomes and 22 ancestral genomic blocks (GBs A – X)[4]. Two genomic copies are indicated in orange and blue, respectively.

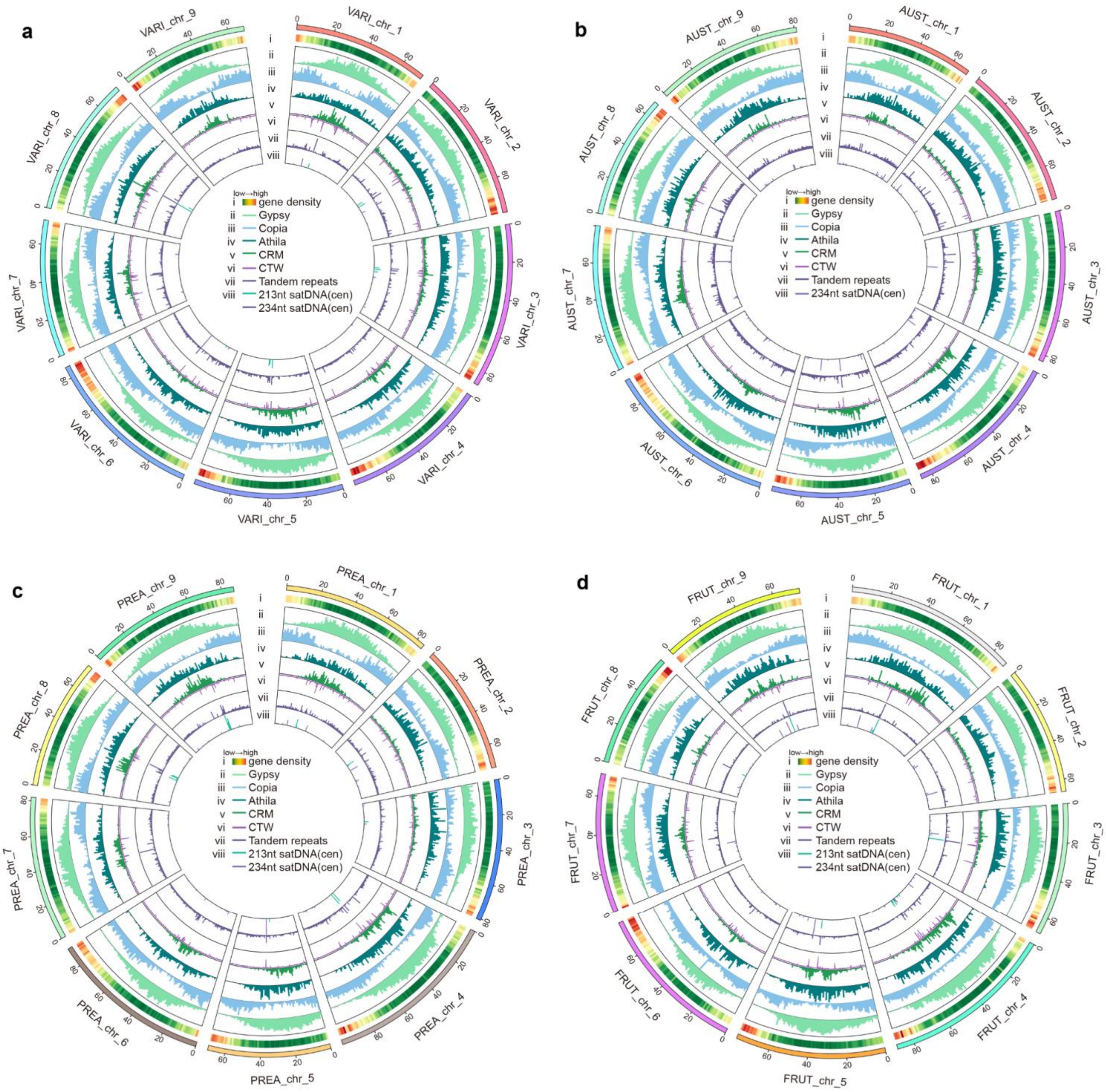

Supplementary Fig. 5 | Distribution of genes and repeats in *B. varia* (**a**), *B. austriaca* (**b**), *B. prealpina* (**c**), and *B. frutescens* (**d**). The circles from outside to inside show (i) the gene density; (ii) the density of Gypsy retrotransposons; (iii) the density of Copia retrotransposons; (iv) the density of Athila retrotransposons; (v) the density of CRM retrotransposons; (vi) DNA compression (CTW); (vii) the density of all tandem repeats; (viii) the density of 213- and 234-bp centromeric (cen) tandem repeats.

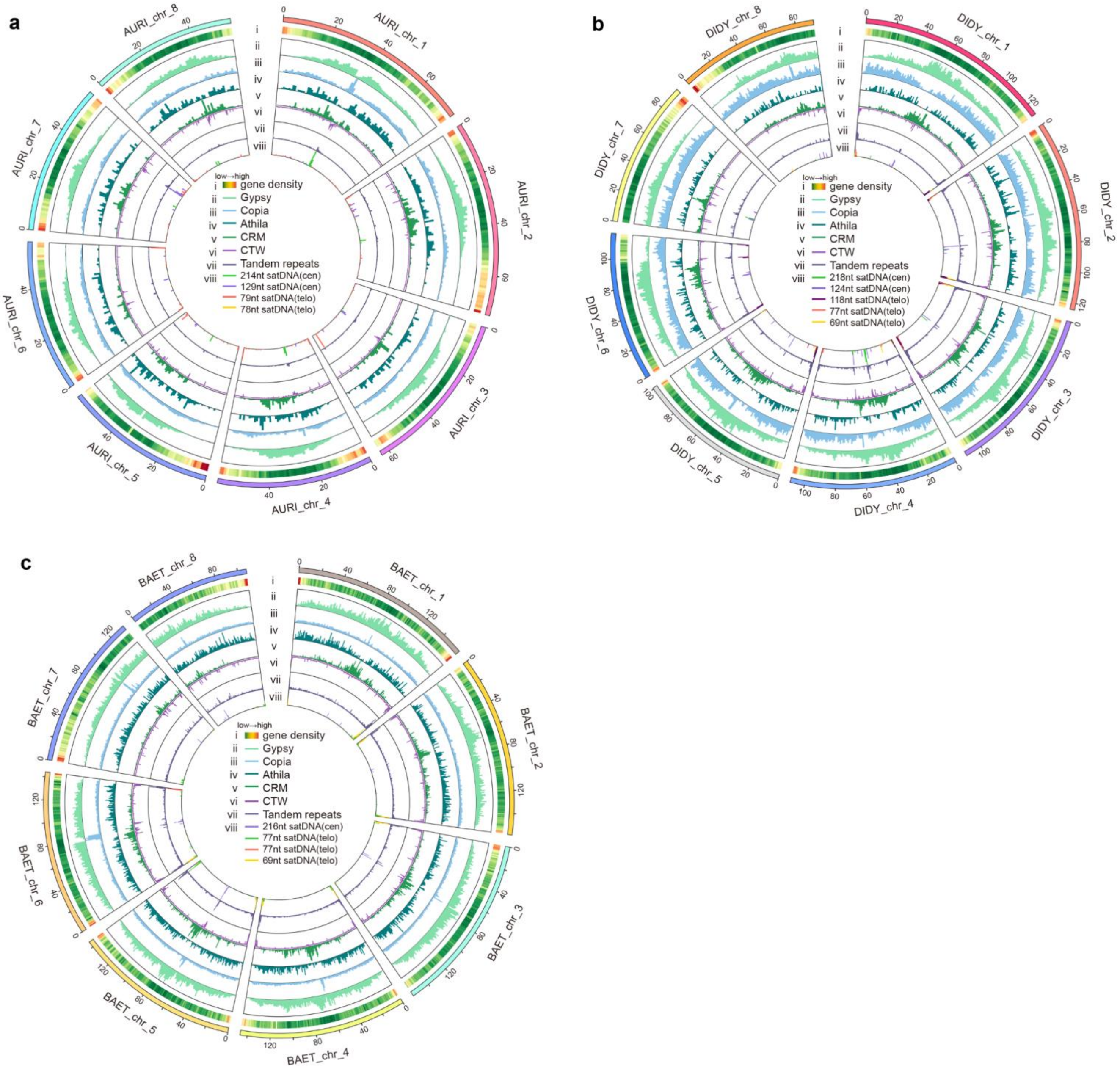

Supplementary Fig. 6 | Distribution of genes and repeats in *B. auriculata* (**a**), *B. didyma* (**b**), and *B. baetica* (**c**). The circles from outside to inside show (i) the gene density; (ii) the density of Gypsy retrotransposons; (iii) the density of Copia retrotransposons; (iv) the density of Athila retrotransposons; (v) the density of CRM retrotransposons; (vi) DNA compression (CTW); (vii) the density of all tandem repeats; (viii) the density of the identified centromeric (cen) and subtelomeric (telo) tandem repeats.

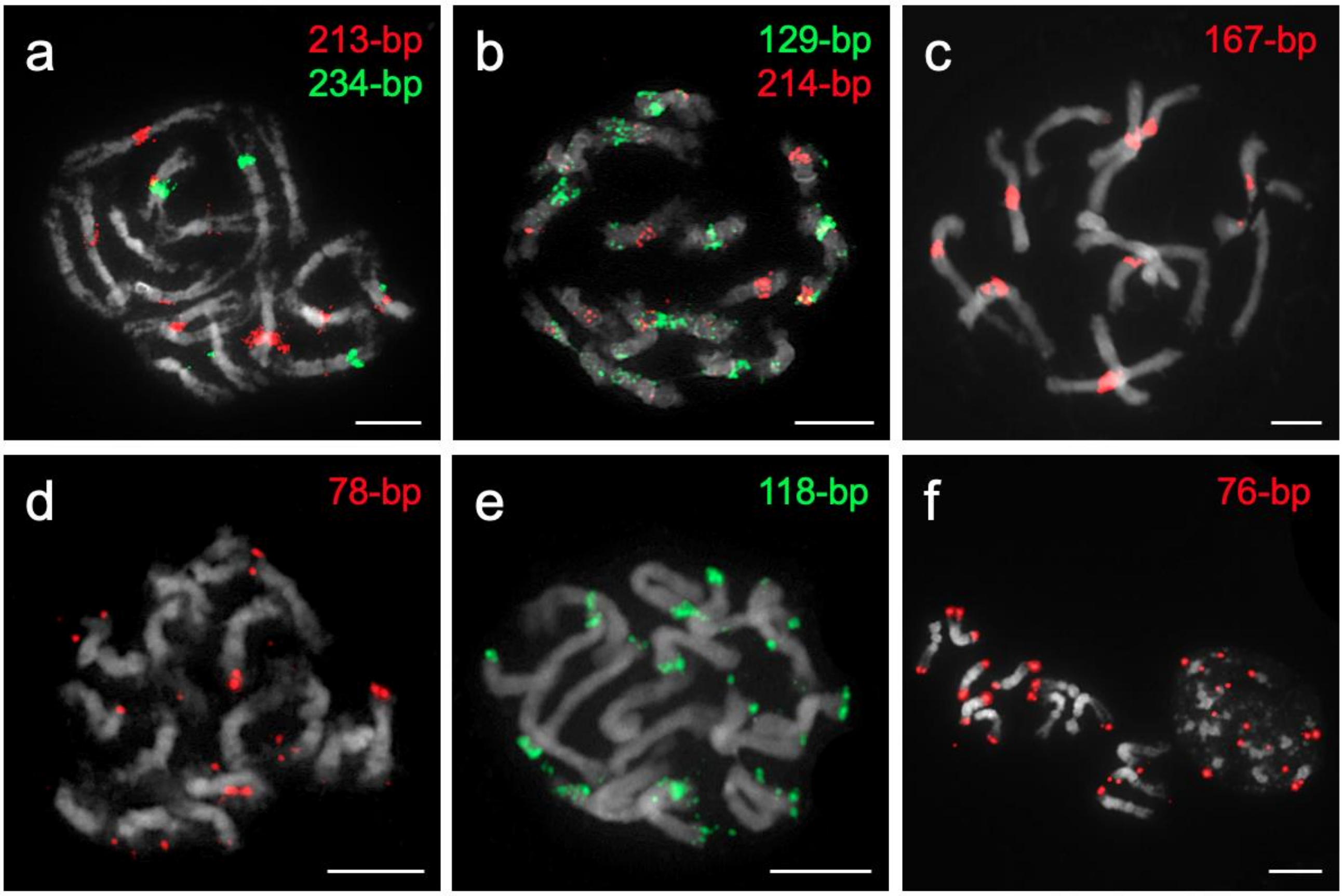

Supplementary Fig. 7 | FISH localization of the selected tandem repeats on mitotic metaphase chromosomes in *Biscutella* species. (**a**) 213- and 234-bp centromeric tandem repeats in *B. varia*. (**b**) 129- and 214-bp centromeric tandem repeats in *B. auriculata*. (**c**) 167-bp centromeric satDNA in *B. lyrata*. (**d**) 78-bp subtelomeric tandem repeat in *B. auriculata*. (**e**) 118-bp subtelomeric satDNAs in *B. didyma*. (**f**) 76-bp subtelomeric tandem repeats in *B. lyrata*. Chromosomes were counterstained by DAPI; FISH signals are shown in color as indicated. Scale bars, 10 μm.

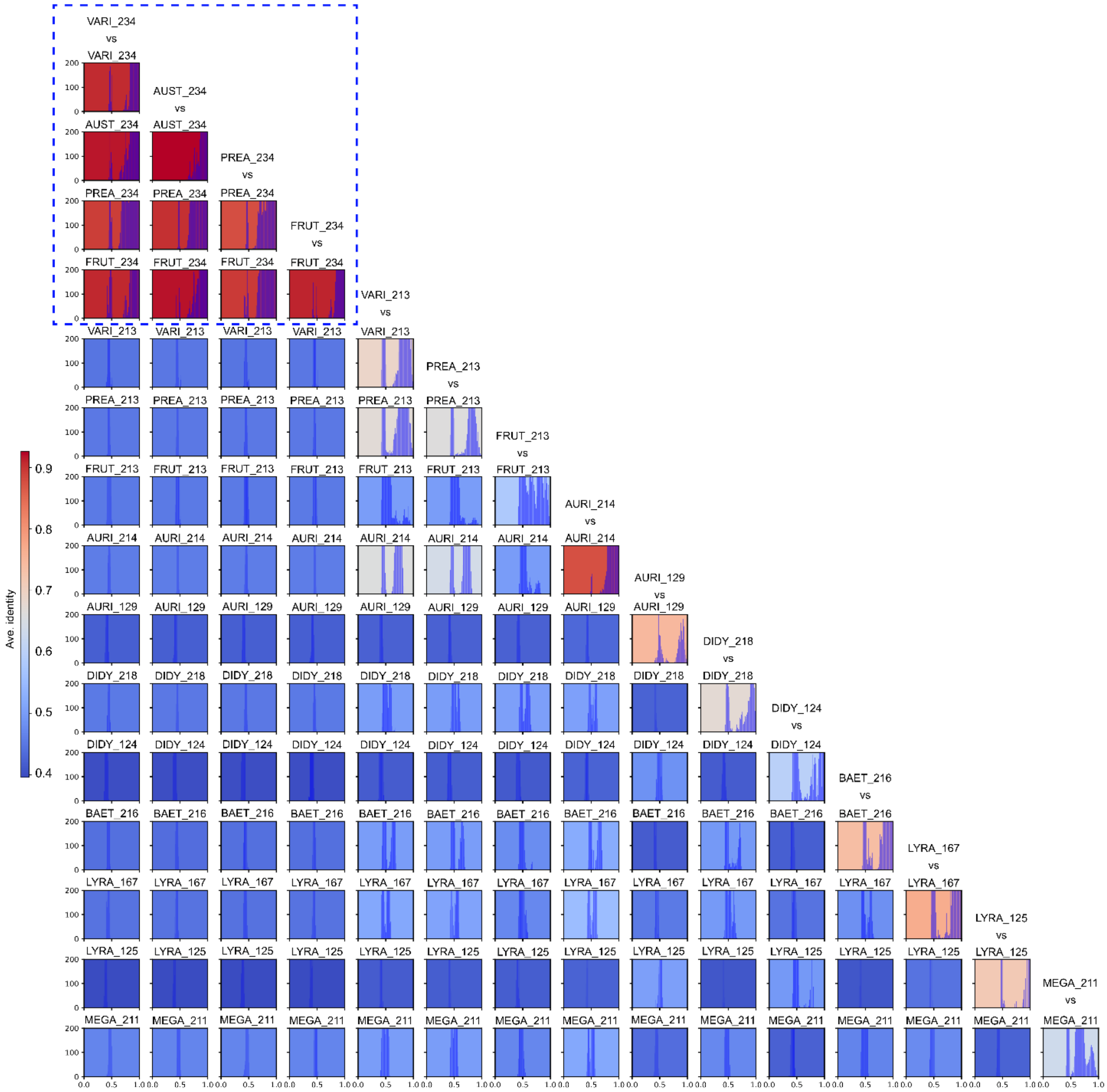

Supplementary Fig. 8 | Pairwise comparison of tandem repeats of different lengths in pericentromeric regions. Shown is a pairwise sequence identity comparison of 15 satDNA variants identified in *Biscutella* species and *Megadenia pygmaea*. Frequency distribution histograms illustrate the sequence identity distribution between each pair of variants, with average identity values indicated by different background colors. The 234-bp satDNA in n = 9 *Biscutella* species shows high similarity and conservation, indicated by blue dashed boxes.

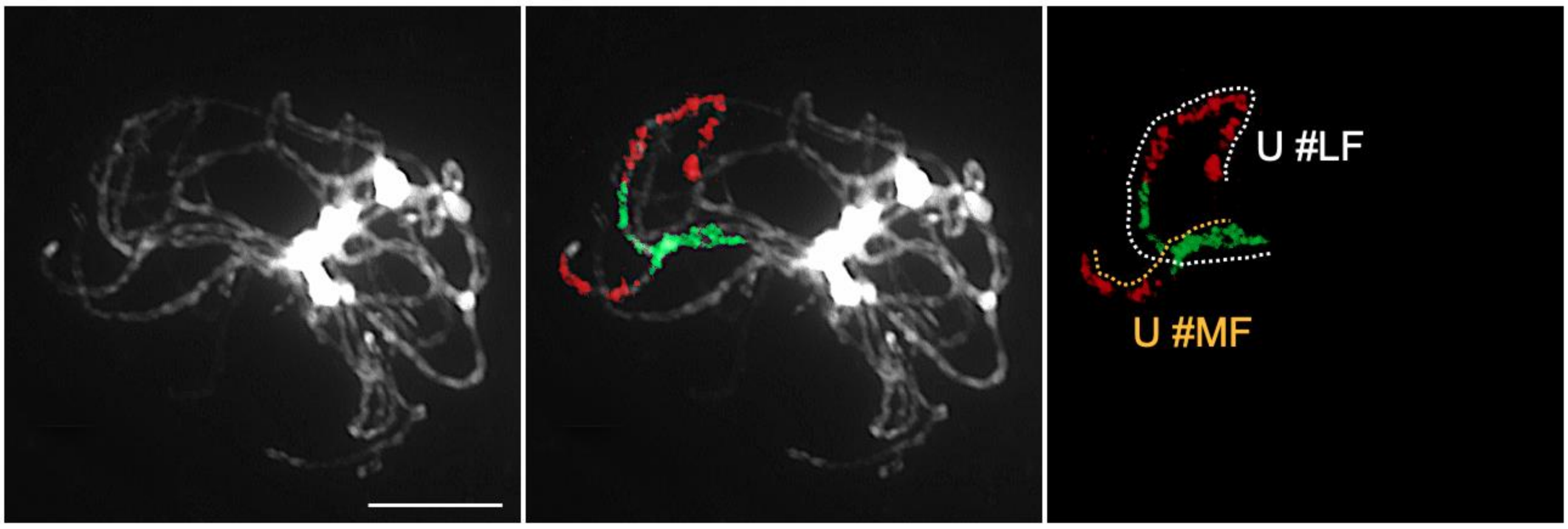

Supplementary Fig. 9 | Cytogenetic evidence for biased subgenome fractionation during post-polyploid diploidization in *B. didyma*. Comparative chromosome painting of ancestral genomic block U using *Arabidopsis thaliana* BAC clones in pachytene complements divided into two differently labeled parts revealed two paralogous copies differing in length and fluorescence intensity. The longer and brighter copy corresponds to the U block in the LF subgenome, the shorter and weaker copy corresponds to the U block in the MF subgenome. The chromosomes were counterstained with DAPI. Scale bar, 10 µm.

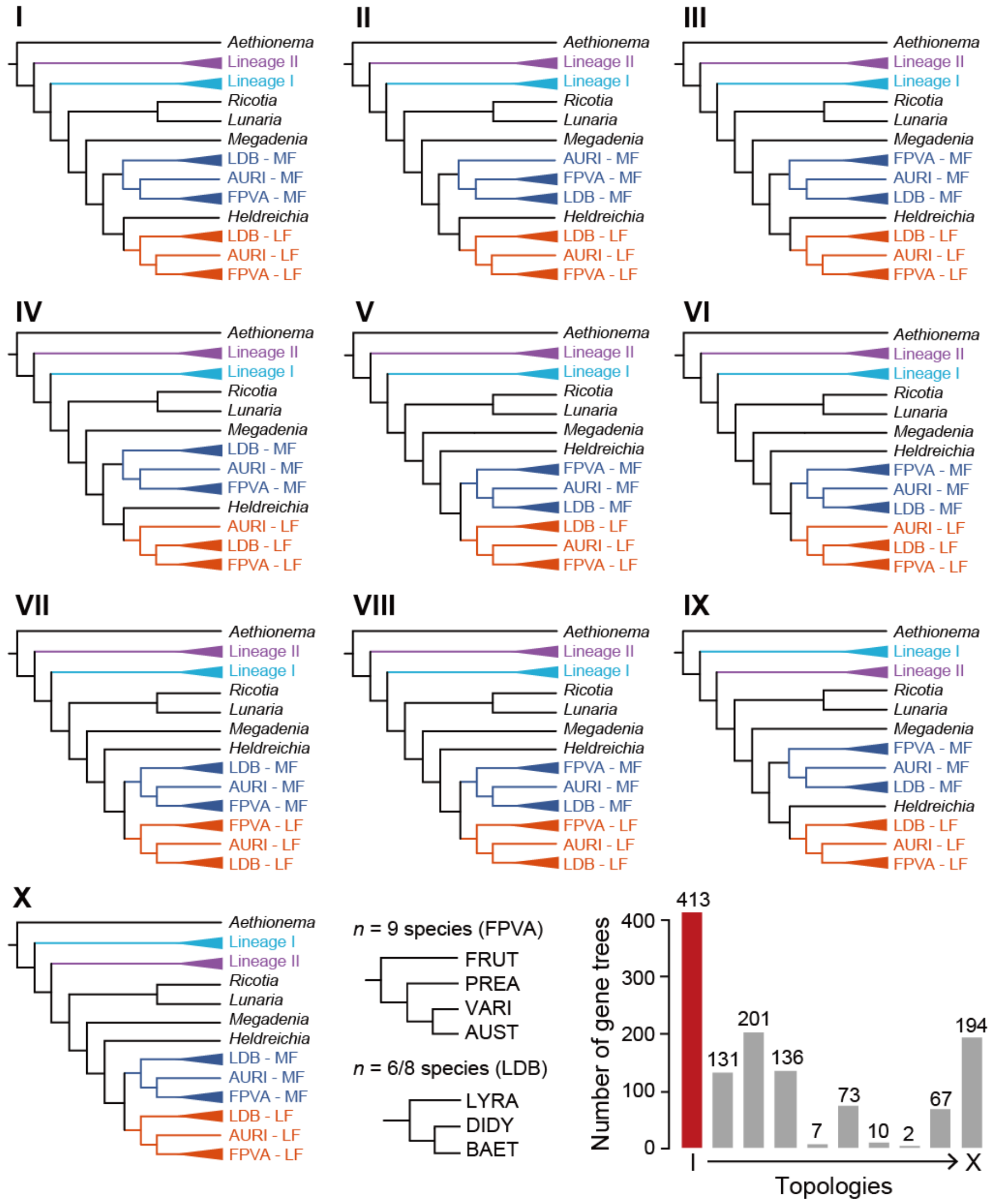

Supplementary Fig. 10 | Alternative tree topologies (I – X) observed in 1,234 syntenic gene trees. The two subgenomes (LF and MF, marked orange and blue, respectively) consistently formed two distinct clades. The n = 9 *Biscutella* species (*B. frutescens*, *B. prealpina*, *B. varia*, and *B. austriaca*, abbreviated as FPVA) and *B. lyrata*, *B. didyma,* and *B. baetica* (abbreviated as LDB) always appeared on separate clades, whereas *B. auriculata* is either sister to FPVA or LDB, or outside of both. The graph in the bottom right corner shows the number of gene trees supporting each topology - topology I was supported by the highest number of gene trees.

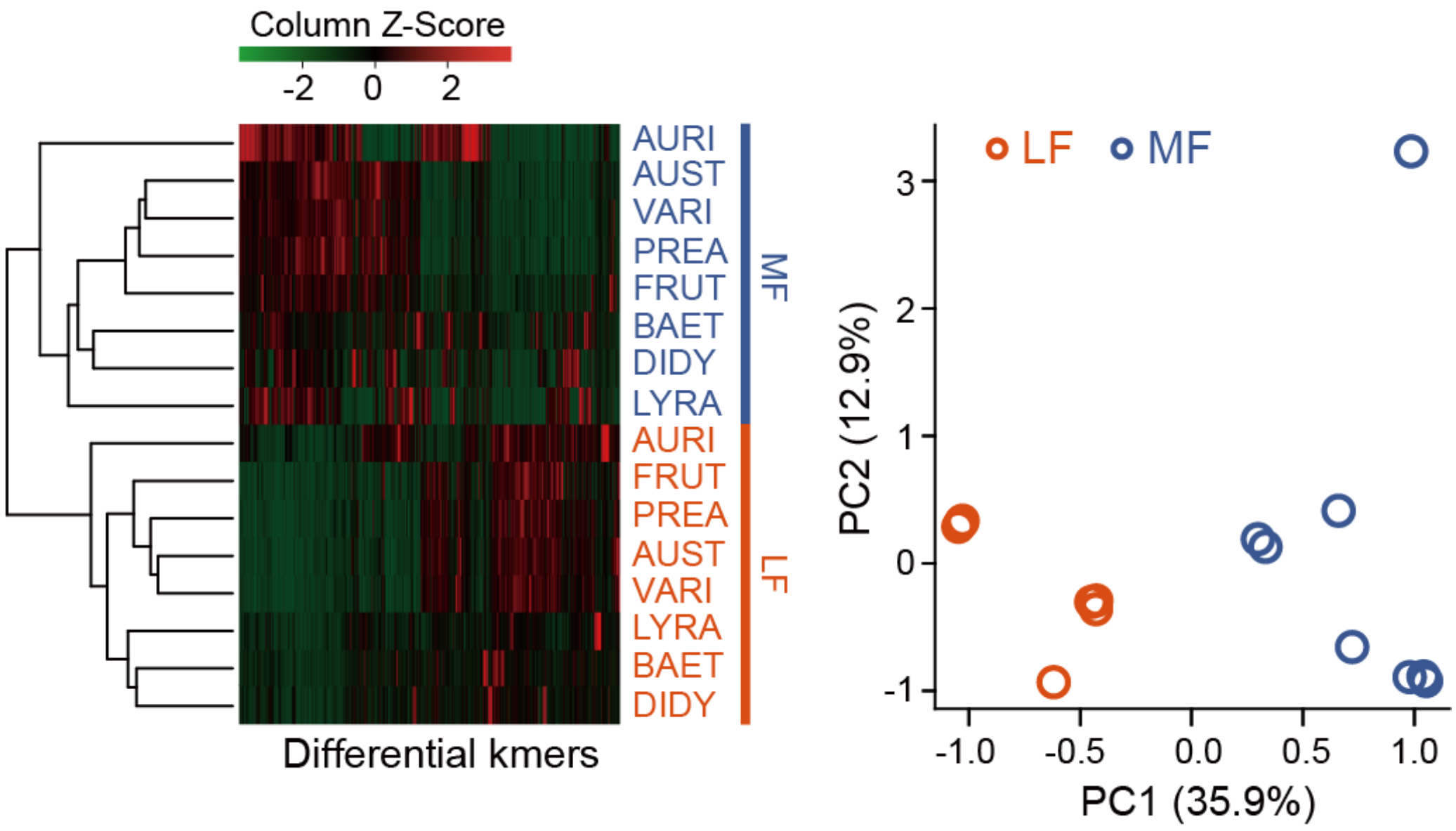

Supplementary Fig. 11 | Hierarchical clustering of 15-mers based on the relative abundance (Z-score; left). The PCA of this data (right) shows distinct clustering of LF and MF subgenomes.

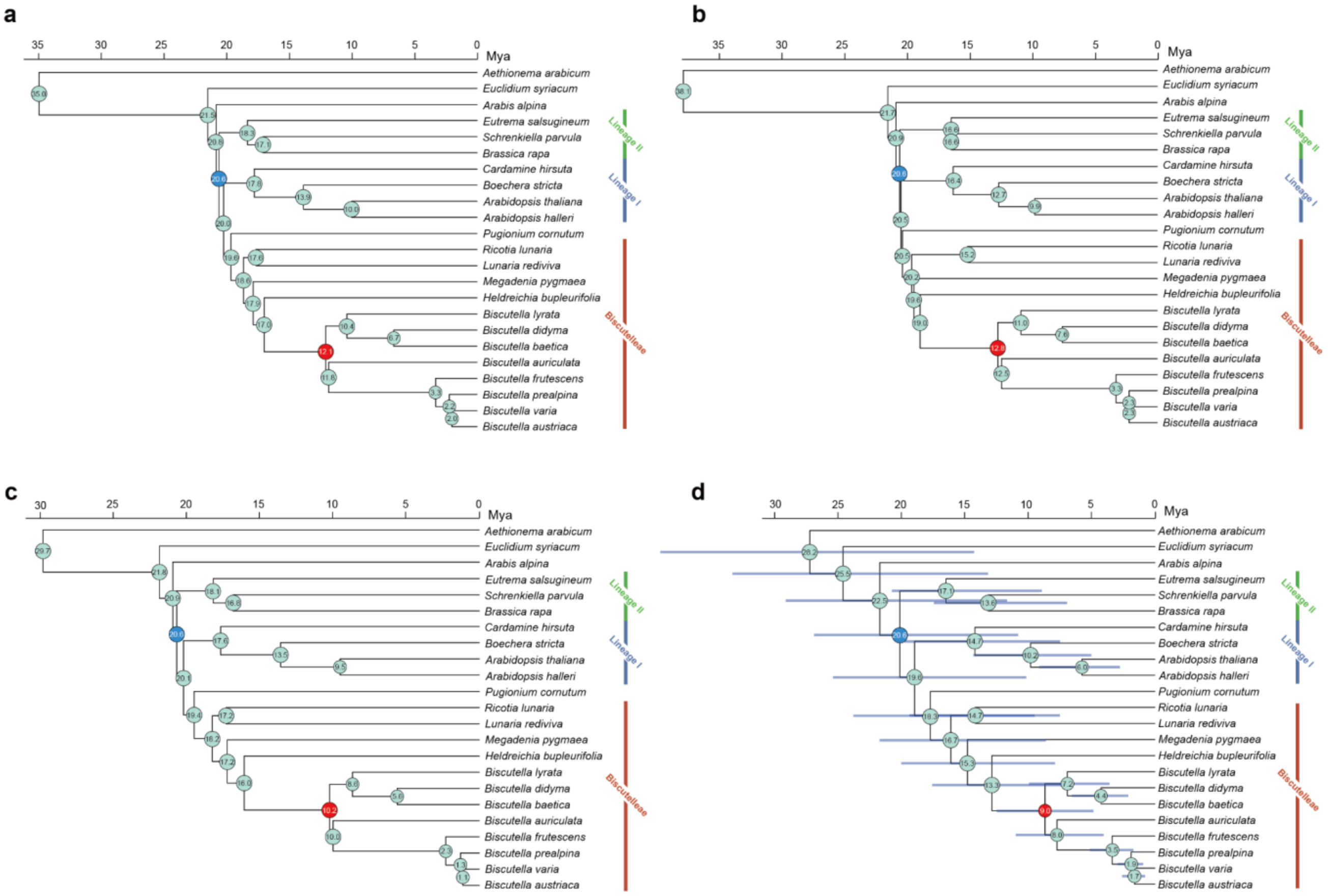

Supplementary Fig. 12 | Time-calibrated trees inferred by r8s (**a**), PATHd8 (**b**), RelTime (**c**), and MCMCtree (**d**) methods. The four different calculating strategies showed high consistency. The divergence time of Lineage I (Camelinodae) and Lineage II (Brassicodae) was used as the calibration node (blue dot at 20.6 Mya, http://www.timetree.org/). The crown node of the *Biscutella* species is labeled as red dots.

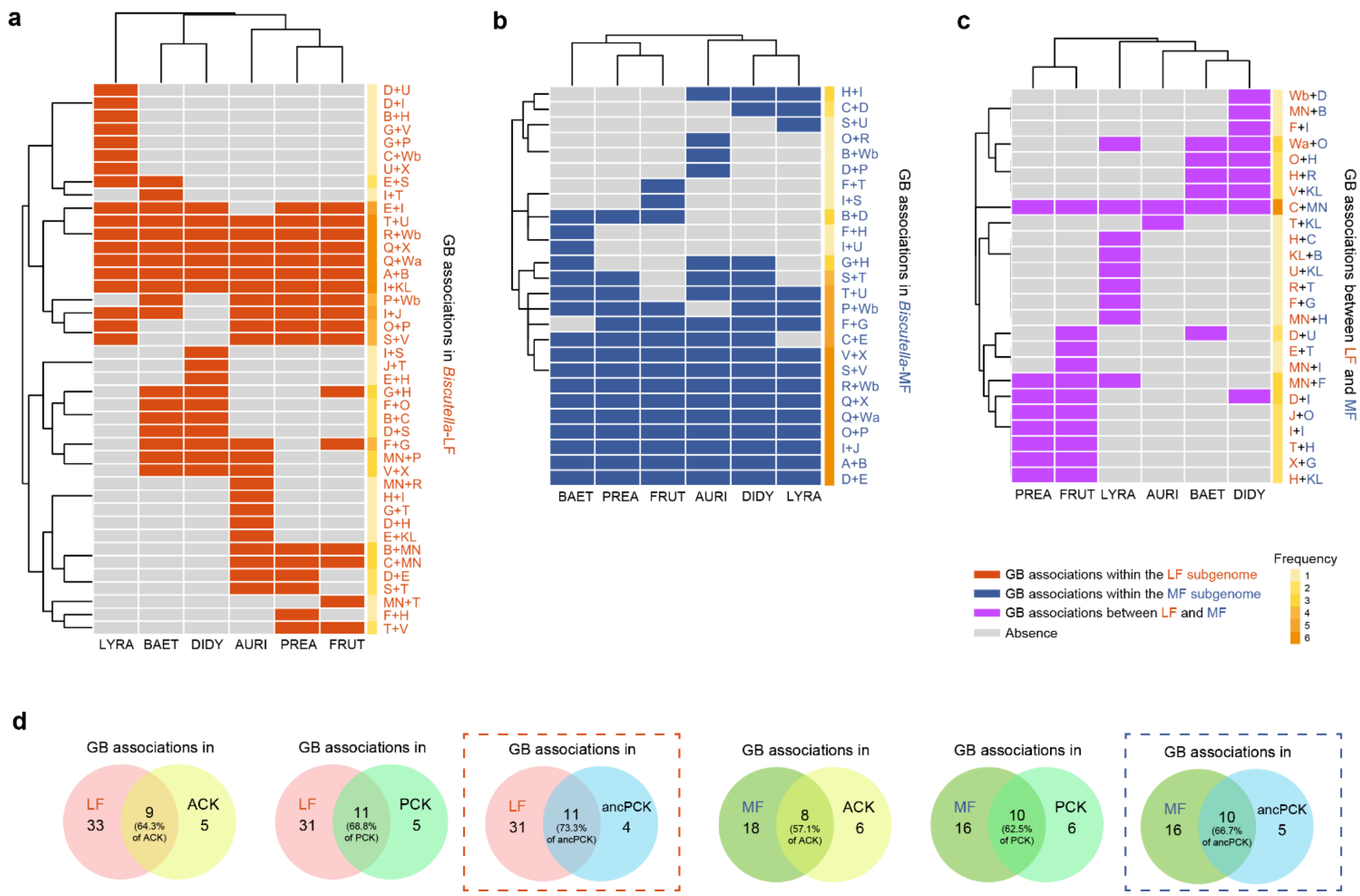

Supplementary Fig. 13 | Analysis of GB associations in *Biscutella* karyotypes. (**a-c**) Presence and absence of pairwise neighboring GB associations within the LF (**a**) and MF (**b**) subgenomes, as well as between LF and MF subgenomes (**c**). The present GB associations are marked in orange (within LF), blue (within MF), and purple (between LF and MF). The missing GB associations are marked in grey. The pheatmap R package (v1.0.12)[5] was used for hierarchical clustering. (**d**) Shared and unique GB associations in LF and MF subgenomes compared to the three known ancestral Brassicaceae karyotypes, including Ancestral Crucifer Karyotype (ACK)[4,6], ancestral Proto-Calepineae Karyotype (ancPCK)[7] and Proto-Calepineae Karyotype (PCK)[8]. The ancPCK karyotype best explains the GB associations observed in *Biscutella*, as it contains a higher proportion of GB associations shared with LF (orange dotted box) and MF (blue dotted box) compared to the ACK and PCK ancestral karyotypes.

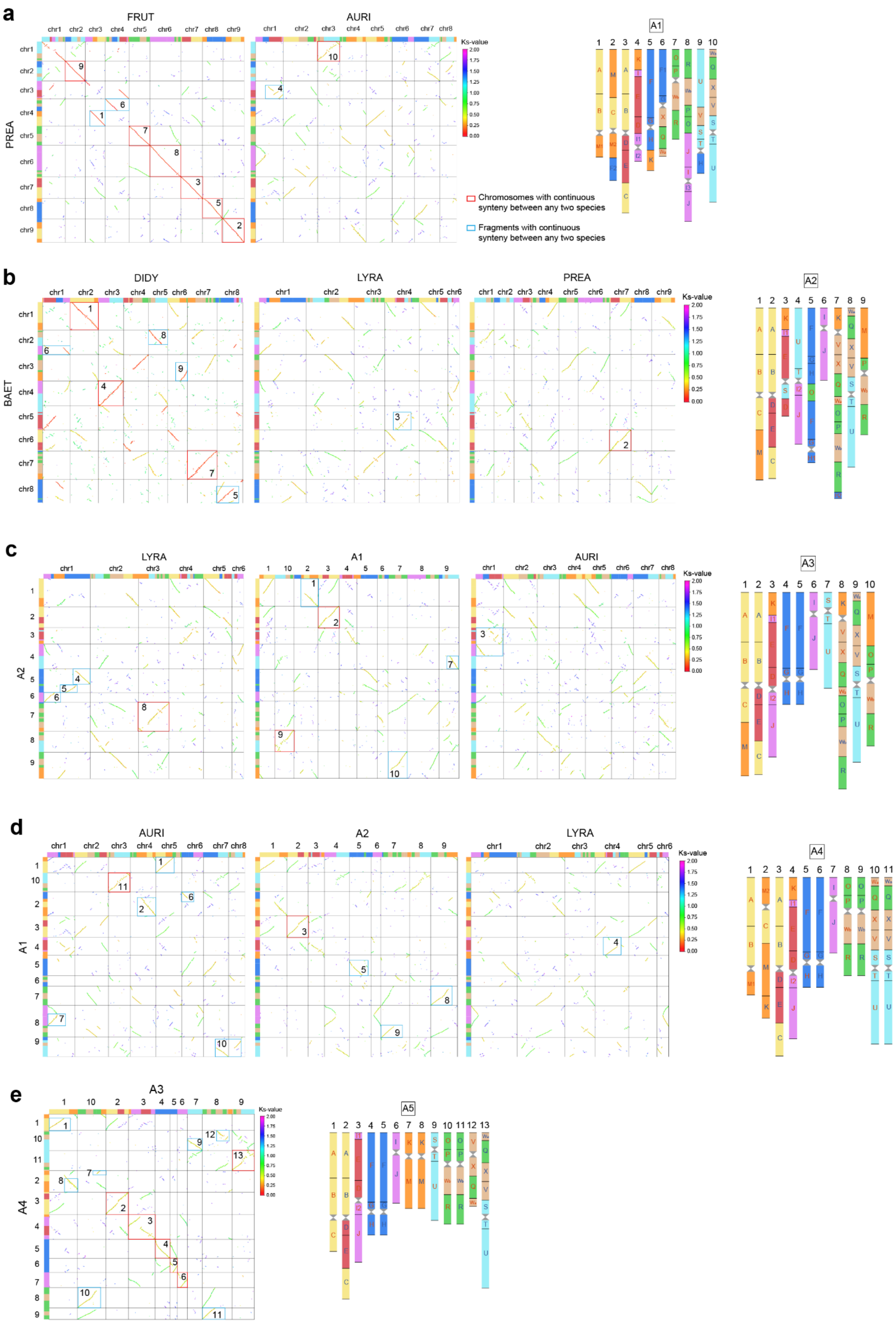

a
FRUT
AURI
PREA
Ks-value
Chromosomes with continuous synteny between any two species
Fragments with continuous synteny between any two species
A1
b
DIDY
LYRA
PREA
BAET
A2
c
LYRA
A1
AURI
A2
A3
d
AURI
A2
LYRA
A1
A4
e
A3
A4
A5

Supplementary Fig. 14 | Bottom-up strategy for inferring ancestral karyotypes at each phylogenetic node (A1 – A5, from the youngest to the oldest). (**a**) Inference of A1, the common ancestor of *n* = 9 species. Based on synteny maps comparing *B. prealpina* (Y-axis) with *B. frutescens* and *B. auriculata* (X-axis), intact chromosomes that maintain collinear relationships between any two species are assumed to have originated from A1 (red box; chromosomes 2, 3, 5, 7, 8, 9, and 10 in A1). The A+B+MN, KL+I+E+D, and F+G+X+Q+Wa block associations are observed in any two species among *B. prealpina*, *B. frutescens*, and *B. auriculata*, suggesting that these associations also originated from A1 (blue box; corresponding to chromosomes 1, 4, and 6 in A1). Therefore, we inferred that A1 had 10 chromosome pairs. (**b**) Inference of A2, the common ancestor of *B. baetica*-*B.didyma* clade. Based on synteny maps comparing *B. baetica* (Y-axis) with *B. didyma*, *B. lyrata*, and *B. prealpina* (X-axis), intact chromosomes that maintain collinear relationships between any two species are assumed to have originated from A2 (red box; chromosomes 1, 2, 4, and 7 in A2). The S+D, F+G+H+O+F+G+H, I+J, Wa+Q+X+V+S+T+U, and MN+P associations are observed in any two species among *B. baetica*, *B.didyma*, and *B. lyrata* (blue box; corresponding to chromosomes 3, 5, 6, 8, and 9 in A2). Therefore, we inferred that A2 had 9 chromosome pairs. (**c**) Inference of A3, the common ancestor of clade II in Fig. 3b. Based on synteny maps comparing A2 (Y-axis) with *B. lyrata*, A1, and *B. auriculata* (X-axis), intact chromosomes that maintain collinear relationships between any two species are assumed to have originated from A3 (red box; chromosomes 2, 8, and 9 in A3). The A+B+C+MN, I1+E+D+I2+J (I1, I2, ..., Ix referring to the broken I block, other GBs with the same abbreviation), F+G+H (in both LF and MF), I+J, S+T+U, and O+P+Wb+R associations are observed in any two species among A2, *B. lyrata*, A1, and *B. auriculata* (blue box; corresponding to chromosomes 1, 3, 4, 5, 6, 7, and 10 in A2). Therefore, we inferred that A3 had 10 chromosome pairs. (**d**) Inference of A4, the common ancestor of clade I in Fig. 3b. Based on synteny maps comparing A1 (Y-axis) with *B. auriculata*, A2, and *B. lyrata* (X-axis), intact chromosomes that maintain collinear relationships between any two species are assumed to have originated from A4 (red box; chromosomes 3 and 11 in A4). The B+MN1, C+MN2, D+I+J, F+G+H (in both LF and MF), I+J, O+P+Wb+R (in both LF and MF), and Wa+Q+X+V+S+T+U associations are observed in any two species among A1, *B. auriculata*, A2, and *B. lyrata* (blue box; corresponding to chromosomes 1, 2, 4, 5, 6, 7, 8, 9, and 10 in A2). Therefore, we inferred that A4 had 11 chromosome pairs. (**e**) Inference of A5. Based on synteny maps comparing A3 (Y-axis) with A4 (X-axis), intact chromosomes that maintain collinear relationships are assumed to have originated from A5 (red box; chromosomes 2, 3, 4, 5, 6, and 13 in A5). The A+B+C, KL+MN (in both LF and MF), S+T+U, O+P+Wb+R (in both LF and MF), and V+X+Q+Wa associations are inferred as seven independent chromosomes. Therefore, we inferred that A5 had 13 chromosome pairs. The GB association I1+E+D+I2+J in chromosome 3 of A5 suggests an insertion event (E+D inserted

in the middle of the I block), but this event was not observed in *Heldreichia*, a species closely related to the LF subgenome, and thus this insertion should be specific to *Biscutella* and originate at an early stage of *Biscutella* speciation.

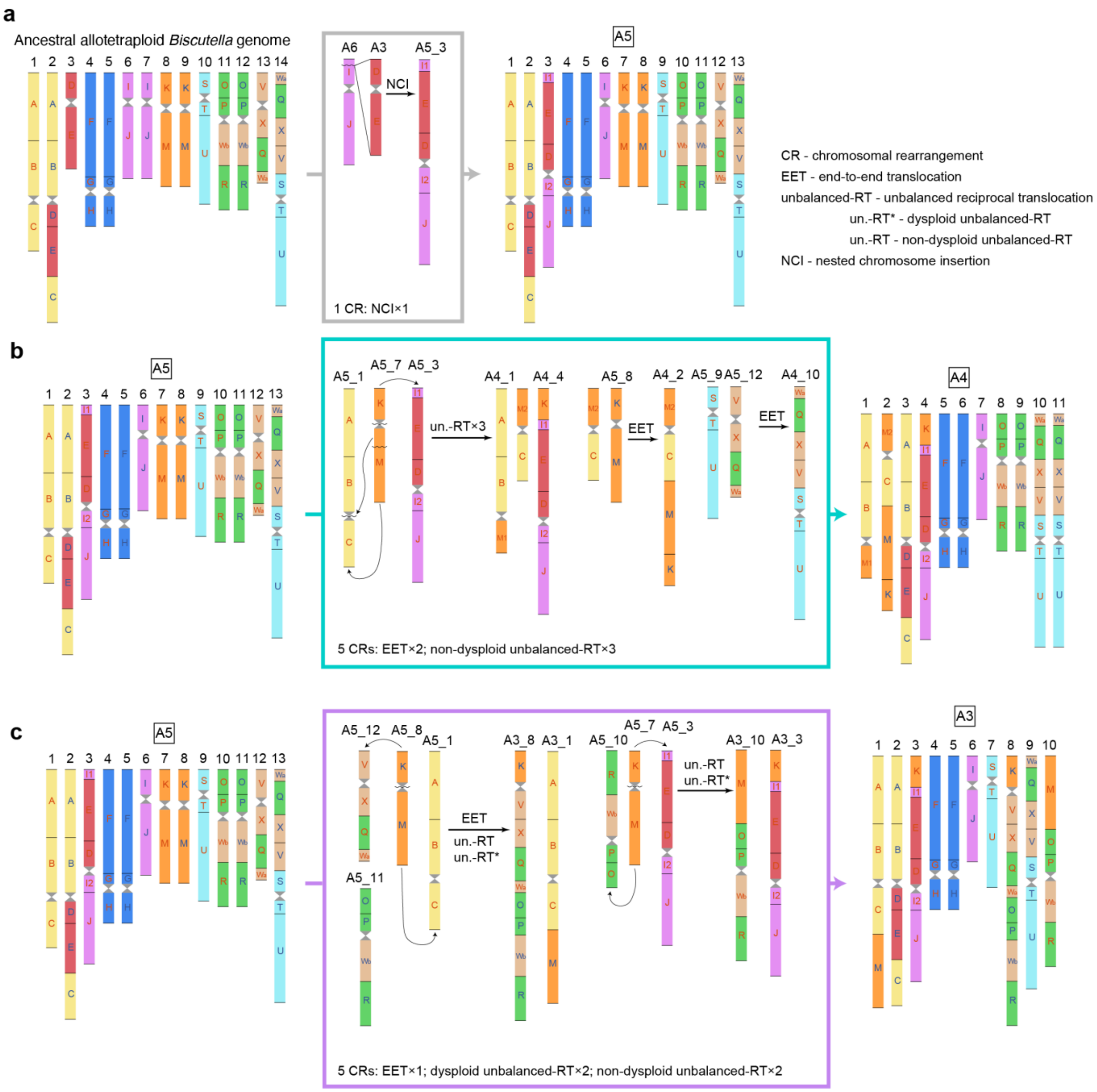
a
Ancestral allotetraploid *Biscutella* genome
A6 A3 A5_3
NCI
1 CR: NCI×1
A5
CR - chromosomal rearrangement
EET - end-to-end translocation
unbalanced-RT - unbalanced reciprocal translocation
un.-RT* - dysploid unbalanced-RT
un.-RT - non-dysploid unbalanced-RT
NCI - nested chromosome insertion
b
A5
A5_1 A5_7 A5_3
un.-RT×3
A4_1 A4_4
A5_8
EET
A4_2 A5_9 A5_12
EET
A4_10
5 CRs: EET×2; non-dysploid unbalanced-RT×3
A4
c
A5
A5_12 A5_8 A5_1
A5_11
EET
un.-RT
un.-RT*
A3_8 A3_1
A5_10 A5_7 A5_3
un.-RT
un.-RT*
A3_10 A3_3
5 CRs: EET×1; dysploid unbalanced-RT×2; non-dysploid unbalanced-RT×2
A3

Supplementary Fig. 15 | Evolutionary pathways from ancestral allotetraploid *Biscutella* genome (n = 14) to A4 and A3 nodes. (**a**) Evolutionary pathway from ancestral *Biscutella* genome to A5 ($n = 14 \rightarrow n = 13$). The ancestral chromosomes 3 (GB D+E; LF) and 6 (I+J; LF) underwent a nested chromosome insertion (NCI) event to form the chromosome 3 (I1+E+D+I2+J; I1, I2, ..., Ix referring to the broken I GB, other GBs with the same abbreviation) of A5. (**b**) Evolutionary pathway from A5 to A4 ($n = 13 \rightarrow n = 11$). The chromosomes 1 (A+B+MN1; LF) and 2 (MN2+C+MN+KL; LF+MF), 4 (KL+I1+E+D+I2+J; LF) in A4 originated from chromosomes 1 (A+B+C; LF), 3 (I1+E+D+I2+J; LF), 7 (KL+MN; LF), and 8 (KL+MN; MF) of A5 genome after three unbalanced (un.) reciprocal translocations (unbalanced-RTs) and one end-to-end translocation (EET). The chromosome 10 (Wa+Q+X+V+S+T+U; LF) originated from chromosomes 9 (S+T+U; LF) and 12 (V+X+Q+Wa; LF) of A5 genome after an EET event. Therefore, the A5 genome underwent five CRs to form A4. (**c**) Evolutionary pathway from A5 to A3 ($n = 13 \rightarrow n = 10$). The chromosomes 1 (A+B+C+MN; LF+MF) and 8 (KL+V+X+Q+Wa+O+P+Wb+R; LF+MF) in A3 originated from chromosomes 1 (A+B+C; LF), 8 (KL+MN; MF), 11 (O+P+Wb+R; MF), and 12 (V+X+Q+Wa; LF) of A5 genome after two unbalanced-RTs and one EET. The chromosomes 3 (KL+I1+E+D+I2+J; LF) and 10 (MN+O+P+Wb+R; LF) in A3 originated from chromosomes 3 (I1+E+D+I2+J; LF), 7 (KL+MF; LF), and 10 (R+Wb+P+O; LF) after two unbalanced-RTs. Therefore, the A5 genome also underwent 5 CRs to form A3, while being distinct from the A5 → A4 pathway in (**b**).

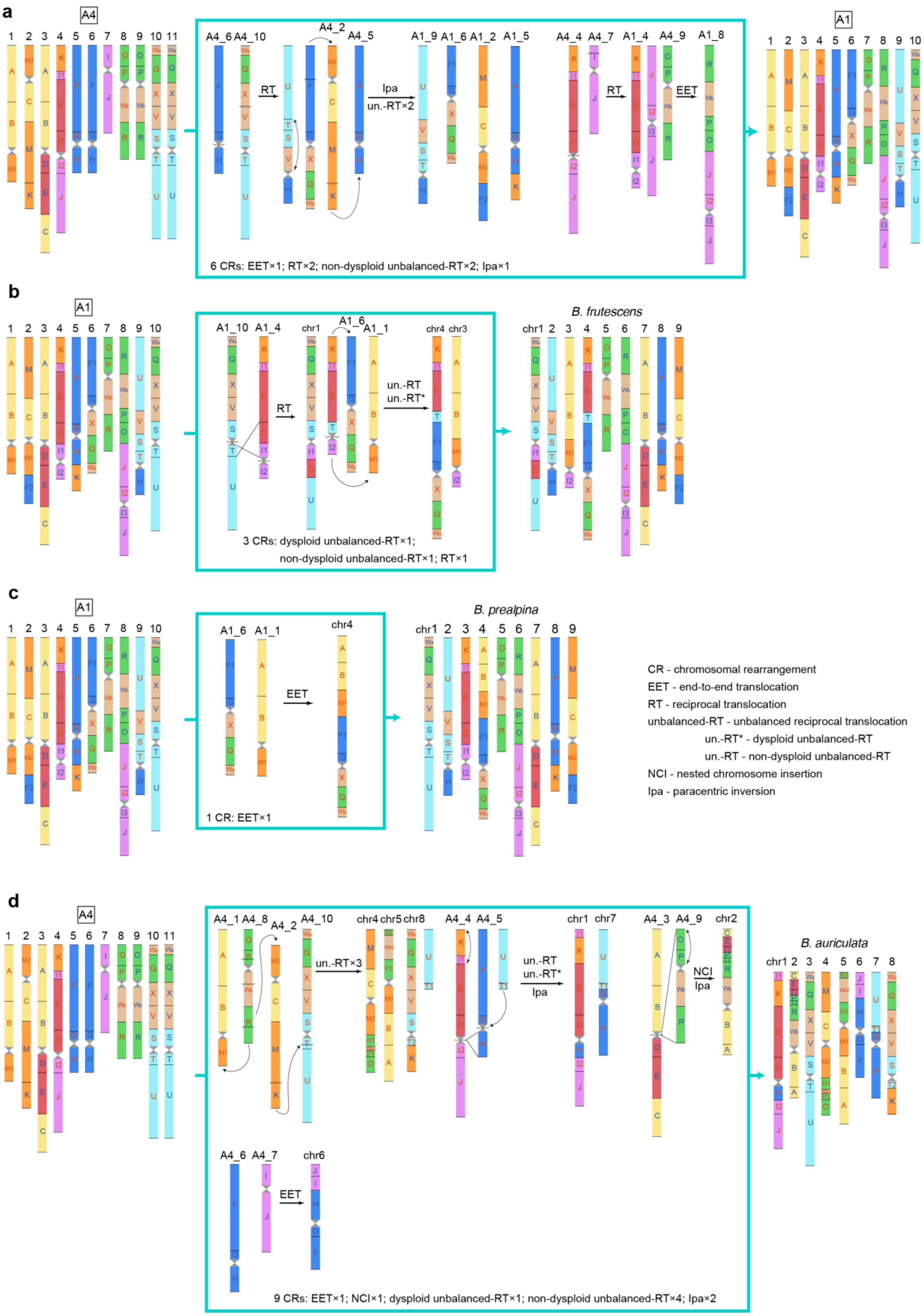
a
A4
A1
6 CRs: EET×1; RT×2; non-dysploid unbalanced-RT×2; Ipa×1
b
A1
B. frutescens
3 CRs: dysploid unbalanced-RT×1;
non-dysploid unbalanced-RT×1; RT×1
c
A1
B. prealpina
1 CR: EET×1
CR - chromosomal rearrangement
EET - end-to-end translocation
RT - reciprocal translocation
unbalanced-RT - unbalanced reciprocal translocation
un.-RT* - dysploid unbalanced-RT
un.-RT - non-dysploid unbalanced-RT
NCI - nested chromosome insertion
Ipa - paracentric inversion
d
A4
B. auriculata
9 CRs: EET×1; NCI×1; dysploid unbalanced-RT×1; non-dysploid unbalanced-RT×4; Ipa×2

Supplementary Fig. 16 | Evolutionary pathways from A4 (*n* = 11) to *n* = 9 species and *B. auriculata*. (**a**) Evolutionary pathway from A4 to A1 (*n* = 11 → *n* = 10). The chromosomes 2 (MN+C+MN2+F2; LF+MF), 5 (F+G+H+KL; LF+MF), 6 (F1+G+X+Q+Wa; LF+MF) and 9 (U+V+S+T+H; LF+MF) in A1 originated from chromosomes 2 (MN2+C+MN+KL; LF+MF), 5 (F+G+H; LF), 6 (F+G+H; MF), and 10 (Wa+Q+X+V+S+T+U; LF) of A4 genome after two unbalanced-RTs, one reciprocal translocation (RT) and one paracentric inversion (Ipa). The chromosomes 4 (KL+I+E+D+I1+I2; LF+MF) and 8 (R+Wb+P+O+J+I2+J3+J; LF+MN) originated from chromosomes 4 (KL+I+E+D+I1+I2+J; LF), 7 (I+J; MF) and 9 (O+P+Wb+R; MF) of A4 genome after an EET and RT event. Therefore, the A4 genome underwent six CRs to form A1. (**b**) Evolutionary pathway from A1 to *B. frutescens* (*n* = 10 → *n* = 9). The chromosomes 1 (Wa+Q+X+V+S+I1+D+U; LF+MF), 3 (A+B+MN1+I2; LF+MF), and 4 (KL+I1+E+T+F1+G+X+Q+Wa; LF+MF) in *B. frutescens* originated from chromosomes 1 (A+B+MN1; LF), 4 (KL+I1+E+D+I1+I2; LF+MF), 6 (F1+G+X+Q+Wa; LF+MF), and 10 (Wa+Q+X+V+S+T+U; MF) of A1 genome after two unbalanced-RTs and one RT event. Therefore, the A1 genome underwent three CRs to form *B. frutescens*. (**c**) Evolutionary pathway from A1 to *B. prealpina* (*n* = 10 → *n* = 9). The chromosome 4 (A+B+MN1+F1+G+X+G+Wa; LF+MF) in *B. prealpina* originated from chromosomes 1 (A+B+MN1; LF) and 6 (F1+G+X+Q+Wa; LF+MF) of A1 genome after an EET event. Therefore, the A1 genome underwent one CR to form *B. prealpina*. (**d**) Evolutionary pathway from A4 to *B. auriculata* (*n* = 11 → *n* = 8). The chromosomes 1 (Wa+Q+X+V+S+I1+D+U; LF+MF), 4 (MN+C+MN2+R1+Wb1+P1+O; LF+MF), 5 (R2+Wb2+P2+MN1+B+A; LF), 7 (U+T1+G+F; LF), and 8 (Wa+Q+X+V+S+T2+KL; LF+MF) in *B. auriculata* originated from chromosomes 1 (A+B+MN1; LF), 2 (MN2+C+MN+KL; LF+MF), 4 (KL+I1+E+D+I2+J; LF), 5 (F+G+H; LF), 8 (O+P+Wb+R; LF), and 10 (Wa+Q+X+V+S+T+U; LF) of A4 genome after five unbalanced-RTs and one Ipa event. The chromosome 2 (C+E+D+P+O+R+Wb+B+A; MF) in *B. auriculata* originated from chromosomes 3 (A+B+D+E+C; MF) and 9 (O+P+Wb+R) of A4 genome after an NCI and Ipa event. The chromosome 6 (J+I+H+G+F; MF) in *B. auriculata* originated from chromosomes 6 (F+G+H; MF) and 7 (I+J; MF) of A4 genome after an EET event. Therefore, the A4 genome underwent nine CRs to form *B. auriculata*.

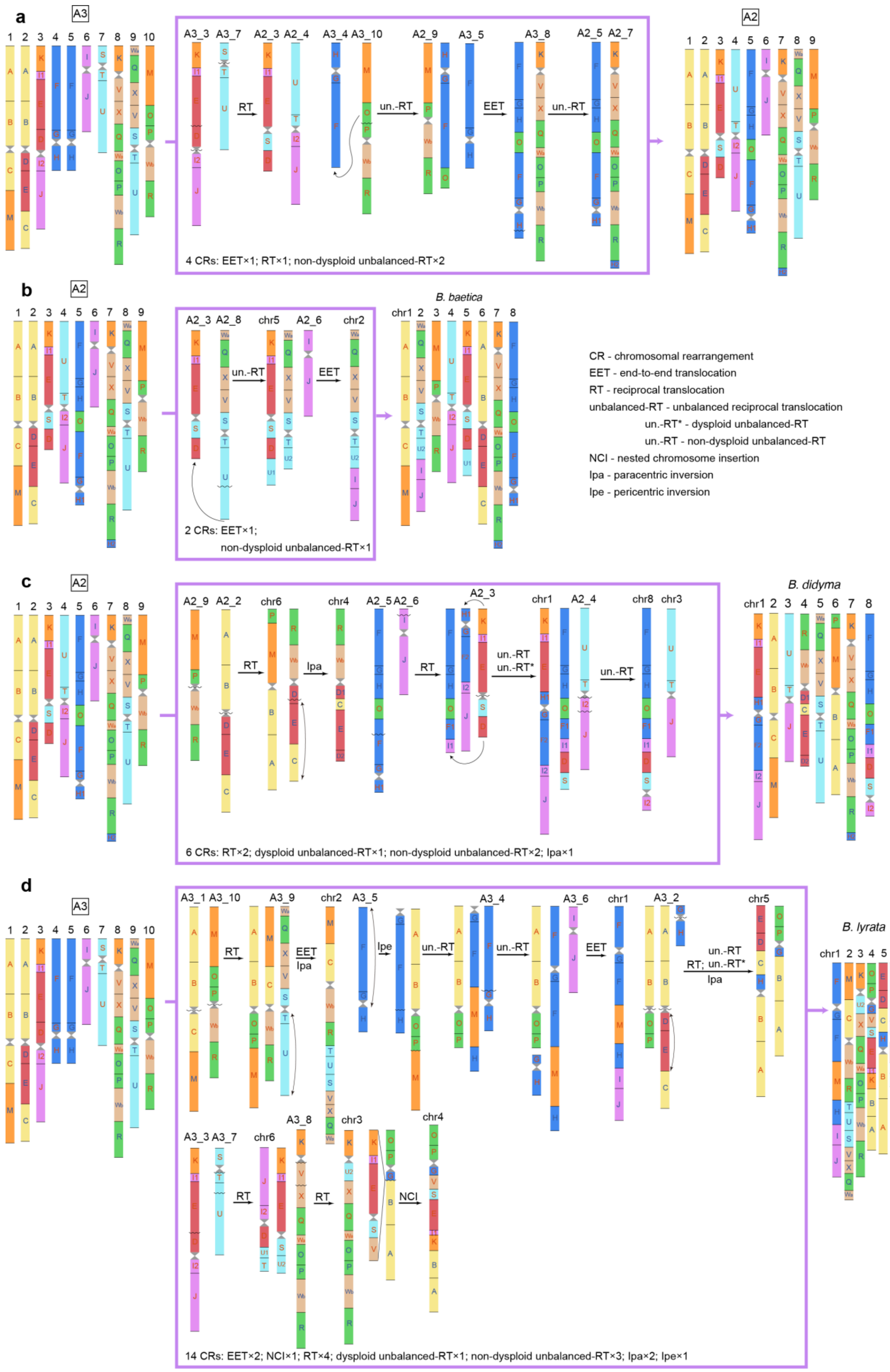
a
A3
A2
RT
un.-RT
EET
4 CRs: EET×1; RT×1; non-dysploid unbalanced-RT×2
b
B. baetica
2 CRs: EET×1;
non-dysploid unbalanced-RT×1
CR - chromosomal rearrangement
EET - end-to-end translocation
RT - reciprocal translocation
unbalanced-RT - unbalanced reciprocal translocation
un.-RT* - dysploid unbalanced-RT
un.-RT - non-dysploid unbalanced-RT
NCI - nested chromosome insertion
Ipa - paracentric inversion
Ipe - pericentric inversion
c
B. didyma
Ipa
un.-RT*
6 CRs: RT×2; dysploid unbalanced-RT×1; non-dysploid unbalanced-RT×2; Ipa×1
d
B. lyrata
Ipe
NCI
RT; un.-RT*
14 CRs: EET×2; NCI×1; RT×4; dysploid unbalanced-RT×1; non-dysploid unbalanced-RT×3; Ipa×2; Ipe×1

Supplementary Fig. 17 | Evolutionary pathways from A3 (n = 10) to *B. baetica*, *B. didyma*, and *B. lyrata*. (**a**) Evolutionary pathway from A3 to A2 ($n = 10 \rightarrow n = 9$). The chromosomes 3 (KL+I1+E+S+D; LF) and 4 (U+T+I2+J; LF) in A2 originated from chromosomes 3 (KL+I1+E+D+I2+J; LF) and 7 (S+T+U; LF) of A3 genome after an RT event. The chromosomes 5 (F+G+H+O+F+G+H1; LF+MF), 7 (KL+V+X+Q+Wa+O+P+Wb+R+H2; LF+MF), and 9 (MN+P+Wb+R; LF) originated from chromosomes 4 (F+G+H; LF), 5 (F+G+H; MF), 8 (KL+V+X+Q+Wa+O+P+Wb+R; LF+MF), and 10 (MN+O+P+Wb+R; LF) of A3 genome after two unbalanced-RTs and an EET event. Therefore, the A3 genome underwent four CRs to form A2. (**b**) Evolutionary pathway from A2 to *B. baetica* ($n = 9 \rightarrow n = 8$). The chromosomes 2 (Wa+Q+X+V+S+T+U2+I+J; MF) and 5 (KL+I1+E+S+D+U1; LF+MF) in *B. baetica* originated from chromosomes 3 (KL+I1+E+S+D; LF), 6 (I+J; MF), and 8 (Wa+Q+X+V+S+T+U; MF) of A2 genome after a unbalanced-RT and an EET event. Therefore, the A2 genome underwent two CRs to form *B. baetica*. (**c**) Evolutionary pathway from A2 to *B. didyma* ($n = 9 \rightarrow n = 8$). The chromosomes 4 (R+Wb+D1+C+E+D2; LF+MF) and 6 (P+MN+A+B; LF+MF) in *B. didyma* originated from chromosomes 2 (A+B+D+E+C; MF) and 9 (MN+P+Wb+R; LF) of A2 genome after an RT and Ipa event. The chromosomes 1 (KL+I1+E+H1+G+F2+I2+J; LF+MF), 3 (U+T+J; LF), and 8 (F+G+H+O+F1+I1+D+S+I2; LF+MF) in *B. didyma* originated from chromosomes 3 (KL+I1+E+S+D; LF), 4 (U+T+I2+J; LF), 5 (F+G+H+O+F+G+H; LF+MF), 6 (I+J; MF) of A2 genome after three unbalanced-RTs and an RT event. Therefore, the A2 genome underwent six CRs to form *B. didyma*. (**d**) Evolutionary pathway from A3 to *B. lyrata* ($n = 10 \rightarrow n = 6$). The chromosomes 1 (F+G+F+MN+H+I+J; LF+MF) and 2 (MN+C+Wb+R+T+U+S+V+X+Q+Wa; LF+MF) in *B. lyrata* originated from chromosomes 1 (A+B+C+MN; LF+MF), 4 (F+G+H; LF), 5 (F+G+H; MF), 9 (Wa+Q+X+V+S+T+U; MF), and 10 (MN+O+P+Wb+R; LF) after two unbalanced-RTs, two EETs, one Ipa, one Ipe, and an RT event. The two intermediate fragments (A+B+O+P; LF, and G+H; LF) produced in this process underwent two unbalanced-RTs, one RT, and one Ipa with chromosome 2 (A+B+D+E+C; MF) in A3 to form chromosome 5 (E+D+C+H+B+A; LF+MF) in *B. lyrata* and another fragment (O+P+G+B+A; LF+MF). The chromosomes 3 (KL+U2+X+Q+Wa+O+P+Wb+R; LF+MF) and 6 (J+I2+D+U1+T; LF) in *B. lyrata* originated from chromosomes 3 (KL+I1+E+D+I2+J; LF), 7 (S+T+U; LF), and 8 (KL+V+X+Q+Wa+O+P+Wb+R; LF+MF) after two RT events. Two intermediate fragments (KL+I1+E+S+V; LF, and O+P+G+B+A; LF+MF) then underwent an NCI event to form chromosome 4 (O+P+G+V+S+E+I1+KL+B+A; LF+MF) in *B. lyrata*. Therefore, the A3 genome underwent 14 CRs to form *B. lyrata*.

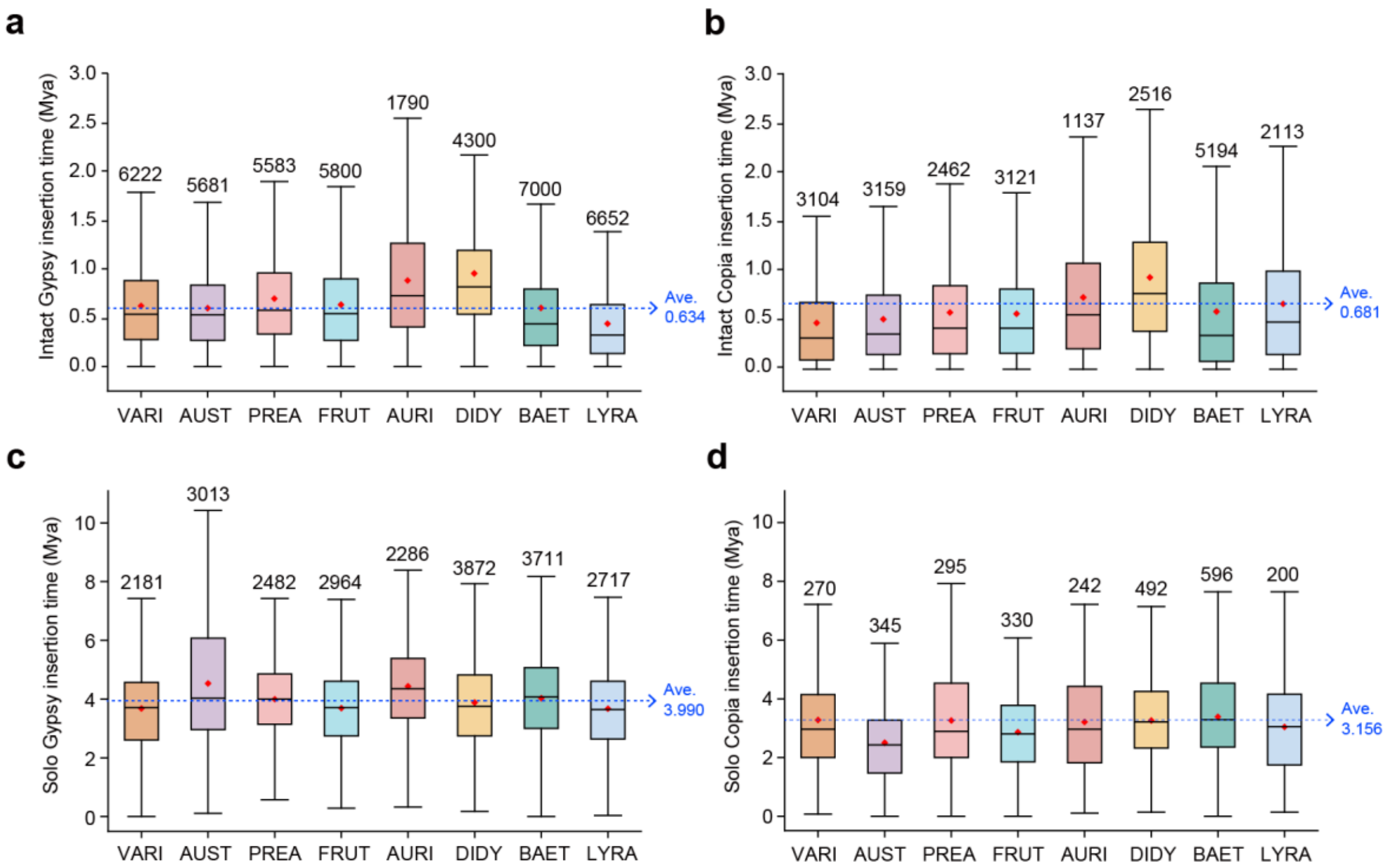

Supplementary Fig. 18 | Insertion time distribution of Gypsy- and Copia-type retrotransposons. (**a**, **b**) Insertion time of intact Gypsy (**a**) and Copia (**b**) elements, estimated based on the sequence divergence between their two LTRs. (**c**, **d**) Insertion time of solo Gypsy (**c**) and Copia (**d**) elements, inferred from sequence divergence between each soloLTR and the consensus LTR sequence of the corresponding family. Box plot shows the median (centre black line), mean (red square), interquartile range (box limits), and whiskers extending to 1.5× the interquartile range. The sample size (n) is indicated above each box. The average (ave.) value of element insertion time across the eight genomes is marked with blue dashed lines.

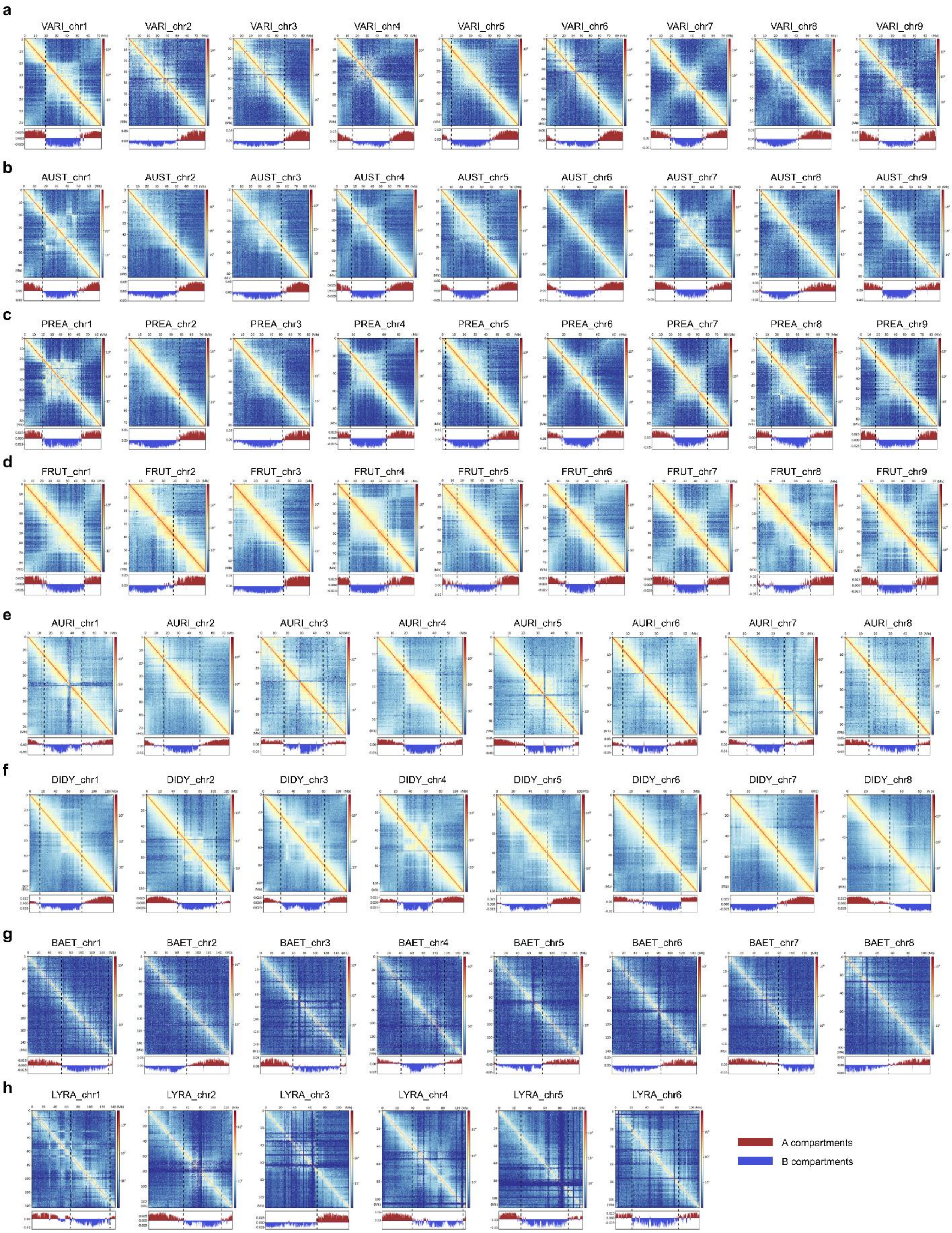
a
VARI_chr1
VARI_chr2
VARI_chr3
VARI_chr4
VARI_chr5
VARI_chr6
VARI_chr7
VARI_chr8
VARI_chr9
b
AUST_chr1
AUST_chr2
AUST_chr3
AUST_chr4
AUST_chr5
AUST_chr6
AUST_chr7
AUST_chr8
AUST_chr9
c
PREA_chr1
PREA_chr2
PREA_chr3
PREA_chr4
PREA_chr5
PREA_chr6
PREA_chr7
PREA_chr8
PREA_chr9
d
FRUT_chr1
FRUT_chr2
FRUT_chr3
FRUT_chr4
FRUT_chr5
FRUT_chr6
FRUT_chr7
FRUT_chr8
FRUT_chr9
e
AURI_chr1
AURI_chr2
AURI_chr3
AURI_chr4
AURI_chr5
AURI_chr6
AURI_chr7
AURI_chr8
f
DIDY_chr1
DIDY_chr2
DIDY_chr3
DIDY_chr4
DIDY_chr5
DIDY_chr6
DIDY_chr7
DIDY_chr8
g
BAET_chr1
BAET_chr2
BAET_chr3
BAET_chr4
BAET_chr5
BAET_chr6
BAET_chr7
BAET_chr8
h
LYRA_chr1
LYRA_chr2
LYRA_chr3
LYRA_chr4
LYRA_chr5
LYRA_chr6
A compartments
B compartments

Supplementary Fig. 19 | Hi-C contact maps and PC1 value tracks of chromosomes in *B. varia* (**a**), *B. austriaca* (**b**), *B. prealpina* (**c**), *B. frutescens* (**d**), *B. auriculata* (**e**), *B. didyma* (**f**), *B. baetica* (**g**), and *B. lyrata* (**h**). The red and blue bars of PC1 value tracks represent the A/B compartments, respectively. The dashed lines show the main boundaries of the A/B compartments.

ID	Block changes	Ancestral or recurrent	Presence or absence	Synteny maps
BP_5	F (MF) ↓ F1 and F2	Ancestral to A1	A1; Pr. Fr.	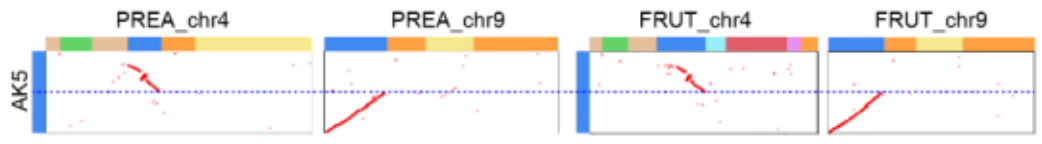
BP_6	G+H (MF) ↓ G and H	Ancestral to A1	A1; Pr. Fr.	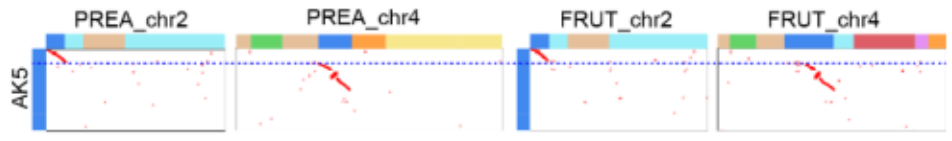
BP_8	I (MF) ↓ I1 and I2	Ancestral to A1	A1; Pr. Fr.	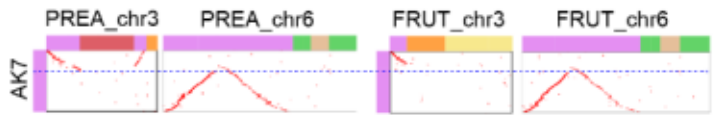
BP_14	V+X (LF) ↓ V and X	Ancestral to A1	A1; Pr. Fr.	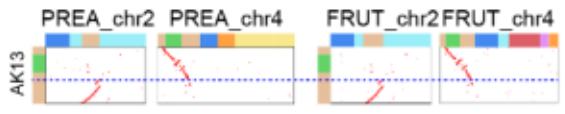
BP_4	H (LF) ↓ H1 and H2	Ancestral to A2	A2; Di. Ba.	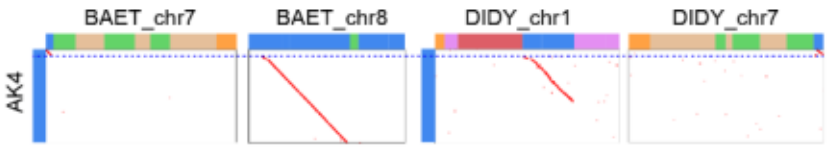
BP_13	O+P (LF) ↓ O and P	Ancestral to A2	A2; Di. Ba.	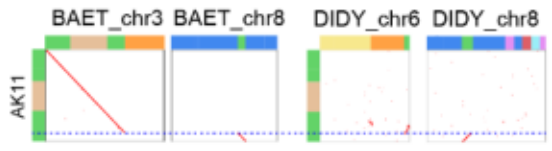
BP_10	MN (LF) ↓ MN1 and MN2	Ancestral to A4	A4, A1; Au. Pr. Fr.	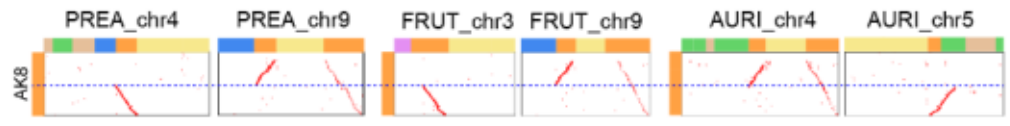
BP_7	I (LF) ↓ I1 and I2	Ancestral to A5	A5, A4, A3, A1, A2; Au. Pr. Fr. Di. Ba. Ly.	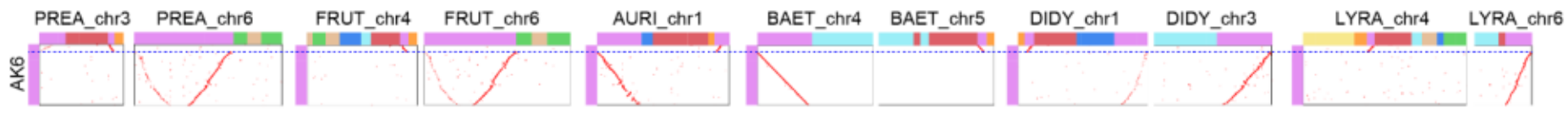
BP_11	KL+MN (MF) ↓ KL and MN	Recurrent in A1, A3 and AURI	A3, A1, A2; Au. Pr. Fr. Di. Ba. Ly.	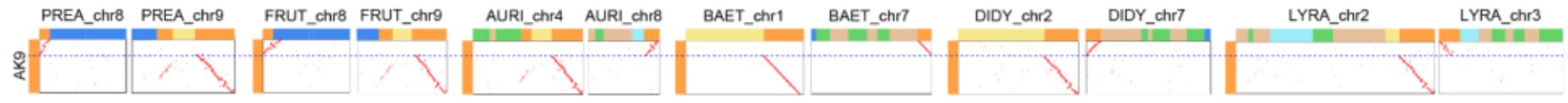
BP_12	S+T (LF) ↓ S and T	Recurrent in A2 and LYRA	A2; Di. Ba. Ly.	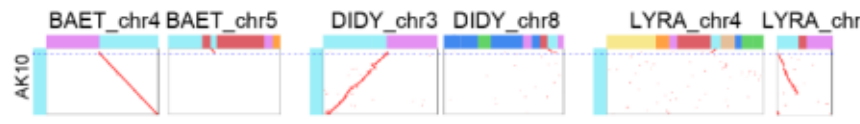
BP_3	D+E (LF) ↓ D and E	Recurrent in A2, FRUT and LYRA	A2; Fr. Di. Ba. Ly.	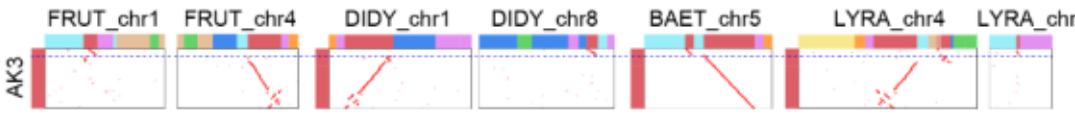
BP_9	KL+MN (LF) ↓ KL and MN	Recurrent in A3 and A4	A4, A3, A1, A2; Au. Pr. Fr. Di. Ba. Ly.	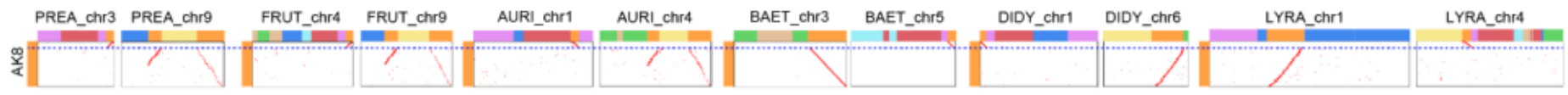
BP_1	B+C (LF) ↓ B and C	Recurrent in A4 and LYRA	A4, A1; Au. Pr. Fr. Ly.	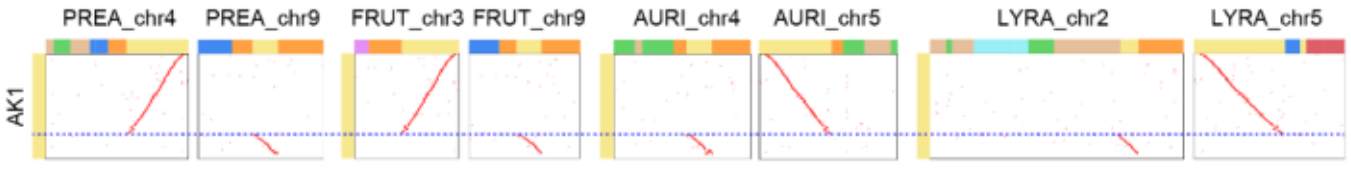
BP_2	B+D (MF) ↓ B and D	Recurrent in DIDY, LYRA and AURI	Au. Di. Ly.	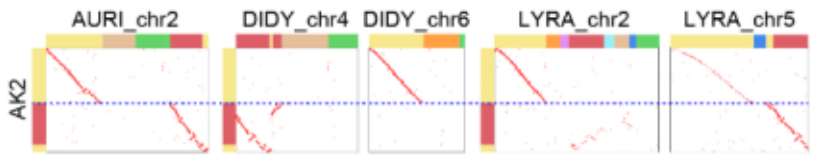

Supplementary Fig. 20 | Location details of fourteen shared chromosome breakpoints. The first column indicates the breakpoint id (BP_1 to BP_14). The second column specifies the GB associations that are altered due to the corresponding breakpoints. The third and fourth columns indicate whether each breakpoint is classified as ancestral (gradient pink) or recurrent (gradient green), based on its presence or absence across different species and phylogenetic nodes. A breakpoint is considered ancestral if it appears within a single clade and is present in the inferred ancestral karyotype of that clade. In contrast, breakpoints found in multiple different clades are classified as recurrent. The fifth column displays synteny comparisons between the reconstructed ancestral genome and the six *Biscutella* genomes. Dashed lines indicate breakpoints that are shared among species, occurring at the same genomic positions.

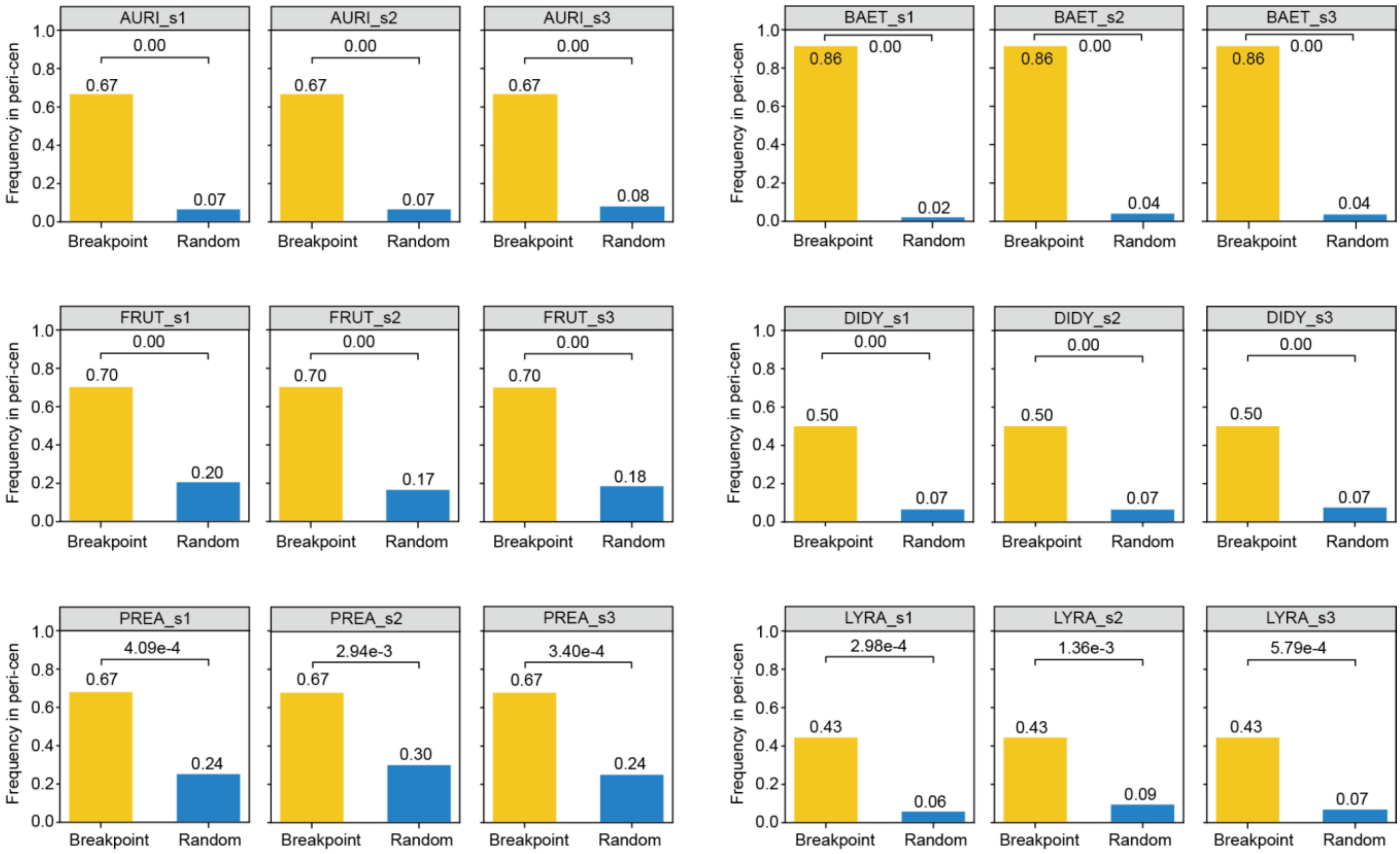

Supplementary Fig. 21 | Frequency of overlap between (peri)centromeres and two sets of genomic regions: breakpoint regions (yellow bar, abbreviated as Breakpoint) and 200 simulated regions randomly sampled from the whole genome (blue bar, abbreviated as Random). Random regions were generated independently three times for each genome (s1–s3), with each set matching the size distribution of breakpoint regions. For each genome and each simulation replicate, statistical significance of the difference in overlap was assessed using two-sided Fisher's exact test. To evaluate the overall trend across genomes and simulations, the overlap frequencies of breakpoint and corresponding random regions were treated as paired observations and compared using a two-sided Wilcoxon signed-rank test. Across all genomes and replicates, breakpoint regions showed a significantly higher frequency of overlap with (peri)centromeres than random regions (*P*-value = 1.94×10^{-4}; two-sided Wilcoxon signed-rank test).

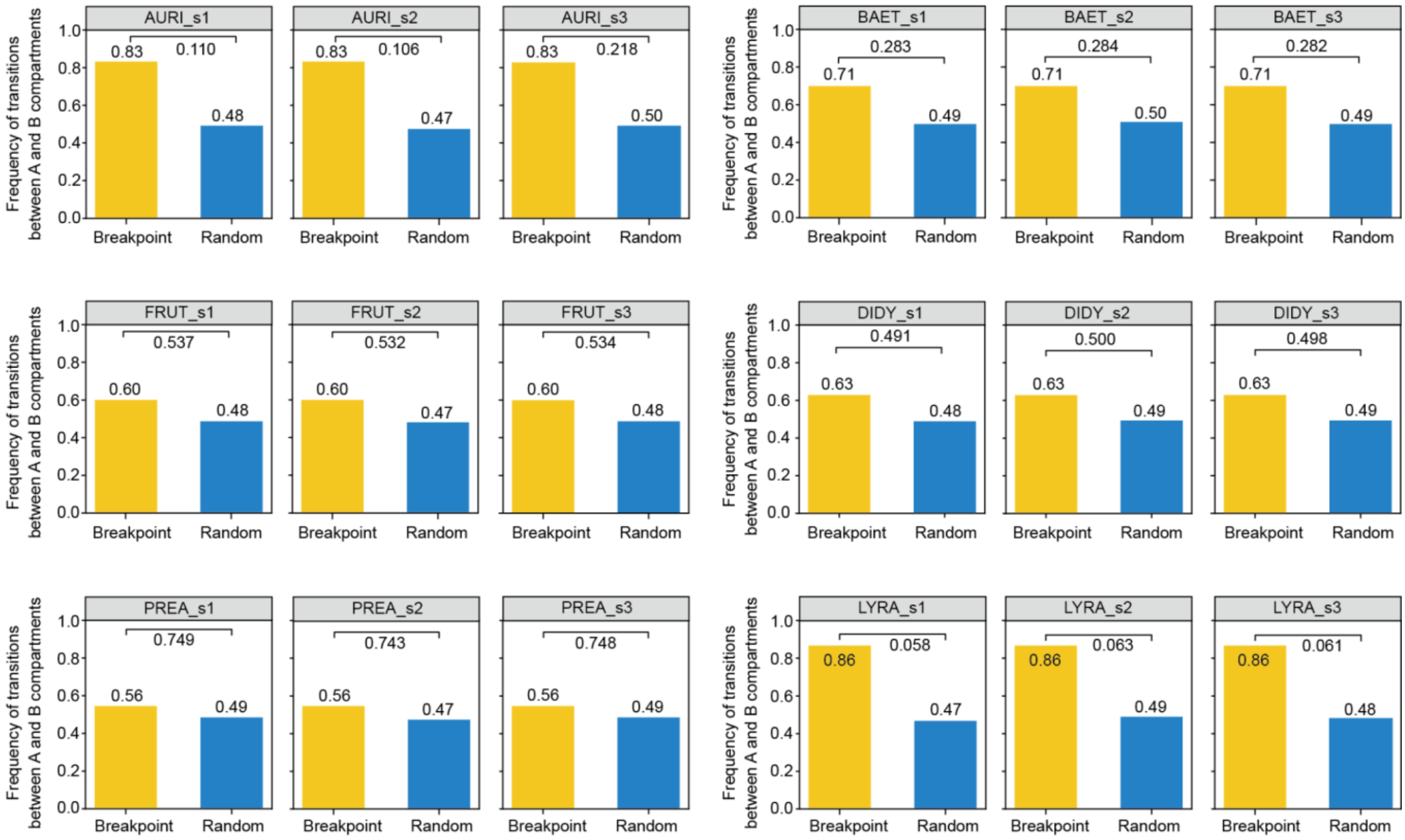

Supplementary Fig. 22 | Frequency of transitions between A and B compartments in the breakpoint regions (yellow bar, abbreviated as Breakpoint) compared to that of 200 simulated random genomic regions (blue bar, abbreviated as Random). Random regions were generated independently three times for each genome (s1–s3), with each set matching the size distribution of breakpoint regions. For each genome and each simulation replicate, statistical significance of the difference in transitions was assessed using two-sided Fisher's exact test. To evaluate the overall trend across genomes and simulations, the transition frequencies of breakpoint and corresponding random regions were treated as paired observations and compared using a two-sided Wilcoxon signed-rank test. Across all genomes and replicates, breakpoint regions showed a significantly higher frequency of transitions between A and B compartments than random regions (*P*-value = 1.95×10^{-4}; two-sided Wilcoxon signed-rank test).

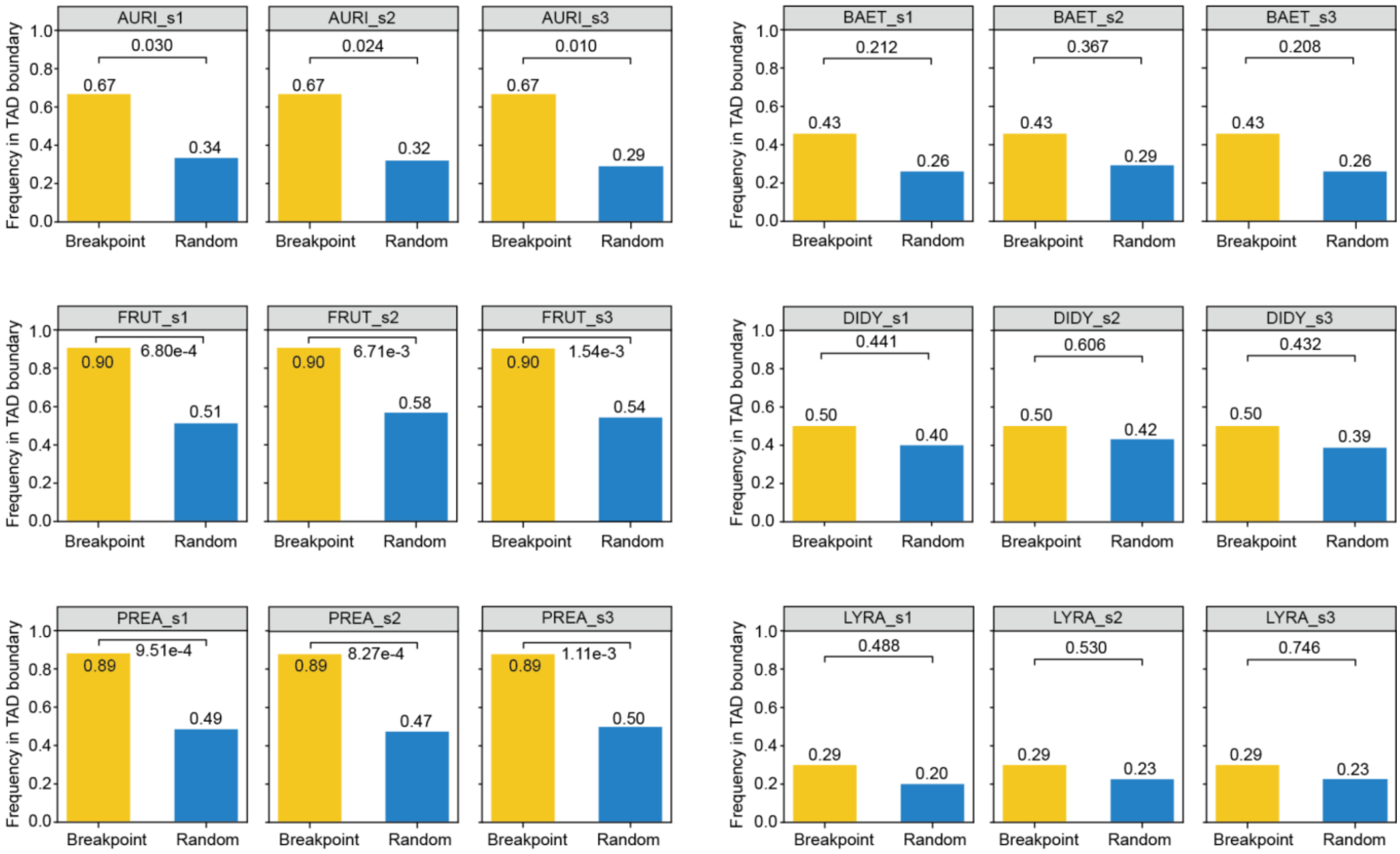

Supplementary Fig. 23 | Comparisons of TAD boundaries distribution in breakpoint regions (yellow bar, abbreviated as Breakpoint) and in 200 simulated random genomic regions (blue bar, abbreviated as Random). Random regions were generated independently three times for each genome (s1–s3), with each set matching the size distribution of breakpoint regions. For each genome and each simulation replicate, statistical significance of the difference in overlap was assessed using two-sided Fisher's exact test. To evaluate the overall trend across genomes and simulations, the overlap frequencies of breakpoint and corresponding random regions were treated as paired observations and compared using a two-sided Wilcoxon signed-rank test. Across all genomes and replicates, breakpoint regions showed a significantly higher frequency of overlap with TAD boundaries than random regions (*P*-value = 1.95×10^{-4}; Wilcoxon signed-rank test).

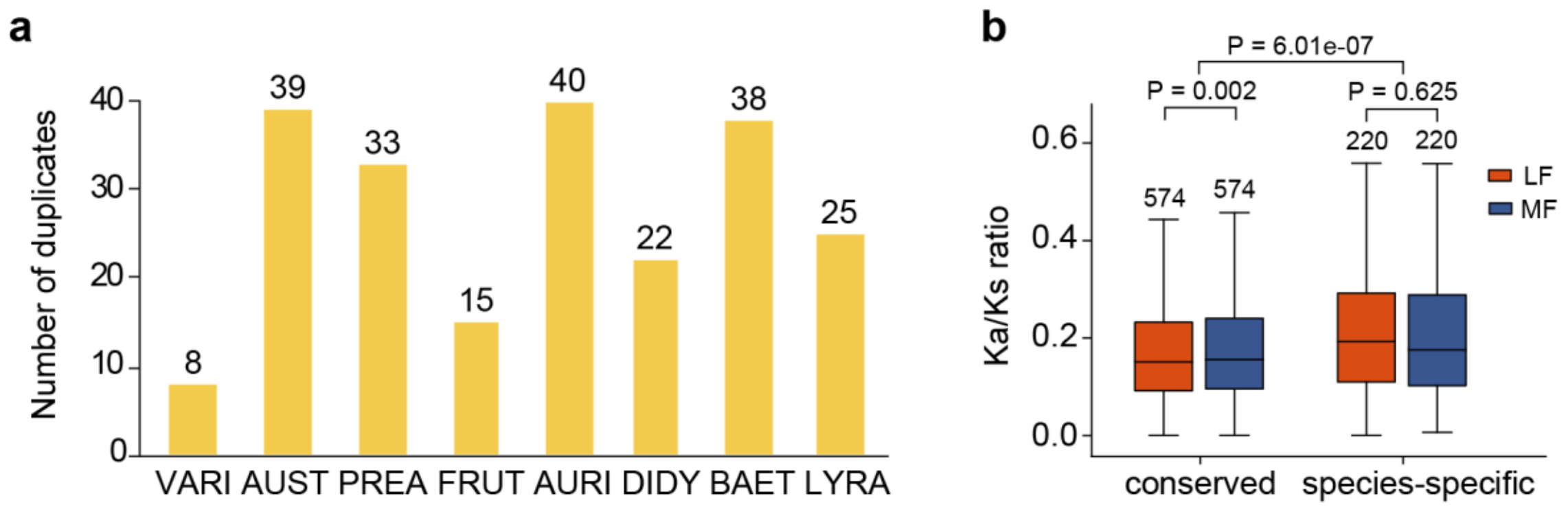

Supplementary Fig. 24 | Sub/neo-functionalization in 1,049 CD gene families. (**a**) Number of duplicates with species-specific sub/neo-functionalization. (**b**) *K*a/*K*s ratios of genes with conserved protein domains and species-specific sub/neo-functionalization in the CD family, with syntelogs in *M. pygmaea* used as reference (two-sided Wilcoxon rank-sum test). Box plot shows the median (centre black line), interquartile range (box limits), and whiskers extending to 1.5× the interquartile range. The sample size (n) is indicated above each box.

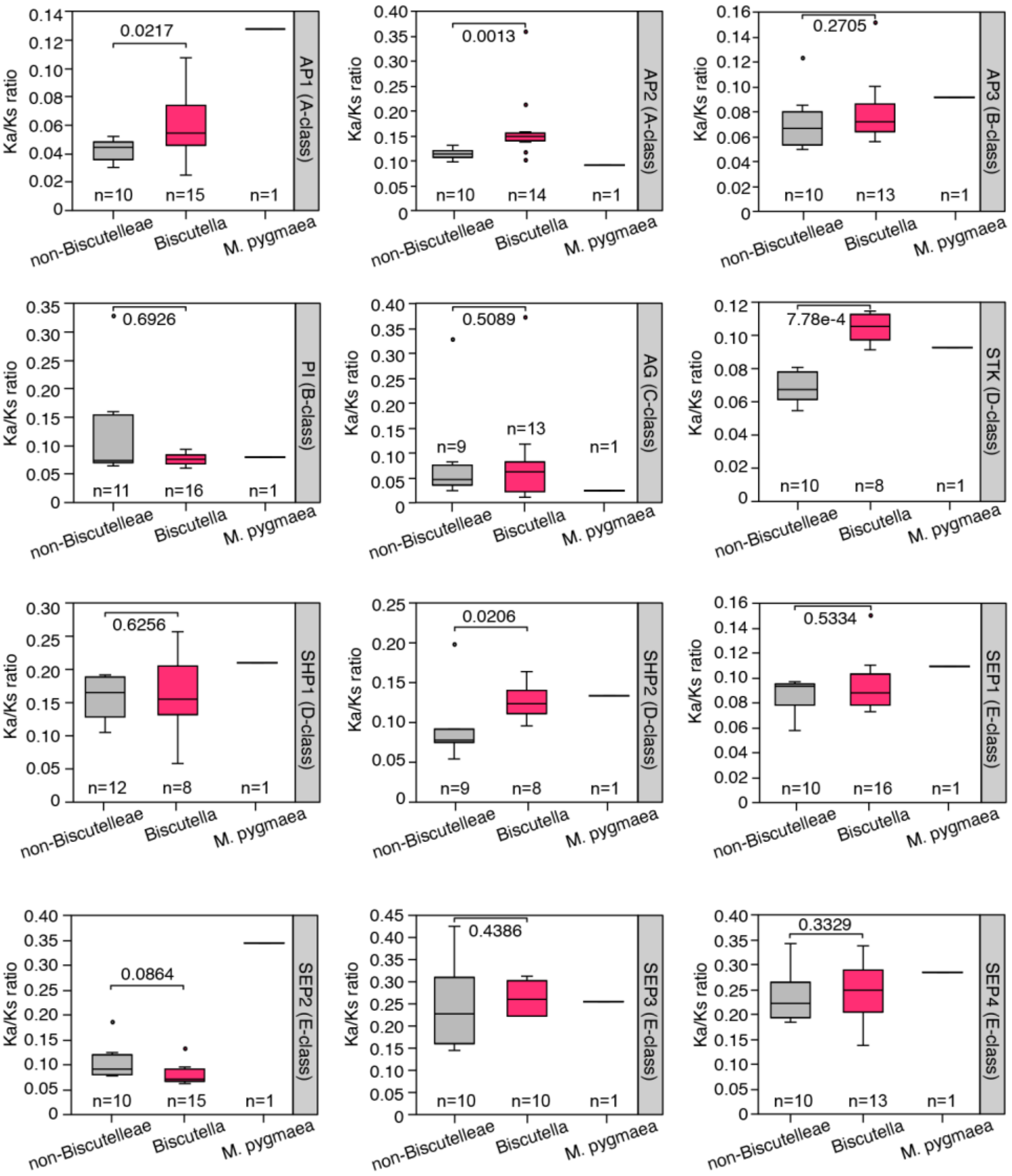

Supplementary Fig. 25 | Box plots of *K*a/*K*s ratios for gene members in the ABCDE flowering model across seven non-Biscutelleae species (including *Arabidopsis thaliana*, *Boechera stricta*, *Brassica rapa*, *Cardamine hirsuta*, *Capsella rubella*, *Pugionium cornutum*, and *Schrenkiella parvula*), eight *Biscutella* species, and *Megadenia pygmaea*, with syntelogs in *Aethionema arabicum* used as reference (two-sided Wilcoxon rank-sum test). Box plot shows the median (centre black line), interquartile range (box limits), and whiskers extending to 1.5× the interquartile range. The sample size (n) is indicated below or above each box.

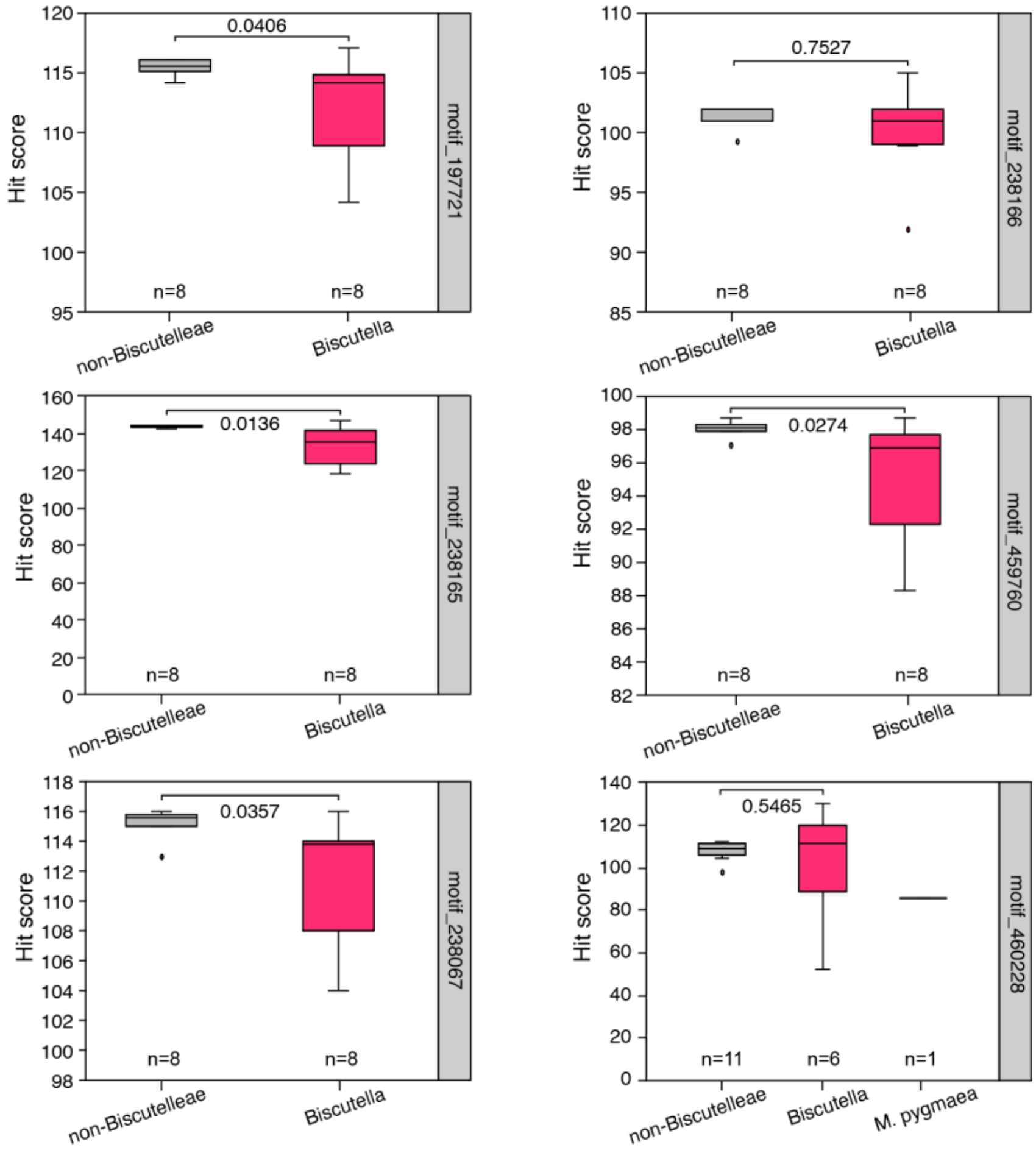

Supplementary Fig. 26 | Hit scores of motifs in the SHP1 gene compared to the NCBI-CDD database. Six major motifs of SHP1 were found based on the MOTIFSearch tool (https://www.genome.jp/tools/motif/), and four of six motifs had significantly lower hit scores in *Biscutella* species than in other Brassicaceae species (including *A. thaliana, Bo. stricta, Br. rapa, Car. hirsuta, Cap. rubella, P. cornutum*, and *S. parvula*). The SHP1 gene in *M. pygmaea* had only one motif (motif_460228) identified and obviously had a low hit score. Box plot shows the median (centre black line), interquartile range (box limits), and whiskers extending to 1.5× the interquartile range. The sample size (n) is indicated below each box. The two-sided Wilcoxon rank-sum tests were performed.

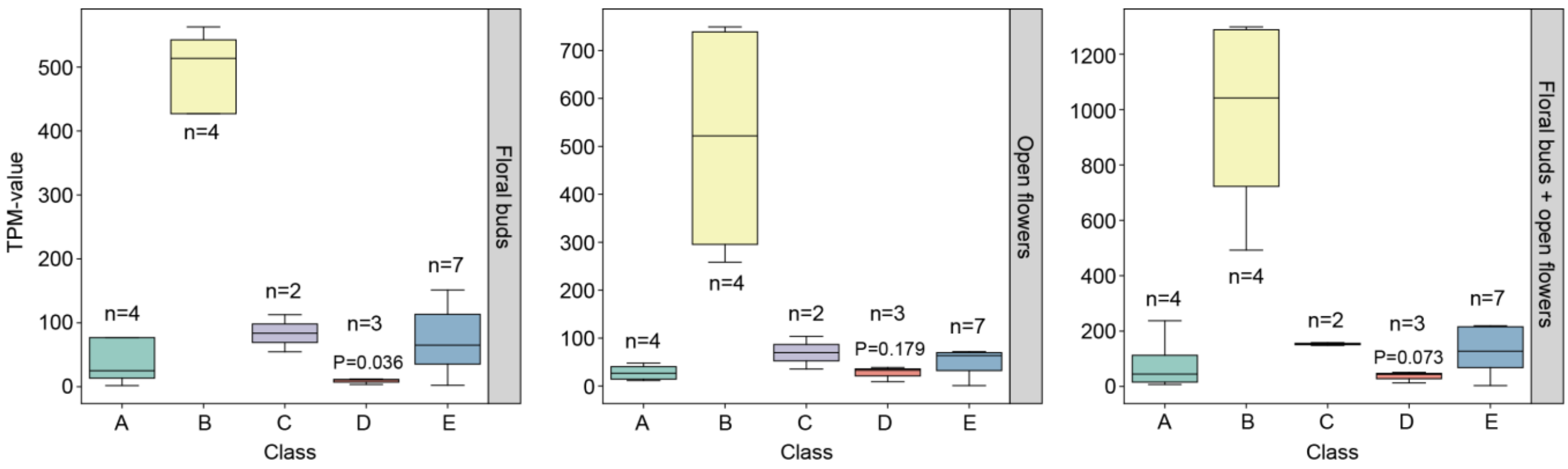

Supplementary Fig. 27 | Expression levels of floral buds and open flower tissues in *B. austriaca* were quantified using transcripts per million (TPM) values. The RNA-seq data for *B. austriaca* were obtained from Beringer et al.[11] The expression levels of D-class genes are generally lower than those of non-D class genes (two-sided Wilcoxon rank-sum test). The sample size (n) is indicated below or above each box.

Supplementary References

1. Mandáková, T. & Lysak, M. A. Post-polyploid diploidization and diversification through dysploid changes. *Curr. Opin. Plant Biol.* **42**, 55-65 (2018).
2. Alonso, M. Á., Vicente, A. & Crespo, M. B. Diversification of *Biscutella* ser. *Biscutella* (Brassicaceae) followed post-Miocene geologic and climatic changes in the Mediterranean basin. *Mol. Phylogenet. Evol.* **142**, 106644 (2020).
3. Vicente, A., Alonso, M. Á. & Crespo, M. B. Born in the Mediterranean: Comprehensive taxonomic revision of *Biscutella* ser. *Biscutella* (Brassicaceae) based on morphological and phylogenetic data. *Ann. Missouri Bot. Gard.* **105**, 195-231 (2020).
4. Lysak, M.A., Mandáková, T. & Schranz, M.E. Comparative paleogenomics of crucifers: ancestral genomic blocks revisited. *Curr. Opin. Plant Biol.* **30**, 108–115 (2016).
5. Kolde, R. & Kolde, M.R. Package 'pheatmap'. *R package* **1**, 790 (2015).
6. Schranz, M. E., Lysak, M. A. & Mitchell-Olds, T. The ABC's of comparative genomics in the Brassicaceae: building blocks of crucifer genomes. *Trends Plant Sci.* **11**, 535–542 (2006).
7. Geiser, C., Mandáková, T., Arrigo, N., Lysak, M. A. & Parisod, C. Repeated whole-genome duplication, karyotype reshuffling, and biased retention of stress-responding genes in Buckler mustard. *Plant Cell* **28**, 17-27 (2016).
8. Mandáková, T. & Lysak, M. A. Chromosomal phylogeny and karyotype evolution in x = 7 crucifer species (Brassicaceae). *Plant Cell* **20**, 2559-2570 (2008).
9. Ou, S., et al. Benchmarking transposable element annotation methods for creation of a streamlined, comprehensive pipeline. *Genome Biol.* **20**, 1-18 (2019).
10. Zhang, R. G., Li, G. Y., Wang, X. L., Dainat, J., Wang, Z. X., Ou, S. & Ma, Y. TEsorter: an accurate and fast method to classify LTR-retrotransposons in plant genomes. *Hortic. Res.* **9**, uhac017 (2022).
11. Beringer, M. et al. Biased retention of environment-responsive genes following genome fractionation. *Mol. Biol. Evol.* **41**, msae155 (2024).
