# Supplementary material for: Post-polyploid chromosomal diploidization in plants is affected by clade divergence and constrained by shared genomic features
Source: Nat Commun. 2026 Jun 6;17:8244. doi: 10.1038/s41467-026-73793-8 (PMC13462695; doi:10.1038/s41467-026-73793-8)
Supplement: Supplementary file 2 [file 41467_2026_73793_MOESM2_ESM.pdf]

Description of Additional Supplementary Files

File Name: Supplementary Data 1

Description: Sequencing reads used for assemblies and gene annotations of the *Biscutella* genomes. The RNA-seq data for *B. baetica* and *B. lyrata* were sourced from Guo *et al*., 2021.; the Illumina, Hi-C, and RNA-seq data for *B. austriaca* were sourced from Beringer *et al*., 2024.

File Name: Supplementary Data 2

Description: Statistics of the eight *Biscutella* genome assemblies.

File Name: Supplementary Data 3

Description: The BUSCO scores of eight *Biscutella* genomes using the genome and protein models.

File Name: Supplementary Data 4

Description: Statistics of various features related to genes based on annotated gene models.

File Name: Supplementary Data 5

Description: Syntenic fragments and (peri)centromeric regions identified in *Biscutella*. The boundary genes at either end of the (peri)centromeres were highlighted in yellow backgrounds.

File Name: Supplementary Data 6

Description: Annotated elements of each transposable element superfamily in *Biscutella* genomes

File Name: Supplementary Data 7

Description: Statistics of tandem repeats in eight *Biscutella* genomes

File Name: Supplementary Data 8

Description: Tandem repeat types abundant in centromeric and subtelomeric regions

File Name: Supplementary Data 9

Description: Chromosomal rearrangement rates in *Biscutella* species

File Name: Supplementary Data 10

Description: Number of solo- and intact-LTRs in the whole-genome, LF, MF, and (peri)centromeric regions

File Name: Supplementary Data 11

Description: GO functional enrichment of the core duplicates gene family. GO terms involved in environmental adaption were highlighted in yellow backgrounds.

File Name: Supplementary Data 12

Description: GO functional enrichment of the core singletons gene family. GO terms involved in chloroplast function and DNA damage repair were highlighted in blue backgrounds.

File Name: Supplementary Data 13

Description: The ABCDE orthologous gene families in 16 Brassicaceae species. Homologous genes (blue font) identified based on similarity using BLAST (Identity %).

File Name: Supplementary Data 14

Description: The detailed data availability information.
