# Supplementary material for: Post-polyploid chromosomal diploidization in plants is affected by clade divergence and constrained by shared genomic features
Source: Nat Commun. 2026 Jun 6;17:8244. doi: 10.1038/s41467-026-73793-8 (PMC13462695; doi:10.1038/s41467-026-73793-8)
Supplement: Supplementary file 3 [file 41467_2026_73793_MOESM4_ESM.pdf]

nature portfolio

Corresponding author(s): Martin A. Lysak and Christian Parisod

Last updated by author(s): Mar 24, 2026

Reporting Summary

Nature Portfolio wishes to improve the reproducibility of the work that we publish. This form provides structure for consistency and transparency in reporting. For further information on Nature Portfolio policies, see our Editorial Policies and the Editorial Policy Checklist.

Statistics

For all statistical analyses, confirm that the following items are present in the figure legend, table legend, main text, or Methods section.

n/a	Confirmed	
☐	☒	The exact sample size (*n*) for each experimental group/condition, given as a discrete number and unit of measurement
☐	☒	A statement on whether measurements were taken from distinct samples or whether the same sample was measured repeatedly
☐	☒	The statistical test(s) used AND whether they are one- or two-sided<br>*Only common tests should be described solely by name; describe more complex techniques in the Methods section.*
☒	☐	A description of all covariates tested
☐	☒	A description of any assumptions or corrections, such as tests of normality and adjustment for multiple comparisons
☐	☒	A full description of the statistical parameters including central tendency (e.g. means) or other basic estimates (e.g. regression coefficient) AND variation (e.g. standard deviation) or associated estimates of uncertainty (e.g. confidence intervals)
☐	☒	For null hypothesis testing, the test statistic (e.g. *F*, *t*, *r*) with confidence intervals, effect sizes, degrees of freedom and *P* value noted<br>*Give P values as exact values whenever suitable.*
☒	☐	For Bayesian analysis, information on the choice of priors and Markov chain Monte Carlo settings
☒	☐	For hierarchical and complex designs, identification of the appropriate level for tests and full reporting of outcomes
☐	☒	Estimates of effect sizes (e.g. Cohen's *d*, Pearson's *r*), indicating how they were calculated

Our web collection on statistics for biologists contains articles on many of the points above.

Software and code

Policy information about availability of computer code

Data collection	Sequencing platforms used to generate the raw data: Illumina HiSeq Xten, Oxford Nanopore Technologies PromethION, and PacBio Sequel II
Data analysis	Genome size measurement: findGSE (v1.94; https://github.com/schneebergerlab/findGSE), Jellyfish (https://github.com/gmarcais/Jellyfish).<br>Genome assembly, scaffolding, and quality assessment: NextDenovo (v2.2; https://github.com/Nextomics/NextDenovo), NextPolish (v1.1.0; https://github.com/Nextomics/NextPolish), hifiasm (v0.19.5; https://github.com/chhylp123/hifiasm), Khaper (https://github.com/lardo/khaper); BUSCO (v3.0.1; https://busco.ezlab.org/), Juicer (v1.5.7; https://github.com/aidenlab/juicer), 3D-DNA (v180922; https://github.com/aidenlab/3d-dna), YaHS (v1.1; https://github.com/c-zhou/yahs), JuiceBox (v1.1.08; https://github.com/aidenlab/Juicebox), GetOrganelle (v1.7.7; https://github.com/Kinggerm/GetOrganelle).<br>Gene prediction and functional annotation: Hisat2 (v2.1.0; https://daehwankimlab.github.io/hisat2/), StringTie (v2.1.4; https://github.com/gpertea/stringtie), GMAP (v2018-07-04; https://github.com/juliangehring/GMAP-GSNAP), TransDecoder (v5.5.0; http://transdecoder.github.io), PASApipeline (v.2.3.3; https://github.com/PASApipeline/PASApipeline), Trinity (v2.11.0; https://github.com/trinityrnaseq/trinityrnaseq), AUGUSTUS (v3.4.0; https://github.com/Gaius-Augustus/Augustus), GeneMark-ET (v4.0; https://genemark.bme.gatech.edu/), GenomeThreader (v1.7.1; https://genomethreader.org/), EVidenceModeler (v1.1.1; https://anaconda.org/bioconda/evidencemodeler), FeatureCounts (https://rnnh.github.io/bioinfo-notebook/docs/featureCounts.html), BLAST (v2.12.0+; https://blast.ncbi.nlm.nih.gov/Blast.cgi), InterProScan (https://github.com/ebi-pf-team/interproscan)<br>Identification of syntenic genes: SynOrths (http://brassicadb.cn:82/download_genome/tools/SynOrths/), NGenomeSyn (https://github.com/hewm2008/NGenomeSyn)<br>Repetitive element annotation: Extensive de novo TE Annotator (EDTA v1.8.3; https://github.com/oushujun/EDTA), LTR_retriever (v2.9.6; https://github.com/oushujun/LTR_retriever), TRASH (https://github.com/vlothec/TRASH), Barrnap (v0.9; https://github.com/tseemann/barrnap), TEsorter (v1.4.6; https://github.com/zhangrengang/TEsorter), soloLTRseeker (https://github.com/estpr/soloLTRseeker), BCT package (https://www.rdocumentation.org/packages/BCT/versions/1.2)

Phylogenetic analysis: MAFFT (v7.427; https://github.com/GSLBiotech/mafft), TrimAL (v1.4; https://vicfero.github.io/trimal/), IQ-TREE (v1.6.11; http://www.iqtree.org/), ASTRAL-Pro (v1.1.3; https://github.com/chaoszhang/A-pro), Toytree (v.3.0.5; https://github.com/eaton-lab/toytree)
Subgenome phasing and validation: SubPhaser (v1.2; https://github.com/zhangrengang/SubPhaser).
Calibration of the WGD timing: KaKs_Calculator (v2.0; https://github.com/lizzhao/Kaks_Calculator), r8s (v1.81; https://sourceforge.net/projects/r8s/), PATHd8 (v1.0; https://www2.math.su.se/PATHd8/), RelTime method in MEGA X (https://www.megasoftware.net/), OrthoFinder (v2.5.4, https://github.com/davidemms/OrthoFinder),
Reconstruction of the ancestral karyotype: WGDI (https://github.com/SunPengChuan/wgdi)
Gene functional enrichment: clusterProfiler package (v3.14.3; https://bioconductor.org/packages/release/bioc/html/clusterProfiler.html)
3D genome analysis: BWA-MEM (v0.7.17; https://github.com/lh3/bwa), HiCExplorer (v3.7.2; https://github.com/deeptools/HiCExplorer)

For manuscripts utilizing custom algorithms or software that are central to the research but not yet described in published literature, software must be made available to editors and reviewers. We strongly encourage code deposition in a community repository (e.g. GitHub). See the Nature Portfolio guidelines for submitting code & software for further information.

Data

Policy information about availability of data

All manuscripts must include a data availability statement. This statement should provide the following information, where applicable:

- Accession codes, unique identifiers, or web links for publicly available datasets
- A description of any restrictions on data availability
- For clinical datasets or third party data, please ensure that the statement adheres to our policy

The genome assemblies and annotations generated in this study have been deposited in the Figshare (https://doi.org/10.6084/m9.figshare.28451828). The raw DNA sequencing data has been deposited in NCBI's Sequence Read Archive database (https://www.ncbi.nlm.nih.gov/) under BioProject number PRJNA1439612 and also in CNGBdb (https://db.cngb.org/) under Project ID CNP0007292. The RNA sequencing data for B. baetica and B. lyrata have previously been deposited in NCBI's Sequence Read Archive database (https://www.ncbi.nlm.nih.gov/) under BioProject number PRJNA634714. The RNA sequencing data for B. austriaca have previously been deposited in the European Nucleotide Archive repository (https://www.ebi.ac.uk/ena/browser/home) under accession number ERP132985. All other data needed to evaluate the conclusions in this study are provided in the Supplementary Information/Source Data file. Source data are provided with this paper.

Research involving human participants, their data, or biological material

Policy information about studies with human participants or human data. See also policy information about sex, gender (identity/presentation), and sexual orientation and race, ethnicity and racism.

Reporting on sex and gender	N/A
Reporting on race, ethnicity, or other socially relevant groupings	N/A
Population characteristics	N/A
Recruitment	N/A
Ethics oversight	N/A

Note that full information on the approval of the study protocol must also be provided in the manuscript.

Field-specific reporting

Please select the one below that is the best fit for your research. If you are not sure, read the appropriate sections before making your selection.

☒ Life sciences ☐ Behavioural & social sciences ☐ Ecological, evolutionary & environmental sciences

For a reference copy of the document with all sections, see nature.com/documents/nr-reporting-summary-flat.pdf

Life sciences study design

All studies must disclose on these points even when the disclosure is negative.

Sample size	Sample sizes (n = 8; i.e., the number of species or individuals sequenced) were determined based on the availability of high-quality biological materials and the aim to adequately represent the phylogenetic diversity and genomic characteristics (covers all chromosomal number variations) relevant to this study.
Data exclusions	No samples were excluded. Raw sequencing data were quality filtered as described in the manuscript.
Replication	All experiments were conducted on different days and showed similar trends. Three different samples from each species were analyzed over three consecutive days to estimate genome size. Genome annotations were validated using three comparative methods. Additional replicate

	analyses supporting these conclusions are listed in the manuscript, such as phylogenomic analyses using four methods and subgenome phasing using three methods.
Randomization	Samples were randomly allocated into experimental groups.
Blinding	Blinding was not applicable to this study. Although multiple species (or samples) were included, they were not assigned to experimental groups, and no treatment or intervention was applied. Sample collection and sequencing followed standardized procedures. All downstream analyses were conducted using computational pipelines.

Reporting for specific materials, systems and methods

We require information from authors about some types of materials, experimental systems and methods used in many studies. Here, indicate whether each material, system or method listed is relevant to your study. If you are not sure if a list item applies to your research, read the appropriate section before selecting a response.

Materials & experimental systems

n/a	Involved in the study	
☒	☐	Antibodies
☒	☐	Eukaryotic cell lines
☒	☐	Palaeontology and archaeology
☒	☐	Animals and other organisms
☒	☐	Clinical data
☒	☐	Dual use research of concern
☐	☒	Plants

Methods

n/a	Involved in the study	
☒	☐	ChIP-seq
☐	☒	Flow cytometry
☒	☐	MRI-based neuroimaging

Plants

Seed stocks	B. varia (V12-4), B. austriaca (Jord.) Mach.-Laur. (A2Schnee 3B), B. prealpina Raffaelli & Baldoin (RCBO_NC17), B. frutescens Coss. (PI 650129), B. auriculata L. (PI 650127, B. didyma L. (LBN-00579), B. baetica Boiss. & Reut. (Gaucín), and B. lyrata L. (Cádiz).
Novel plant genotypes	No novel genotypes were produced.
Authentication	No authentication procedures were used.

Flow Cytometry

Plots

Confirm that:

☒ The axis labels state the marker and fluorochrome used (e.g. CD4-FITC).

☒ The axis scales are clearly visible. Include numbers along axes only for bottom left plot of group (a 'group' is an analysis of identical markers).

☐ All plots are contour plots with outliers or pseudocolor plots.

☐ A numerical value for number of cells or percentage (with statistics) is provided.

Methodology

Sample preparation	The young intact leaf, ~1 cm in length, were prepared. The samples were stained using a solution containing propidium iodide + RNAase IIA, both at final concentrations of 50 µg/ml, for 5 min at room temperature.
Instrument	CyFlow cytometer Partec equipped with a 532 nm diodepumped solid-state laser Cobolt Samba.
Software	FloMax FCS 2.0
Cell population abundance	CyFlow cytometer automatically collects cells and counts the number (~5000 nuclei).

Gating strategy

N/A

☐ Tick this box to confirm that a figure exemplifying the gating strategy is provided in the Supplementary Information.
