# Supplementary material for: Post-polyploid chromosomal diploidization in plants is affected by clade divergence and constrained by shared genomic features
Source: Nat Commun. 2026 Jun 6;17:8244. doi: 10.1038/s41467-026-73793-8 (PMC13462695; doi:10.1038/s41467-026-73793-8)
Supplement: Supplementary file 4 [file 41467_2026_73793_MOESM5_ESM.pdf]

nature portfolio

Peer Review File

Post-polyploid chromosomal diploidization in plants is affected by clade divergence and constrained by shared genomic features

Corresponding Author: Professor Martin Lysak

This file contains all reviewer reports in order by version, followed by all author rebuttals in order by version.

Version 0:

Reviewer comments:

Reviewer #1

(Remarks to the Author)
I appreciate the opportunity to provide a review report for this interesting work, which has been carry over as part of the ECR scheme of Nature Communications Journal and therefore completed in collaboration with a younger researcher.
In this work, authors identified the allotetraploid origin of the plant Biscutella genus, and assembled and annotated the genome of nine species. They demonstrate a range of features contributing to the divergence in genome structure and time of divergence to transposon content. Specifically, authors identified several chromosomal rearrangements, which can explain the different chromosomal number observed in these genomes. Two merged sub-genomes were found in Biscutella originated by previous hybridisation of two ancestors, characterised by a different gene density. This work significantly expands our understanding related to plant genome evolution, by analysing a genus with significant variation in the chromosome number. Authors were able to reconstruct the genome dynamic in the genus and provide a convincing explanation of the variations observed. The work also provides significant resources in term of novel genome assemblies and annotation of Biscutella genus, which will also contribute to downstream metagenomic studies.
The manuscript is generally well written, and the description of the analysis and results is generally well presented. Nonetheless, the manuscript is difficult to follow in some places, with field-specific abbreviations not given in full or explained before use (see below specific comments). There are few main concerns which will be described below:
1) Authors appear to assume that a high S/I ratio can be univocally associated to a higher LTR transposable elements removal rate (figure 4c), but this is true only assuming that transposition rate is constant in time. However, for AURI and DYDY species the LTR TE insertions appear to be generally older compared to other species (figure 4a), suggesting that transposition rates have not been the same in different Biscutella species). So, the higher proportion of solo LTRs found in mostly AURI and DIDY genomes could be simply a consequence of a lower transposition rate, rather than a higher degree of removal? Does this represent an alternative interpretation to the author interpretation?
2) Also, at line 270 authors write that “no specific pattern of soloLTR loss was observed between subgenomes”. However, from figure 4d appears that in a couple of LTR families (Retand and maybe Athila) the S/I ratio is actually quite different among the two subgenomes, for AURI and LYRA species. These families are also the two groups that appear to lead the difference of ratio in early-diverging species. So again, I think this might reflect differential activation rate of these TEs in the different subgenomes for some species, with low S/I ratio being possibly associated to recent transposition bursts?
3) Figure 2a illustrate the difference in term of gene density in the two subgenomes across all species analysed. While in general term it is clear that the trend is different in LF and MF subgenomes, there are exceptions for some blocks. For example, blocks G and T for FRUT and AUST genome appear swapped in the two groups. Also, in block D (and maybe G) for DYDY, BAET and LYRA genomes show the same effect (this is also visible in block O for PREA. Considering that the gene density was used as a parameter to classify the subgenomes, I am wondering why these blocks have been assigned in an apparently inconsistent way. Also, the C block appears to be much more consistent in the difference of gene density and will be probably an outlier compare to both LF and MF averages. Does this reflect the specific genetic composition of this block (e.g. high euchromatin or heterochromatin content)?
4) Finally, related to the finding that breakage hotspots (HOTs) are associated to higher density of LTR-TEs. Authors link this finding with a general hypothesis that interspersed stretches of similar TEs may be substrate of recombination and chromosome rearrangements. While this could be true (and it is discussed also in other works), from the analysis made here it is clear that most of genome rearrangements in Biscutella genus are dated much earlier than the average date of transposition of most of LTR families analysed. In other words, it is probable that most of LTR-TE insertions occurred after

the chromosome rearrangement events. Therefore, I think it is more likely that HOTs are regions that allow for accumulation of TE insertions, rather than TE accumulation here have caused or facilitated the rearrangement itself. Does the authors have any evidence that the accumulation of TEs at HOT was present at speciation time? Moreover, it appears that authors considered only LTR-TE in this analysis, and it is unclear if DNA elements also play a role in HOTs.

Minor comments:
Introduction
Line 94: there is no reference given for the B. baetica studies
Line 102: “eight Biscutella species from morphologically and phylogenetically distinct infrageneric clades”. I think it would be helpful to provide some sort of measure or phylogenetic tree/analysis demonstrating that these are species are phylogenetically distinct. This would help visualise the evolutionary links between the chosen species
Results
First paragraph: Authors state Illumina short-read sequencing has also been used but it is not clear how this has been applied. In the methods it says this was used for chloroplast genome assembly but I could not find this in the results. Maybe for this first paragraph, you should omit the use of Illumina as I was waiting to read about it in the section but it did not appear.
Line 203-228: Multiple abbreviations are given in this section without previous explanation e.g. ACK, ancPCK, V+Wa+Q+X, etc. This makes this section difficult to read and is not accessible to those not familiar with the field and terminology. Are these names of the subgenomes?
Line 246-249: Should Tsuneq be Tuneq as defined in line 233? Are these two different things?
Line 257: what are you defining as an “intact” LTR-RT and how did you identify these?
Line 311: “67.5% of these regions had higher LTR-RT content than the genome-wide average mainly contributed by Athila and CRM retroelements (Figure 5c).” There is no presentation of the genome wide average, this would be helpful to include to aid comparison and give context for the result
Line 344: “the late-diverging species had significantly higher Ka/Ks ratios (Figures 6e and 6f)”. There is no significant difference between early and late diverging in 6f
Discussion
The discussion is very short and fails to discuss some of interesting findings from this paper e.g. the differences in retention of the ABCDE flowering genes, the possible contributions of TEs to genome evolution and divergence etc. It also ends very abruptly with no discussion on the evolutionary significance of the data.
Methods and Figures
Were any biological replicates used?
What weight of tissue was used for Illumina, Hi-C, and RNAseq?
Line 458: what do you mean by “young leaf”? What age were the plants?
In the pdf version of the manuscript formulas are not printed correctly (e.g. line 648, line 721).
Figure 4C: I do not think it is correct to present this as continuous data. It suggests you are tracking a change when you are noting a difference between species. A heading for each of the Gypsy and Copia lineages would also be helpful for understanding the figure immediately
Figure 5a: you indicate HOTs shared within clade I or II with the same arrow colour. Can you separate out and provide a different arrow for each? This currently does not provide information on the HOTs identified that may contribute to clade divergence.

Reviewer #2

(Remarks to the Author)
The study provides a comprehensive analysis of post-polyploid genome evolution in Biscutella species, emphasizing descending dysploidy and chromosomal rearrangements after whole-genome duplication. Its focus on the recurrent, independent chromosome reductions and the underlying genomic features, such as LTR retrotransposons and 3D genome architecture, significantly advances our understanding of karyotype evolution in Brassicaceae. The integration of high-quality genome assemblies with evolutionary inference adds valuable insights that are currently underrepresented in the literature. Overall, the work addresses a notable gap concerning the mechanistic basis of descending dysploidy, especially in a comparative, multi-species context. However, several critical issues must be addressed before the manuscript is suitable for publication in Nature Communications.

Major Comments
1. The study offers valuable insight into independent versus convergent diploidization pathways within a single genus. However, similar analyses have been reported in other Brassicaceae—for example, Brassica spp. (Lysak et al., 2007 https://pmc.ncbi.nlm.nih.gov/articles/PMC2048728/ ; Mandáková & Lysak, 2018 https://pubmed.ncbi.nlm.nih.gov/29567623/) and in cotton (Wang et al., 2021 https://academic.oup.com/mbe/article/38/9/3621/6262643). The authors should more clearly articulate what novel principles emerge specifically from Biscutella that were not apparent in those systems.
2. The identification of 12 “breakage hotspots” enriched in LTR-RTs and overlapping TAD boundaries is intriguing, but the causal link between TE content, 3D architecture, and recurrent rearrangements remains correlative. Could the authors provide any functional assays (e.g., cytological evidence of DSB hotspots or chromatin accessibility data) to strengthen this claim?
3. Eight species spanning three chromosome numbers is a solid start, but the phylogenetic sampling is uneven: the n = 9 clade is represented by four species, whereas the n = 6/8 clade includes only three plus B. auriculata. Given the young divergence of the late-diverging clade (~3.3 Mya), are four genomes sufficient to capture its variability? Conversely, should

the authors have included more early-diverging taxa (e.g., additional outgroups) to more robustly infer ancestral karyotypes?
4. The coalescent analyses (ASTRAL-Pro) show substantial gene-tree discordance. The manuscript would benefit from quantitative measures (e.g., proportion of quartets or gene trees supporting key nodes) and discussion of how this uncertainty impacts the reconstruction of the ancestral genome.
5. Table S2 reports contig N50 = 2.9 Mb for ONT assemblies versus 22.9 Mb for the PacBio B. lyrata genome. The methods should specify whether any haplotigs or alternative haplotypes were purged, and what BUSCO duplications (if any) indicate residual haplotype duplication.
6. Solo LTR identification via the novel “soloLTRseeker” pipeline is briefly described, but no benchmarking is provided against established tools (e.g., LTR_retriever’s soloLTR calls). Moreover, the mutation rate ($r = 1.5\times10^{-8}$) assumed for insertion dating should be justified for Biscutella specifically, as rates can vary substantially across lineages.
7. The assignment of A/B compartments at 50-kb resolution and TAD calling methods are insufficiently detailed. Which tool and parameters were used to call TADs? How sensitive are the results to resolution or calling thresholds? Including reproducibility details (e.g., code repositories, parameter files) is essential.
8. Rates of chromosomal rearrangements (CRs/Mya) are reported (e.g., 3.75 CRs/Mya early vs. 0.72 CRs/Mya late), but no confidence intervals or statistical tests are provided. Given phylogenetic uncertainty in divergence times, how robust are these rate estimates?
9. The manuscript asserts that shared genomic features predispose certain regions to recurrent breakage. Yet, without functional validation (e.g., assays of recombination frequency or chromatin state), this remains hypothetical. The discussion should be more tempered and explicitly acknowledge the correlative nature of the evidence.
10. The proposed link between D-class MADS-box gene fractionation and evolution of lens-like silicles is intriguing, but entirely based on Ka/Ks and copy-number patterns. Are there expression data or phenotypic analyses that can corroborate this hypothesis? Otherwise, it should be presented only as a plausible model.
Minor Comments
1. The Abstract contains a duplicate sentence (lines 18–22): “we identified 12 chromosome breakage hotspots…” Please remove redundancy.
2. Clarify whether the TE enrichment analyses account for local GC content or other genomic features that influence TE distribution
3. Inconsistent nomenclature: the terms “Tsuneq,” “Tuneq,” and “unequal reciprocal translocations” appear interchangeably. Adopt a single abbreviation throughout.
4. The description of the two clades as “early-diverging (n = 8/6)” and “late-diverging (n = 9)” may confuse readers; consider labeling them “clade I” and “clade II” or similar, with an explicit note that clade I corresponds to basal lineages.
5. Describe the criteria used for defining and detecting chromosomal breakpoints and rearrangements.
6. In Figure 3b, the CR events are represented by colored arrows but the legend does not clarify which color corresponds to which event type.

Overall, the manuscript presents a valuable dataset and addresses an important question in plant genome evolution, but the concerns above must be addressed before acceptance.

Reviewer #3

(Remarks to the Author)
I co-reviewed this manuscript with one of the reviewers who provided the listed reports. This is part of the Nature Communications initiative to facilitate training in peer review and to provide appropriate recognition for Early Career Researchers who co-review manuscripts.

Reviewer #4

(Remarks to the Author)
Dear authors:

Overall, this is a very well analyzed, interpreted, and written manuscript, presenting findings of broad general interest: by sequencing eight Biscutella species descended from an allotetraploid ancestor (n=14), the study demonstrates that independent descending dysploidy (reducing chromosomes to n=9, 8, 6) involves both convergent and divergent genomic features and identifies recurrent chromosome breakpoint hotspots enriched in LTR retrotransposons at TAD boundaries, suggesting underlying shared mechanisms despite the process appearing random. I only have a few minor comments as shown below:
Line 40-42: This sentence was written twice in the Abstract section.
Line 66: Please provide the full expression of DSB for the first time.
Line 69-70: Please provide the reference for this sentence.

Reviewer #5

(Remarks to the Author)
Huang and colleagues present a manuscript titled: “Post-polyploid chromosomal diploidization in plants is affected by clade divergence and constrained by shared genomic features” that reports on the diploidization process from an allopolyploid ancestor in the Biscutella genus using whole genome sequences and Hi-C chromosome conformation of 8 (sub)species.

The authors show that the different species underwent distinct diploidization histories leading to variable chromosome numbers and arrangements. Interestingly, even species with the same chromosome number underwent different diploidization histories. The early-diverging clade is shown to have in general lower chromosome number, higher variability of TAD length/number and A/B compartment assignment, and higher retrotransposon removal. Interestingly, the authors also identify specific breakpoint hotspots that are found across multiple species, which very often occur in (peri)centromeric regions and are associated with TAD boundaries. Finally, the authors explore (more briefly) genic diploidization and functional biases therein. In all, the authors present a thorough and high quality description of genome evolution in the Biscutella genus. The methodology is sound and reproducible. The authors use different approaches to confirm their conclusions (e.g., bottom-up and top-down approach for inferring the ancestral karyotype).
We believe this work will be of considerable significance for the field of polyploidy and diploidization. To our knowledge, this is the first paper to investigate chromosomal diploidization by analyzing the genomes across multiple species in a genus originating from a common polyploid ancestor. It follows on earlier cytogenic-based work on diploidization of the same research group (but not this clade, e.g., https://doi.org/10.1111/mec.14379).
Me and my co-reviewer have five major comments on the methods and discussion that need more consideration and we raise a number of small comments in a line-by-line manner.
For the top-down ancestral reconstruction, the authors indicate “A shared paracentric inversion event (Ipa), which restructured the ancestral chromosome AK8/6 by rearranging the V+Wa+Q+X association into V+X+Q+Wa, was identified in both subgenomes in all Biscutella species” (Lines 211-213). However, I do not observe this in Figure S12a, at least not completely. For example the association between V+X in the LF sub-genome is only found in 3 (all n = 8 species) of the 6 karyotypes. How can this be explained?
Related to results of this top-down strategy that are represented in figure S12. If I correspond the presence and absence of gene blocks neighbor pairs, not all of them seem to be present here. E.g., according to figure S17 the lyrata genome on chr4 has a K gene block neighboring I1 and B. Figure S12 has the I+KL as presence for lyrata, but no K+B (red nor grey). Was this K+B not considered? Is this only a partial representation or was there only a subset of neighbor pairs considered? It does not seem the case that only neighbors in the ancestral (A3) reconstruction are shown because D + U is a pair that was formed between A3 and lyrata. Only pairs between a MF and LF gene block are not considered? Please clarify in methods.
The breakpoint hotspot regions were identified as breakpoints shared by multiple species compared to the ancestral genome. What remains unclear to me is whether these breakpoints were shared because it originated in the ancestor of these species or whether the breakpoints occurred by convergence. The authors mention that these breakpoints are recurrent, but it is not clear whether this means that these breakpoints occurred multiple times independently or if it occurred once in their post-polyploid common ancestor. If this was not considered yet, I believe that categorizing breakpoint hotspots in ancestral and convergent breakpoints is essential for the interpretation of convergence.
While the article does propose a hypothesis on the convergent genome evolution in the diploidization process through chromosome rearrangements (line 75), the results and discussion do not really focus on this hypothesis explicitly. Consider making the focus on this hypothesis stronger by discussing what results show a convergent pattern and what underlying universal process could explain it.The current state of the manuscript provides does not go further than a discription of a number of patterns, but by answering this hypothesis could provide a stronger scientific conclusion and justify the title of the manuscript.
Finally, I think the paper would be even stronger if the discussion delved a bit deeper in the findings of the study. For example, I think the underlying cause of the association between TAD boundaries and breakpoint hotspots is not obvious and I wonder whether the authors have any hypotheses regarding that. In addition, I wonder what might have caused the observed patterns between the early-diverging and late-diverging clades. Is it just because the divergence time was more ancient that there was more variability observed in the early-diverging clade? I also wondered whether the authors have an idea about the underlying cause of the differential gene fractionation between the sub-genomes.
Specific comments, by line number:
Line 37: It would be more clear to use the term “gene” fractionation (across the whole manuscript) as it emphasizes the fact that you are talking about “gene” loss following polyploidy.
Line 42: “descendig” should be “descending”.
Line 45: Which species are you referring to, to have two and several hundred chromosome pairs?
Lines 46-48: “This extraordinary variation is due to two antagonistic evolutionary processes: polyploidy (whole-genome duplication, WGD) and diploidization.” It's a strong claim that only these two processes are responsible for the variation in chromosome numbers in all land plants. While maybe the case in Biscutella, is there support for this claim across all plants (by means of references and/or better argument)?
Lines 56-57: “a common way of descending dysploidy is non-allelic homologous recombination (NAHR) between two or more different chromosomes, reducing the number of chromosomes typically by one...” Since this process is central to the manuscript, you may want to explain this a bit further (E.g., how can recombination reduce the number of chromosomes?) and/or provide a figure (if journal limits allow it; something along the lines of figure S14 but maybe simpler).
Lines 58-59: Is “diploidizing descending dysploidy” not by definition after WGD events?
Line 65: This refers to “DNA homology and repeat content” between non-homologous chromosomes then?
Line 66: DSB not yet explained
Lines 69-71: “WGD events often occur before the diversification of a clade, leading to the assumption that all clade members are descended from a single polyploid genome or metapopulation. The polyploid clade diversifies as a result of adaptive radiation...” Please include references for these statements.
Line 135: Could you introduce these 22 ancestral genomic blocks? At least I was not familiar with it.
Line 141: I do not observe this exception for block G in B. prealpina and B. varia in Figure S2, maybe due to lower resolution.
Lines 193-195: Note that the dating represents not necessarily the time of hybridization, but rather the divergence of the (diploid) progenitor species.
Line 207: Please write ACK, ancPCK and PCK first time in full.

Lines 246-249: Why would you have expected Tsuneq to be correlated and for example not EET and NCI, as these reduce the number of chromosomes directly?
Lines 267-270: Have you done a statistical test to confirm this pattern?
Line 308: "In the extant Biscutella genomes, more than half of the rejoined junctions aligned with pericentromeric regions (Figure 5b)." Is there a (statistical) test against an expectation possible to show that rejoined paleojunctions associated with a) pericentromere, b) TAD boundaries and c) A/B compartment boundaries (so figure 5b). E.g., the likelihood of being a TAD boundary by chance can be expected to be (the zone that is considered boundary)/(average TAD length). It would grant more power to statements being made based on arbitrary TAD boundary size and pericentromeric region sizes. 23/40 sounds like not a lot if half the chromosome is considered as pericentromeric. Alternatively, mention this context somewhere of how big the pericentromeric region, TAD boundaries and A/B boundaries are to better interpret these results.
Line 401 "and chromosome translocations" are you meaning "unequal chromosome translocations"? Otherwise overlap with the end-to-end translocations
L505: What is the reason for 2 strategies for different species and how did you choose the strategy for each particular species?
Lines 618-619: The methodology of how orthogroups were inferred seems to be missing.
Line 648: Formula conversion in PDF format failed.
Line 721: Formula conversion in PDF format failed.

Reviewer #6

(Remarks to the Author)
I co-reviewed this manuscript with one of the reviewers who provided the listed reports. This is part of the Nature Communications initiative to facilitate training in peer review and to provide appropriate recognition for Early Career Researchers who co-review manuscripts.

Reviewer #7

(Remarks to the Author)
I co-reviewed this manuscript with one of the reviewers who provided the listed reports. This is part of the Nature Communications initiative to facilitate training in peer review and to provide appropriate recognition for Early Career Researchers who co-review manuscripts.

Version 1:

Reviewer comments:

Reviewer #1

(Remarks to the Author)
In this revised manuscript, authors addressed most of my points providing a remarkable high amount of information.
There are however few answers to the main points still matter of concern for me:
1) Concerning potential different transposition rate in AURI and DYDY:
If it is assumed that solo LTRs are necessary deriving from full insertions, I do not understand why the estimation of full insertions and solo LTRs age should be different. I appreciate the time of insertion should be more precise when measured on the full size LTR elements (taking advantage of direct comparison of the two LTRs), compared than the solo LTRs. In fact, a sudden increase of LTR activity (in more recent time) might alter the S/I ratio independently by the age of solo LTR insertions (accumulated on a larger timeframe). In my opinion, the fact that the alteration of the S/I appear to be only associated to certain TE families (figure R1.2) is in fact reinforcing the idea which is linked to transposition rate (which might be variable depending by the TE), rather than LTR-TE removal (which should happen at the same rate for all LTR-TE). In absence of different evidence or a potential misunderstand from my side of author rebuttal, I would like to have the alternative hypothesis of variable LTR-TE transposition clearly considered in the manuscript text as potential alternative explanation of the altered S/I ration observed.

2) Concerning the breakage hotspots (HOTs)
In my understanding authors have confirmed that more recent TE activity (compared to the WGD) could have masked potential evidence of TE accumulation at HOTs when Biscutella speciation occurred. Therefore, most of insertion find at HOTs indeed occurred after chromosomal rearrangements. I am therefore surprised by the relatively mild revision of the text occurred at lines 310-312. I think the text still exclude (or avoid to mention) the idea, still very plausible to me, which are HOT regions that attract (or maintain) TE insertions, rather than TE accumulation favouring rearrangement. It could be simply because these highly variable regions do not contain regulatory or coding DNA (so that rearrangements are mostly under neutral selection) or maybe are characterised by specific chromatin arrangements which favour both recombination and TE insertion. Again, also in this case I would specifically mention about this alternative hypothesis, if authors cannot rule out the possibility.

Reviewer #2

(Remarks to the Author)

I would like to thank the authors for their thorough and thoughtful responses to my previous comments. They have addressed all of my concerns meticulously, and the manuscript has been significantly improved as a result. I have no further suggestions and recommend acceptance.

"I co-reviewed this manuscript with one of the reviewers who provided the listed reports. This is part of the Nature Communications initiative to facilitate training in peer review and to provide appropriate recognition for Early Career Researchers who co-review manuscripts"

Reviewer #5

(Remarks to the Author)
Reply on rebuttal “Post-polyploid chromosomal diploidization in plants is affected by clade divergence and constrained by shared genomic features”

The authors have addressed our remarks in a thoughtful and detailed manner, which is highly appreciated. I believe they successfully addressed our remarks, and, where necessary, adapted the manuscript. By performing additional analyses (namely analysis of the evolution of breakpoints) and expanding the discussion, I believe the manuscript has been strengthened. As indicated in the first review round, this paper will be of considerable significance for the field of polyploidy and diploidization. Nonetheless, I still have a few remarks:
Remarks on the hotspot part
1. You categorize hotspots into ancestral and recurrent “hotspots”. However, the ancestral hotspots indicate that only one chromosome junction happened which was inherited in multiple species, while a “hotspot” implies that it happened multiple times in this region. Consider renaming the ancestral hotspot category.
2. The authors use a simulation-based approach to assess significance of overlap between HOT regions and other genomic features (pericentromere, TAD boundaries, A/B compartment boundaries), which is justified. In Lines 756-759, the authors indicate that HOT regions encompass typically two rejoined junctions that have a typical length of 300kb and that is the reason for the choice of the 150 kb random segments. This only makes sense if the random segments are 200 combination of two 150kb segments but this does not seem to be mentioned? Either way, I think it would be better to simulate using the exact sizes of the identified HOT regions (or the sizes of the identified joined segments that make up the HOT region), so that you can really rule out effect of size of a HOT region. If not, could you clarify further how the 150kb was choosen?
3. You show the results of these simulations by comparing patterns your identified HOT regions to an expected average pattern in the simulated segments. However, you do not compare statistically, which would involve variation in both observed and random expected, even though you mention in your response to my previous comment that differences are ‘statistically significant’. You test significance in in Supp figure 23, but not in 21, 25 and 26. While the association with pericentric regions are convincing, those with TAD and A/B are less so.

Small remarks:
Line 59-60: I do agree with the authors that descending dysploidy can occur independently from a WGD event. What I meant with the previous remark “Is diploidizing descending dysploidy not by definition after WGD events?” is that adding the “diploidizing” in front of the term already implies that it is following a WGD. I think the sentence would be more accurate if phrased as e.g. “Descending dysploidy is particularly common after WGD events (i.e., diploidizing descending dysploidy)”.
Line 140-141: I think the fact that it supports the reliability of gene annotations is based on the fact that you get the expected 2:1 pattern given its meso-tetraploid ancesstry. I think, in that case, it would be better to put these lines after the lines about its meso-tetraploid ancestry where the 2:1 pattern is explained.
Line 212: If you see the X+Q+Wa association it could also support the opposite claim that it does not support the shared paracentric inversion event. Nonetheless, I agree with the authors that the presence of the complete V+Wa+Q+X association in most of the species on the LF sub-genome and all species on the MF sub-genome supports the shared hypothesis.
Line 748: “the interval region often encompassed the centromere and excessively large, which could bias downstream analyses” The “and excessively large” seems out of place or missing a part in this sentence.
Line 763: Could you clarify whether these orthologous gene families identified with OrthoFinder or SynOrths?
Supplementary figure 23: “…our findings indicate that non-pericentromeric HOTs also generally display elevated TE accumulation” You show it convincingly for the DNA-TE and helitron, but only in 2 of 6 species for LTR-RT. Consider toning down or specifying the conclusion on the TE association.

Reviewer #6

(Remarks to the Author)
I co-reviewed this manuscript with one of the reviewers who provided the listed reports. This is part of the Nature Communications initiative to facilitate training in peer review and to provide appropriate recognition for Early Career Researchers who co-review manuscripts.

Reviewer #7

(Remarks to the Author)
I co-reviewed this manuscript with one of the reviewers who provided the listed reports. This is part of the Nature Communications initiative to facilitate training in peer review and to provide appropriate recognition for Early Career Researchers who co-review manuscripts.

Version 2:

Reviewer comments:

Reviewer #5

(Remarks to the Author)
We thank the authors of the manuscript “Post-polyploid chromosomal diploidization in plants is affected by clade divergence and constrained by shared genomic features” for taking our and the other reviewers feedback at heart. We feel that the manuscript is ready for acceptance except for one of the added statistical tests (Wilcoxon signed rank). We outline our problem with it in a comment below but suggest to just remove it and tone down the conclusion about association with TAD boundaries and A/B transitions.

308-311: “Analysis of the LTR-RT density in 100-kb regions on each side of rejoined junctions showed that more than 97% of these regions had higher values than the genome-wide average, and around half of them higher than expected from random controls (Supplementary Figs. 22, 23 and Supplementary Table 11).” The 97% percent comes from 46/47 breakpoint regions seen in fig. S22. This suggests the breakpoint regions are independent, which they are not because of phylogenetic relatedness. This statement is also a bit misleading since the number of regions with higher LTR-RT density than the average does not say anything about how much more LTR-RT you can find there compared to the random expected.
From the response: “While the Fisher’s exact test results did not reach statistical significance in certain cases (e.g., Supplementary Figure 24: A/B compartment transitions and Supplementary Figure 25: TAD boundaries distribution in BAET, DIDY, and LYRA), the three independent simulations consistently yielded the same qualitative trends of difference between the breakpoint regions and random sampled regions (which we believe is more important). To validate these trends further, we conducted Wilcoxon signed-rank tests (a non-parametric statistical test used to compare paired samples) to compare the replicate results for the breakpoint and random regions across all species. All the P-values were extremely significant. Collectively, we believe that the consistent directional trends across replicates, combined with the significant results of the Wilcoxon signed-rank tests, provide robust support for the conclusions presented.” I appreciate that the authors provide Fisher’s exact test significance scores for the other plots. However, the authors now also mention a Wilcoxon signed-rank test that shows significance across all species in both A/B and TAD association with breakpoint regions. There is very little explanation given here as to what exactly is tested. Most of it I derived from their response, but with (as far as I could find) no real explanation in the manuscript. Therefore, readers will have a hard time understanding this analysis.
I am going of the assumption that the authors performed the test on each pair of summary frequencies of A/B transitions in (1) breakpoint vs (2) the random segments of equal length from that genome, i.e., for every panel of fig. S24 (and similarly for S25). This seems like a solution to find support for the intuition that for each species the association for both A/B and TAD with the breakpoint regions is more likely than in the random expectation but without sufficient support within each species individually. I find this unsatisfactory. Apart from problems with non-parametric tests, if I understood what you did correctly, the input for these tests include re-using the same frequency of each species multiple (3) times, which again means that the tested data is not independent (hypothetically, 1 out of 6 species having an extreme frequency may be randomness in a small sample but repeat each observation three times and you have 3 out of 18 which seems more “significant”). The species are furthermore also related which means that in this case, TAD or A/B associations may be inherited from a common ancestor which means there is a phylogenetic signal and therefore additional non-independence.
To pool these data and simulated random expectation across all species, you would need a more complicated model I would think (at first glance, a binomially distributed model with the number of TAD associated regions as outcome across total number of regions as draws, and with being breakpoint or simulation and species as predictor and with the phylogenetic signal also as covariate). I will not propose that you do this, because of the developed stage of the manuscript. I would suggest you tone down statements where you claim associations with A/B and TAD: that for each species there is not sufficient evidence but that the higher frequency than the random expectation is found in all species, suggesting some association. E.g. “We found that up to 68% of the breakpoint junctions showed inconsistent A/B compartment assignments and 64% matched TAD boundaries across Biscutella genomes (Fig. 5b). Both of these values are significantly higher than expected from 200 random simulations (Supplementary Figs. 24, 25).” This statement is not factual, since you did compare a set of 200 simulations with all breakpoints that are 68% associated with A/B transitions.
Fig. S23 and Fig. S24: caption does not mention the Fisher’s exact test. Neither does the methods section

Reviewer #6

(Remarks to the Author)
I co-reviewed this manuscript with one of the reviewers who provided the listed reports. This is part of the Nature Communications initiative to facilitate training in peer review and to provide appropriate recognition for Early Career Researchers who co-review manuscripts.

Open Access This Peer Review File is licensed under a Creative Commons Attribution 4.0 International License, which permits use, sharing, adaptation, distribution and reproduction in any medium or format, as long as you give appropriate credit to the original author(s) and the source, provide a link to the Creative Commons license, and indicate if changes were made.
In cases where reviewers are anonymous, credit should be given to 'Anonymous Referee' and the source.
The images or other third party material in this Peer Review File are included in the article's Creative Commons license, unless indicated otherwise in a credit line to the material. If material is not included in the article's Creative Commons license and your intended use is not permitted by statutory regulation or exceeds the permitted use, you will need to obtain permission directly from the copyright holder.
To view a copy of this license, visit https://creativecommons.org/licenses/by/4.0/

Response to reviewers' comments

1. The reviewers' comments are indicated in black font; authors' responses are indicated in red font.
2. Questions are numbered sequentially in the Reviewer-Question (**R-Q**) format.
3. The figures and tables in this file are numbered as **Figure/Table.R (Reviewer number).N (Figure/Table number)**. If a figure was included in the Supplementary Information, its number is also provided.

Reviewer #1 (Remarks to the Author):
I appreciate the opportunity to provide a review report for this interesting work, which has been carry over as part of the ECR scheme of Nature Communications Journal and therefore completed in collaboration with a younger researcher.
In this work, authors identified the allotetraploid origin of the plant Biscutella genus, and assembled and annotated the genome of nine species. They demonstrate a range of features contributing to the divergence in genome structure and time of divergence to transposon content. Specifically, authors identified several chromosomal rearrangements, which can explain the different chromosomal number observed in these genomes. Two merged sub-genomes were found in Biscutella originated by previous hybridisation of two ancestors, characterised by a different gene density. This work significantly expands our understanding related to plant genome evolution, by analysing a genus with significant variation in the chromosome number. Authors were able to reconstruct the genome dynamic in the genus and provide a convincing explanation of the variations observed. The work also provides significant resources in term of novel genome assemblies and annotation of Biscutella genus, which will also contribute to downstream metagenomic studies.
A(authors): We thank the reviewer for their insightful evaluation of our work!

The manuscript is generally well written, and the description of the analysis and results is generally well presented. Nonetheless, the manuscript is difficult to follow in some places, with field-specific abbreviations not given in full or explained before use (see below specific comments). There are few main concerns which will be described below:

R1-Q1: Authors appear to assume that a high S/I ratio can be univocally associated to a higher LTR transposable elements removal rate (figure 4c), but this is true only assuming that transposition rate is constant in time. However, for AURI and DYDY species the LTR TE insertions appear to be generally older compared to other species (figure 4a), suggesting that transposition rates have not been the same in different Biscutella species). So, the higher proportion of solo LTRs found in mostly AURI and DIDY genomes could be simply a consequence of a lower transposition rate, rather than a higher degree of removal? Does this represent an alternative interpretation to the author interpretation?

A: We thank the reviewer for highlighting the importance of transposition rate variation. We concur that differences in transposition rate could, in principle, affect the solo:intact (S/I) LTR ratio, which may complicate its interpretation as a direct proxy of LTR-RT removal rate. To further clarify this point, we performed several additional analyses.

First, although intact LTR-RTs in AURI and DYDY do show slightly older average insertion times (as illustrated in Figure R1.1 below or Supplementary Fig. 18 in the new Supplementary Information), **the insertion times of solo LTRs do not appear to be elevated in these genomes and remain largely comparable across all specie**s. If lower transposition rates were the primary driver of the elevated S/I LTR ratios in AURI/DIDY, we would expect their solo LTRs to also be older due to prolonged degradation periods. However, our results do not support this pattern, suggesting that solo LTR formation continues in these genomes and is not merely a relic of ancient insertion bursts.

Second, to account for potential differences in transposition rates, we calculated the relative ratio of solo LTR and intact LTR accumulation over time, using the following formula:
Relative S/I ratio = (number of solo LTRs / average insertion time of solo LTRs) : (number of intact LTRs / average insertion time of intact LTRs)
This metric provides an estimate of the relative accumulation rate of solo LTRs compared to intact LTRs, normalized by the time available for their formation. **The relative S/I ratios follow a pattern highly consistent with the raw S/I ratios** (as shown in Figure R1.2 below), revealing elevated values in early-diverging species. This reinforces our interpretation that the increased S/I ratio in these species likely reflects more active LTR-RT removal through solo LTR formation, rather than simply lower insertion rates.
We acknowledge that this relative ratio is not commonly used in the literature and may not be broadly familiar. For this reason, we decided to retain the raw S/I ratios in the main text and figures, while providing the relative S/I analysis here to bolster our conclusions.

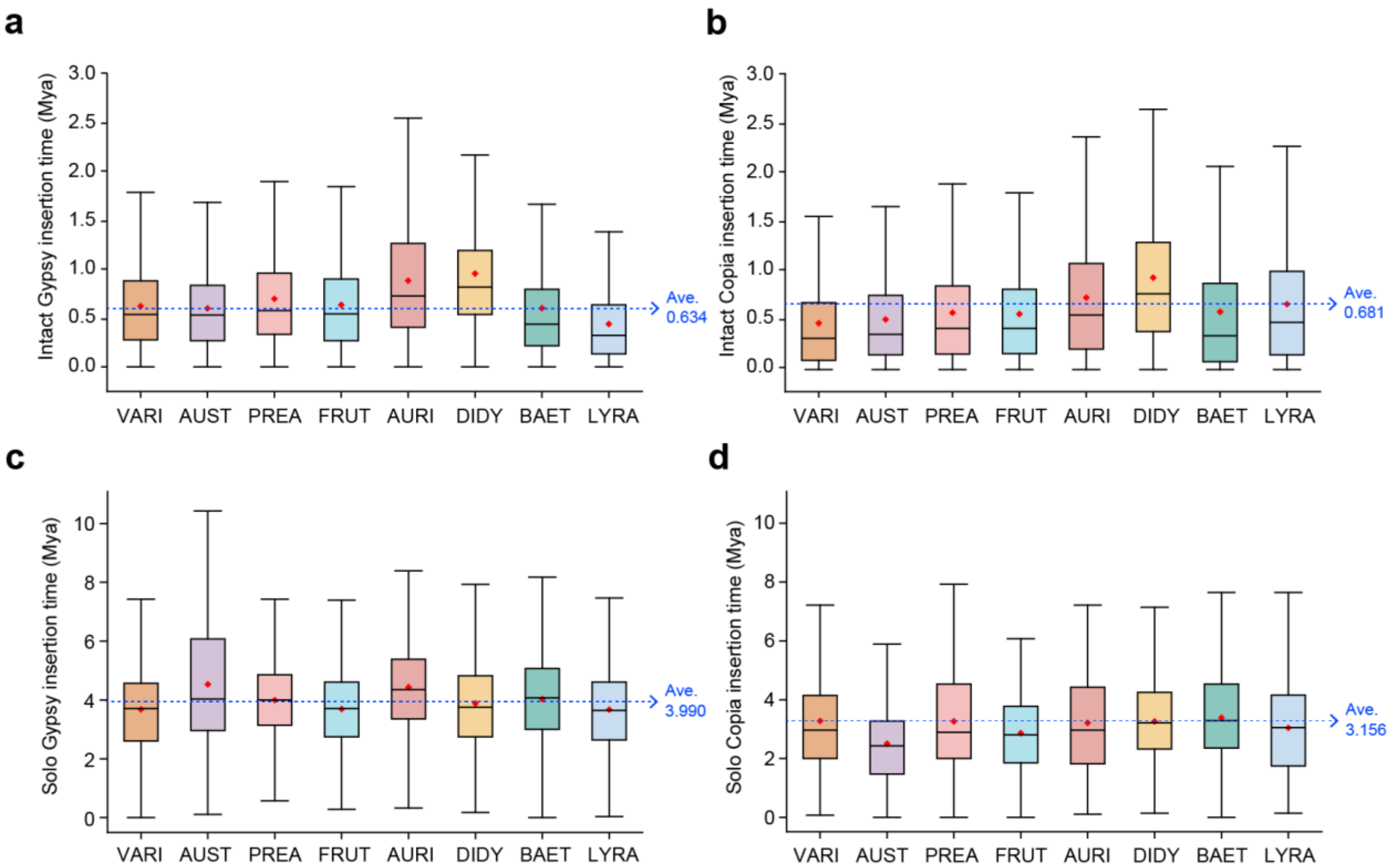

Figure R1.1 (Supplementary Fig. 18). Insertion time distribution of Gypsy- and Copia-type retrotransposons.

(a, b) Insertion time of intact Gypsy (a) and Copia (b) elements, estimated based on the sequence divergence between their two LTRs.

(c, d) Insertion time of solo Gypsy (c) and Copia (d) elements, inferred from sequence divergence between each solo LTR and the consensus LTR sequence of the corresponding family.

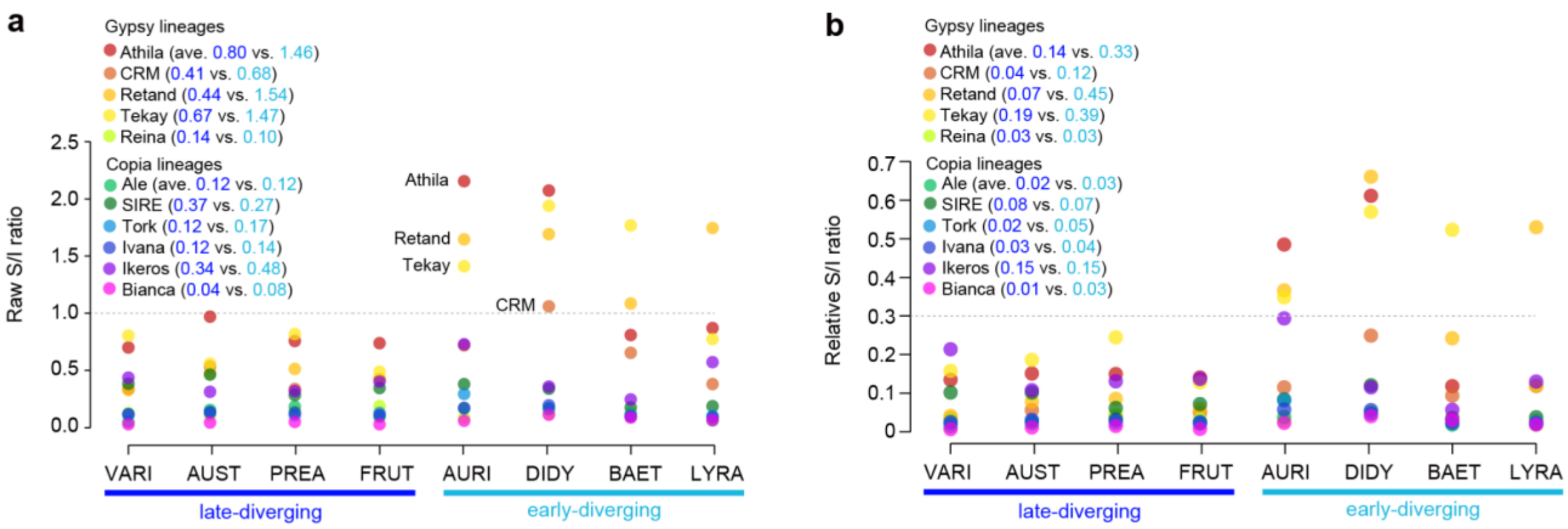

Figure R1.2. Raw (a) and relative (b) solo:intact (S/I) LTR ratios.

The average values of S/I LTR ratio for late-diverging (dark blue) and early-diverging (light blue) species are labelled in the upper left corner.

R1-Q2: Also, at line 270 authors write that: no specific pattern of soloLTR loss was observed between subgenomes;. However, from figure 4d appears that in a couple of LTR families (Retand

and maybe Athila) the S/I ratio is actually quite different among the two subgenomes, for AURI and LYRA species. These families are also the two groups that appear to lead the difference of ratio in early-diverging species. So again, I think this might reflect differential activation rate of these TEs in the different subgenomes for some species, with low S/I ratio being possibly associated to recent transposition bursts?

A: We thank the reviewer for this careful observation. We agree that when examining individual LTR-RT lineages (e.g., Retand), the S/I ratios may show variations between the two subgenomes in certain species, such as AURI and LYRA. This could reflect the dynamics of specific elements within a subgenome to some extent. However, our original intention was to convey that **no statistically significant overall bias was detected between the subgenomes** across all species.

To clarify this point, we have revised the Main Text to state the following:

Original Text: no specific pattern of solo LTR loss was observed between subgenomes

Revised Text (lines 267-268): no significant differences in the S/I ratios were observed for the Ty1/Copia lineages (Fig. 4b) and between the subgenomes (Fig. 4c)

Additionally, we have included the P-value from the Wilcoxon signed-rank test in the revised Fig. 4c.

Furthermore, we also calculated the relative S/I ratios for each subgenome individually (normalized by the average insertion times, as done in R1-Q1), and found that these patterns were highly consistent with the raw S/I ratios (see Figure R1.3 below). This again suggests that while some family- or lineage-specific variation may exist, the overall trend does not indicate a systematic bias in removal dynamics between the subgenomes.

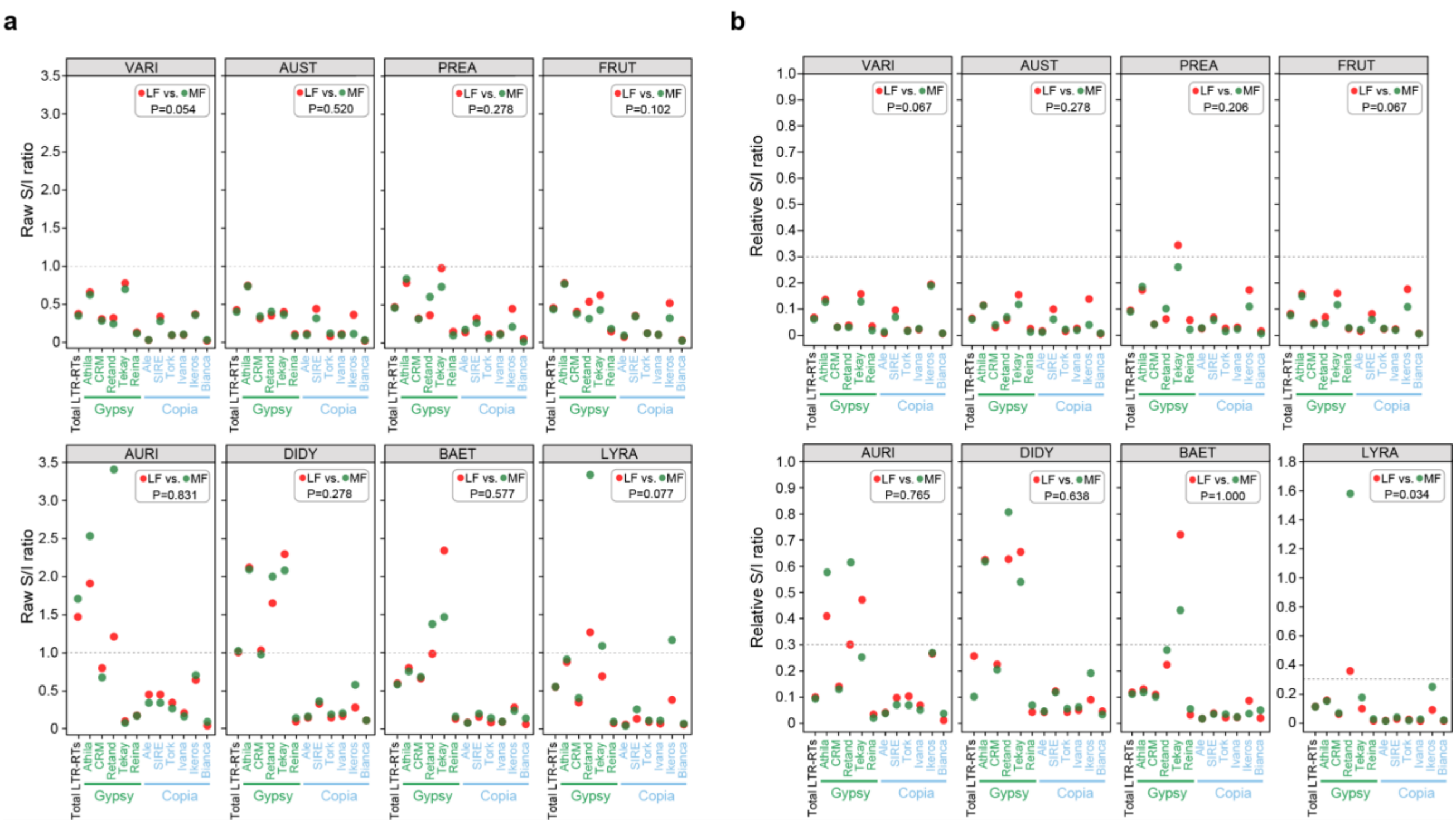

Figure R1.3. **The raw (a) and relative (b) S/I LTR ratios in the subgenomes.** The S/I ratios of total LTR-RTs (first column of the X-axis) and 11 Gypsy or Copia lineages (2nd to 12th column of the X-axis) were analyzed for the LF and MF subgenome, respectively. No significant differences were found between the two subgenomes across the eight species (the Wilcoxon signed-rank test).

R1-Q3: Figure 2a illustrate the difference in term of gene density in the two subgenomes across all species analysed. While in general term it is clear that the trend is different in LF and MF subgenomes, there are exceptions for some blocks. For example, blocks G and T for FRUT and AUST genome appear swapped in the two groups. Also, in block D (and maybe G) for DYDY, BAET and LYRA genomes show the same effect (this is also visible in block O for PREA. Considering that the gene density was used as a parameter to classify the subgenomes, I am wondering why these blocks have been assigned in an apparently inconsistent way. Also, the C block appears to be much more consistent in the difference of gene density and will be probably an outlier compare to both LF and MF averages. Does this reflect the specific genetic composition of this block (e.g. high euchromatin or heterochromatin content)?
A: We thank the reviewer for raising this insightful question. Indeed, although most blocks follow the LF>MF gene density pattern, there are some blocks that display the opposite pattern (MF>LF). This inconsistency can primarily be attributed to **variation in chromosome content**. For example, if a given LF block is located near centromeric or heterochromatic regions (typically gene-poor), while its MF counterpart resides in more gene-rich euchromatic arms, this can result in a locally inverted gene density pattern. For more detailed information, please refer to Figures R1.4 to R1.10.

Regarding the partitioning of subgenomes, we would like to clarify that while gene density is an important parameter, it is not the only criterion used. Our subgenome assignments also rely on:
1. Block associations and structural integrity. For example, if blocks D+E consistently appear together on the same chromosome across different species and show strong syntenic conservation, we infer a shared subgenome origin, regardless of gene density variation in the individual blocks.
2. Phylogenetic evidence. We constructed phylogenetic trees for each block separately (see Supplementary Fig. 10 in the updated Supplementary Information file), and these trees consistently grouped LF and MF blocks into distinct clades. This provides independent evolutionary support for our subgenome phasing.
3. K-mer abundance. K-mer-based hierarchical clustering and PCA (see Supplementary Fig. 11 in the revised Supplementary Information file) revealed a clear separation between the LF and MF subgenomes, further validating our assignments.
Here, we provide further demonstrations and explanations of blocks that exhibit opposite patterns (MF>LF):

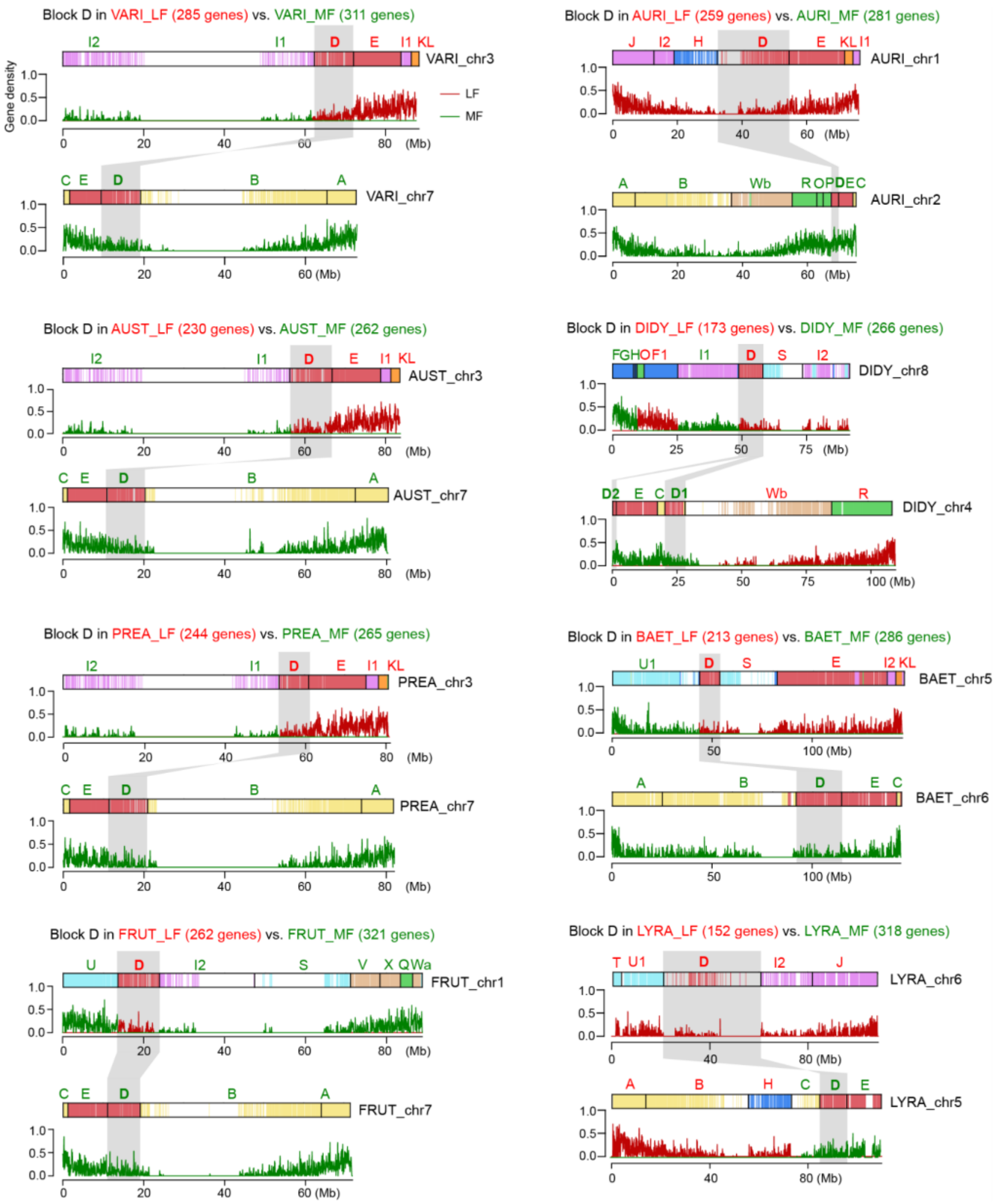

Figure R1.4. Block D shows a higher gene density in the MF subgenome (MF>LF pattern; gray background).

In VARI, AUST, PREA, FRUT, DIDY, BAET and LYRA, both the LF (red) and MF (green) copies of block D are positioned near pericentromeric, gene-poor regions. In AURI, the LF copy of block D lies near the pericentromere, while the MF copy is located in a gene-rich chromosome arm. Therefore, gene density between the subgenomes does not always follow the LF > MF pattern, but is also constrained by the chromosomal context of local gene distribution.

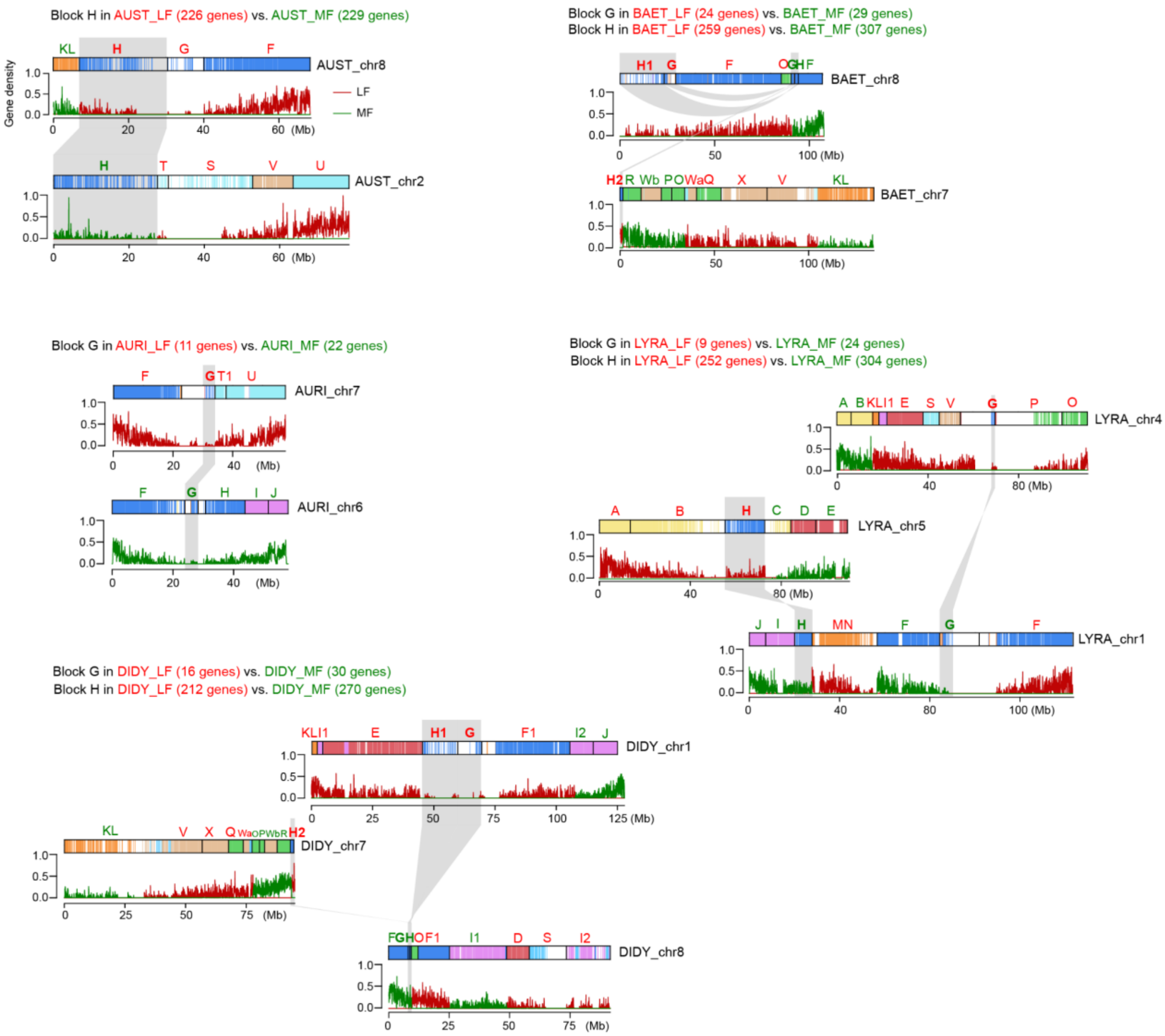

Figure R1.5. Blocks G/H show a higher gene density in the MF subgenome (MF>LF pattern; gray background).

In AUST, AURI and LYRA, both LF (red) and MF (green) copies of blocks G/H are located near pericentromeric, gene-poor regions. In DIDY and BAET, the LF copies of blocks G and H1 (a part of block H) are located near the pericentromere, while their MF counterparts are positioned in gene-rich chromosome arms.

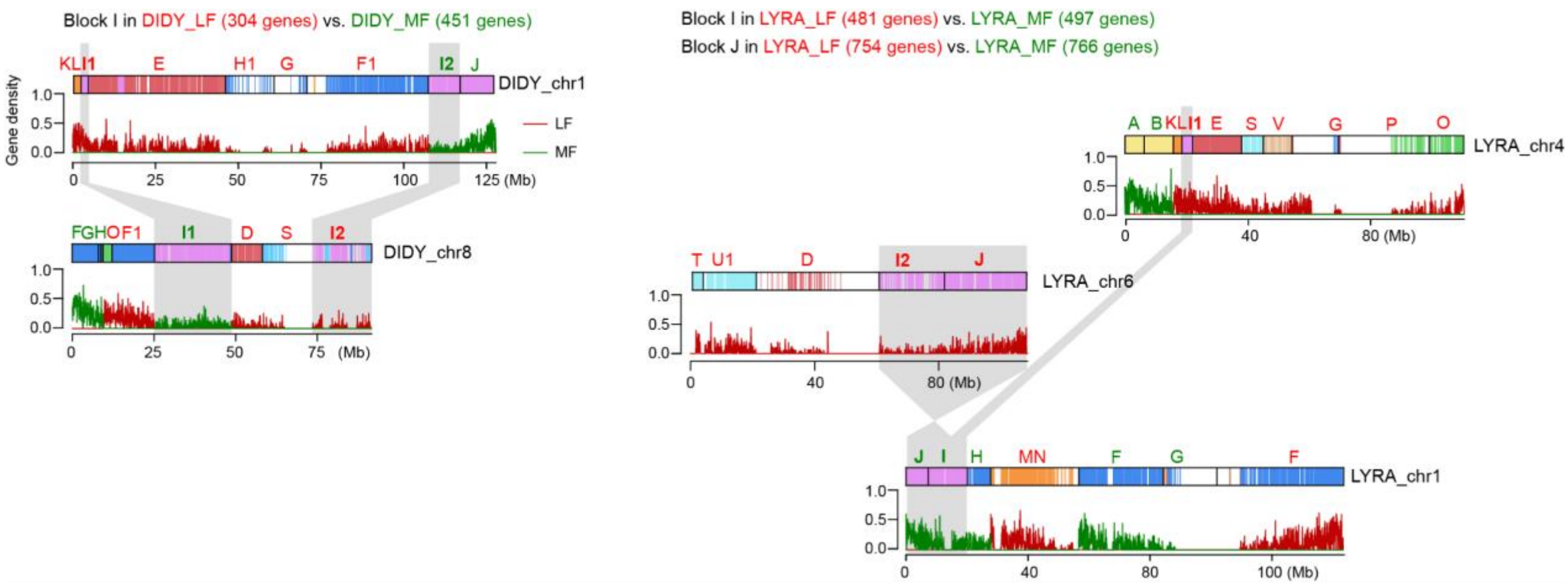

Figure R1.6. Blocks I/J show a higher gene density in the MF subgenome (MF>LF pattern; gray background).

In DIDY, both copies of block I are broken into two parts (I1 and I2), the LF copy of I2 is located on the short arm of an acrocentric chromosome (DIDY_chr8), while the MF copy lies within a gene-rich chromosome arm.

In LYRA, the LF copy of I2+J occupies one chromosome arm, with I2 located near the pericentromeric, gene-poor regions, while the MF copy is positioned in a gene-rich chromosome arm.

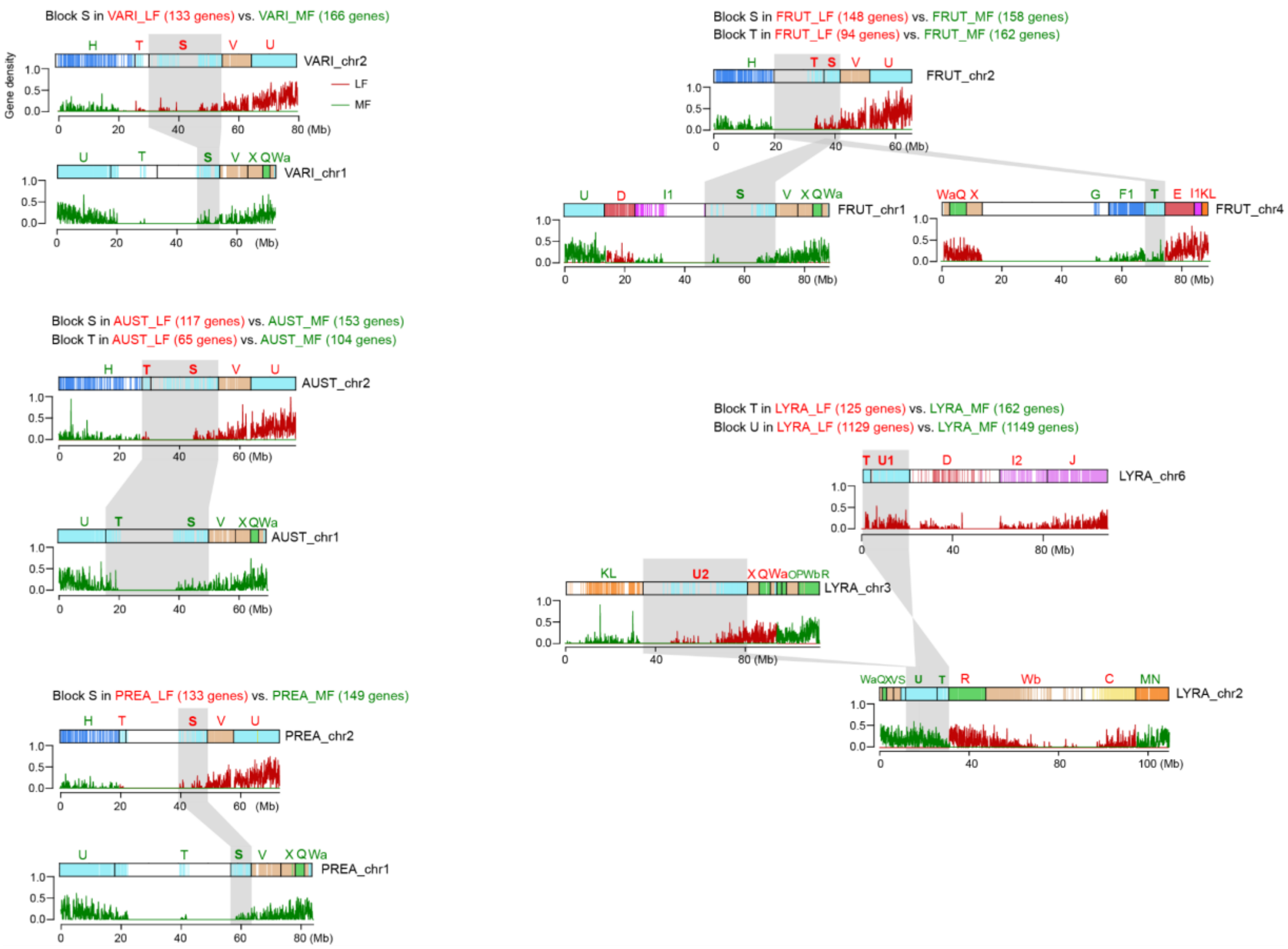

Figure R1.7. Blocks S/T/U show a higher gene density in the MF subgenome (MF>LF pattern; gray background).

In VARI, AUST and PREA, both LF (red) and MF (green) copies of blocks S/T are located near pericentromeric, gene-poor regions.

In FRUT, the LF copy of S/T and the MF copy of S are located near pericentromeric, gene-poor regions, while the MF copy of T is positioned within a gene-rich chromosome arm.

In LYRA, the LF copy of block U is broken into two parts (U1 and U2), the U2 block is located near pericentromeric, gene-poor regions. The LF copy of block U1+T and the MF copy of block T+U are located in a gene-rich chromosome arm.

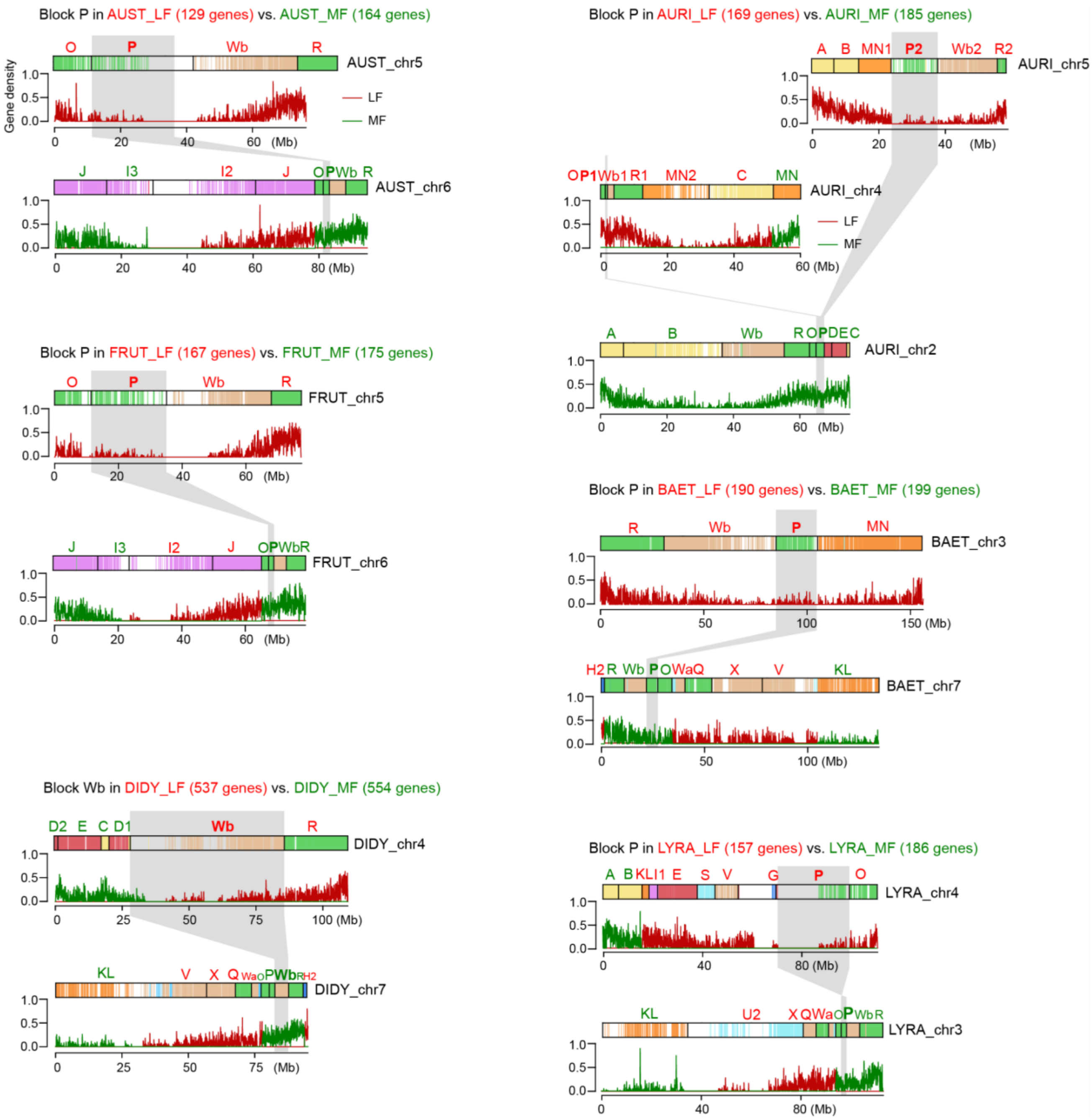

Figure R1.8. Block P/Wb showing higher gene density in the MF subgenome (MF>LF pattern; gray background).
In AUST, FRUT, DIDY and LYRA, the LF copies of block P/Wb are located near pericentromeric, gene-poor regions, while their MF counterparts are positioned in a gene-rich chromosome arm.
In AURI, the LF copy of block P is broken into two parts (P1 and P2). The LF copy of block P2 is located near pericentromeric, gene-poor regions, while the LF copy of block P1 and the MF copy of P are positioned in gene-rich chromosome arms.

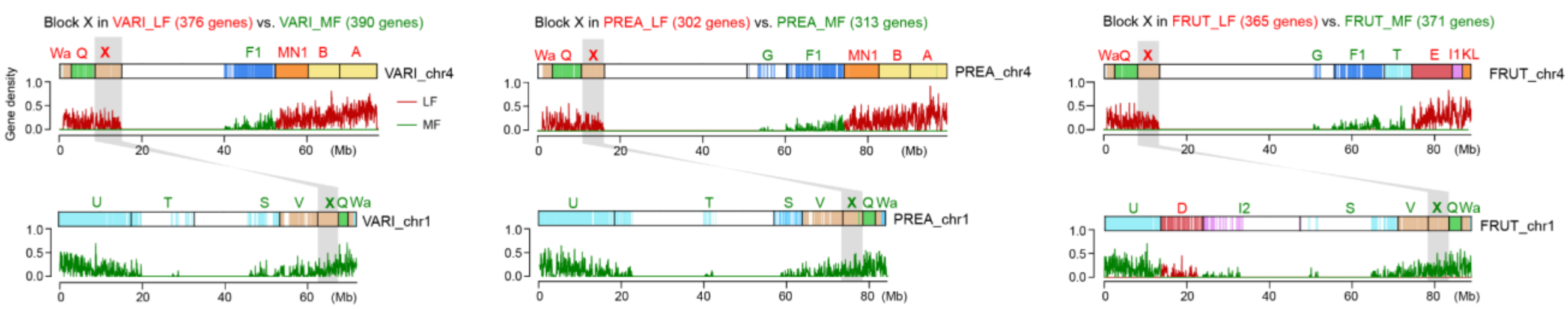

Figure R1.9. Block X shows a higher gene density in the MF subgenome (MF>LF pattern; gray background).
In VARI, PREA and FRUT, the LF copies of block X are located near pericentromeric, gene-poor regions, while their MF counterparts are positioned within gene-rich chromosome arms.

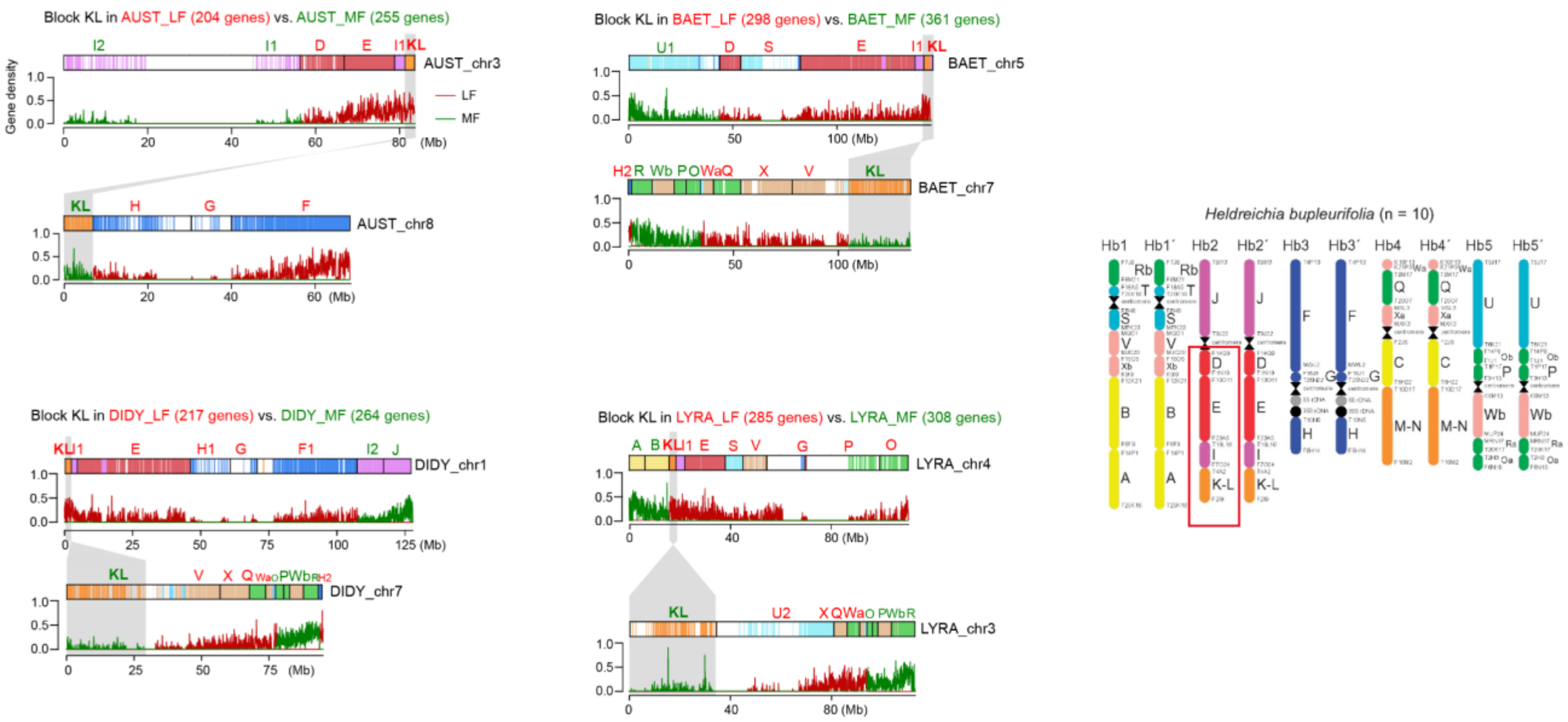

Figure R1.10. Blocks KL show a higher gene density in the MF subgenome (MF>LF pattern; gray background). Both the LF and MF copies of blocks KL are located on chromosome arms. Although the gene densities of the two copies are very similar, which makes it uncertain to phase them solely based on gene density, cytogenetic evidence from *Heldreichia* (a sister genus to the LF clade; Guo et al., 2021, https://doi.org/10.1093/molbev/msaa327) supports the conserved association of KL+I+E, consistent with our current phasing.

R1-Q4: Finally, related to the finding that breakage hotspots (HOTs) are associated to higher density of LTR-TEs. Authors link this finding with a general hypothesis that interspersed stretches of similar TEs may be substrate of recombination and chromosome rearrangements. While this could be true (and it is discussed also in other works), from the analysis made here it is clear that most of genome rearrangements in Biscutella genus are dated much earlier than the average date of transposition of most of LTR families analysed. In other words, it is probable that most of LTR-TE insertions occurred after the chromosome rearrangement events. Therefore, I think it is more likely that HOTs are regions that allow for accumulation of TE insertions, rather than TE accumulation here have caused or facilitated the rearrangement itself. Does the authors have any evidence that the accumulation of TEs at HOT was present at speciation time? Moreover, it appears that authors considered only LTR-TE in this analysis, and it is unclear if DNA elements also play a role in HOTs.
A: Thank you for bringing this to our attention. To address this, we included DNA transposons (DNA-TEs) and Helitrons in our analysis (see Supplementary Fig. 22 in the revised Supplementary Information file) and further examined the insertion times of both LTR- and DNA-TEs. Insertion ages were estimated following the method implemented in EDTA (Ou et al., 2019, https://doi.org/10.1186/s13059-019-1905-y), which is based on sequence divergence from two LTR pairs (for intact LTR-RTs) or consensus sequence for the remaining elements. The results showed that although HOT regions are indeed enriched in both LTR-TEs and DNA-TEs, their estimated insertion times (~5-6 Mya) are significantly younger than the inferred WGD (~11 Mya), and basically comparable to the genome-wide averages (see Figure R1.11 below or Supplementary Fig. 24 in the revised Supplementary Information file). Additionally, we did not observe patterns consistent with the timing of chromosomal rearrangements (CRs). For example, we would expect the CR associated with HOT14 (shared only among the n = 9 species) to be relatively recent, yet the TE insertion times in these regions are still indistinguishable from the genome-wide background. This likely reflects the continuous and recent turnover of TE activity, which can erase traces of ancient insertions and complicate the task of distinguishing ancestral TE signals.

Considering these findings and the methodological limitations, we have revised the Main Text to state the following:
Original Text: This is consistent with previous findings in Arabidopsis and *Orychophragmus* (Jiao & Schneeberger, 2020; Zhang et al., 2023a), suggesting that interspersed stretches of similar TEs may be substrate for NAHR that facilitates the recurrent occurrence of some CRs.
Revised Text (lines 310-312): This is consistent with previous findings in Arabidopsis[39] and *Orychophragmus*[40], suggesting that continuously accumulated TEs may serve as substrates for NAHR (Supplementary Fig. 24).

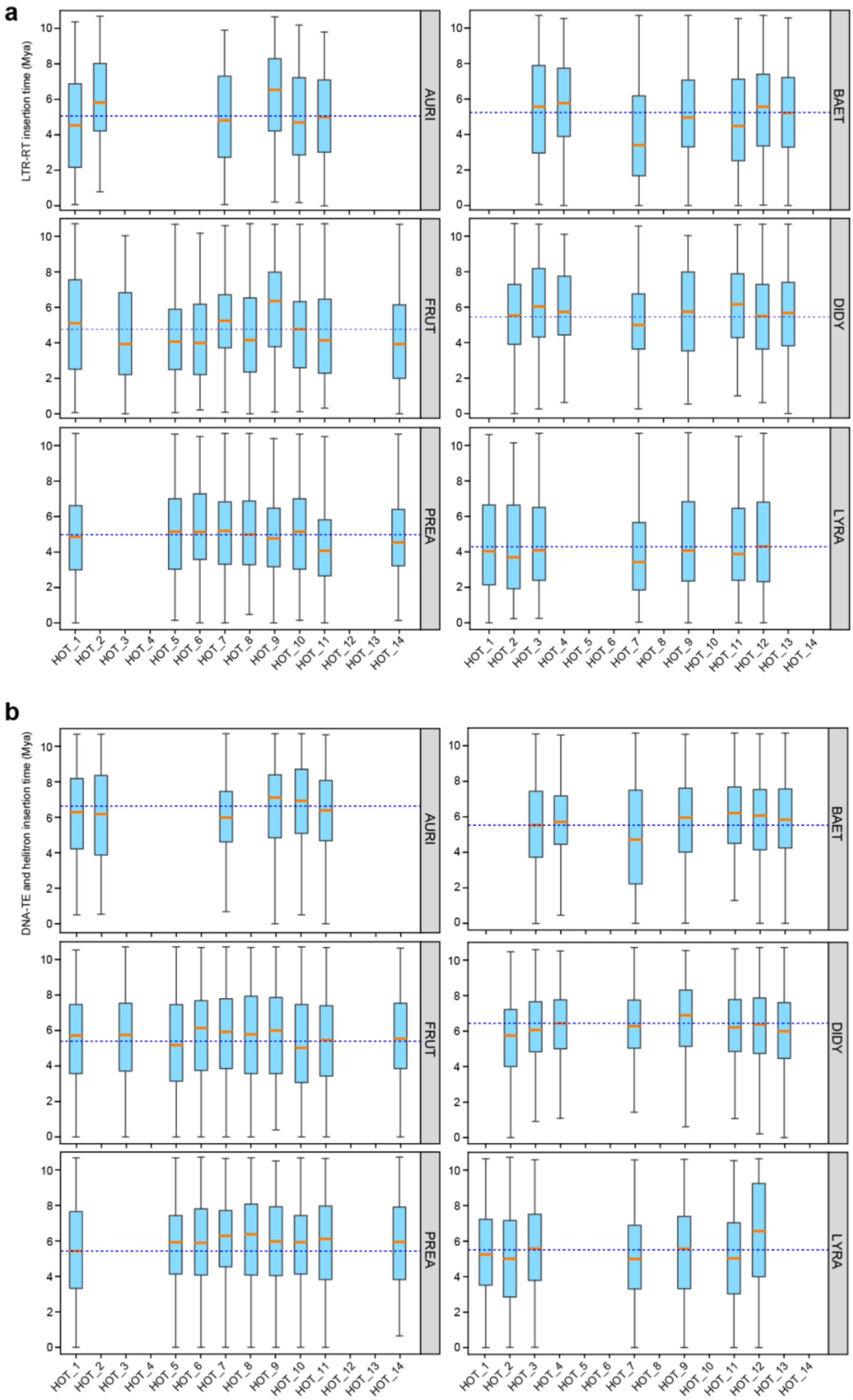

Figure R1.11 (Supplementary Fig. 24). The distribution of insertion times of LTR-RTs (a), DNA transposons and helitrons (b) within the 14 HOTs in six *Biscutella* species. The genome-wide average TE insertion age is indicated by blue dashed lines. Insertion ages were estimated using the method implemented in EDTA (Ou et al., 2019), based on the sequence divergence between LTR pairs for intact LTR-RTs or relative to a consensus sequence for other transposable elements.

Minor comments:
Introduction
R1-Q5: Line 94: there is no reference given for the *B. baetica* studies
A: Thank you for pointing this out. We have added the references accordingly (see below). Actually, the studies by Mandáková et al. (2017) (https://doi.org/10.1111/tpj.14065) and Guo et al. (2021) (https://doi.org/10.1093/molbev/msaa327) cover both *B. baetica* and *B. lyrata.*

Original Text: Much later, however, transcriptomic and cytogenomic studies of the diploid species *B. laevigata* subsp. *varia* (Dumort.) Rouy & Foucaud (2*n* = 18; Geiser et al., 2016), *B. baetica* Boiss. & Reuter (2*n* = 16) and *B. lyrata* (2*n* = 12; Mandáková et al., 2017a) indicated that these species underwent a common WGD event.
Revised Text (lines 91-94): Much later, however, transcriptomic and cytogenomic studies of the diploid species *B. laevigata* subsp. *varia* (Dumort.) Rouy & Foucaud (2*n* = 18)[25], *B. baetica* Boiss. & Reuter (2*n* = 16)[26,27] and *B. lyrata* (2*n* = 12)[26,27] indicated that these species underwent a common WGD event.

25. Geiser, C., Mandáková, T., Arrigo, N., Lysak, M. A. & Parisod, C. Repeated whole-genome duplication, karyotype reshuffling, and biased retention of stress-responding genes in Buckler mustard. *Plant Cell* **28**, 17-27 (2016).
26. Mandáková, T., Li, Z., Barker, M. S. & Lysak, M. A. Diverse genome organization following 13 independent mesopolyploid events in Brassicaceae contrasts with convergent patterns of gene retention. *Plant J.* **91**, 3-21 (2017).
27. Guo, X., Mandáková, T., Trachtová, K., Özüdoğru, B., Liu, J. & Lysak, M. A. Linked by ancestral bonds: multiple whole-genome duplications and reticulate evolution in a Brassicaceae tribe. *Mol. Biol. Evol.* **38**, 1695-1714 (2021).

R1-Q6: Line 102: “eight *Biscutella* species from morphologically and phylogenetically distinct infrageneric clades;”. I think it would be helpful to provide some sort of measure or phylogenetic tree/analysis demonstrating that these are species are phylogenetically distinct. This would help visualise the evolutionary links between the chosen species
A: Thank you for your suggestion. The original statement was based on the morphological and phylogenetic surveys of *Biscutella* conducted by Vicente et al. (2020; https://doi.org/10.3417/2020554) and Alonso et al. (2020; https://doi.org/10.1016/j.ympev.2019.106644). We have now added the corresponding references. However, these two studies did not include *n* = 9 species analyzed in this study. To address this, we extracted the corresponding chloroplast gene sequences and constructed phylogenetic trees including a broader set of species (*n* = 9, 8, and 6). The resulting phylogenies are provided in Supplementary Fig. 2 in the revised Supplementary Information file, enabling visualization of the evolutionary relationships among the selected species.

Results
R1-Q7: First paragraph: Authors state Illumina short-read sequencing has also been used but it is not clear how this has been applied. In the methods it says this was used for chloroplast genome

assembly but I could not find this in the results. Maybe for this first paragraph, you should omit the use of Illumina as I was waiting to read about it in the section but it did not appear.

A: We thank the reviewer for pointing this out. In fact, Illumina data were used in three key steps: (1) estimation of genome size and heterozygosity using *K*-mer frequency analysis (genome survey), (2) polishing Nanopore-based genome assemblies, and (3) assembling chloroplast genomes. We have revised the Methods section to clarify these applications:

Original Text: Genome sizes of *Biscutella* species were further confirmed by *K*-mer frequency (*K* = 21) analysis with the findGSE (v1.94; Sun et al., 2018), after counting 21-mers with Jellyfish (Marcais & Kingsford, 2011).

Revised Text (lines 543-546): In addition, using ~74.2 Gb of Illumina short-read data, genome sizes of *Biscutella* species were further confirmed by K-mer frequency (K = 21) analysis with the findGSE (v1.94)[68], The 21-mers were counted with Jellyfish (v2.2.10)[69]. The estimated genome sizes and heterozygosity are summarized in Supplementary Table 2.

Original Text: Initial assemblies were generated from Nanopore long reads using NextDenovo (v2.2; https://github.com/Nextomics/NextDenovo) with its standard pipeline, and the resulting contigs were polished in two iterative rounds with both long and short reads using NextPolish (v1.1.0; Hu et al., 2020).

Revised Text (lines 549-553): Initial assemblies were generated from Nanopore long reads using NextDenovo (v2.2; https://github.com/Nextomics/NextDenovo) with default parameters. The resulting contigs were further polished in two iterative rounds using both Nanopore long reads and Illumina short reads with NextPolish (v1.1.0)[71].

R1-Q8: Line 203-228: Multiple abbreviations are given in this section without previous explanation e.g. ACK, ancPCK, V+Wa+Q+X, etc. This makes this section difficult to read and is not accessible to those not familiar with the field and terminology. Are these names of the subgenomes?

A: We agree with the reviewer and made revisions to the Main Text to enhance clarity and accessibility. Here are some additional clarifications:

1. Genomic Blocks: The boundaries of 24 conserved genomic blocks (GBs) were identified based on cross-species *in situ* hybridization of chromosome-specific BAC contigs of the model species *Arabidopsis thaliana* and comparative genetic mapping between *A. thaliana* and other crucifer species (Schranz et al., 2006; https://doi.org/10.1016/j.tplants.2006.09.002); a later revision reduced the number of GBs to 22 (Lysak et al., 2016; https://doi.org/10.1016/j.pbi.2016.02.001). The 22 GBs are abbreviated as capital letters A to X; GB associations, such as V+Wa+Q+X, refer to the physical connections/positions of these GBs on chromosomes.

2. Ancestral karyotypes: We refer to several inferred ancestral karyotypes in the Brassicaceae family:

- Ancestral Crucifer Karyotype (ACK), proposed by Schranz et al. (2006), consists of eight chromosomes and initially represented the putative ancestral genome of the Camelinodae supertribe (Lineage I).

- Ancestral Proto-Calepineae Karyotype (ancPCK) was proposed for the Biscutelleae clade (Geiser et al., 2016; Mandáková et al., 2018; Guo et al., 2021), differs from ACK by a single reciprocal translocation.
- Proto-Calepineae Karyotype (PCK; Mandáková and Lysak, 2008) is considered the ancestral karyotype of Brassicodae (Lineage II).

The full names of these karyotypes are also provided in the revised Main Text.

Original Text: The top-down approach included comparing frequent GB associations in *Biscutella* species with those in the known ancestral karyotypes of Brassicaceae (ACK, ancPCK, and PCK; Schranz et al., 2006; Mandáková & Lysak, 2008; Geiser et al., 2016; Lysak et al., 2016).
Revised Text (lines 205-208): The top-down approach included comparing frequent GB associations in Biscutella species with those in the known ancestral karyotypes of Brassicaceae, including Ancestral Crucifer Karyotype (ACK)[31,32], ancestral Proto-Calepineae Karyotype (ancPCK)[25] and Proto-Calepineae Karyotype (PCK)[34].

25. Geiser, C., Mandáková, T., Arrigo, N., Lysak, M. A. & Parisod, C. Repeated whole-genome duplication, karyotype reshuffling, and biased retention of stress-responding genes in Buckler mustard. *Plant Cell* **28**, 17-27 (2016).
31. Schranz, M. E., Lysak, M. A. & Mitchell-Olds, T. The ABC's of comparative genomics in the Brassicaceae: building blocks of crucifer genomes. *Trends Plant Sci.* **11**, 535–542 (2006).
32. Lysak, M.A., Mandáková, T. & Schranz, M.E. Comparative paleogenomics of crucifers: ancestral genomic blocks revisited. *Curr. Opin. Plant Biol.* **30**, 108–115 (2016).
34. Mandáková, T. & Lysak, M. A. Chromosomal phylogeny and karyotype evolution in x = 7 crucifer species (Brassicaceae). *Plant Cell* **20**, 2559-2570 (2008).

3. The two subgenomes identified in all *Biscutella* genomes were classified as **the less fractionated (LF) and more fractionated (MF) subgenomes**, following established nomenclature in *Brassica* (Wang et al., 2011; https://doi.org/10.1038/ng.919).

R1-Q9: Line 246-249: Should Tsuneq be Tuneq as defined in line 233? Are these two different things?
A: Thank you for pointing this out! We realized that we incorrectly abbreviated "Tuneq" and "Tsuneq" to differentiate between singular and plural forms. To clarify, we reviewed the literature. In human studies, such translocations have been described as "unbalanced reciprocal translocations" (Duckett et al., 1984 https://doi.org/10.1007/BF00272991; Chang et al., 2013 https://doi.org/10.1016/j.jcma.2012.11.002). Schubert and Lysak (2011; https://doi.org/10.1016/j.tig.2011.03.004) referred to these translocations as "non-reciprocal translocations". To avoid any confusion, we have updated all instances of "unequal reciprocal translocations" in the Main Text to "unbalanced reciprocal translocations", abbreviated as "unbalanced-RT", in line with the traditional nomenclature used in human studies.

R1-Q10: Line 257: what are you defining as an intact LTR-RT and how did you identify these?

A: Intact LTR-RTs were identified using LTR_retriever (Ou & Jiang, 2018; https://github.com/oushujun/LTR_retriever), which integrates predictions from both LTRharvest and LTR_FINDER. According to the structural criteria described by Ou et al. (Ou & Jiang, 2018, https://doi.org/10.1104/pp.17.01310), an intact LTR-RT contains: 5’ and 3’ LTRs, a pair of dinucleotide palindromic motifs flanking each LTR, an internal region including protein-coding sequences for gag, pol, and env, and a 5-bp target site duplication (TSD) flanking the element. We have added this definition and the details of the identification process to the Methods section for clarity (lines 613-617).

R1-Q11: Line 311: “67.5% of these regions had higher LTR-RT content than the genome-wide average mainly contributed by Athila and CRM retroelements (Figure 5c)”; There is no presentation of the genome wide average, this would be helpful to include to aid comparison and give context for the result

A: Thank you for your suggestion. The genome-wide average of LTR-RT content was calculated using the same approach as for HOT regions, i.e., the number of LTR-RTs divided by the total region length, representing the content of these elements. To facilitate direct comparison, we have now added a new table (Supplementary Table 11) reporting the genome-wide average values.

R1-Q12: Line 344: “the late-diverging species had significantly higher Ka/Ks ratios (Figures 6e and 6f)”. There is no significant difference between early and late diverging in 6f

A: The figure reference has now been fixed.

Original Text: Moreover, TE accumulation flanking retained genes was comparable between late- and early-diverging species, yet the late-diverging species had significantly higher Ka/Ks ratios (Figures 6e and 6f), indicating more relaxed purifying selection.

Revised Text (lines 340-343): Moreover, TE accumulation flanking retained genes was comparable between late- and early-diverging species (Fig. 6e), yet the late-diverging species had significantly higher Ka/Ks ratios (Fig. 6f), indicating more relaxed purifying selection.

Discussion

R1-Q13: The discussion is very short and fails to discuss some of interesting findings from this paper e.g. the differences in retention of the ABCDE flowering genes, the possible contributions of TEs to genome evolution and divergence etc. It also ends very abruptly with no discussion on the evolutionary significance of the data.

A: Thank you for your opinion. In the previous version, the Discussion section was relatively short due to word count limitations. In the revised manuscript, we have substantially expanded and refined this section. The updated Discussion now covers several key aspects, including: the origin of the *Biscutella* subgenome (second paragraph); the mechanisms and rates of descending dysploidy (third paragraph); the potential relationship between chromosome breakage and its three-dimensional chromatin architecture (fourth paragraph); and the role of the diploidization in the emergence or stabilization of fruit morphological diversity within the Biscutelleae (fifth paragraph). We believe that these additions provide a more comprehensive interpretation of our findings.

Methods and Figures

R1-Q14: Were any biological replicates used?

A: No biological replicates were used for whole-genome sequencing. For each species, genome assemblies were generated from DNA extracted from a single individual, which was deliberately chosen to minimize heterozygosity. This approach follows the standard practice in plant genome projects (e.g., the recently sequenced bread wheat, Liu et al., 2025, https://doi.org/10.1038/s41588-025-02137-x; T2T cotton genome, Huang et al., 2024, https://doi.org/10.1038/s41588-024-01877-6) where the primary goal is to obtain a representative haploid assembly rather than to assess individual variations.

R1-Q15: What weight of tissue was used for Illumina, Hi-C, and RNAseq?

A: Thank you. For Illumina sequencing, genomic DNA was extracted from ~50 mg of young leaves using the NucleoSpin Plant II kit. For Hi-C libraries, 1 g of leaf tissue was used for the Proximo Hi-C protocol, and 300 mg was used for the Omni-C Proximity Ligation Assay. For RNA sequencing, total RNA was isolated from ~50 mg of leaves and flowers, and ~100 mg of roots. These details have now been included into the revised Main Text (lines 498-499, 504, 517, and 525).

R1-Q16: Line 458: what do you mean by young leaf? What age were the plants?

A: For genomic DNA extraction and Hi-C experiments, leaf samples were repeatedly collected from young, green, and undamaged leaves from plants at the pre-reproductive stage. Tissues for RNA extraction were harvested from blooming plants. These details have now been included in the revised Main Text (lines 498-499).

R1-Q17: In the pdf version of the manuscript formulas are not printed correctly (e.g. line 648, line 721).

A: This problem is related to the submission system. We will try to resolve it when submitting a new manuscript version. If it still fails, we will communicate with the editor how to solve it.

R1-Q18: Figure 4C: I do not think it is correct to present this as continuous data. It suggests you are tracking a change when you are noting a difference between species. A heading for each of the Gypsy and Copia lineages would also be helpful for understanding the figure immediately

A: Thank you. The figure has been modified based on your suggestions.

R1-Q19: Figure 5a: you indicate HOTs shared within clade I or II with the same arrow colour. Can you separate out and provide a different arrow for each? This currently does not provide information on the HOTs identified that may contribute to clade divergence.

A: Thank you for this suggestion. The revised Fig. 5a has labelled breakpoints in different colors.

Reviewer #2 (Remarks to the Author):

The study provides a comprehensive analysis of post-polyploid genome evolution in Biscutella species, emphasizing descending dysploidy and chromosomal rearrangements after whole-genome duplication. Its focus on the recurrent, independent chromosome reductions and the underlying genomic features, such as LTR retrotransposons and 3D genome architecture, significantly advances our understanding of karyotype evolution in Brassicaceae. The integration of high-quality genome assemblies with evolutionary inference adds valuable insights that are currently underrepresented in the literature. Overall, the work addresses a notable gap concerning the mechanistic basis of descending dysploidy, especially in a comparative, multi-species context. However, several critical issues must be addressed before the manuscript is suitable for publication in Nature Communications.

A(authors): We sincerely appreciate your positive feedback and perception of our work.

Major Comments

R2-Q1: The study offers valuable insight into independent versus convergent diploidization pathways within a single genus. However, similar analyses have been reported in other Brassicaceae, for example, Brassica spp. (Lysak et al., 2007 https://pmc.ncbi.nlm.nih.gov/articles/PMC2048728/ ; Mandakova; & Lysak, 2018 https://pubmed.ncbi.nlm.nih.gov/29567623/) and in cotton (Wang et al., 2021 https://academic.oup.com/mbe/article/38/9/3621/6262643). The authors should more clearly articulate what novel principles emerge specifically from Biscutella that were not apparent in those systems.

A: Thank you for mentioning these publications. However, we are not aware of any similarly extensive work that has been published for any genus within the Brassicaceae family, including *Brassica*. The main difference compared to our previously published papers is that we focus on whole-genome assemblies and the analysis of actual DNA sequences. This level of detail is not achievable through comparative cytogenomics, which is limited to analyzing and interpreting only a few parameters and phenomena.

Through further investigation of the literature, we believe that the genus *Biscutella* is one of the most comprehensive examples of post-polyploid diploidization within the Brassicaceae family. Other genera also show evidence of these processes, but the comprehensive data within a single genus like *Biscutella* is particularly valuable. We have added a highlighted sentence at the end of the Introduction section (lines 107-108).

R2-Q2: The identification of 12 breakage hotspots enriched in LTR-RTs and overlapping TAD boundaries is intriguing, but the causal link between TE content, 3D architecture, and recurrent rearrangements remains correlative. Could the authors provide any functional assays (e.g., cytological evidence of DSB hotspots or chromatin accessibility data) to strengthen this claim?

A: We appreciate the reviewer's insightful comment. We concur that our current evidence indicates a correlative rather than a causal link between recurrent rearrangements, TE enrichment, and 3D

genome architecture. To enhance the robustness of our findings, we conducted additional simulations using 200 randomly selected genomic regions. These simulations consistently demonstrated that the observed genomic features of the hotspot regions are highly unlikely to occur by chance (see Supplementary Figs. 21, 23, 25, and 26 in the updated Supplementary Information). These analyses further confirm the non-random nature of the identified breakage hotspots.

We also explored potential methods for cytological validation of breakpoint hotspots. However, due to the significant age of the lineages we studied, it is unlikely that ancient DSB footprints can be identified using FISH. Based on our 25 years of experience with FISH and comparative chromosome painting, detecting a 100-kb probe on extended pachytene chromosomes is already a challenge. Moreover, our extensive experience with various plant genomes, has shown that it is not trivial (and sometimes impossible) to specifically localize different types of LTR retrotransposons by FISH. Given these constraints, we decided to concentrate on computational and comparative genomic evidence. In the revised manuscript, we clarified that our conclusions focus on the correlation between DSB-prone regions and the underlying genomic features. We also emphasize that establishing causal relationships will require future experimental studies.

R2-Q3: Eight species spanning three chromosome numbers is a solid start, but the phylogenetic sampling is uneven: the n = 9 clade is represented by four species, whereas the n = 6/8 clade includes only three plus B. auriculata. Given the young divergence of the late-diverging clade (~3.3 Mya), are four genomes sufficient to capture its variability? Conversely, should the authors have included more early-diverging taxa (e.g., additional outgroups) to more robustly infer ancestral karyotypes?
A: We thank the reviewer for addressing the issue of phylogenetic sampling and the representation of chromosome number diversity within *Biscutella*.

First, it is important to highlight that **the distribution of chromosome numbers in *Biscutella* is inherently uneven**. Most species have *n* = 9 (~40 species), a smaller number have *n* = 8 (~10 species), and ONLY a single species has *n* = 6 (*B. lyrata*). Our study comprehensively includes all known chromosome numbers within the genus, thereby capturing the full extant of chromosome number diversity in *Biscutella*. We acknowledge that, particularly among early-diverging species, it is (very) likely that species sharing the same chromosome number could differ in their underlying genomic structures. However, due to constraints related to sequencing costs and time, we were unable to obtain chromosome-scale assemblies for additional extant species. As a result, we selected representative species that capture the full known diversity of chromosome numbers and phylogenetic variation within *Biscutella*.

Second, we constructed a plastome-based phylogeny (see Supplementary Fig. 2 in the revised Supplementary Information file) incorporating a broader range of species beyond those with whole-genome data. The chloroplast genomes of the species we studied were newly assembled, and we

obtained additional plastomes from Vicente et al. (2020; https://doi.org/10.3417/2020554). This phylogeny supports the classification of early- and late-diverging species presented in our manuscript.

As for the outgroup selection, the tribe Biscutelleae comprises five genera: *Biscutella*, *Megadenia*, *Heldreichia*, *Lunaria*, and *Ricotia*. Our study includes representatives from all these genera. For *Megadenia*, both the chromosome-scale genome assembly and cytogenomic data are available and were included. While *Heldreichia*, *Lunaria*, and *Ricotia* do not have genome assemblies, we have incorporated available cytogenomic data for these genera. **Thus, our inference of the ancestral *Biscutella* karyotype integrates all currently available genomic and cytogenomic information for the tribe**. Furthermore, our conclusions align closely with those of Guo et al. (2021; https://doi.org/10.1093/molbev/msaa327), who also inferred ancestral karyotypes using transcriptomic and cytogenomic data. Overall, we believe that our sampling strategy and integrative approach offer a robust framework for reconstructing ancestral karyotypes and capturing chromosomal variation within *Biscutella* and its relatives.

R2-Q4: The coalescent analyses (ASTRAL-Pro) show substantial gene-tree discordance. The manuscript would benefit from quantitative measures (e.g., proportion of quartets or gene trees supporting key nodes) and discussion of how this uncertainty impacts the reconstruction of the ancestral genome.

A: We appreciate the reviewer's valuable suggestion. In our manuscript, we have clarified that while the ASTRAL-Pro analyses based on 1,234 syntenic gene trees indicated some discordance, these conflicts are highly localized. Specifically, they are concentrated at the basal node of the Biscutelleae clade (which includes non-*Biscutella* species, such as *Megadenia*, *Lunaria*, and *Ricotia*; these species do not share a common ancestor with *Biscutella*). Additionally, the placement of *B. auriculata* shows both concordant and discordant gene trees: in the LF subgenome, there are 430 concordant versus 804 discordant trees, and in the MF subgenome, there are 376 concordant versus 858 discordant trees, indicating a moderate level of discordance. However, the relationships among the other *Biscutella* species are strongly supported, with $n = 9$ species forming a well-resolved clade, and *B. baetica*, *B. didyma*, and *B. lyrata* forming another distinct clade (as shown in Supplementary Fig. 10 of the revised Supplementary Information). The placement of *B. auriculata* is further corroborated by independent chloroplast phylogenies (see Supplementary Fig. 2 in the revised Supplementary Information file) and by conserved genomic block associations in the dominant LF subgenome, which consistently align *B. auriculata* with the $n = 9$ species clade (see Supplementary Fig. 13 in the revised Supplementary Information).

We agree that such topological discordance can potentially affect local nodes in the reconstruction of ancestral genomes. In our case, the uncertainty is only restricted to the presence or absence of node A4 (Fig. 3b), i.e., whether the $n = 9$ clade and *B. auriculata* diverged sequentially or more directly from the ancestor (both scenarios are feasible). However, the reconstruction of the ancestral *Biscutella* karyotype ($n = 14$) remains robust, supported by three independent lines of

evidence: (1) pairwise bottom-up genome comparisons that incorporate phylogenetic context; (2) top-down inference based on conserved genomic block associations (independent of phylogenetic relationships); and (3) congruence with earlier cytogenetic and transcriptome-based studies (Guo et al, 2021, https://doi.org/10.1093/molbev/msaa327; Mandáková et al., 2018, https://doi.org/10.1111/tpj.14065; Geiser et al., 2015, https://doi.org/10.1105/tpc.15.00791). Taken together, these results provide confidence that the observed gene-tree discordance does not compromise the key conclusions about the structure of the ancestral genome.

R2-Q5: Table S2 reports contig N50 = 2.9 Mb for ONT assemblies versus 22.9 Mb for the PacBio B. lyrata genome. The methods should specify whether any haplotigs or alternative haplotypes were purged, and what BUSCO duplications (if any) indicate residual haplotype duplication.
A: Thank you for your comment. We employed the Khaper program to remove redundant contigs, with specific parameters further detailed in the Methods section. We evaluated the performance of the Khaper program using BUSCO tests. For example, in *B. baetica*, the original BUSCO duplication score of raw assembly was 31.6%; after applying Khaper, the BUSCO duplication score was 30.5%; in *B. auriculata,* the original BUSCO duplication score of raw assembly was 19.6%; after applying Khaper, the BUSCO duplication score was 17.3%. For the high-fidelity assembly of *B. lyrata*, hifiasm was run in Hi-C Integrated mode. Downstream analyses employed the primary assembly (p_ctg). Further details are also described in the Methods section (lines 553-558).

R2-Q6: "Solo LTR identification via the novel soloLTRseeker"; pipeline is briefly described, but no benchmarking is provided against established tools (e.g., LTR_retriever: soloLTR calls). Moreover, the mutation rate (r = 1.5e-7) assumed for insertion dating should be justified for Biscutella specifically, as rates can vary substantially across lineages.
A: We thank the reviewer for giving us the opportunity to discuss the topic of soloLTR annotation, and explain why we decided to develop the soloLTRseeker pipeline (https://github.com/estpr/soloLTRseeker) and apply it to the *Biscutella* genomes. It is relatively common in genome-wide studies that analyse transposon activity to identify soloLTRs, when the aim is to calculate and compare the dynamics of TE deletion between species. This is a topic of great interest in the lab of co-authors Perez-Roman/Bousios, so the last few years we have been looking at methods that annotate soloLTRs and what approaches previous papers have used. To our surprise, there is no dedicated tool out there that has been rigorously tested and developed into a user-friendly pipeline that provides running parameters to suit different projects and detailed output files that can help downstream analyses. This is reflected in the literature, where studies have used in-house simple pipelines or not fully-developed methodologies passed down from previous papers (e.g., Cossu et al., 2017, https://doi.org/10.1093/gbe/evx260; Wan et al., 2018, https://doi.org/10.1038/s41477-017-0097-2; Wan et al., 2021, https://doi.org/10.1038/s41467-021-24528-4; Niu et al., 2022, https://doi.org/10.1016/j.cell.2021.12.006). LTR_retriever is an exceptionally highly-cited tool (that is mostly run as part of EDTA) and indeed has scripts for soloLTR detection, but these do not appear as an independent pipeline that the user can easily access and install, while all parameters are hardcoded in the scripts. More importantly, the soloLTR

pipeline has not been tested in the LTR_retriever paper (Ou & Jiang, 2018, https://doi.org/10.1104/pp.17.01310).

Overall, the lack of detailed information about the available soloLTR detection methods and in their output files is the reason why there is inconsistency and ad-hoc approaches in the literature. We believe that in some of these cases there is a large number of false positives, where fragments of TEs are annotated as soloLTRs. This is why we decided a year ago to design a dedicated pipeline, soloLTRseeker, rigorously test it including simulations, and make it available to the community. The pipeline is available in Github, and we are currently preparing the publication. For the *Biscutella* genomes, at the request of the reviewer, we compared soloLTRseeker and LTR_retriever in the *B. lyrata* genome. The results of our analysis are shown in the Figure R2.1 below. Generally, LTR_retriever annotates a much higher number of soloLTRs than soloLTRseeker, but our data suggest that there is a lot of noise in these annotations with a potentially high number of LTR retrotransposon fragments and very short hits included.

Taken together, we hope we have convinced the reviewer why a dedicated soloLTR annotation pipeline will be a valuable addition to the annotation toolbox of genome-wide studies, and that soloLTRseeker generates more reliable annotation than LTR-retriever.

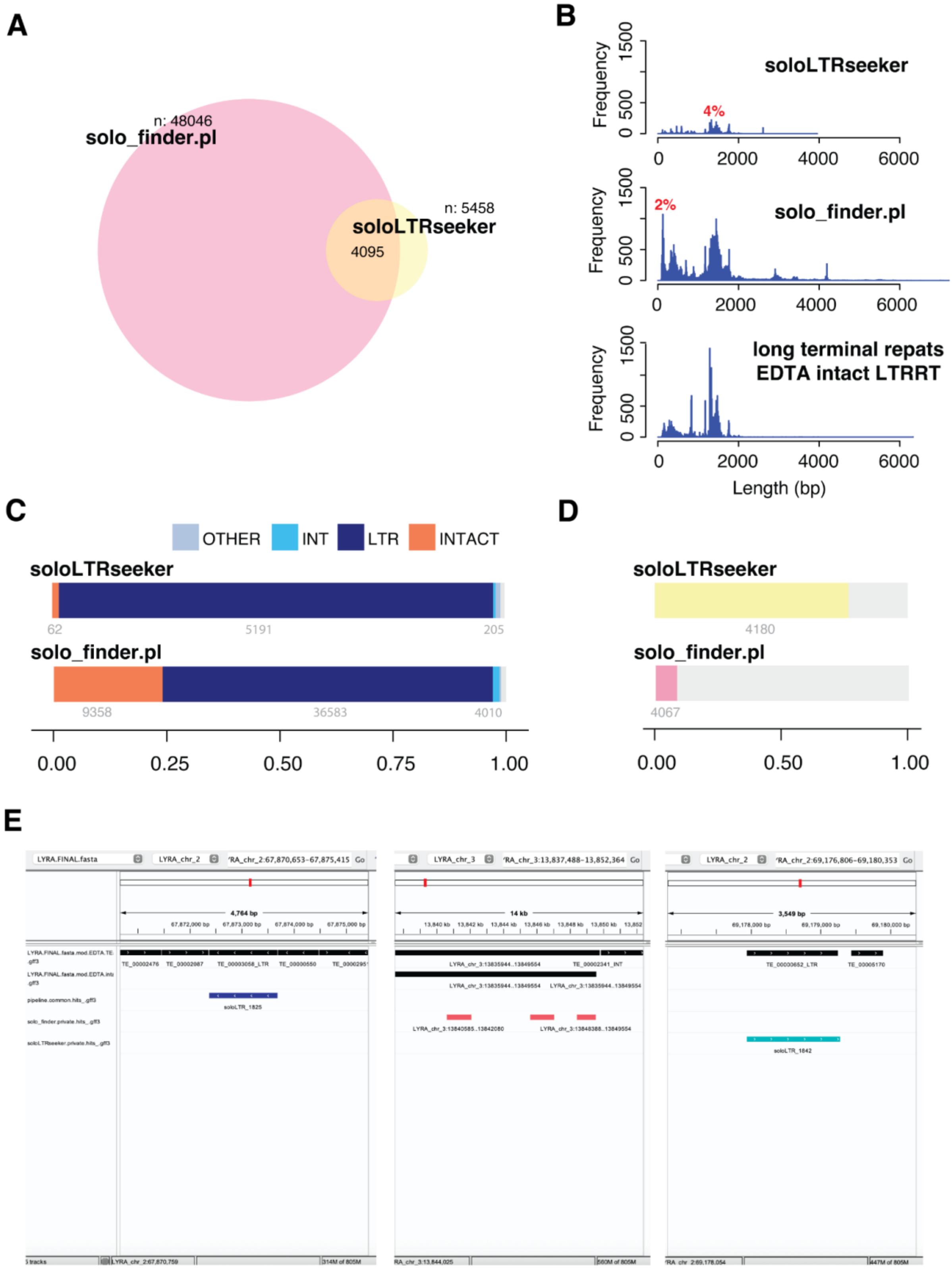

Figure R2.1. Comparisons between the soloLTRseeker and LTR-retriever.

A. Venn Diagram with the total number of annotated soloLTRs for each pipeline plus the number of common annotations. Most common annotations overlap perfectly. It is worth mentioning that 2,008 LTR_retriever soloLTRs overlap each other to some degree, of which 593 have >10 bp of overlap.

B. The length distribution (in 10 bp bins) of soloLTRs of soloLTRseeker (top), LTR-retriever (middle), and, for comparison, the LTRs of all intact LTR retrotransposons annotated by EDTA (bottom). The number in red indicates the bin with the highest frequency and the percentage of soloLTRs it includes. The highest peak of the LTR-retriever histogram is 120-130 bp, which is on the extreme low end of the LTR length distribution of intact elements. Similarly, there are many LTR-retriever soloLTRs with lengths that are rare among intact elements (e.g. ~4.3 kb).
C. The proportion of soloLTR nucleotides for all soloLTRs combined for soloLTRseeker (top) and LTR-retriever (bottom) that overlap different TE types based on the *EDTA.TEanno.split.gff3 file, which is the main EDTA output file that contains whole-genome non-redundant TE annotation. The grey numbers indicate the number of soloLTRs that overlap with the different TE types. For example, ~25% of the combined soloLTR length (i.e. total nucleotides) of 48,046 soloLTRs from LTR-retriever overlapped intact LTR retrotransposons. This overlap was derived from 9,358 soloLTRs. In contrast, for soloLTRseeker the proportion was 1.5%, derived from 62 soloLTRs.
D. The proportion of soloLTRs that are flanked with perfect 5 bp target site duplications (TSDs) for soloLTRseeker (77%, 4,180/5,458) and LTR-retriever (8.5%, 4,067/48,046).
E. Three IGV examples of soloLTRseeker and LTR-retriever annotations. The left screenshot shows a soloLTR identified by both pipelines, which overlap perfectly with an LTR annotation based on the EDTA.TEanno.gff3 file. The screenshot in the middle shows three soloLTRs identified only by LTR-retriever, which all overlap with a single intact LTR retrotransposon (EDTA.intact.gff3 file). The right screenshot shows a soloLTR identified only by soloLTRseeker, which overlaps perfectly with an LTR annotation.

For the mutation rate (r = 1.5e-7), we agree that mutation rates can vary across lineages, and therefore provide additional justification for the value used in our dating analyses:
1. The mutation rate (r = 1.5e-7) was originally inferred by Koch et al. (2000; https://doi.org/10.1093/oxfordjournals.molbev.a026248) from fossil-calibrated pollen data in *Arabidopsis*, *Arabis*, and related taxa within tribe Arabideae. To our knowledge, there are no lineage-specific estimates available for Biscutelleae; thus, we adopted the available rate from closely related Brassicaceae lineages. However, we emphasize that the assumed rate only affects the absolute values in dating, while the relative comparisons among species or TE elements remain unchanged.
2. The robustness of this mutation rate within Brassicaceae has been further confirmed by Hendriks et al. (2023; https://doi.org/10.1016/j.cub.2023.08.026), who constructed the largest Brassicaceae phylogeny to date. Their divergence time estimates for the crown group of the core Brassicaceae (20–23 Mya) closely match the estimates (23–26 Mya) obtained by Koch et al. (see Table R2.1 below, the two papers are highlighted in yellow).
3. Furthermore, we also note that the inferred subgenome divergence times in *Biscutella* are highly similar to those reported in *Brassica* (Figure 2f), a lineage for which the same mutation rate has been widely applied in WGD/WGT dating studies (Cheng et al., 2013, https://doi.org/10.1105/tpc.113.110486; Song et al., 2020, https://doi.org/10.1038/s41477-019-

0577-7; Jeong et al., 2016, https://doi.org/10.1007/s00122-016-2708-0). Therefore, we believe the current conclusions are sufficiently robust.

Table R2.1 Brassicaceae divergence time estimates

Studies	Crown core Brassicaceaea (Ma)	Method	Dataset	Calibration
Koch et al. 2000 (We employed the mutation rate proposed in this study)	25.9–23.1	synonymous substitution rate	Adh and *Chs*	synonymous substitution rate
Franzke et al. 2009	28.0–11.0–1.0	BEAST	nad4	one secondary calibration
Beilstein et al. 2010	54.3–46.9–39.4	BEAST	ndhF and *PHYA*	four fossils
Couvreur et al. 2010	43.8–32.3–20.9	BEAST	eight genes from nuclei, chloroplast, and mitochondria	one fossil
Kagale et al. 2014	26.6	synonymous substitution rate	213 nuclear orthologs	synonymous substitution rate
Hohmann et al. 2015	27.3–23.4–19.9	BEAST	plastomes	four fossils
Huang et al. 2016	30.3–29.7–29.1	r8s	113 low-copy nuclear orthologs	18 fossils
Mohammadin et al. 2017	35.4	BEAST	plastomes	one secondary calibration
Guo et al. 2017	29.8–25.1–21.3	MCMCTree	plastomes	14 fossils
Mandáková et al. 2017	30.6	BEAST	plastomes	four fossils
Huang et al. 2020	22.9–21.3–19.6	BEAST	plastomes	four fossils
Walden et al. 2020	29.6–25.1–20.9	BEAST	plastomes	four fossils
Legalov et al. 2022	>36.4	indirect calibration via phytophagous beetles	–	Ceutorhynchus beetle fossils
Hendriks et al., 2023 (The most large-scale phylogenetic tree of the Brassicaceae family to date)	22.4–21.1–19.9	treePL	phylogeny using superstrict routine; calibration using subset of 20 most clock-like genes	one fossil validated by nine secondary calibration points

Hendriks et al., 2023	24.3–16.9–10.2	treePL	plastomes	one fossil validated by eight secondary calibration points

R2-Q7: The assignment of A/B compartments at 50-kb resolution and TAD calling methods are insufficiently detailed. Which tool and parameters were used to call TADs? How sensitive are the results to resolution or calling thresholds? Including reproducibility details (e.g., code repositories, parameter files) is essential.

A: Thank you for this comment. We have now expanded the Methods section to provide more details. Specifically, A/B compartments were identified using the hicPCA module in HiCExplorer. TADs were annotated via the hicFindTADs module. Detailed parameter settings are now provided in the Methods section. To assess sensitivity, we tested multiple resolutions (10 kb, 20 kb, 50 kb, and 100 kb; see Figure R2.2 below) and selected 50 kb as a compromise between resolution and sequencing depth, as described by Ramírez et al. (2018; https://doi.org/10.1038/s41467-017-02525-w).

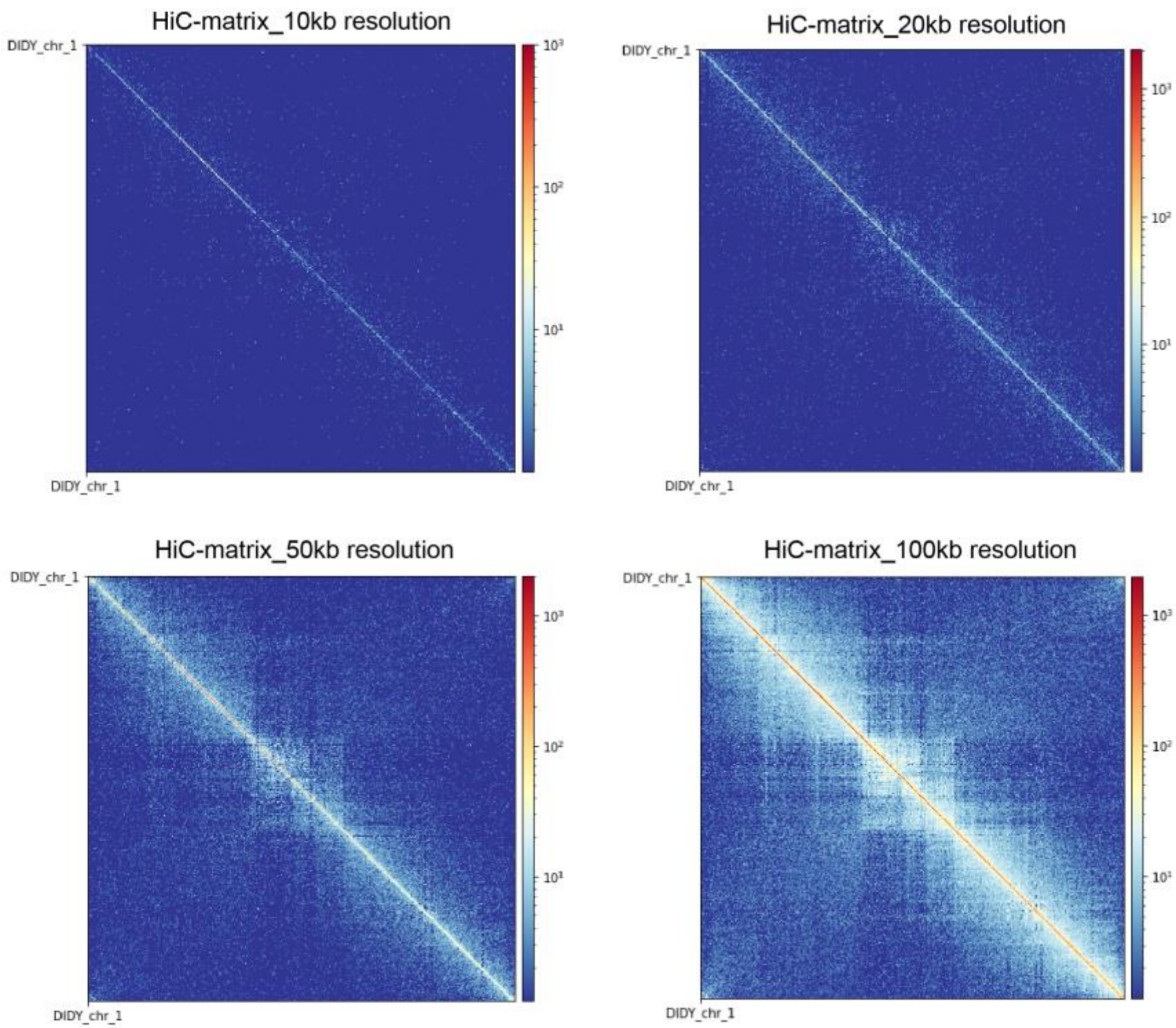

Figure R2.2. Raw HiC maps of chromosome 1 in *B. didyma* at resolutions of 10, 20, 50, and 100 kb.

R2-Q8: Rates of chromosomal rearrangements (CRs/Mya) are reported (e.g., 3.75 CRs/Mya early vs. 0.72 CRs/Mya late), but no confidence intervals or statistical tests are provided. Given phylogenetic uncertainty in divergence times, how robust are these rate estimates?

A: We appreciate the reviewer’s comment. To address this concern, we have now included divergence time estimates from MCMCtree, which provides Bayesian confidence intervals.

Importantly, the divergence times inferred with MCMCtree are highly consistent with those obtained using r8s, PATHd8, and the RelTime method in MEGA X (these three algorithms do not provide confidence intervals; see Figure R2.3 below or Supplementary Fig. 12 in the revised Supplementary Information). For further clarity, we have revised the Main Text (lines 247-250) to ensure that the divergence times used in the rate calculations represent the average values across all four methods. This approach helps to minimize potential biases from relying on any single method.

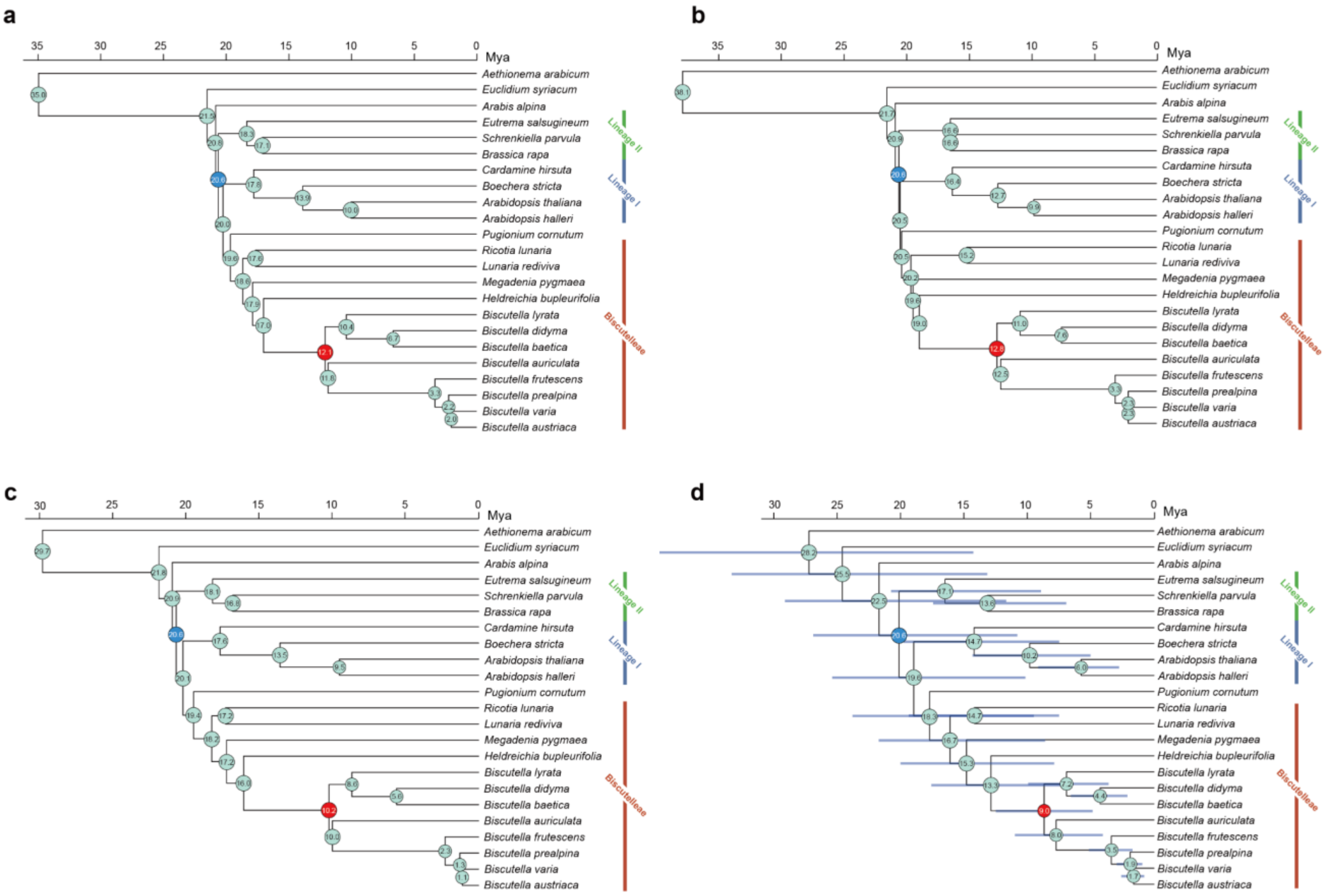

Figure R2.3 (Supplementary Fig. 12). Time-calibrated trees inferred by r8s (a), PATHd8 (b), RelTime (c), and MCMCtree (d) methods. The four different calculating strategies show high consistency. The divergence time of Lineage I (Camelinodae) and Lineage II (Brassicodae) was used as the calibration node (the blue dot at 20.6 Mya, http://www.timetree.org/). The crown node of the *Biscutella* species is labeled as red dots.

R2-Q9: The manuscript asserts that shared genomic features predispose certain regions to recurrent breakage. Yet, without functional validation (e.g., assays of recombination frequency or chromatin state), this remains hypothetical. The discussion should be more tempered and explicitly acknowledge the correlative nature of the evidence.

A: Thank you for your suggestions. We addressed this objection above. We concur that our current

evidence indicates a correlative rather than a causal link between recurrent rearrangements, TE enrichment, and 3D genome architecture. As stated above, to enhance the robustness of our findings, we conducted additional simulations using 200 randomly selected genomic regions. These simulations consistently demonstrated that the observed genomic features of the hotspot regions are highly unlikely to occur by chance (see Supplementary Figs. 21, 23, 25, and 26 in the updated Supplementary Information). All in all, these analyses further support the non-random nature of the identified breakage hotspots.
We acknowledge the reviewer's argument and emphasize the correlational nature of the evidence in our revised Discussion from a broader perspective.

R2-Q10: The proposed link between D-class MADS-box gene fractionation and evolution of lens-like silicles is intriguing, but entirely based on Ka/Ks and copy-number patterns. Are there expression data or phenotypic analyses that can corroborate this hypothesis? Otherwise, it should be presented only as a plausible model.
A: Thank you for your comments. To address this point, we performed an additional expression analysis (see Figure R2.4 below or Supplementary Fig. 30 in the revised Supplementary Information file). RNA-seq data of floral buds and open flower tissues from *Biscutella austriaca* were obtained from Beringer et al. (2024; https://doi.org/10.1093/molbev/msae155). Compared to non-D-class MADS-box genes, D-class genes generally showed lower expression levels, with significantly reduced expression in floral buds. Although the analyzed *B. austriaca* accession is not identical to the AUST accession used in our study, these data nonetheless suggest that the D-class genes are suppressed in the floral bud and flower tissues. In addition, we have acknowledged these limitations in the Discussion section and clarified that our conclusion remains a plausible model, which will require further functional and phenotypic analyses in the future.

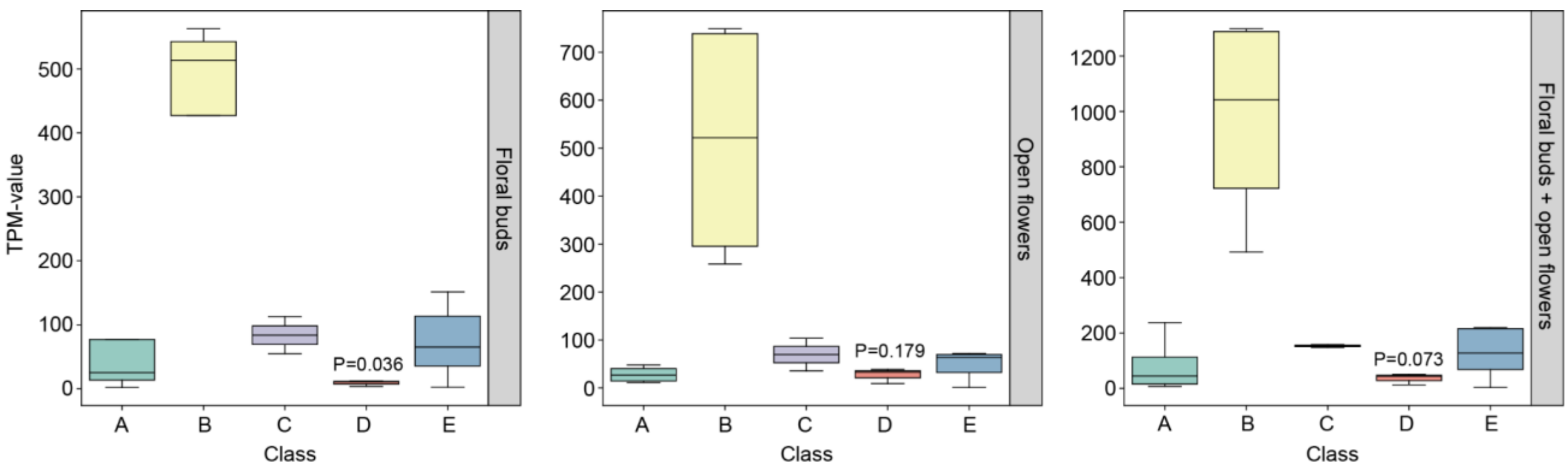

Figure R2.4 (Supplementary Fig. 30). Expression levels of floral buds and open flower tissues in *B. austriaca* were quantified using transcripts per million (TPM) values. The RNA-seq data were obtained from Beringer et al., 2024. Compared to non-D class genes, the expression levels of D-class genes are generally lower (Wilcoxon rank-sum test).

Minor Comments

R2-Q11: The Abstract contains a duplicate sentence (lines 1822): we identified 12 chromosome breakage hotspots; Please remove redundancy.

A: Thank you (we realized the duplication too late…). The error was fixed.

R2-Q12: Clarify whether the TE enrichment analyses account for local GC content or other genomic features that influence TE distribution

A: We appreciate the reviewer's question. In our analysis, we calculated the density and distribution of transposable elements (TEs) based on genome-wide annotations. We hard-masked the gene-flanking sequences as "N" whenever they overlapped with the coding regions of adjacent genes, as detailed further in the Methods section. We did not explicitly control for local GC content or other sequence features in our calculations. It is important to note that our analysis was conducted on a genome-wide scale to illustrate the overall distribution pattern of TEs in relation to genes. While local sequence composition, such as GC content, can influence transposon insertion preferences at specific sites, these biases are not likely to systematically affect the genome-wide trends we observed. Similar methodologies have been utilized in various studies (e.g., Cheng et al., 2016, https://doi.org/10.1111/nph.13884; Zhang et al., 2023, https://doi.org/10.1073/pnas.2305208120), and we also asked Zhang et al. regarding this strategy and got a similar answer as described above. Thus we believe they are sufficiently robust.

R2-Q13: Inconsistent nomenclature: the terms "Tsuneq"; "Tuneq"; and "unequal reciprocal translocations"; appear interchangeably. Adopt a single abbreviation throughout.

A: Thank you for pointing this out! We realized that we incorrectly abbreviated "Tuneq" and "Tsuneq" to differentiate between singular and plural forms. To clarify, we reviewed the literature. In human studies, such translocations have been described as "unbalanced reciprocal translocations" (Duckett et al., 1984 https://doi.org/10.1007/BF00272991; Chang et al., 2013 https://doi.org/10.1016/j.jcma.2012.11.002). Schubert and Lysak (2011; https://doi.org/10.1016/j.tig.2011.03.004) referred to these translocations as "non-reciprocal translocations". To avoid any confusion, we have updated all instances of "unequal reciprocal translocations" in the Main Text to "unbalanced reciprocal translocations", abbreviated as "unbalanced-RT", in line with the traditional nomenclature used in human studies.

R2-Q14: The description of the two clades as "early-diverging (n = 8/6) and late-diverging (n = 9)"; may confuse readers; consider labeling them "clade I"; and "clade II"; or similar, with an explicit note that clade I corresponds to basal lineages.

A: Thank you for your suggestion. We initially followed the proposed terminology, but we later concluded that the terms "I" and "II" do not provide any additional (useful) information. The phylogenetic relationships within *Biscutella* are not best represented by a simple "clade I / clade II" scheme. For instance, *B. auriculata* does not cluster with the species characterized by n = 8/6; instead, it is phylogenetically closer to the species with n = 9, while still being an early-diverging species. We acknowledge that our nomenclature is also not ideal, as it fails to adequately describe the degree of genome diploidization—here we come again to the issue of n = 9 species vs. *B.*

auriculata mentioned above. Overall, we would prefer to use the terms "early-diverging" and "late-diverging," which have also been endorsed by other reviewers.

R2-Q15: Describe the criteria used for defining and detecting chromosomal breakpoints and rearrangements.
A: Thank you for your suggestion. We have now included more detailed criteria information in the revised Methods section (lines 726-761).

R2-Q16: In Figure 3b, the CR events are represented by colored arrows but the legend does not clarify which color corresponds to which event type.
A: Thank you. The new Fig. 5a has labelled breakpoints of different origins in different colors.

Overall, the manuscript presents a valuable dataset and addresses an important question in plant genome evolution, but the concerns above must be addressed before acceptance.

Reviewer #3 (Remarks to the Author):
I co-reviewed this manuscript with one of the reviewers who provided the listed reports. This is part of the Nature Communications initiative to facilitate training in peer review and to provide appropriate recognition for Early Career Researchers who co-review manuscripts.
Authors: Thank you very much for your time and effort.

Reviewer #4 (Remarks to the Author):
Dear authors:
Overall, this is a very well analyzed, interpreted, and written manuscript, presenting findings of broad general interest: by sequencing eight Biscutella species descended from an allotetraploid ancestor (n=14), the study demonstrates that independent descending dysploidy (reducing chromosomes to n=9, 8, 6) involves both convergent and divergent genomic features and identifies recurrent chromosome breakpoint hotspots enriched in LTR retrotransposons at TAD boundaries, suggesting underlying shared mechanisms despite the process appearing random. I only have a few minor comments as shown below:
A(authors): We appreciate the time and effort you have dedicated to evaluating our work.

R4-Q1: Line 40-42: This sentence was written twice in the Abstract section.
A: Thank you (we realized the duplication too late…). The error was fixed.

R4-Q2: Line 66: Please provide the full expression of DSB for the first time.
A: The full expression has been provided.

R4-Q3: Line 69-70: Please provide the reference for this sentence.
A: Thank you for pointing this out. We have added the references accordingly.

Original Text: WGD events often occur before the diversification of a clade, leading to the assumption that all clade members are descended from a single polyploid genome or metapopulation.

Revised Text (lines 69-70): WGD events often occur before the diversification of a clade[18,19], leading to the assumption that all clade members are descended from a single polyploid genome or metapopulation[20].

18. Schranz, M. E., Mohammadin, S. & Edger, P. P. Ancient whole genome duplications, novelty and diversification: the WGD Radiation Lag-Time Model. *Curr. Opin. Plant Biol.* **15**, 147-153 (2012).
19. Soltis, P. S. & Soltis, D. E. Ancient WGD events as drivers of key innovations in angiosperms. *Curr. Opin. Plant Biol.* **30**, 159-165 (2016).
20. Jiao, Y. et al. Ancestral polyploidy in seed plants and angiosperms. *Nature* **473**, 97-100 (2011).

Reviewer #5 (Remarks to the Author):

Huang and colleagues present a manuscript titled: Post-polyploid chromosomal diploidization in plants is affected by clade divergence and constrained by shared genomic features; that reports on the diploidization process from an allopolyploid ancestor in the Biscutella genus using whole genome sequences and Hi-C chromosome conformation of 8 (sub)species. The authors show that the different species underwent distinct diploidization histories leading to variable chromosome numbers and arrangements. Interestingly, even species with the same chromosome number underwent different diploidization histories. The early-diverging clade is shown to have in general lower chromosome number, higher variability of TAD length/number and A/B compartment assignment, and higher retrotransposon removal. Interestingly, the authors also identify specific breakpoint hotspots that are found across multiple species, which very often occur in (peri)centromeric regions and are associated with TAD boundaries. Finally, the authors explore (more briefly) genic diploidization and functional biases therein. In all, the authors present a thorough and high quality description of genome evolution in the Biscutella genus. The methodology is sound and reproducible. The authors use different approaches to confirm their conclusions (e.g., bottom-up and top-down approach for inferring the ancestral karyotype).

We believe this work will be of considerable significance for the field of polyploidy and diploidization. To our knowledge, this is the first paper to investigate chromosomal diploidization by analyzing the genomes across multiple species in a genus originating from a common polyploid ancestor. It follows on earlier cytogenic-based work on diploidization of the same research group (but not this clade, e.g., https://doi.org/10.1111/mec.14379).

A(authors): We sincerely appreciate both reviewers for their positive evaluation of our work and valuable suggestions.

Me and my co-reviewer have five major comments on the methods and discussion that need more consideration and we raise a number of small comments in a line-by-line manner.

R5-Q1: For the top-down ancestral reconstruction, the authors indicate; A shared paracentric inversion event (Ipa), which restructured the ancestral chromosome AK8/6 by rearranging the V+Wa+Q+X association into V+X+Q+Wa, was identified in both subgenomes in all Biscutella species; (Lines 211-213). However, I do not observe this in Figure S12a, at least not completely. For example the association between V+X in the LF sub-genome is only found in 3 (all n = 8 species) of the 6 karyotypes. How can this be explained?
A: We appreciate the reviewers for bringing this to our attention. It is true that the V+X+Q+Wa association is not consistently retained in the LF subgenome across all *Biscutella* species (see Table R5.1 below). In three species (FRUT, PREA, and LYRA) only the X+Q+Wa association remains intact, as the V block has been translocated to different chromosomes. This translocation occurred either through a shared event (in FRUT and PREA) or a species-specific event (in LYRA).

Nevertheless, **all *Biscutella* species maintain the X+Q+Wa association in the LF subgenome and the complete V+X+Q+Wa association in the MF subgenome**. Furthermore, the V+X+Q+Wa association is generally conserved in the closely related genera *Megadenia* (Yang et al., 2020; https://doi.org/10.1111/1755-0998.13291) and *Heldreichia* (Guo et al., 2021; https://doi.org/10.1093/molbev/msaa327). These findings collectively support the hypothesis that V+X+Q+Wa represents one of the ancestral associations.

To clarify this point and prevent misunderstandings, we have revised the original text as follows:

Original Text: A shared paracentric inversion event (Ipa), which restructured the ancestral chromosome AK8/6 by rearranging the V+Wa+Q+X association into V+X+Q+Wa, was identified in both subgenomes in all *Biscutella* species, as well as in two other Biscutelleae species – *M. pygmaea* and *H. bupleurifolia* (Figures 3a and S12a).
Revised Text (lines 210-214): A shared paracentric inversion event (Ipa), which restructured the ancestral chromosome AK8/6 by rearranging the V+Wa+Q+X association into V+X+Q+Wa, is supported by the presence of the derived V+X+Q+Wa or X+Q+Wa association in both subgenomes of all *Biscutella* species, as well as in two other Biscutelleae species – *M. pygmaea* and *H. bupleurifolia* (Fig. 3a and Supplementary Fig. 13).

Table R5.1 Presence of V+X+Q+Wa or X+Q+Wa associations in the LF and MF subgenomes of *Biscutella* and other Biscutelleae species.

LF subgenome			MF subgenome			Other species		
GB association	Species	Chr.	GB association	Species	Chr.	GB association	Species	Chr.
**X+Q+Wa**	**FRUT**	**chr4**	V+X+Q+Wa	FRUT	chr1	V+X+Q+Wa	*M. pygmaea*	chr6
**X+Q+Wa**	**PREA**	**chr4**	V+X+Q+Wa	PREA	chr1	V+X1	*H. bupleurifolia*	chr1

V+X+Q+Wa	AURI	chr8	V+X+Q+Wa	AURI	chr3	X2+Q+Wa	*H. bupleurifolia*	chr4
V+X+Q+Wa	BAET	chr7	V+X+Q+Wa	BAET	chr2			
V+X+Q+Wa	DIDY	chr7	V+X+Q+Wa	DIDY	chr5			
**X+Q+Wa**	**LYRA**	**chr3**	V+X+Q+Wa	LYRA	chr2			

R5-Q2: Related to results of this top-down strategy that are represented in figure S12. If I correspond the presence and absence of gene blocks neighbor pairs, not all of them seem to be present here. E.g., according to figure S17 the lyrata genome on chr4 has a K gene block neighboring I1 and B. Figure S12 has the I+KL as presence for lyrata, but no K+B (red nor grey). Was this K+B not considered? Is this only a partial representation or was there only a subset of neighbor pairs considered? It does not seem the case that only neighbors in the ancestral (A3) reconstruction are shown because D + U is a pair that was formed between A3 and lyrata. Only pairs between a MF and LF gene block are not considered? Please clarify in methods.
A: Thank you for your valuable comment. In the original version of Supplementary Fig. 12, we only included genomic block (GB) associations within the LF and MF subgenomes, and did not show the associations between these two subgenomes. Therefore, the KL (from LF) + B (from MF) in LYRA you mention was not included in the original Supplementary Fig. 12. Our initial rationale was that the goal of this analysis is to reconstruct the ancestral karyotypes of the two progenitor genomes (LF and MF), and inter-subgenomic GB associations provide limited information compared to intra-subgenomic associations. As a result, we focused solely on intra-subgenomic GB associations in the earlier version of the figure. To address this ambiguity and improve clarity, we have now updated this figure in the revised Supplementary Information (now Supplementary Fig. 13) to include a heatmap of GB associations between the LF and MF subgenomes as well.

R5-Q3: The breakpoint hotspot regions were identified as breakpoints shared by multiple species compared to the ancestral genome. What remains unclear to me is whether these breakpoints were shared because it originated in the ancestor of these species or whether the breakpoints occurred by convergence. The authors mention that these breakpoints are recurrent, but it is not clear whether this means that these breakpoints occurred multiple times independently or if it occurred once in their post-polyploid common ancestor. If this was not considered yet, I believe that categorizing breakpoint hotspots in ancestral and convergent breakpoints is essential for the interpretation of convergence.
While the article does propose a hypothesis on the convergent genome evolution in the diploidization process through chromosome rearrangements (line 75), the results and discussion do not really focus on this hypothesis explicitly. Consider making the focus on this hypothesis stronger by discussing what results show a convergent pattern and what underlying universal process could explain it. The current state of the manuscript provides does not go further than a discription of a

number of patterns, but by answering this hypothesis could provide a stronger scientific conclusion and justify the title of the manuscript.

A: We would like to thank the reviewers for their constructive critique, which we found to be well justified. As often happens in science, our expectations were somewhat too optimistic, and the reality proved to be more complex. In the following text (and in the revised Main Text), we have made an effort to implement your suggestions, and we believe we have successfully addressed your questions to a large extent. We again thank you for this stimulus, which has further improved the interpretation and impact of our results.

In our original analysis, we compared hotspots based on phylogenetic relationships. To address the reviewer's concerns, we have now performed synteny comparisons between any two species, regardless of their phylogenetic relationships. This approach allowed us to identify more recurrent breakpoints, increasing their number from 12 to 14. Based on their distribution across the eight *Biscutella* genomes and five phylogenetic nodes, we classified these 14 hotspots (HOTs) into two categories: (i) ancestral HOTs (8), which are present exclusively in a single phylogenetic clade, and (ii) recurrent HOTs (6), which are shared by extant species or reconstructed ancestors from at least two independent phylogenetic clades. To provide further clarification, we have added a new supplementary figure that traces the phylogenetic origin of each HOT region (see Figure R5.1 below or Supplementary Fig. 20 in the revised Supplementary Information file). Both ancestral and recurrent hotspots exhibit similar genomic features, including transposable element enrichment, frequent A/B compartment switches, and co-localization with TAD boundaries.

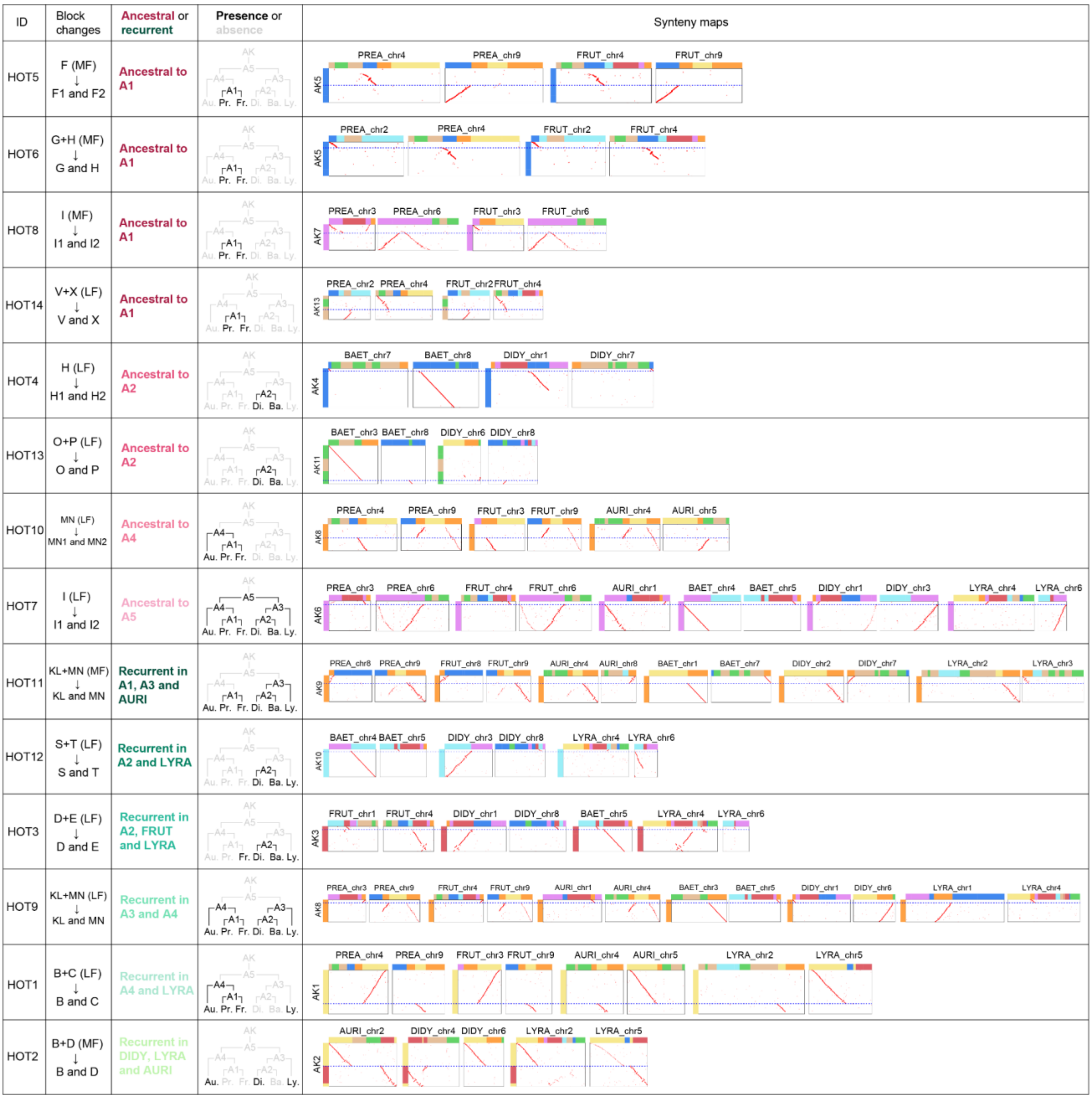

Figure R5.1 (Supplementary Fig. 20). The location details of 14 chromosome breakage hotspots (HOT1 to HOT14). The second column specifies the GB associations that are altered due to the corresponding breakpoints. The third and fourth columns indicate whether each breakpoint is classified as ancestral (in gradient red) or recurrent (in gradient green), based on its presence or absence across different species and phylogenetic nodes. A breakpoint is considered ancestral if it appears within a single clade and is present in the inferred ancestral karyotype of that clade. In contrast, breakpoints found in multiple different clades are classified as recurrent. The fifth column displays synteny comparisons between the reconstructed ancestral genome and the six *Biscutella* genomes. Dashed lines indicate breakpoints that are shared among species, occurring at the same genomic positions.

R5-Q4: Finally, I think the paper would be even stronger if the discussion delved a bit deeper in the findings of the study. For example, I think the underlying cause of the association between TAD boundaries and breakpoint hotspots is not obvious and I wonder whether the authors have any hypotheses regarding that. In addition, I wonder what might have caused the observed patterns between the early-diverging and late-diverging clades. Is it just because the divergence time was more ancient that there was more variability observed in the early-diverging clade? I also wondered whether the authors have an idea about the underlying cause of the differential gene fractionation between the sub-genomes.
A: Thank you for your suggestion. In the previous version, the Discussion section was relatively short due to word count limitations. In the revised manuscript, we have substantially expanded and refined this section. The updated Discussion now covers several key aspects, including: the origin of the *Biscutella* subgenome (second paragraph); the mechanisms and rates of descending dysploidy (third paragraph); the potential relationship between chromosome breakage and its three-dimensional chromatin architecture (fourth paragraph); and the role of the diploidization in the emergence or stabilization of fruit morphological diversity within the Biscutelleae (fifth paragraph). We believe that these additions provide a more comprehensive interpretation of our findings.

Specific comments, by line number:
R5-Q5: Line 37: It would be more clear to use the term “gene fractionation” (across the whole manuscript) as it emphasizes the fact that you are talking about “gene loss“ following polyploidy.
A: We thank you for your suggestion, we agree with the reviewer’s point and have clarified the “gene fractionation” here refers to gene loss following WGD.

R5-Q6: Line 42: descendig; should be descending;.
A: The typo was fixed.

R5-Q7: Line 45: Which species are you referring to, to have two and several hundred chromosome pairs?
A: We have added detailed examples for further clarification.
Original Text: Land plants are known for their remarkable karyological variation, with chromosome numbers varying from two pairs to several hundred pairs.
Revised Text (lines 45-47): Land plants are known for their remarkable karyological variation, with chromosome numbers varying from two pairs (n = 2; e.g., *Colpodium versicolor* in Poaceae) to several hundred pairs (e.g., *Ophioglossum* species in Ophioglossaceae)[1].

1. Rice, A. et al. The Chromosome Counts Database (CCDB)–a community resource of plant chromosome numbers. *New Phytol.* **206**, 19-26 (2015).

R5-Q8: Lines 46-48: This extraordinary variation is due to two antagonistic evolutionary processes: polyploidy (whole-genome duplication, WGD) and diploidization; It's a strong claim that only these two processes are responsible for the variation in chromosome numbers in all land plants. While maybe the case in *Biscutella*, is there support for this claim across all plants (by means of references and/or better argument)?

A: We agree with your comment - our statement oversimplifies complex processes with many drivers and diverse consequences. We have revised the original sentence and added several examples to further support our view. Also, "primarily" was added.

Original Text: This extraordinary variation is due to two antagonistic evolutionary processes: polyploidy (whole-genome duplication, WGD) and diploidization

Revised Text (lines 47-49): This extraordinary variation is primarily shaped by two antagonistic evolutionary processes: polyploidy (whole-genome duplication, WGD) and diploidization[2,3,4,5,6,7,8].

2. Mandáková, T. & Lysak, M. A. Post-polyploid diploidization and diversification through dysploid changes. *Curr. Opin. Plant Biol.* **42**, 55-65 (2018).

3. Ren, L., Huang, W. & Cannon, S. B. Reconstruction of ancestral genome reveals chromosome evolution history for selected legume species. *New Phytol.* **223**, 2090-2103 (2019).

4. Winterfeld, G., Ley, A., Hoffmann, M. H., Paule, J. & Röser, M. Dysploidy and polyploidy trigger strong variation of chromosome numbers in the prayer-plant family (Marantaceae). *Plant Syst. Evol.* **306**, 36 (2020).

5. Wang, Z. H. et al. Reshuffling of the ancestral core-eudicot genome shaped chromatin topology and epigenetic modification in *Panax*. *Nat. Commun.* **13**, 1902 (2022).

6. Huang, Y., Guo, X., Zhang, K., Mandakova, T., Cheng, F. & Lysak, M. A. The meso-octoploid *Heliophila variabilis* genome sheds a new light on the impact of polyploidization and diploidization on the diversity of the Cape flora. *Plant J.* **116**, 446-466 (2023).

7. Jiang, X. et al. Chromosome fusions shaped karyotype evolution and evolutionary relationships in the model family Brassicaceae. *Nat. Commun.* **16**, 4631 (2025).

8. Zhang, R. G. et al. Reticulate allopolyploidy and subsequent dysploidy drive evolution and diversification in the cotton family. *Nat. Commun.* **16**, 7480 (2025).

R5-Q9: Lines 56-57: "a common way of descending dysploidy is non-allelic homologous recombination (NAHR) between two or more different chromosomes, reducing the number of chromosomes typically by one…"; Since this process is central to the manuscript, you may want to explain this a bit further (E.g., how can recombination reduce the number of chromosomes?) and/or provide a figure (if journal limits allow it; something along the lines of figure S14 but maybe simpler).

A: We appreciate the reviewer's suggestion. Considering the complexity of descending dysploidy mechanisms and the space limitations in the Main Text, we have moved the original Figure S14 to the new Supplementary Fig. 1, which specifically illustrates these processes. A more detailed explanation has been provided in the figure legend.

R5-Q10: Lines 58-59: Is diploidizing descending dysploidy; not by definition after WGD events?

A: No, it is not. This is somewhat of a philosophical issue. If we consider all seed plants to be polyploids (originated after a WGD), then the comment may hold some validity. However, it is more practical to distinguish cases of (non-diploidizing) descending dysploidy that occurred, for example, 25 to 30 million years after the last WGD. A good example of this is *Arabidopsis thaliana*, which has five chromosomes that originated from an ancestral genome with n = 8 (through n = 7 and 6?). Therefore, the question becomes: to what extent do n-1 descending dysploidies contribute to the diploidization of a genome? The term "diploidizing descending dysploidy" should emphasize the significance of chromosome number reduction in the post-polyploid diploidization process.

R5-Q11: Line 65: This refers to DNA homology and repeat content; between non-homologous chromosomes then?
A: This is not simple to answer unambiguously. In the origin of complex fusion chromosomes, intrachromosomal rearrangements (typically inversions), can also occur. However, it is true that non-homologous chromosomes are primarily what is meant in this context. We believe that the original wording is sufficiently general while still being specific enough.

R5-Q12: Line 66: DSB not yet explained
A: The full expression has been provided.

R5-Q13: Lines 69-71: WGD events often occur before the diversification of a clade, leading to the assumption that all clade members are descended from a single polyploid genome or metapopulation. The polyploid clade diversifies as a result of adaptive radiation...; Please include references for these statements.
A: Thank you for pointing this out. We have added the references accordingly.
Original Text: WGD events often occur before the diversification of a clade, leading to the assumption that all clade members are descended from a single polyploid genome or metapopulation.
Revised Text (lines 69-70): WGD events often occur before the diversification of a clade[18,19], leading to the assumption that all clade members are descended from a single polyploid genome or metapopulation[20].

18. Schranz, M. E., Mohammadin, S. & Edger, P. P. Ancient whole genome duplications, novelty and diversification: the WGD Radiation Lag-Time Model. *Curr. Opin. Plant Biol.* **15**, 147-153 (2012).
19. Soltis, P. S. & Soltis, D. E. Ancient WGD events as drivers of key innovations in angiosperms. *Curr. Opin. Plant Biol.* **30**, 159-165 (2016).
20. Jiao, Y. et al. Ancestral polyploidy in seed plants and angiosperms. *Nature* **473**, 97-100 (2011).

R5-Q14: Line 135: Could you introduce these 22 ancestral genomic blocks? At least I was not familiar with it.
A: Thank you for asking. The boundaries of 22 conserved genomic blocks (GBs) were identified based on cross-species *in situ* hybridization of chromosome-specific BAC contigs of the model

species *A. thaliana* and comparative genetic mapping between *A. thaliana* and other crucifer species. These GBs have been widely used to reconstruct chromosome and genome evolution in the Brassicaceae family (e.g., Lysak et al., 2016; Mandáková et al., 2008, 2010, 2013; Burrell et al., 2011; Wu et al., 2012; Gup et al., 2021; Huang et al., 2023; Jiang et al., 2025).
We also revised our sentences to help the reader better understand the concept.
Original Text: Synteny analysis further identified 22,492 to 31,397 gene pairs in the eight *Biscutella* genomes, corresponding to 22 ancestral genomic blocks (GBs A – X; Schranz et al., 2006; Lysak et al., 2016; Figure S2).
Revised Text (lines 133-136): Synteny analysis further identified 22,492 to 31,397 gene pairs across the eight *Biscutella* genomes, corresponding to 22 ancestral genomic blocks (GBs A – X; defined as conserved chromosomal segments in the Brassicaceae genomes)[31,32].

31. Schranz, M. E., Lysak, M. A. & Mitchell-Olds, T. The ABC's of comparative genomics in the Brassicaceae: building blocks of crucifer genomes. *Trends Plant Sci.* **11**, 535–542 (2006).
32. Lysak, M.A., Mandáková, T. & Schranz, M.E. Comparative paleogenomics of crucifers: ancestral genomic blocks revisited. *Curr. Opin. Plant Biol.* **30**, 108–115 (2016).

R5-Q15: Line 141: I do not observe this exception for block G in B. prealpina and B. varia in Figure S2, maybe due to lower resolution.
A: Block G is the shortest genomic block in physical length, which may make it less clear in the original figure. We have enhanced the figure resolution and rearranged the subgraphs to improve visualization.

R5-Q16: Lines 193-195: Note that the dating represents not necessarily the time of hybridization, but rather the divergence of the (diploid) progenitor species.
A: We thank the reviewer for this comment. We have revised the Main Text as follows:
Original Text: The ancestral *Biscutella* genome originated from hybridization between two divergent progenitor species around 11 – 13 million years ago (Mya),
Revised Text (lines 194-195): The ancestral *Biscutella* genome originated from hybridization between two progenitor species that had diverged around 9 – 13 million years ago (Mya),

R5-Q17: Line 207: Please write ACK, ancPCK and PCK first time in full.
A: We apologize for any confusion this may have caused. The complete expressions have been provided.
Original Text: The top-down approach included comparing frequent GB associations in *Biscutella* species with those in the known ancestral karyotypes of Brassicaceae (ACK, ancPCK, and PCK; Schranz et al., 2006; Mandáková & Lysak, 2008; Geiser et al., 2016; Lysak et al., 2016).
Revised Text (lines 205-208): The top-down approach included comparing frequent GB associations in *Biscutella* species with those in the known ancestral karyotypes of Brassicaceae, including Ancestral Crucifer Karyotype (ACK)[31,32], ancestral Proto-Calepineae Karyotype (ancPCK)[25] and Proto-Calepineae Karyotype (PCK)[34].

25. Geiser, C., Mandáková, T., Arrigo, N., Lysak, M. A. & Parisod, C. Repeated whole-genome duplication, karyotype reshuffling, and biased retention of stress-responding genes in Buckler mustard. *Plant Cell* **28**, 17-27 (2016).
31. Schranz, M. E., Lysak, M. A. & Mitchell-Olds, T. The ABC's of comparative genomics in the Brassicaceae: building blocks of crucifer genomes. *Trends Plant Sci.* **11**, 535–542 (2006).
32. Lysak, M.A., Mandáková, T. & Schranz, M.E. Comparative paleogenomics of crucifers: ancestral genomic blocks revisited. *Curr. Opin. Plant Biol.* **30**, 108–115 (2016).
34. Mandáková, T. & Lysak, M. A. Chromosomal phylogeny and karyotype evolution in x = 7 crucifer species (Brassicaceae). *Plant Cell* **20**, 2559-2570 (2008).

R5-Q18: Lines 246-249: Why would you have expected Tsuneq to be correlated and for example not EET and NCI, as these reduce the number of chromosomes directly?

A: We appreciate the reviewers' comments and the chance to clarify our interpretation. Our aim was to show that descending dysploidy in *Biscutella* is not solely influenced by any one type of chromosomal rearrangement (CR). Unbalanced reciprocal translocations (previously referred to as Tuneq and now abbreviated to unbalanced-RT) were mentioned because they represent the most frequently identified category of CRs in *Biscutella* genomes. In the updated manuscript, we have further categorized unbalanced-RTs into dysploid and non-dysploid types (also can see Supplementary Fig. 1 for illustration). Considering contextual connections, the original sentence has now been deleted.

R5-Q19: Lines 267-270: Have you done a statistical test to confirm this pattern?

A: Thank you. The Wilcoxon rank-sum *P*-value is now included in the Main Text (lines 266-267).

R5-Q20: Line 308: In the extant Biscutella genomes, more than half of the rejoined junctions aligned with pericentromeric regions (Figure 5b). Is there a (statistical) test against an expectation possible to show that rejoined paleojunctions associated with a) pericentromere, b) TAD boundaries and c) A/B compartment boundaries (so figure 5b). E.g., the likelihood of being a TAD boundary by chance can be expected to be (the zone that is considered boundary)/(average TAD length). It would grant more power to statements being made based on arbitrary TAD boundary size and pericentromeric region sizes. 23/40 sounds like not a lot if half the chromosome is considered as pericentromeric. Alternatively, mention this context somewhere of how big the pericentromeric region, TAD boundaries and A/B boundaries are to better interpret these results.

A: Thank you for your suggestion. We agree with the reviewers that assessing statistical expectations is important in determining whether rejoined paleojunctions are non-randomly associated with pericentromeres, TAD boundaries, and A/B compartment transitions. To address this, we conducted 200 simulations by randomly sampling genomic regions of comparable size (150 kb) across the *Biscutella* genomes. We then compared:

(1) the overlap frequency of between HOT regions and random regions with pericentromeric regions (Figure R5.2 or Supplementary Fig. 21 in the revised Supplementary Information)
(2) the content of LTR-RTs within HOT regions versus random regions (Figure R5.3 or Supplementary Fig. 23 in the revised Supplementary Information)

(3) the frequency of A/B compartment transitions within HOT regions versus random regions (Figure R5.4 or Supplementary Fig. 25 in the revised Supplementary Information)
(4) the overlap frequency of between HOT regions and random regions with TAD boundaries (Figure R5.5 or Supplementary Fig. 26 in the revised Supplementary Information)

The observed frequencies or content mentioned above in HOT regions were consistently and significantly higher than those expected from the random controls.

It is worth further clarifying the question you raised regarding the size of different genomic regions:

a. As we constructed the Hi-C map at a resolution of 50 kb, the called TAD boundaries are fixed at 50 kb.;
b. Similarly, the window size for assigning A/B compartments was set at 50 kb. The separation score was calculated for every 50-kb sliding window throughout the entire genome;
c. Pericentromeric regions. The position of syntenic genes delimiting the GBs flanking the gene-poor regions were used to define the boundaries of pericentromeric regions, thus their sizes are not fixed values, as noted in Supplementary Table 5;
d. HOT regions. The genomic regions spanning the adjacent border genes of two rejoined blocks, along with 100-kb of flanking sequences on each side (total length = 200 kb + the inter-boundary interval). When one end of a synteny block was located within a pericentromere, the interval region often encompassed the centromere and become excessively large, potentially biasing downstream analyses. In these cases, we extracted the boundary gene of the rejoined block plus 100 kb downstream (or upstream; depending on the orientation of the blocks) sequence, along with 100 kb of flanking sequences on each side (total length = 300 kb).

These methods and strategies have been further refined and clarified in the Methods section (lines 726-761).

In summary, these analyses support the conclusion that the associations of HOT regions with pericentromeres, LTR-RT enrichments, A/B compartment transitions and TAD boundaries are statistically significant, indicating genuine enrichment rather than occurring by chance.

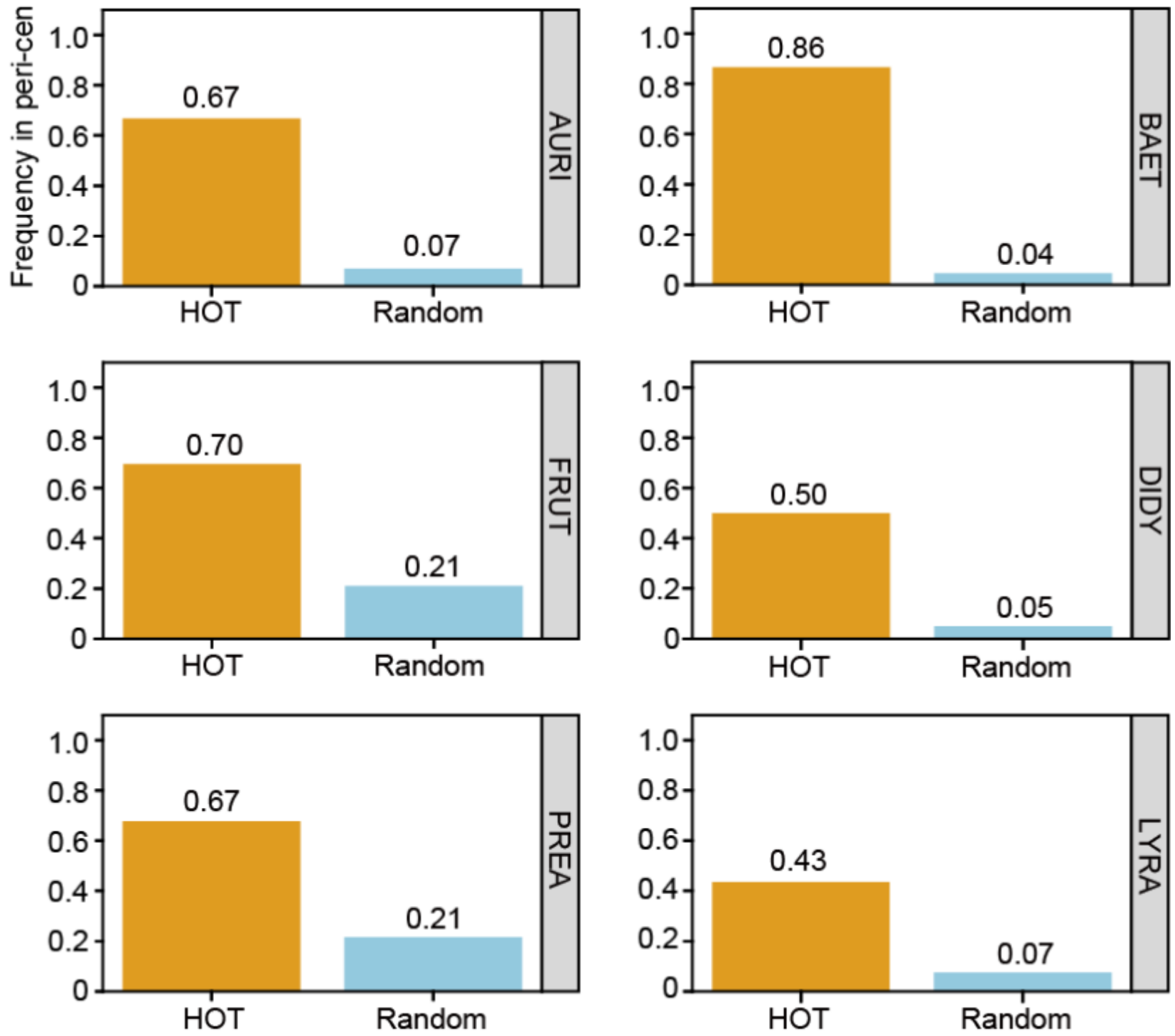

Figure R5.2 (Supplementary Fig. 21). The frequency of HOT regions aligning with pericentromeric regions (in gold), compared to that of 200 simulated 150-kb random genomic regions located within pericentromeric regions (in blue).

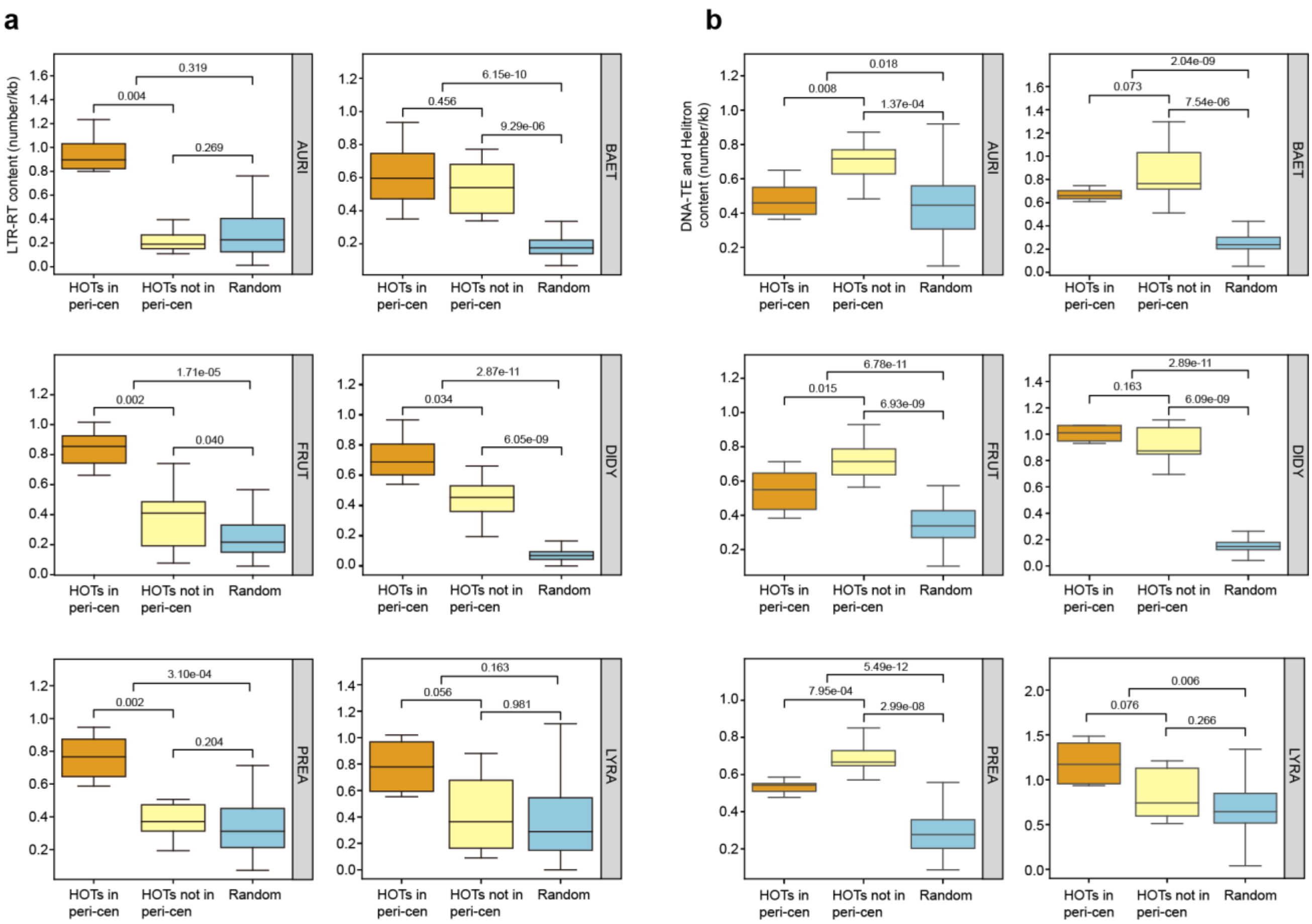

Figure R5.3 (Supplementary Fig. 23). Comparisons of LTR-RT content (a) and DNA transposon and Helitron content (b) distribution in HOT regions (gold and light yellow boxes) and 200 simulated 150-kb random regions (blue boxes; Wilcoxon rank-sum test). Based on the distribution across *Biscutella* chromosomes, HOT regions are further categorized as those located in the pericentromeric region (gold boxes) and located on the chromosomal arms (light yellow boxes; see Fig. 5b for detailed classification). While it is anticipated that pericentromeric HOTs exhibit significantly higher TE content density compared to randomly sampled chromosomal regions, our findings indicate that non-pericentromeric HOTs also generally display elevated TE accumulation.

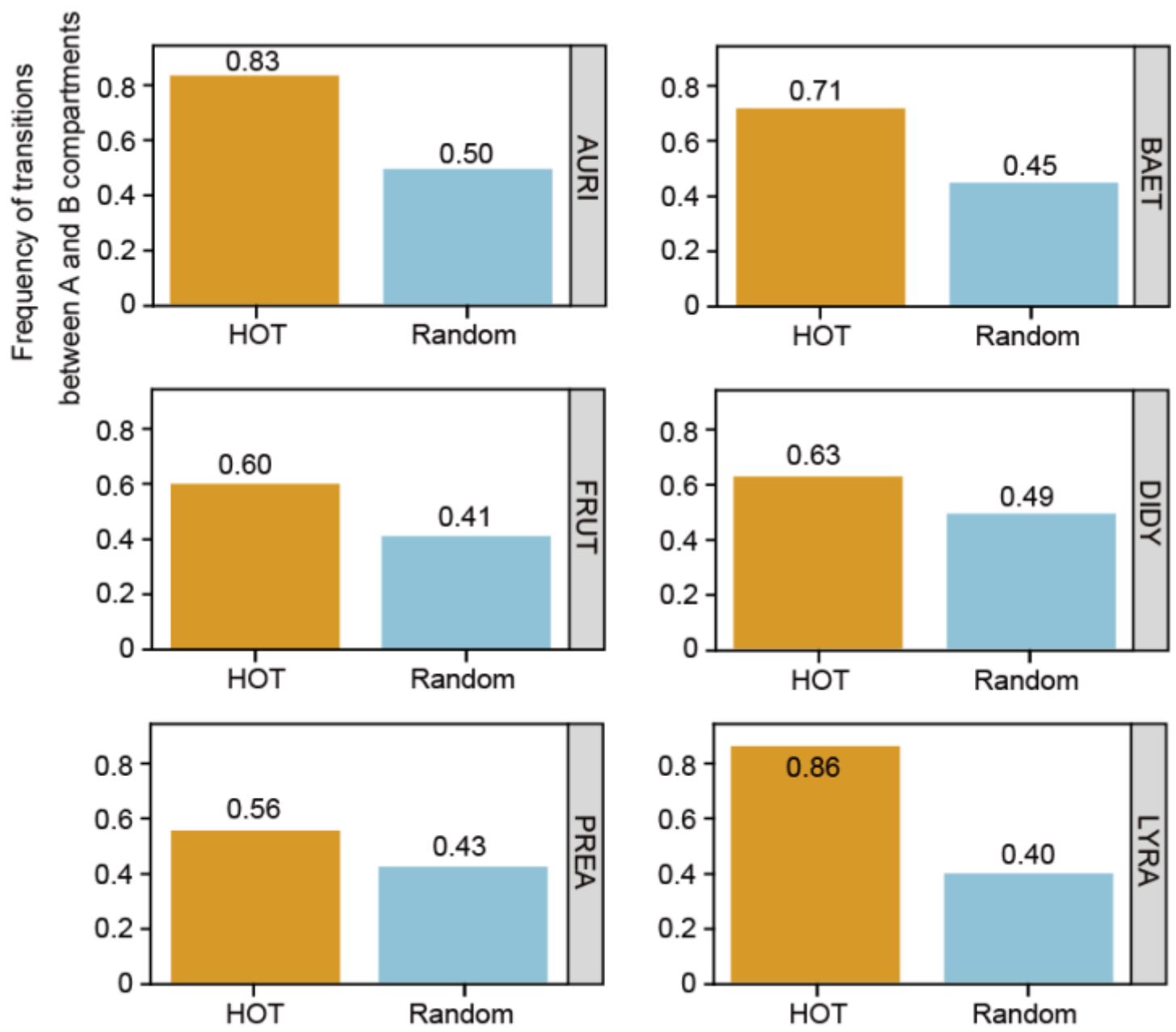

Figure R5.4 (Supplementary Fig. 25). The frequency of transitions between A and B compartments in HOT regions (in gold) with that of 200 simulated 150-kb random regions (in blue).

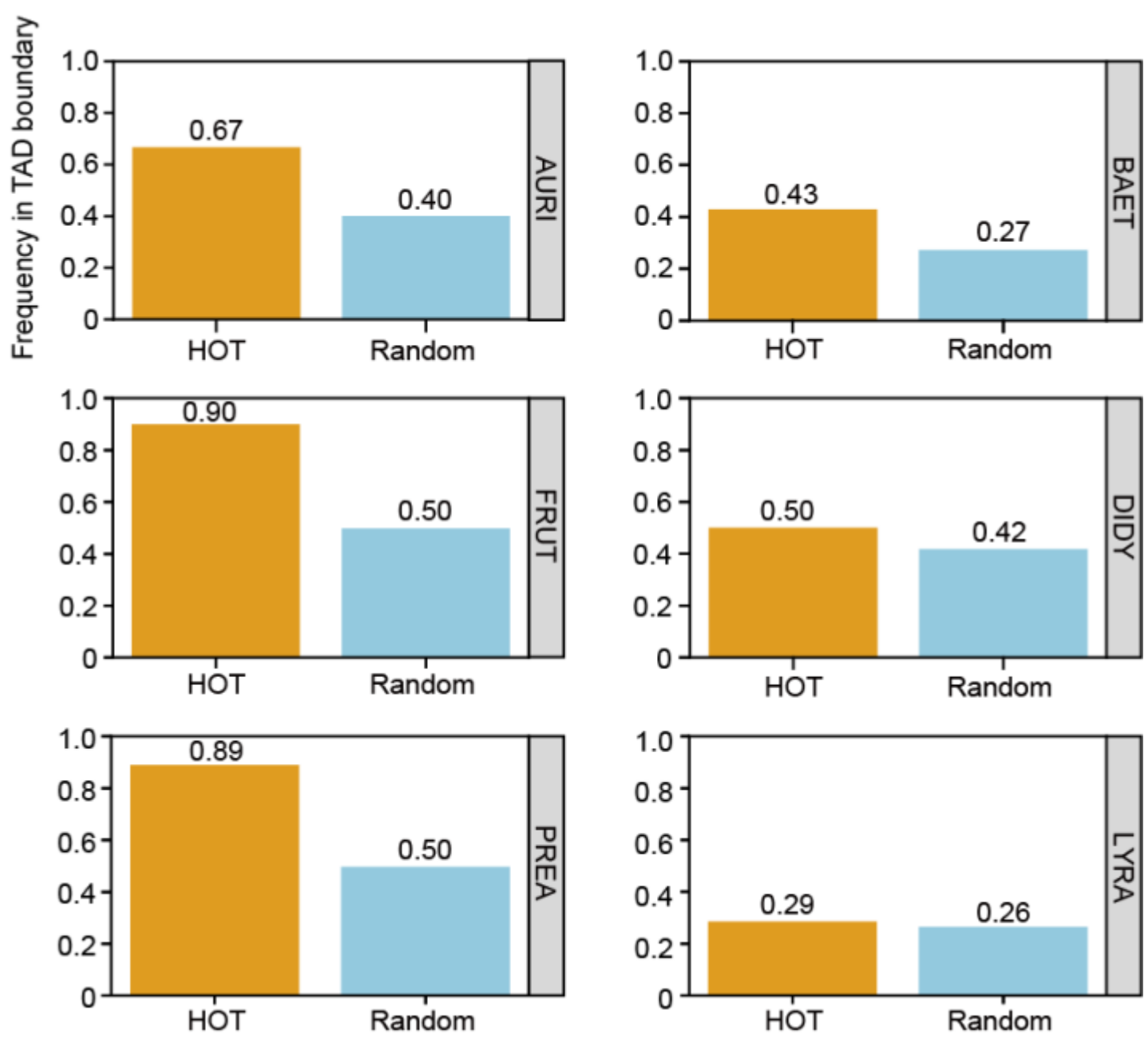

Figure R5.5 (Supplementary Fig. 26). Distribution of TAD boundaries in HOT regions (in gold) compared to 200 simulated 150-kb random regions (in blue).

R5-Q21: Line 401 "and chromosome translocations"; are you meaning "unequal chromosome translocations"? Otherwise overlap with the end-to-end translocations

A: Yes, the sentence has been revised (lines 416-417).

R5-Q22: L505: What is the reason for 2 strategies for different species and how did you choose the strategy for each particular species?

A: We utilized two different sequencing strategies due to practical and biological considerations. Firstly, our budget did not permit us to conduct high-coverage PacBio HiFi sequencing for all species. For most species, we found that ONT long-read sequencing and Hi-C sequencing provided sufficiently contiguous assemblies for our comparative analyses. However, *B. lyrata* is the only known species of *Biscutella* with a chromosome number of n = 6, making its genome structure particularly important for our evolutionary inferences. To ensure a high-quality reference assembly for *B. lyrata*, we generated additional PacBio HiFi data specifically for this species. Additionally, we encountered repeated failures with the standard Hi-C library construction for *B. lyrata* for reasons that remain unclear. Consequently, we adopted the Omni-C protocol, which allowed us to obtain a high-quality chromosome-level assembly.

R5-Q23: Lines 618-619: The methodology of how orthogroups were inferred seems to be missing.

A: Thank you for your suggestion. We have added further details and identification criteria into the revised Methods section (lines 668-671).

R5-Q24: Line 648: Formula conversion in PDF format failed.

A: This problem is related to the submission system. We will try to resolve it when submitting a new manuscript version. If it still fails, we will communicate with the editor how to solve it.

R5-Q25: Line 721: Formula conversion in PDF format failed.

A: This problem is related to the submission system. We will try to resolve it when submitting a new manuscript version. If it still fails, we will communicate with the editor how to solve it.

Reviewer #6 (Remarks to the Author):

I co-reviewed this manuscript with one of the reviewers who provided the listed reports. This is part of the Nature Communications initiative to facilitate training in peer review and to provide appropriate recognition for Early Career Researchers who co-review manuscripts.

A: Thank you very much for your time and effort!

Reviewer #7 (Remarks to the Author):

I co-reviewed this manuscript with one of the reviewers who provided the listed reports. This is part

of the Nature Communications initiative to facilitate training in peer review and to provide appropriate recognition for Early Career Researchers who co-review manuscripts.
A: Thank you very much for your time and effort!

Response to reviewers (v2)

Reviewer #1 (Remarks to the Author):

In this revised manuscript, authors addressed most of my points providing a remarkable high amount of information. There are however few answers to the main points still matter of concern for me:

1) Concerning potential different transposition rate in AURI and DYDY:

If it is assumed that solo LTRs are necessary deriving from full insertions, I do not understand why the estimation of full insertions and solo LTRs age should be different. I appreciate the time of insertion should be more precise when measured on the full size LTR elements (taking advantage of direct comparison of the two LTRs), compared than the solo LTRs. In fact, a sudden increase of LTR activity (in more recent time) might alter the S/I ratio independently by the age of solo LTR insertions (accumulated on a larger timeframe).

In my opinion, the fact that the alteration of the S/I appear to be only associated to certain TE families (figure R1.2) is in fact reinforcing the idea which is linked to transposition rate (which might be variable depending by the TE), rather than LTR-TE removal (which should happen at the same rate for all LTR-TE). In absence of different evidence or a potential misunderstand from my side of author rebuttal, I would like to have the alternative hypothesis of variable LTR-TE transposition clearly considered in the manuscript text as potential alternative explanation of the altered S/I ratio observed.

Authors (A): We sincerely appreciate the Reviewer's insightful comment!

We agree that the estimation of insertion times differs substantially between intact LTR-RTs and solo LTRs. Currently, the most widely used approach—also adopted in this study—is based on sequence divergence between paired LTRs for intact LTR-RTs, or divergence relative to a consensus sequence for truncated or solo LTRs. However, we acknowledge that this strategy has inherent limitations and that methodological differences in age estimation can introduce considerable uncertainty and noise into the inferred temporal patterns. We therefore only put the age estimates into the supplementary files (Supplementary Figure 18) for the reader's reference.

We are also grateful to the Reviewer for suggesting an alternative explanation, i.e., LTR-RT-specific transposition rates. The lineages may exhibit different activities and bursts, which can then influence the S/I LTR ratio. In addition, we note that some lineage-dependent factors may influence solo LTR dynamics, including chromosomal location of TEs (near genes, away from genes, in euchromatin, in pericentromeric heterochromatin), as well as lineage-specific differences in LTR length, which can affect the efficiency of recombination between paired LTRs.

To better reflect this complexity, we have revised the manuscript in the following ways:

(1) We have changed the term “LTR removal ratio” in the original manuscript to the actual “solo-to-intact LTR ratio”.
(2) We have removed the original explanations in the Result section and added more alternative explanations of variable transposition rates in the **Discussion** section.
(3) We have added more references supporting the discussed topic.

Original (line 38): “the early-diverging genomes showed a higher removal ratio of LTR retrotransposons and…”

Revised: “the early-diverging genomes showed a higher solo-to-intact LTR ratio of Ty3/Gypsy retrotransposons and…”

Original (line 251): “Ty3/Gypsy retrotransposons show a higher removal rate in early diverging *Biscutella* species”

Revised: “Ty3/Gypsy retrotransposons show a higher solo-to-intact LTR ratio in early diverging *Biscutella* species”

Original (lines 263-269): “Moreover, the Ty3/Gypsy lineages exhibited higher removal rates in early-diverging species than in late-diverging species, as shown by the increased solo:intact LTR ratios (S/I ratios) for Athila, CRM, Retand, and Tekay (average S/I ratio of ~0.577 in late-diverging species vs. ~1.290 in early-diverging species; Wilcoxon rank-sum *P*-value = 4.25×10^{-4}; Fig. 4b). Nevertheless, no significant differences in the S/I ratios were observed for the Ty1/Copia lineages (Fig. 4b) and between the subgenomes (Fig. 4c), indicating that the deletion of LTR-RTs (at least through soloLTR formation) is not linked to differential gene fractionation”

Revised: “Comparison of solo-to-intact LTR ratios (S/I LTR ratios) revealed significantly higher values for several Ty3/Gypsy lineages, including Athila, CRM, Retand, and Tekay, in early-diverging species compared with late-diverging species (average S/I LTR ratio of ~1.290 vs. ~0.577; Wilcoxon rank-sum *P*-value = 4.25×10^{-4}; Fig. 4b). Nevertheless, no significant differences in the S/I LTR ratios were observed for the Ty1/Copia lineages (Fig. 4b) and between the subgenomes (Fig. 4c).”

Discussion (adding after line 406): The differences in S/I LTR ratios between early- and late-diverging genomes for specific Ty3/Gypsy lineages likely reflect lineage-dependent turnover dynamics. These dynamics may result from variation in recombination-mediated removal and/or recent transposition activities[52,53]. Specifically, differences in recombination frequencies among lineages and across

chromosomal regions may lead to varying probabilities of soloLTR formation. In addition, lineage-specific transposition bursts can temporarily increase the number of intact LTR-RTs, thereby reducing S/I LTR ratios independently of removal processes.

52. Frahry, M. B., Sun, C., Chong, R. A. & Mueller, R. L. Low levels of LTR retrotransposon deletion by ectopic recombination in the gigantic genomes of salamanders. *J. Mol. Evol.* **80**, 120-129 (2015).

53. Ji, Y. & DeWoody, J. A. Genomic landscape of Long Terminal Repeat Retrotransposons (LTR-RTs) and solo LTRs as shaped by ectopic recombination in Chicken and Zebra Finch. *J. Mol. Evol.* **82**, 251-263 (2016).

2) Concerning the breakage hotspots (HOTs)

In my understanding authors have confirmed that more recent TE activity (compared to the WGD) could have masked potential evidence of TE accumulation at HOTs when Biscutella speciation occurred. Therefore, most of insertion find at HOTs indeed occurred after chromosomal rearrangements. I am therefore surprised by the relatively mild revision of the text occurred at lines 310-312. I think the text still exclude (or avoid to mention) the idea, still very plausible to me, which are HOT regions that attract (or maintain) TE insertions, rather than TE accumulation favouring rearrangement. It could be simply because these highly variable regions do not contain regulatory or coding DNA (so that rearrangements are mostly under neutral selection) or maybe are characterised by specific chromatin arrangements which favour both recombination and TE insertion. Again, also in this case I would specifically mention about this alternative hypothesis, if authors cannot rule out the possibility.

A: Thank you for bringing this up again. Given that the current evidence does not yet allow conclusions to be drawn about any of the hypotheses, we have deleted the explanations in the Result section (lines 309-312) and moved the interpretations and alternative hypotheses to the **Discussion** section.

Original Discussion (lines 425-428): “We identified 14 chromosome breakage hotspots (HOTs) shared by several *Biscutella* species. These regions exhibit enriched LTR-RTs, sustaining loci prone to frequent DSBs and NAHR, as evidenced by paleocentromeric HOTs enriched in Athila and CRM elements, still detectable at chromosomal junctions.”

Revised: “We identified 14 chromosome breakpoints that are shared by several *Biscutella* species. These regions are enriched with TEs and potentially prone to frequent DSBs and NAHR, a pattern supported by paleocentromeric breakpoints enriched in Athila and CRM elements, which can still be detected at some chromosomal junctions (Fig. 5c and Supplementary Fig. 22). Given the relatively recent activity of TEs, these breakpoints may also represent intrinsically dynamic chromosomal regions that preferentially retain or tolerate TE insertions. In this scenario, recurrent rearrangements and TE accumulation are likely parallel outcomes of intrinsic chromosomal instability rather than a direct causal

relationship."

Reviewer #2 (Remarks to the Author):

I would like to thank the authors for their thorough and thoughtful responses to my previous comments. They have addressed all of my concerns meticulously, and the manuscript has been significantly improved as a result. I have no further suggestions and recommend acceptance.

A: Thank you!

Reviewer #5 (Remarks to the Author):

Reply on rebuttal "Post-polyploid chromosomal diploidization in plants is affected by clade divergence and constrained by shared genomic features"; The authors have addressed our remarks in a thoughtful and detailed manner, which is highly appreciated. I believe they successfully addressed our remarks, and, where necessary, adapted the manuscript. By performing additional analyses (namely analysis of the evolution of breakpoints) and expanding the discussion, I believe the manuscript has been strengthened. As indicated in the first review round, this paper will be of considerable significance for the field of polyploidy and diploidization. Nonetheless, I still have a few remarks:

A: We appreciate the reviewers for taking the time to critically evaluate and revise our manuscript!

Remarks on the hotspot part:

1. You categorize hotspots into ancestral and recurrent "hotspots";. However, the ancestral hotspots indicate that only one chromosome junction happened which was inherited in multiple species, while a "hotspot"; implies that it happened multiple times in this region. Consider renaming the ancestral hotspot category.

A: Thank you for your suggestion, which we followed. We have renamed the original "HOTs" as "breakpoints", and indicated which are ancestral and which are recurrent.

2. The authors use a simulation-based approach to assess significance of overlap between HOT regions and other genomic features (pericentromere, TAD boundaries, A/B compartment boundaries), which is justified. In Lines 756-759, the authors indicate that HOT regions encompass typically two rejoined junctions that have a typical length of 300kb and that is the reason for the choice of the 150 kb random segments. This only makes sense if the random segments are 200 combination of two 150kb segments but this does not seem to be mentioned? Either way, I think it would be better to simulate using the exact

sizes of the identified HOT regions (or the sizes of the identified joined segments that make up the HOT region), so that you can really rule out effect of size of a HOT region. If not, could you clarify further how the 150kb was choosen?

A: In the previous version of our manuscript (and also in the first rebuttal letter), we indeed performed simulations using 200 random combinations of 150-kb windows. We greatly appreciate Reviewer's insightful suggestion regarding real size-matched simulation designs. We followed Reviewer's suggestion and changed our simulation strategy to use size-matched random segments corresponding to the exact lengths of the identified breakpoint regions (formerly referred to as HOTs; see Q1). Each full simulation process was independently replicated three times to ensure robust results. We got basically the same results as before. This revised approach and the corresponding results are now described in the Methods section and presented in revised Supplementary Figures 21, 23, 24, and 25 (as shown below).

Revised figures:

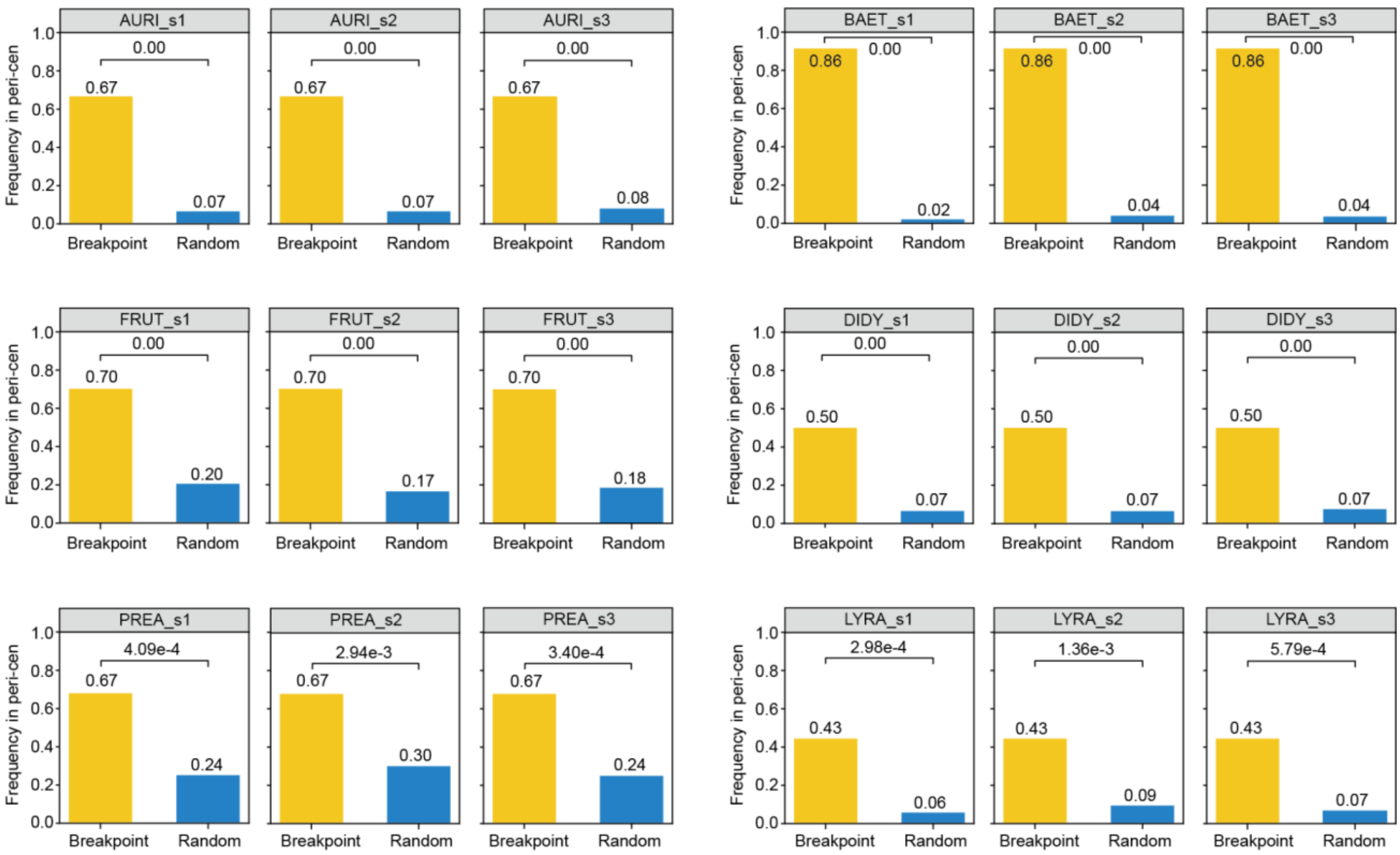

Supplementary Fig. 21 | Frequency of overlap between (peri)centromeres and two sets of genomic regions: breakpoint regions (yellow bar, abbreviated as Breakpoint) and 200 simulated regions randomly sampled from the whole genome (blue bar, abbreviated as Random). Three independent simulation runs were carried out for each genome (s1, s2, and s3). Individual subplot *P*-values were calculated via Fisher's exact tests. Across all simulations, the frequency of breakpoint regions overlapping (peri)centromeres was significantly higher than that of random regions (*P*-value = 1.94×10^{-4}; Wilcoxon signed-rank test).

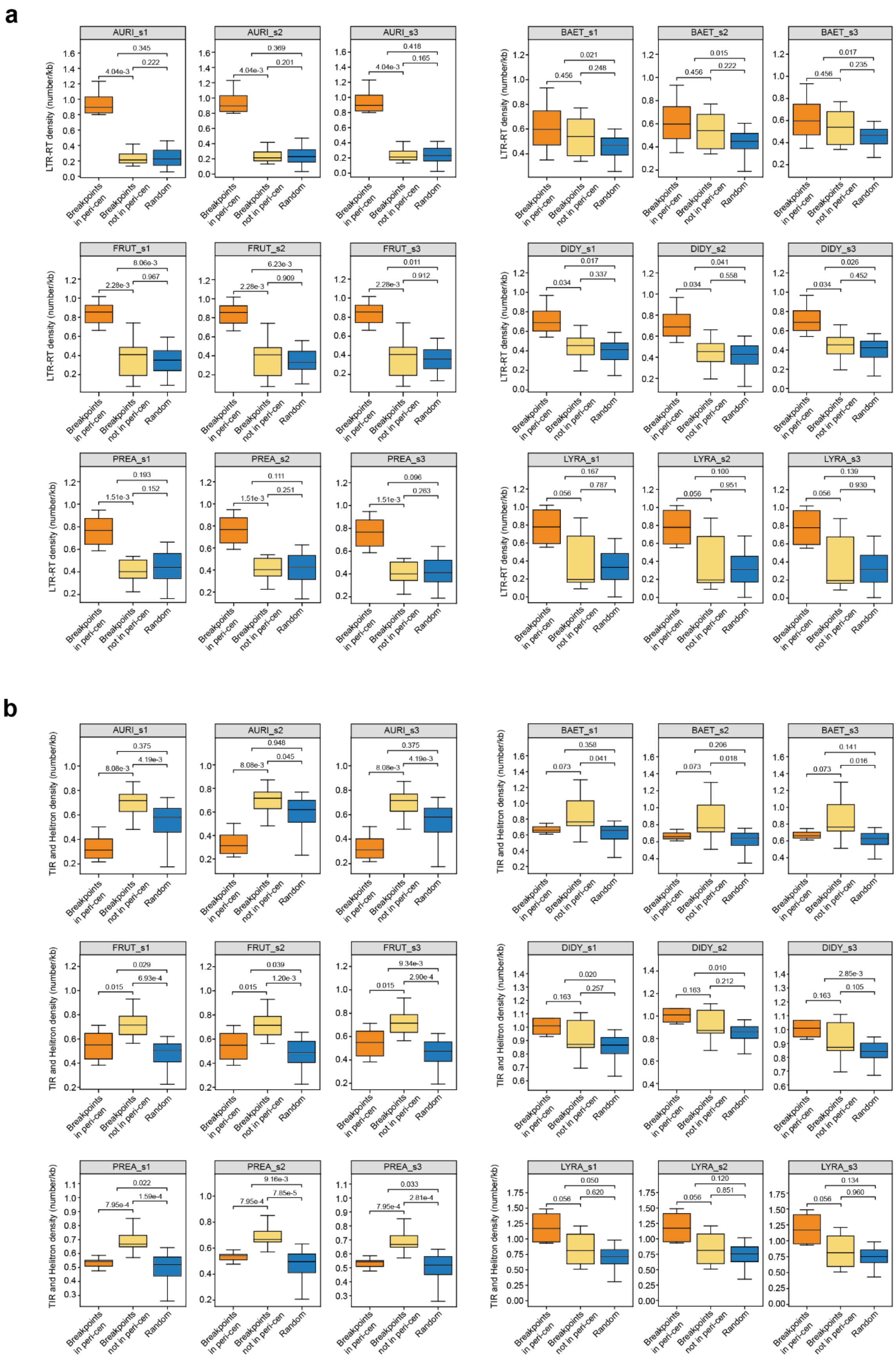

Supplementary Fig. 23 | Comparisons of LTR-RT density (**a**), TIR and Helitron density (**b**) distribution in the

breakpoint regions (orange and light yellow boxes) and 200 simulated random regions (blue boxes; Wilcoxon rank-sum test). Three independent simulation runs were carried out for each genome (s1, s2, and s3). The breakpoint regions were categorized as located in the (peri)centromeres (orange boxes) and those located within chromosome arms (light yellow boxes; see Fig. 5b for detailed classification).

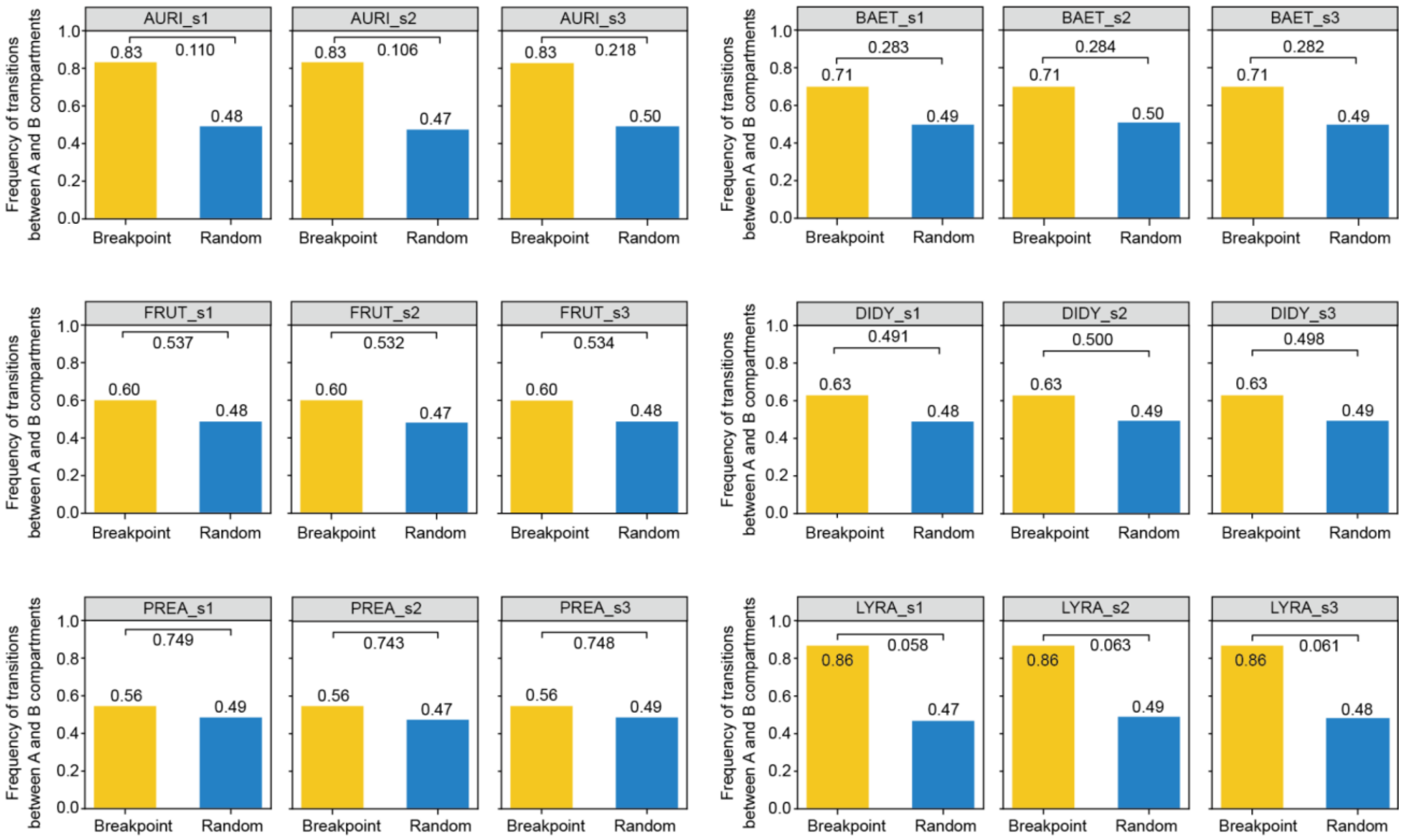

Supplementary Fig. 24 | The frequency of transitions between A and B compartments in the breakpoint regions (yellow bar, abbreviated as Breakpoint) compared to that of 200 simulated random genomic regions (blue bar, abbreviated as Random). Three independent simulation runs were carried out for each genome (s1, s2, and s3). Individual subplot *P*-values were calculated via Fisher's exact tests. Across all simulations, the frequency of transition between A and B compartments in breakpoint regions was significantly higher than that of random regions (*P*-value = 1.95×10^{-4}; Wilcoxon signed-rank test).

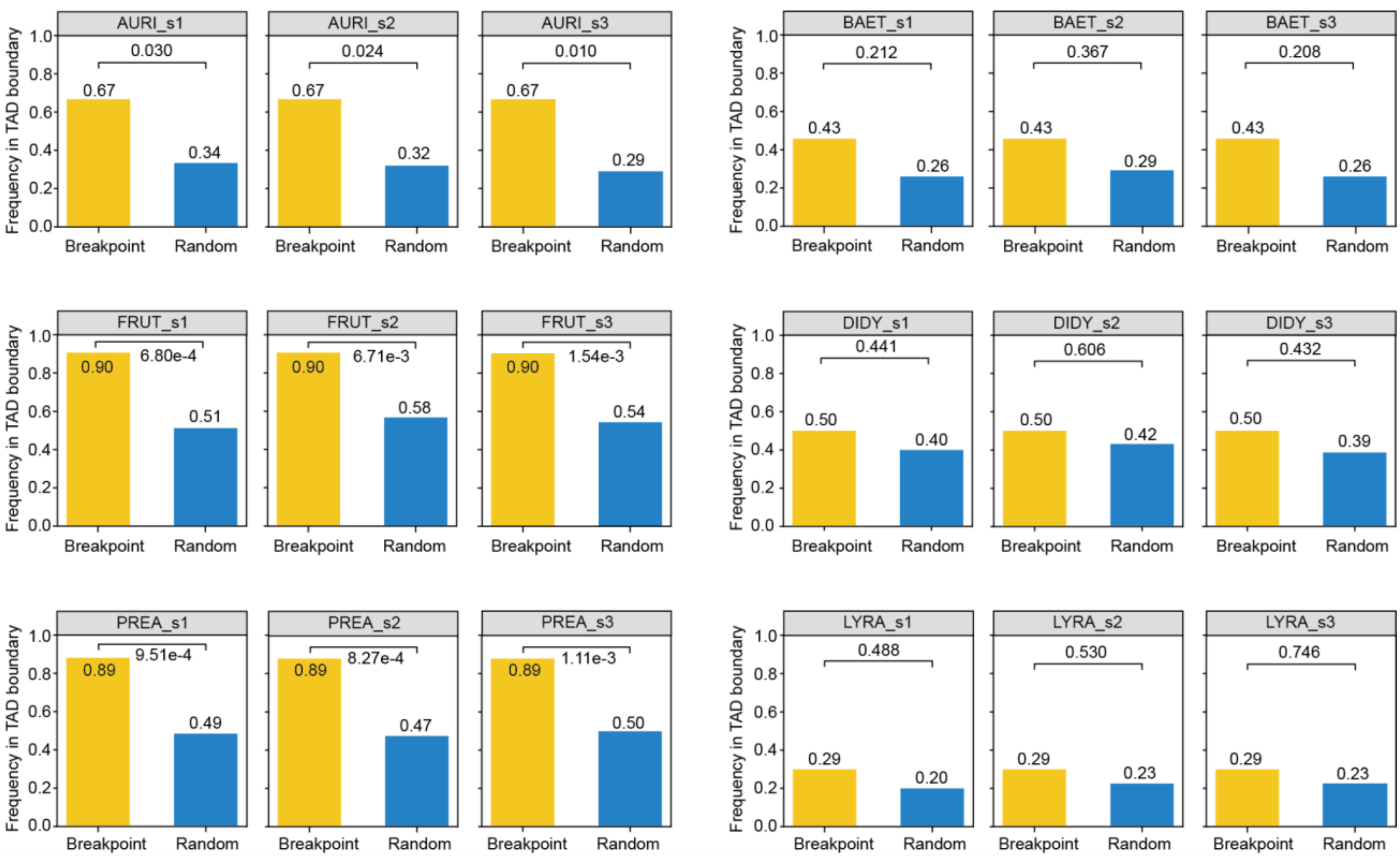

Supplementary Fig. 25 | Comparisons of TAD boundaries distribution in breakpoint regions (yellow bar, abbreviated as Breakpoint) and in 200 simulated random genomic regions (blue bar, abbreviated as Random). Three independent simulation runs were carried out for each genome (s1, s2, and s3). Individual subplot *P*-values were calculated via Fisher's exact tests. Across all simulations, the frequency of TAD boundaries distribution in breakpoint regions was significantly higher than that of random regions (*P*-value = 1.95×10^{-4}; Wilcoxon signed-rank test).

3. You show the results of these simulations by comparing patterns your identified HOT regions to an expected average pattern in the simulated segments. However, you do not compare statistically, which would involve variation in both observed and random expected, even though you mention in your response to my previous comment that differences are "statistically significant". You test significance in in Supp figure 23, but not in 21, 25 and 26. While the association with pericentric regions are convincing, those with TAD and A/B are less so.

A: We sincerely appreciate Reviewer's comment. To address this concern, we have supplemented Fisher's exact tests (suited for small sample sizes and 2×2 contingency table analyses) for the datasets presented as Supplementary Figures 21, 23, 24, and 25 (see these supplementary figures under Q2 above). In addition, we performed three independent simulation runs to ensure the reliability of our findings.

While the Fisher's exact test results did not reach statistical significance in certain cases (e.g., Supplementary Figure 24: A/B compartment transitions and Supplementary Figure 25: TAD boundaries

distribution in BAET, DIDY, and LYRA), the three independent simulations consistently yielded the same qualitative trends of difference between the breakpoint regions and random sampled regions (which we believe is more important). To validate these trends further, we conducted Wilcoxon signed-rank tests (a non-parametric statistical test used to compare paired samples) to compare the replicate results for the breakpoint and random regions across all species. All the *P*-values were extremely significant. Collectively, we believe that the consistent directional trends across replicates, combined with the significant results of the Wilcoxon signed-rank tests, provide robust support for the conclusions presented.

Small remarks:

Line 59-60: I do agree with the authors that descending dysploidy can occur independently from a WGD event. What I meant with the previous remark "Is diploidizing descending dysploidy not by definition after WGD events?"; is that adding the "diploidizing"; in front of the term already implies that it is following a WGD. I think the sentence would be more accurate if phrased as e.g. "Descending dysploidy is particularly common after WGD events (i.e., diploidizing descending dysploidy)";

A: We agree. What we meant by "diploidizing" here is that descending dysploidy after WGDs contributes to the **diploidization** process. To avoid confusion, we modified this sentence to "Descending dysploidy…" (omitting "diploidizing" as being redundant in the given context).

Line 140-141: I think the fact that it supports the reliability of gene annotations is based on the fact that you get the expected 2:1 pattern given its meso-tetraploid ancesstry. I think, in that case, it would be better to put these lines after the lines about its meso-tetraploid ancestry where the 2:1 pattern is explained.

A: Thank you. We modified this passage accordingly.

Original (Lines 140-141): "Utilizing these syntenic gene pairs as pillars, we identified two genomic copies for nearly all 22 GBs (Supplementary Fig. 4), supporting the reliability of the gene annotations. An exception was block G in *B. prealpina* and *B. varia*, where only one copy was detected, likely due to extensive gene loss or misassembly in these regions. The overall 2:1 syntenic relationship between the *Biscutella* genomes and the 22 GBs confirms a meso-tetraploid WGD predating diversification of the genus."

Revised: "Utilizing these syntenic gene pairs as pillars, we identified two genomic copies for nearly all 22 GBs (Supplementary Fig. 4). An exception was block G in *B. prealpina* and *B. varia*, where only one copy was detected, likely due to extensive gene loss or misassembly in these regions. The overall 2:1 syntenic relationship between the *Biscutella* genomes and the 22 GBs confirms a mesotetraploid WGD predating diversification of the genus and further supports the reliability of the gene annotations."

Line 212: If you see the X+Q+Wa association it could also support the opposite claim that it does not support the shared paracentric inversion event. Nonetheless, I agree with the authors that the presence of the complete V+Wa+Q+X association in most of the species on the LF sub-genome and all species on the MF sub-genome supports the shared hypothesis.

A: Thank you for your comment. Our inference for this event is based on the fact that the complete V+X+Q+Wa association is present in the LF subgenome of some species and in the MF subgenome of all species. If the "opposite claim" you mentioned would be true, i.e., that the event did not occur or only occurred in the MF subgenome, then it is unlikely that we would have detected the V+X+Q+Wa association in the LF subgenome as well.

For further clarification, we have added more details in the main text:

Original (Lines 210-214): "A shared paracentric inversion event (Ipa), which restructured the ancestral chromosome AK8/6 by rearranging the V+Wa+Q+X association into V+X+Q+Wa, is supported by the presence of the derived V+X+Q+Wa or X+Q+Wa association in both subgenomes of all *Biscutella* species, as well as in two other Biscutelleae species – *M. pygmaea* and *H. bupleurifolia* (Fig. 3a and Supplementary Fig. 13)."

Revised: "A shared paracentric inversion (Ipa) predating the speciation of *Biscutella*, *M. pygmaea* and *H. bupleurifolia* restructured the ancestral chromosome AK8/6 by rearranging the V+Wa+Q+X association into V+X+Q+Wa. This event is supported by the presence of the derived V+X+Q+Wa association in the MF subgenome of all *Biscutella* species and in the LF subgenome of three species (*B. auriculata*, *B. baetica*, *B. didyma*). The four n = 9 species and *B. lyrata* retained a truncated X+Q+Wa association in the LF subgenome, resulting from subsequent rearrangements that disrupted the V+X association (Fig. 3a and Supplementary Fig. 13).

Line 748: "the interval region often encompassed the centromere and excessively large, which could bias downstream analyses"; The "and excessively large" seems out of place or missing a part in this sentence.

A: Thank you! We have revised this part.

Original (Lines 747-751): "When one end of a synteny block was located in a pericentromeric region, the interval region often encompassed the centromere and excessively large, which could bias downstream analyses. In these cases, we extracted the boundary gene of the rejoined block plus a 100-kb downstream (or upstream; depending on the orientation of the blocks) sequence, along with 100-kb of flanking sequences on each side (total length = 300-kb)."

Revised: When one end of a synteny block was located in a (peri)centromeric region, we extracted the boundary gene of the rejoined block plus a 100-kb downstream (or upstream; depending on the

orientation of the block) sequence, along with 100 kb of flanking sequences on each side (total length = 300 kb).

Line 763: Could you clarify whether these orthologous gene families identified with OrthoFinder or SynOrths?

A: Thank you. This is now clarified.

Original (line 763): "We classified orthologous gene families across eight *Biscutella* species into core, softcore, and private categories."

Revised: "We classified orthologous gene families identified by SynOrths across eight *Biscutella* species into core, softcore, and private categories."

Supplementary figure 23: "our findings indicate that non-pericentromeric HOTs also generally display elevated TE accumulation"; You show it convincingly for the DNA-TE and helitron, but only in 2 of 6 species for LTR-RT. Consider toning down or specifying the conclusion on the TE association.

A: Thank you very much for your suggestion. We acknowledge that for both simulation strategies (fixed-size or matched-size windows, see Q2), breakpoint regions do not show a (statistically significant) uniform pattern across random regions in all genomes. For this reason, we intentionally avoided framing these associations in terms of "universal statistical significance", as consistent enrichment is not observed across all TE families and species (for example, LTR-RTs you mentioned). To improve clarity, we reduced the emphasis on the association between breakpoints and TEs.

Specifically:

(1) We have removed the relevant statement in the Supplementary Fig. 23.
(2) We have removed the explanation on the association between breakpoints and LTR-RTs from the main text (original lines 309-312).
(3) We have changed the heading of this section.

Original (Line 294): Chromosome breakage hotspots are associated with LTR-RT enrichment and changes in 3D genome organization

Revised: Chromosome breakpoints are associated with changes in 3D genome organization

Reviewer #6 (Remarks to the Author):

I co-reviewed this manuscript with one of the reviewers who provided the listed reports. This is part of the

Nature Communications initiative to facilitate training in peer review and to provide appropriate recognition for Early Career Researchers who co-review manuscripts.

A: Thank you!

Reviewer #7 (Remarks to the Author):

I co-reviewed this manuscript with one of the reviewers who provided the listed reports. This is part of the Nature Communications initiative to facilitate training in peer review and to provide appropriate recognition for Early Career Researchers who co-review manuscripts.

A: Thank you!

REVIEWERS' COMMENTS

Reviewer #5 (Remarks to the Author):

We thank the authors of the manuscript “Post-polyploid chromosomal diploidization in plants is affected by clade divergence and constrained by shared genomic features” for taking our and the other reviewers feedback at heart. We feel that the manuscript is ready for acceptance except for one of the added statistical tests (Wilcoxon signed rank). We outline our problem with it in a comment below but suggest to just remove it and tone down the conclusion about association with TAD boundaries and A/B transitions.

Authors (A): We really appreciate you taking the time to share your thoughts.

308-311: “Analysis of the LTR-RT density in 100-kb regions on each side of rejoined junctions showed that more than 97% of these regions had higher values than the genome-wide average, and around half of them higher than expected from random controls (Supplementary Figs. 22, 23 and Supplementary Table 11).” The 97% percent comes from 46/47 breakpoint regions seen in fig. S22. This suggests the breakpoint regions are independent, which they are not because of phylogenetic relatedness. This statement is also a bit misleading since the number of regions with higher LTR-RT density than the average does not say anything about how much more LTR-RT you can find there compared to the random expected.

A: In fact, these breakpoints are not entirely independent; we have already distinguished between recurrent and ancestral breakpoints (in Fig.5a). We agree with your point that a more complex mathematical model may be needed in the future to more accurately characterize the relationship between these breakpoints and various features. Given that previous reviewers raised similar concerns regarding LTR-RT density, we have removed this section and not emphasize the relationship between breakpoints and LTR-RT density.

From the response: “While the Fisher’s exact test results did not reach statistical significance in certain cases (e.g., Supplementary Figure 24: A/B compartment transitions and Supplementary Figure 25: TAD boundaries distribution in BAET, DIDY, and LYRA), the three independent simulations consistently yielded the same qualitative trends of difference between the breakpoint regions and random sampled regions (which we believe is more important). To validate these trends further, we conducted Wilcoxon signed-rank tests (a non-parametric statistical test used to compare paired samples) to compare the replicate results for the breakpoint and random regions across all species. All the P-values were extremely significant. Collectively, we believe that the consistent directional trends across replicates, combined with the significant results of the Wilcoxon signed-rank tests, provide robust support for the conclusions presented.” I appreciate that the authors provide Fisher’s exact test significance scores for the other plots. However, the authors now also mention a Wilcoxon signed-rank test that shows significance across all species in both A/B and TAD association with breakpoint regions. There is very

little explanation given here as to what exactly is tested. Most of it I derived from their response, but with (as far as I could find) no real explanation in the manuscript. Therefore, readers will have a hard time understanding this analysis.

A: Thank you for your comment. We have added more detailed explanations in the Methods section and in the legends of corresponding Supplementary Figures (revised Supplementary Figs 21-23).

I am going of the assumption that the authors performed the test on each pair of summary frequencies of A/B transitions in (1) breakpoint vs (2) the random segments of equal length from that genome, i.e., for every panel of fig. S24 (and similarly for S25). This seems like a solution to find support for the intuition that for each species the association for both A/B and TAD with the breakpoint regions is more likely than in the random expectation but without sufficient support within each species individually. I find this unsatisfactory. Apart from problems with non-parametric tests, if I understood what you did correctly, the input for these tests include re-using the same frequency of each species multiple (3) times, which again means that the tested data is not independent (hypothetically, 1 out of 6 species having an extreme frequency may be randomness in a small sample but repeat each observation three times and you have 3 out of 18 which seems more "significant"). The species are furthermore also related which means that in this case, TAD or A/B associations may be inherited from a common ancestor which means there is a phylogenetic signal and therefore additional non-independence.

To pool these data and simulated random expectation across all species, you would need a more complicated model I would think (at first glance, a binomially distributed model with the number of TAD associated regions as outcome across total number of regions as draws, and with being breakpoint or simulation and species as predictor and with the phylogenetic signal also as covariate). I will not propose that you do this, because of the developed stage of the manuscript. I would suggest you tone down statements where you claim associations with A/B and TAD: that for each species there is not sufficient evidence but that the higher frequency than the random expectation is found in all species, suggesting some association. E.g. "We found that up to 68% of the breakpoint junctions showed inconsistent A/B compartment assignments and 64% matched TAD boundaries across Biscutella genomes (Fig. 5b). Both of these values are significantly higher than expected from 200 random simulations (Supplementary Figs. 24, 25)." This statement is not factual, since you did compare a set of 200 simulations with all breakpoints that are 68% associated with A/B transitions.

A: We appreciate your insightful comments and agree with your point that a more sophisticated model/algorithm will be needed in the future to study these breakpoints. To soften this argument, we have made the following revisions:

Original lines 315-318: We found that up to 68% of the breakpoint junctions showed inconsistent A/B compartment assignments and 64% matched TAD boundaries across *Biscutella* genomes (Fig. 5b). Both of these values are significantly higher than expected from 200 random simulations (Supplementary Figs. 24, 25).

Revised Text: Among the 47 shared breakpoints present in the *Biscutella* genomes, 32 showed inconsistent A/B compartment assignments and 30 matched TAD boundaries (Fig. 5b). **Although statistical support within individual species is limited,** both proportions consistently higher than expected from 200 random simulations (Supplementary Figs. 22, 23), suggesting **a potential association** between breakpoints and 3D genome organization.

Fig. S23 and Fig. S24: caption does not mention the Fisher's exact test. Neither does the methods section

A: We have added more detailed explanations in the Methods section and in the legends of corresponding Supplementary Figures (revised Supplementary Figs 21-23).
